# Supplementary material for: DELE1 maintains muscle proteostasis to promote growth and survival in mitochondrial myopathy
Source: EMBO J. 2024 Oct 8;43(22):5548–85. doi: 10.1038/s44318-024-00242-x (PMC11574132; doi:10.1038/s44318-024-00242-x)
Supplement: Supplementary file 1 — Appendix [file 44318_2024_242_MOESM1_ESM.pdf]

Appendix

DELE1 maintains muscle proteostasis to promote growth and survival in mitochondrial myopathy

Hsin-Pin Lin[1], Jennifer D. Petersen[1], Alexandra J. Gilsrud[1], Angelo Madruga[1], Theresa M. D'Silva[1], Xiaoping Huang[1], Mario K. Shammas[1], Nicholas P. Randolph[1], Kory R. Johnson[2], Yan Li[3], Drew R. Jones[4], Michael E. Pacold[5,6], Derek P. Narendra[1*]

Appendix Figure S1: **2 - 3**

Appendix Figure S2: **4 - 5**

Appendix Figure S3: **6 - 7**

Appendix Figure S4: **8 - 9**

Appendix Figure S5: **10 - 11**

Appendix Figure S6: **12 - 13**

Appendix Figure S7: **14 - 15**

Appendix Figure S8: **16 - 17**

Appendix Figure S9: **18 - 19**

Appendix Figure S10: **20 - 21**

Appendix Figure S11: **22 - 23**

Appendix Figure S12: **24 - 25**

Appendix Figure S13: **26 - 27**

Appendix Figure S14: **28 - 29**

Appendix Figure S15: **30 - 31**

Appendix Figure S16: **32 - 33**

Appendix Figure S17: **34 - 35**

Appendix Figure S1

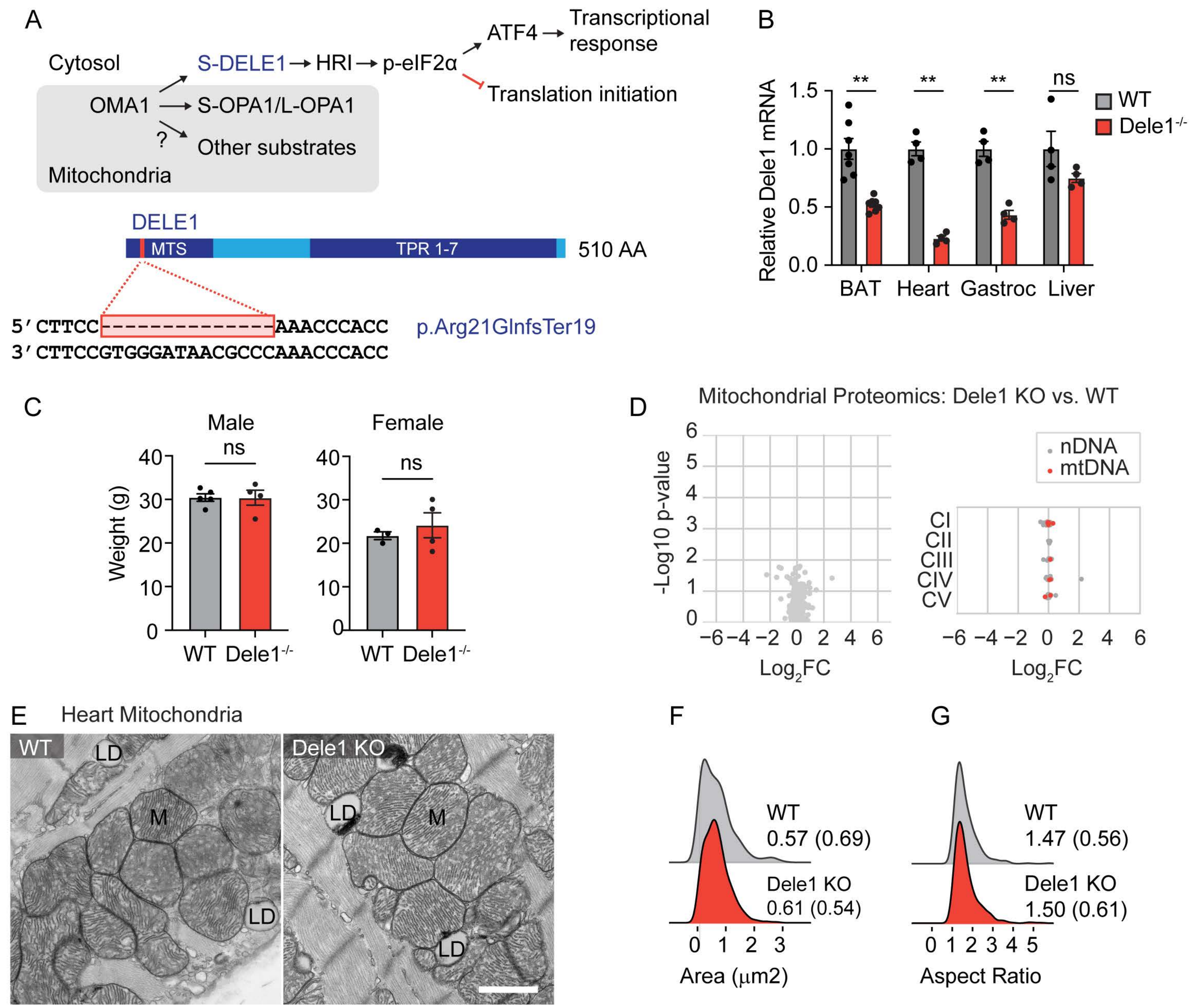

Appendix Figure S1. OMA1-DELE1 signaling mediates the integrated stress response under physiologic cold stress.

(A) Diagram depicting the OMA1 stress signaling from mitochondria through cleavage of DELE1, activating the integrated stress response (ISR), and other substrates such as the inner mitochondrial membrane fusion protein OPA1 (top); and diagram of novel *Dele1* KO mouse, depicting the 510 amino acid (AA)-long DELE1 protein with mitochondrial targeting sequence (MTS) and tetratricopeptide repeat (TPR)-domains. The red bar indicates the site of 14 bp deletion in *Dele1*, resulting in a frameshift mutation (p.Arg21GlnfsTer19) and early termination (bottom).

(B) *Dele1* mRNA levels measured from microarray experiments of *Dele1* KO mice vs. WT littermates in four tissues indicated (BAT, brown adipose tissue; Gastroc, gastrocnemius muscle). Data from these microarray experiments also appears in (Figure 2E, 4B, and 4G). N= 4 for all groups except for C10 G58R; Dele1+ liver, which was N=3.

(C) Weights of Dele1 KO mice vs. WT littermates at 4 – 6 months of age. Statistical analysis performed using Welch's t-test.

(D) Volcano plot represents all mitochondrial proteins (in MitoCarta3) measured from crude mitochondrial fraction of *Dele1* KO vs. WT littermates by mass spectrometry (left) and scatterplot depicting relative abundance of OXPHOS complexes I – V subunits. No mitochondrial proteins reached significance (data also in Table S3). Statistical testing performed as described in methods for proteomics data. WT group also is control for proteomics experiments in (Figure 2G, 2I, 4E, and 4G). N = 4 mice per group.

(E) Representative TEM images of mitochondria from hearts of P28 *Dele1* KO and WT littermate mice. Scale bar = 1 μm; M = mitochondria; LD = lipid droplet.

(F - G) Quantification of mitochondrial area and aspect ratio from TEM images like those shown in (E). Median values with interquartile range are shown.

Appendix Figure S2

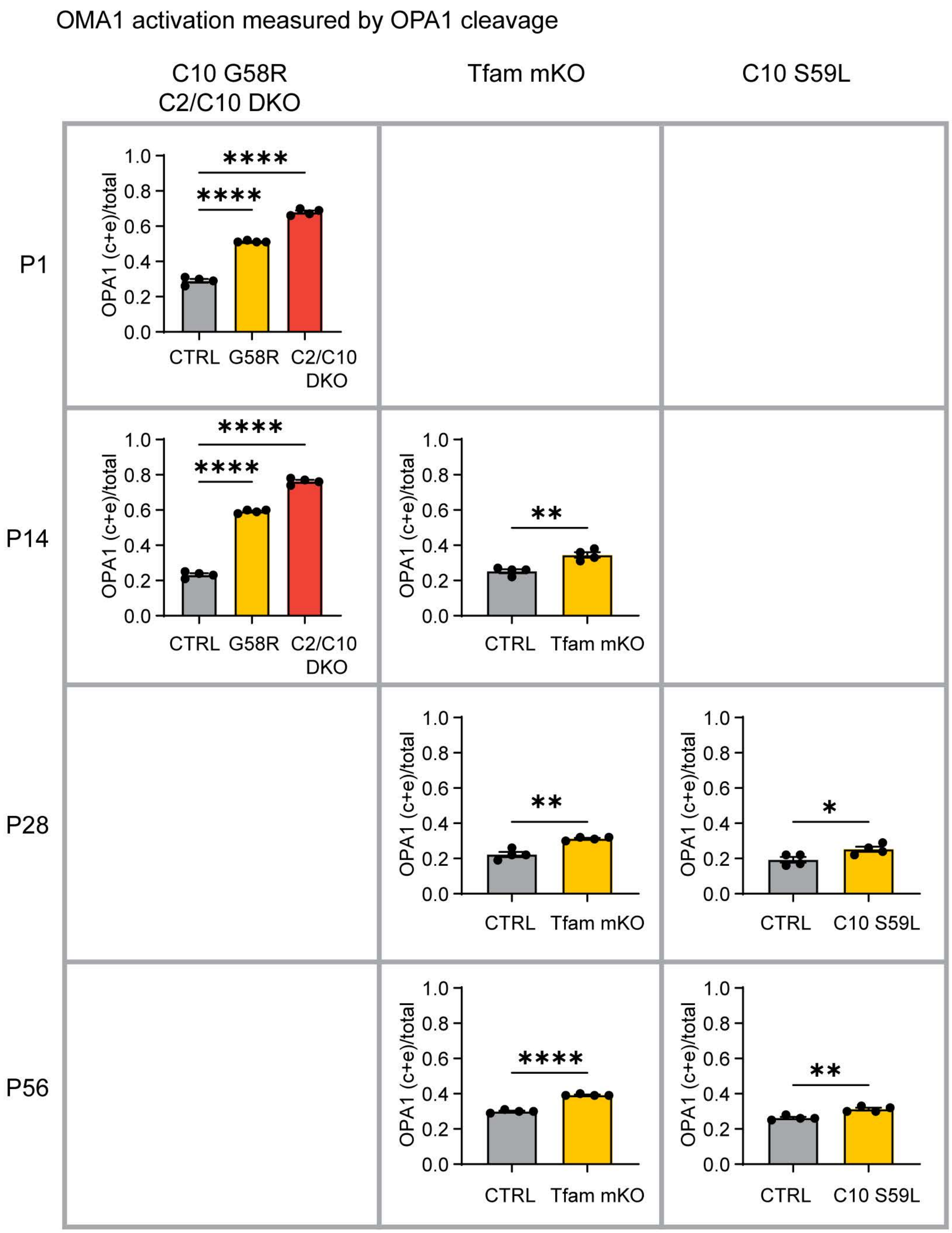

Appendix Figure S2. OMA1 activation in four models of myopathy/cardiomyopathy measured from L-OPA1 cleavage. Quantification of blots from Figure 1M. Statistical analysis performed with ordinary one-way ANOVA with Holm-Šidák's correction for multiple comparisons (left two graphs) and unpaired t test (other graphs).

Appendix Figure S3

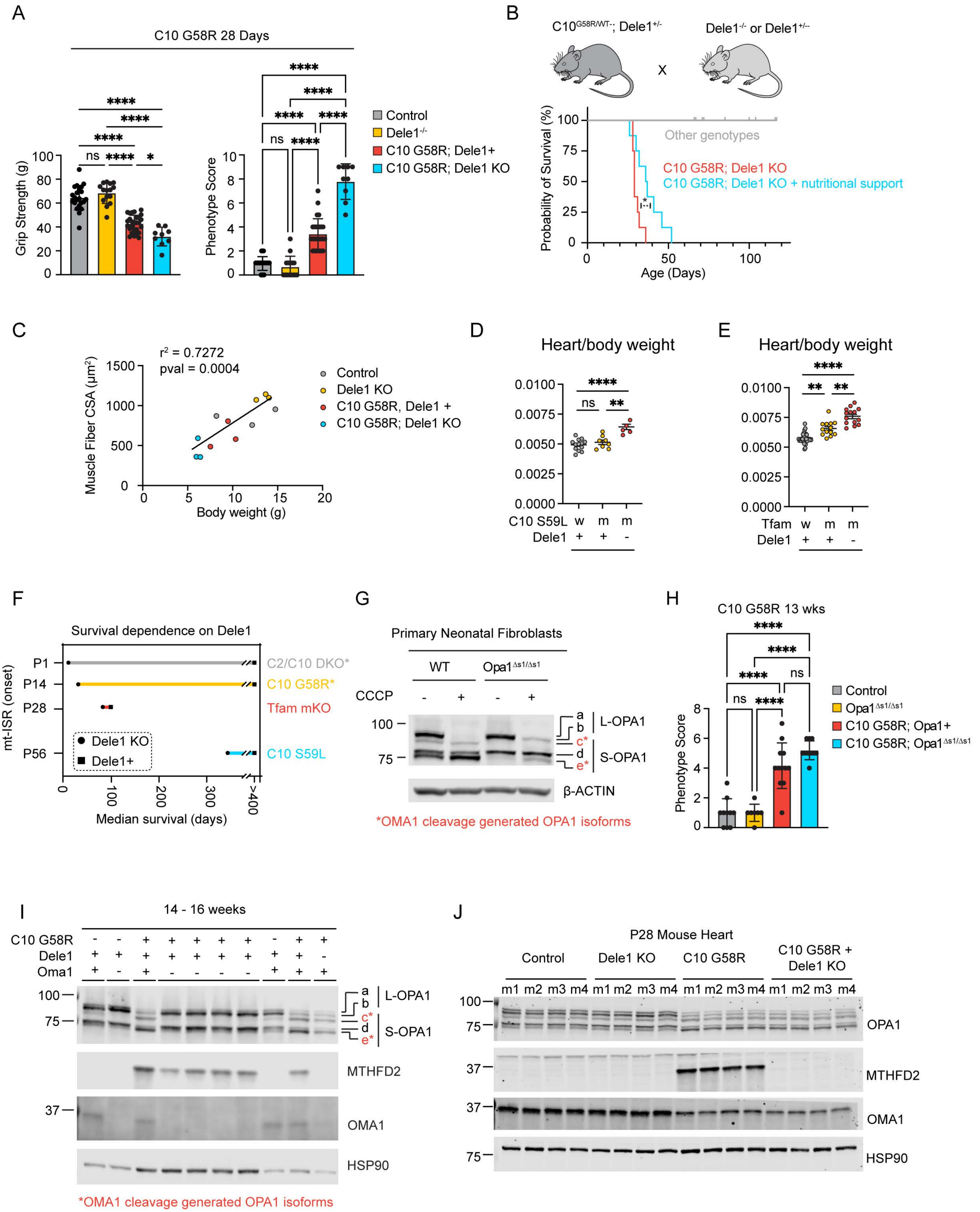

Appendix Figure S3. DELE1 mt-ISR promotes survival in diverse models of mitochondrial stress.

(A) Grip strength (left) and a composite phenotype score (right) of C10 G58R; *Dele1* KO mice and littermates at P28. Composite phenotype score is comprised of ledge test, hindlimb clasping, gait test, and kyphosis.

(B) Survival analysis of C10 G58R mice with and without nutritional support involving hand feeding twice per day.

(C) Simple linear regression between gastrocnemius muscle fiber cross sectional area (CSA) and body weight for of C10 G58R; *Dele1* KO mice and littermates.

(D) Heart to body weight ratio for C10 S59L mice; *Dele1* KO mice and littermates at P140.

(E) Heart to body weight ratio for *Tfam* mKO mice; Dele1 KO mice and littermates at P56.

(F) Correlation between age at stress onset and DELE1 survival benefit among the models. * indicates genotypes for which some lifespan estimates were determined from prior studies (Nguyen *et al*, 2022; Shammas *et al*, 2022).

(G) Primary fibroblasts from *Opa1*$^{\Delta s1/\Delta s1}$ mice treated with CCCP 20 μM or vehicle for 16 hrs. The *c* and *e* bands generated by OMA1 cleavage (from *a* and *b*, respectively) are reduced at baseline and following uncoupling with CCCP; the *b* band is also relatively retained with CCCP, together demonstrating that L-OPA1$^{\Delta s1/\Delta s1}$ (*a* and *b* bands) is resistant to OMA1 cleavage.

(H) Composite phenotype score of C10 G58R; *Opa1*$^{\Delta s1/\Delta s1}$ mice and littermates at 13 weeks.

(I) Immunoblot compares OPA1 cleavage by OMA1 and elevation of the mt-ISR marker protein MTHFD2 in C10 G58R animals with or without OMA1 or DELE1 that survived to 14 – 16 weeks. Lysates from *Oma1* KO animals are from samples that were previously generated and appeared in (Shammas *et al*, 2022).

(J) Immunoblot demonstrates OPA1 cleavage by OMA1 and elevation of the mt-ISR marker protein MTHFD2 in C10 G58R animals with or without DELE1 at P28. *Dele1* KO blocks MTHFD2 protein elevation but not OPA1 cleavage by OMA1. Consistently, OMA1 levels are decreased in C10 G58R mice in the presence or absence of DELE1.

Appendix Figure S4

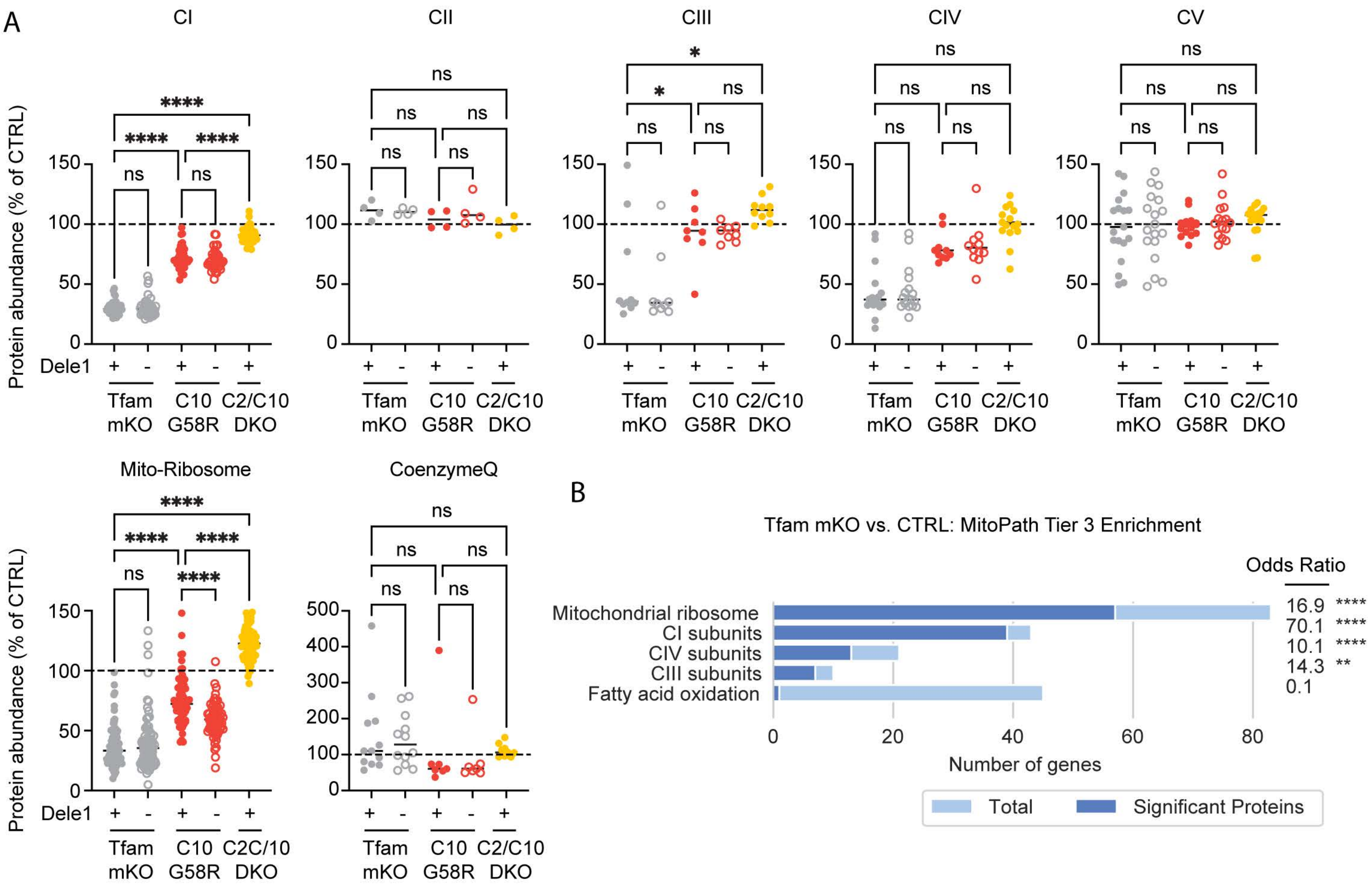

Appendix Figure S4. Comparison of OXPHOS subunit expression in heart mitochondria from diverse models of mitochondrial myopathy/cardiomyopathy.

(A) Scatterplots depicting relative abundance of OXPHOS complexes I – V subunits, mito-ribosome, and Coenzyme Q from the indicated genotypes. Data are from the same datasets represented in (Figure 2F – H), replotted to compare disease models. For statistics, a one-way ANOVA was performed followed by post-hoc testing, corrected for the multiple comparisons depicted within the graph with Dunnett's test. All values are relative to littermate controls except for C2/C10 DKO which are matched to unrelated age-matched controls. Data from these proteomics datasets also appear in Figure 4E - G.

(B) Enrichment analysis mitochondrial proteins that significantly changed in *Tfam* mKO vs. control mitochondria isolated from hearts, using Tier 3 MitoPaths from MitoCarta 3.0.

Appendix Figure S5

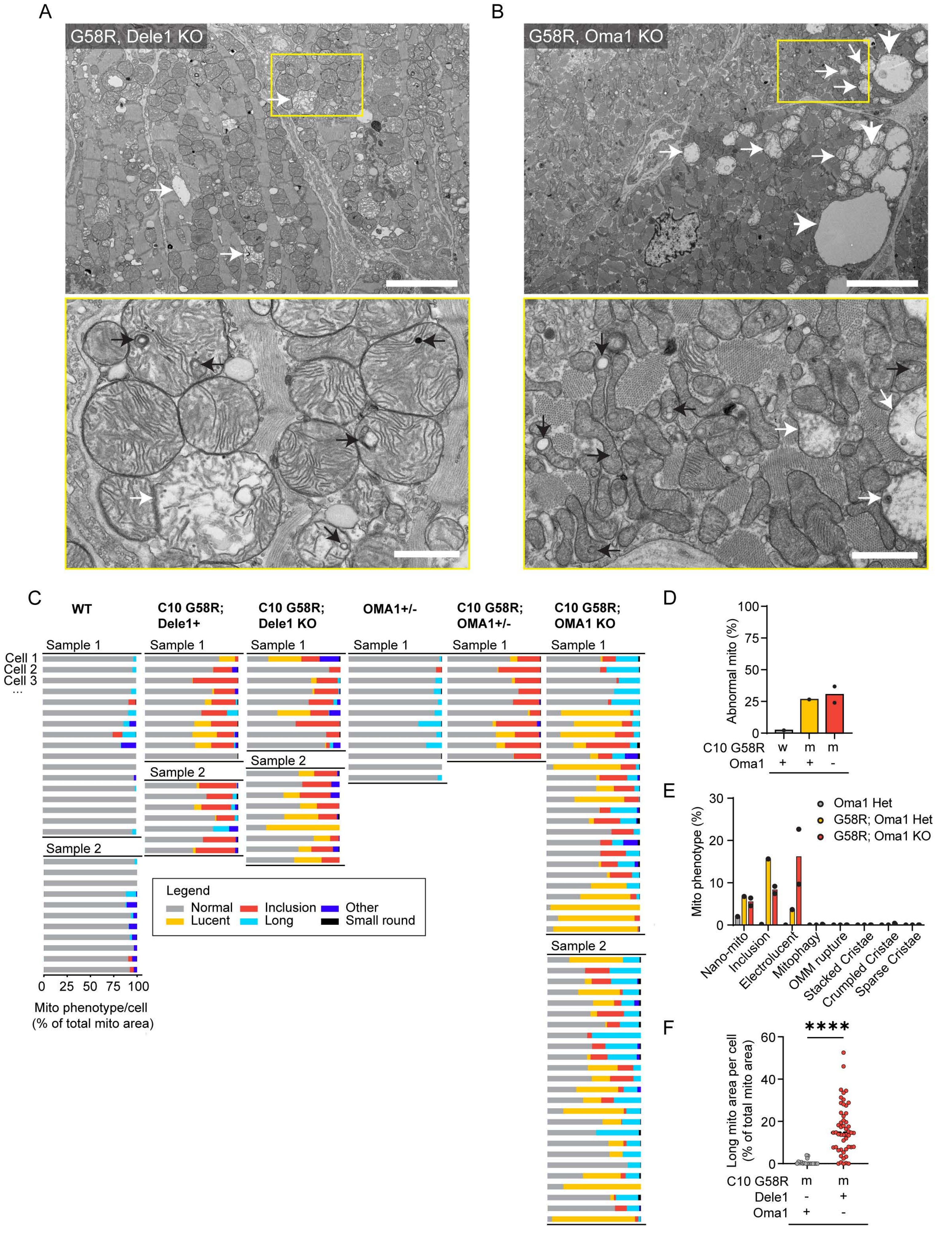

Appendix Figure S5. TEM of myocardium and ultrastructural features of mitochondria in C10 G58R on Oma1 KO vs. Dele1 KO backgrounds.

(A and B, top) Representative TEM images acquired at 2000x direct magnification show areas of myocardium of indicated genotype used for analysis of mitochondria. Large areas of some cells are occupied by extremely large electrolucent mitochondria. Scale bar = 5 µm. (A and B bottom) Image of the subarea boxed above, acquired at 5000x direct magnification and representative of the images used to quantify ultrastructural features of mitochondria detailed in (C – E). A population of long thin mitochondria observed on the *Oma1* KO background are not seen on the *Dele1* KO background. Scale bar = 2.5 µm. Black arrows = cristal membrane inclusions, white arrows = electrolucent mitochondria.

(C) Stacked bar graphs show proportion of mitochondria phenotype in each cell (as percent of total mitochondrial area in cell).

(D and E) Quantification of ultrastructural features for 14-week-old C10 G58R; Oma1 KO and indicated littermates.

(F) Graph comparing mitochondrial area with long phenotype per cell for C10 G58R; *Oma1* KO vs. C10 G58R; *Dele1* KO. Statistical analysis performed using a Mann-Whitney test as the data distribution was non-parametric.

Appendix Figure S6

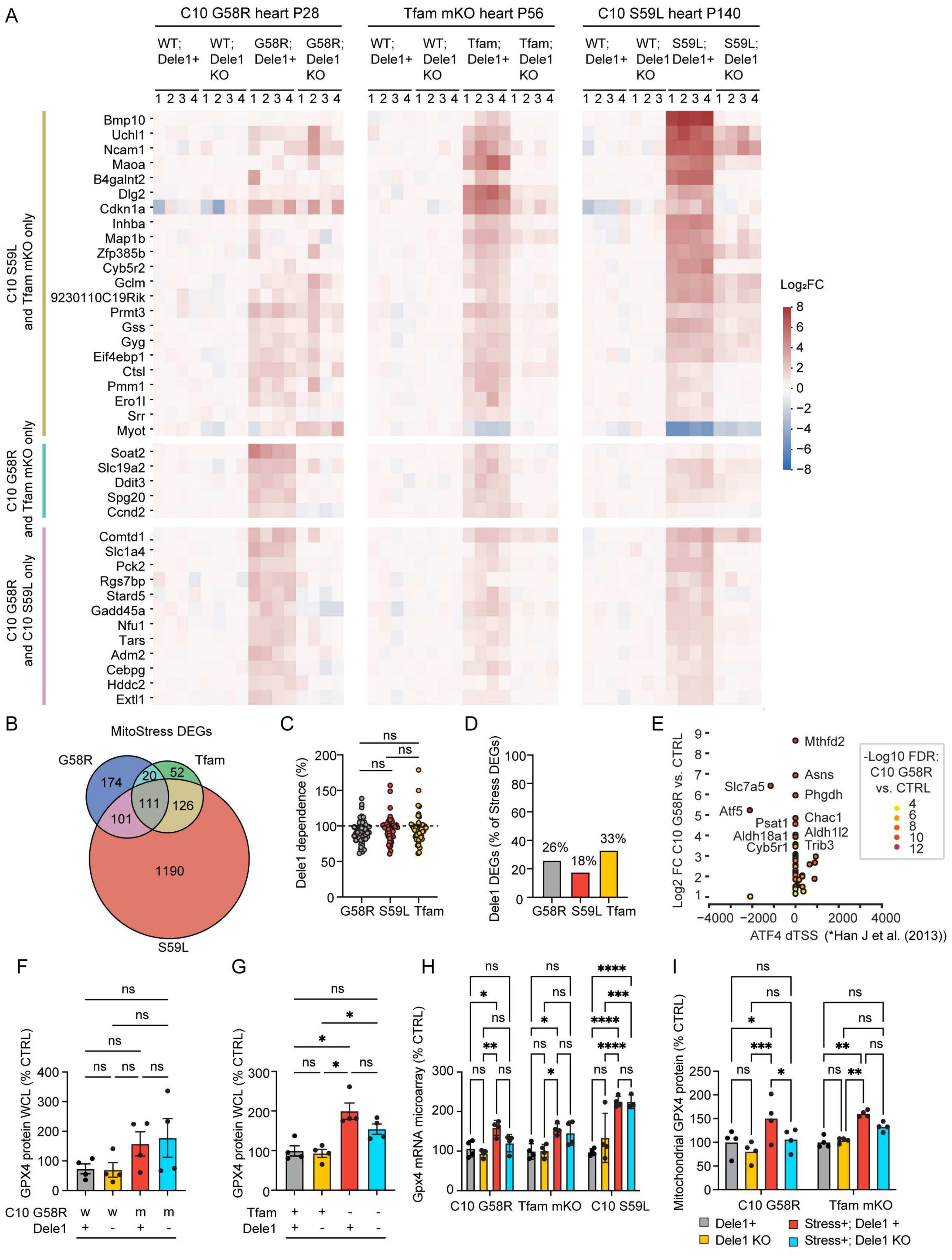

Appendix Figure S6. DELE1 mt-ISR transcriptional response in heart is similar in response to diverse mitochondrial stressors.

(A) Heat map of Log_2FC for DELE1-dependent DEGs detected in hearts of two out of three myopathy/cardiomyopathy models.

(B) Venn diagram showing intersection of stress-induced DEGs in heart among the 3 models of myopathy/cardiomyopathy.

(C) Scatterplot compares percent DELE1 dependence for the 51 genes in the heart DELE1 mt-ISR signature among the three models.

(D) Bar graph represents the percent of stress DEGs in each model that are significantly DELE1-dependent.

(E) Scatter plot shows the location of ATF4 peaks from previously published MEF ChIP-Seq data, published in (Han *et al*, 2013), for DELE1-dependent DEGs in the heart.

(F-G) GPX4 protein levels from heart whole cell lysate (WCL) of indicated genotypes. Statistical analysis was performed using Welch ANOVA followed by Dunnett's T3 multiple comparison test. Correction for multiple comparisons was limited to the groups shown in the Figure.

(H) *Gpx4* mRNA levels measured in microarray experiments, described in (Fig 4B). Statistical analysis was performed using Welch ANOVA followed by Dunnett's T3 multiple comparison test. Correction for multiple comparisons was limited to the groups shown in the Figure.

(I) GPX4 mitochondrial protein levels measured in proteomics experiments, described in (Fig 4E and F). Statistical analysis was performed using Welch ANOVA followed by Dunnett's T3 multiple comparison test. Correction for multiple comparisons was limited to the groups shown in the Figure.

Appendix Figure S7

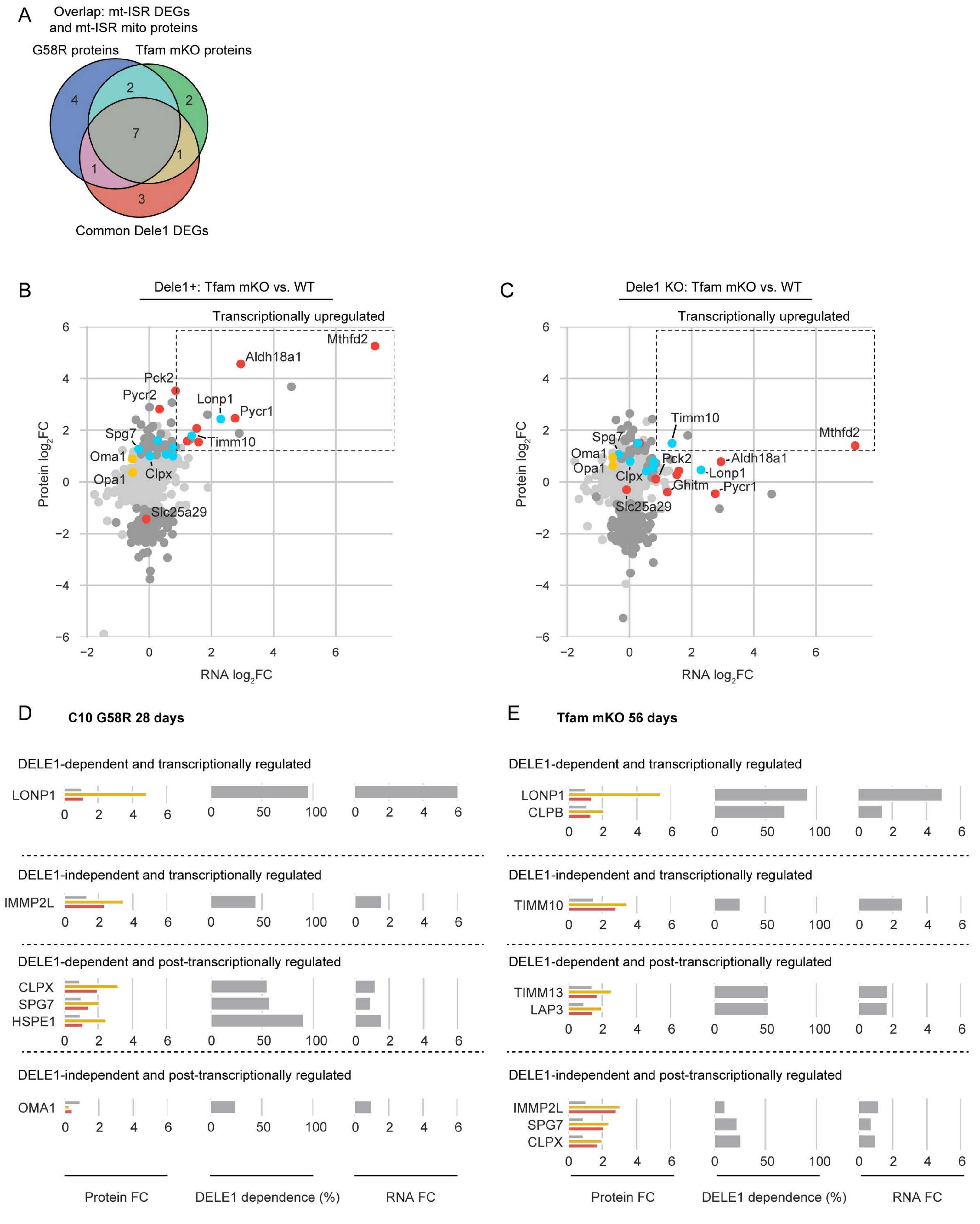

Appendix Figure S7. DELE1 mt-ISR mediates most transcriptionally driven increases in mitochondrial proteins involved in proteostasis.

(A) Venn diagram depicts intersection of the transcriptional DELE1 mt-ISR signature with significant DELE1-dependent changes in the mitochondrial proteome of C10 G58R and *Tfam* mKO animals.

(B - C) Scatterplot compares RNA log_2FC for *Tfam* mKO vs. control (in the presence of DELE1) and mitochondrial protein log_2FC for *Tfam* mKO vs. control animals in the presence of DELE1 (left) or the absence of DELE1 (right).

(D – E) Bar graphs showing protein and RNA fold changes for proteins annotated as proteases and chaperones in MitoCarta3.0, from experiments described in (Figure 4E, F, and G).

Appendix Figure S8

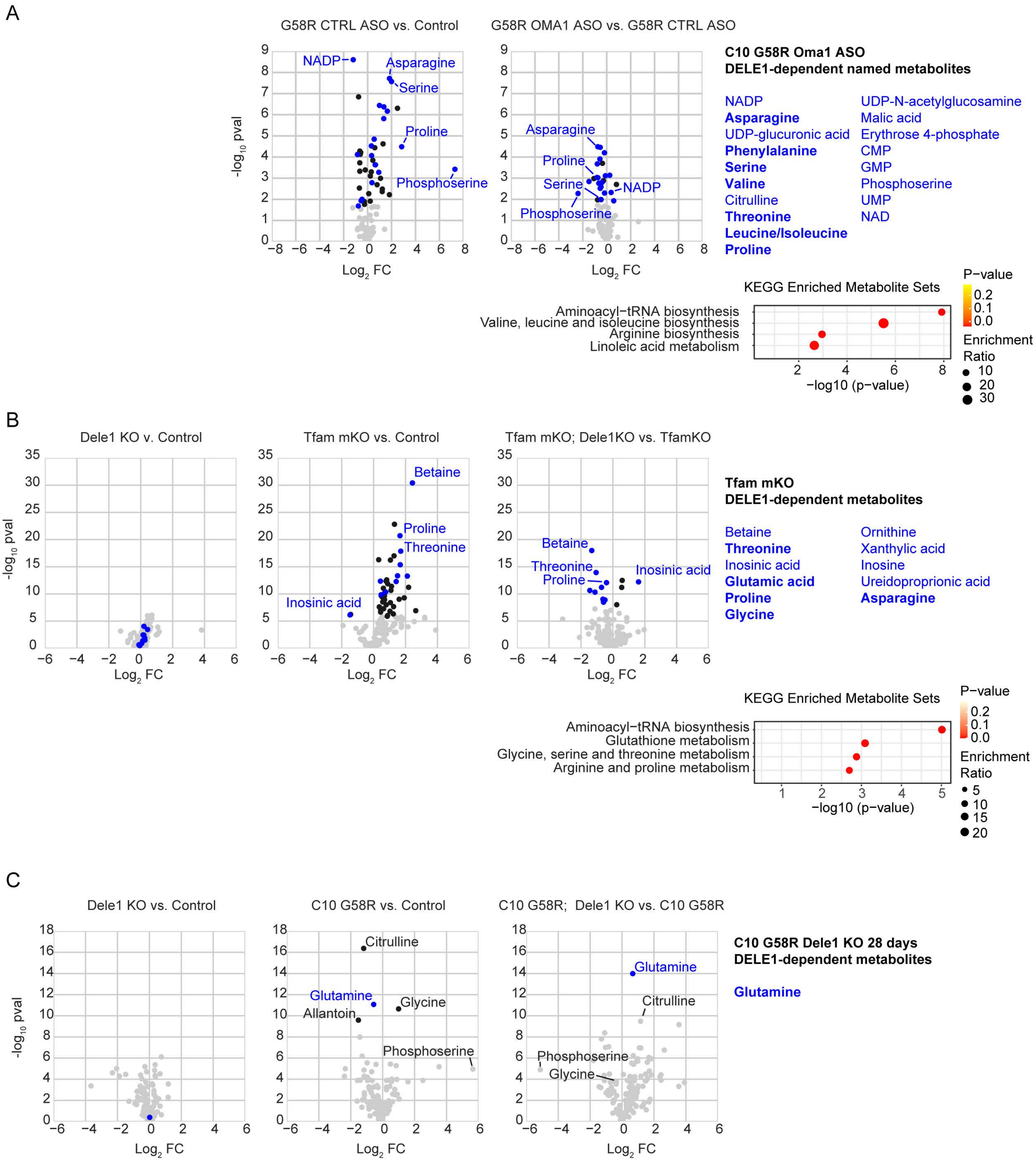

Appendix Figure S8. Metabolomics from hearts of C10 G58R and *Tfam* mKO models of mitochondrial myopathy/cardiomyopathy.

(A - C) Volcano plots of metabolites identified in targeted (B and C) or untargeted (A) metabolomics experiments and enrichment analysis among KEGG metabolite sites for the DELE1-dependent metabolites. Only named features in the untargeted metabolomics data are plotted in (A). DELE1-dependent metabolites are in blue, with amino acids bolded. Data from these metabolomics experiments also appear in Tables S4 and S5. Statistics were performed as described in the methods for metabolomics datasets.

Appendix Figure S9

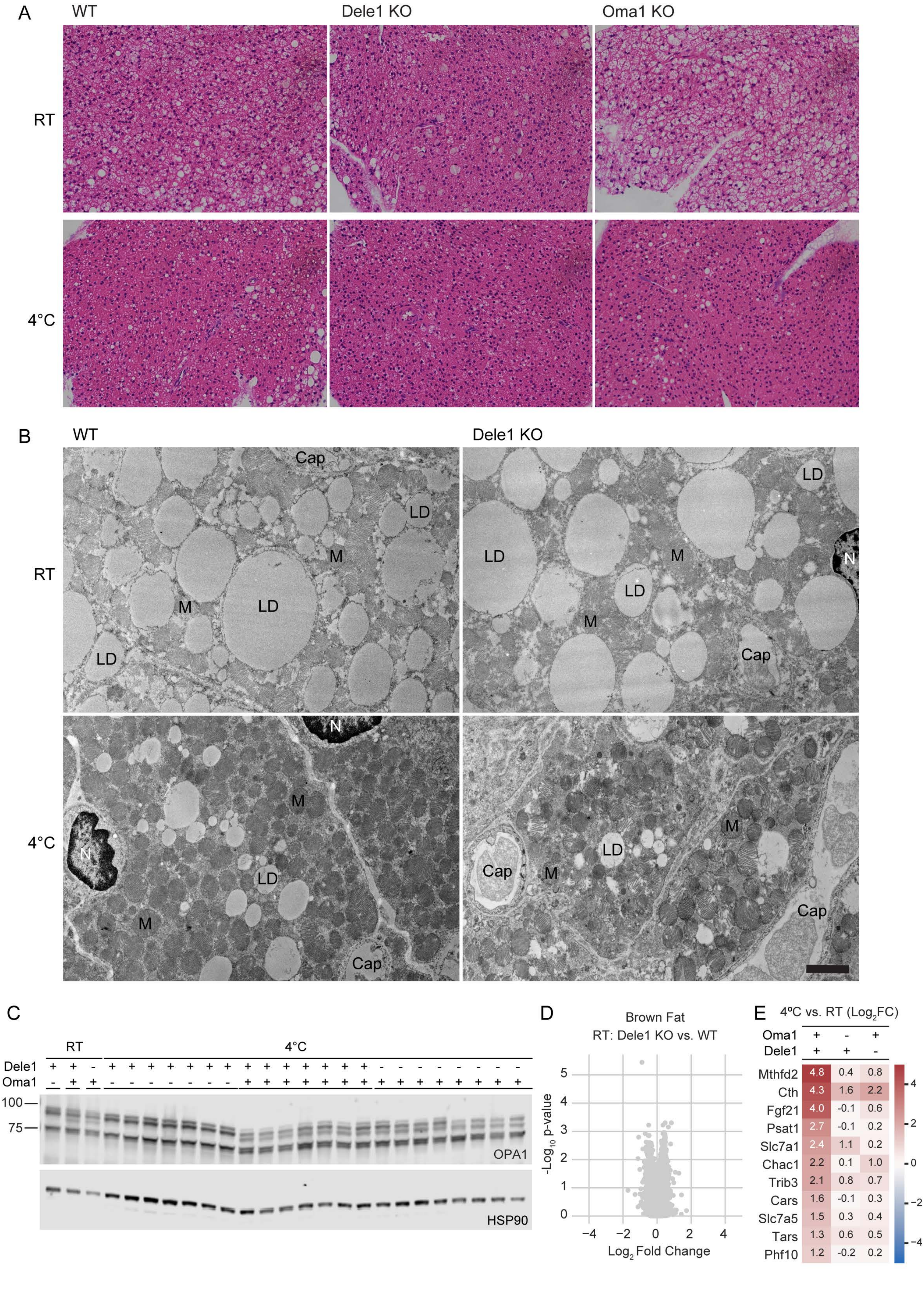

Appendix Figure S9. OMA1-DELE1 pathway mediates the integrated stress response in brown adipose tissue under cold stress.

(A) H&E-stained section of brown adipose tissue from *Dele1* KO, *Oma1* KO, and WT littermates (of *Dele1* KO) subjected to cold stress in (Figure 6A and B) shows reduction in brown adipose lipid droplets in all genotypes, which appear as unstained spheres, after cold stress.

(B) Immunoblot showing OPA1 cleavage by OMA1 in WT and *Dele1* KO but not *Oma1* KO mice that were analyzed in (Figure 6B and Appendix Figure S9E).

(C) TEM images of BAT from WT and Dele1 KO mice subjected to cold stress in (Fig. 6A and B) shows reduction in brown adipose lipid droplets in all genotypes after cold stress. M = mitochondria, LD = lipid droplet, Cap = capillary, N = nucleus.

(D) Volcano plot of microarray data from experiment in (Figure 6A and B) comparing gene expression changes between *Dele1* KO and WT littermates left at room temperature (RT). No significant gene changes were observed. Statistics performed as described in the methods for all microarray transcriptomics experiments. N=7 mice per group.

(E) Heat map compares fold change of BAT DELE1-dependent DEGs on WT, Oma1 KO, and Dele1 KO backgrounds for cold stress vs room temperature. Data is from microarray transcriptomics.

Appendix Figure S10

A

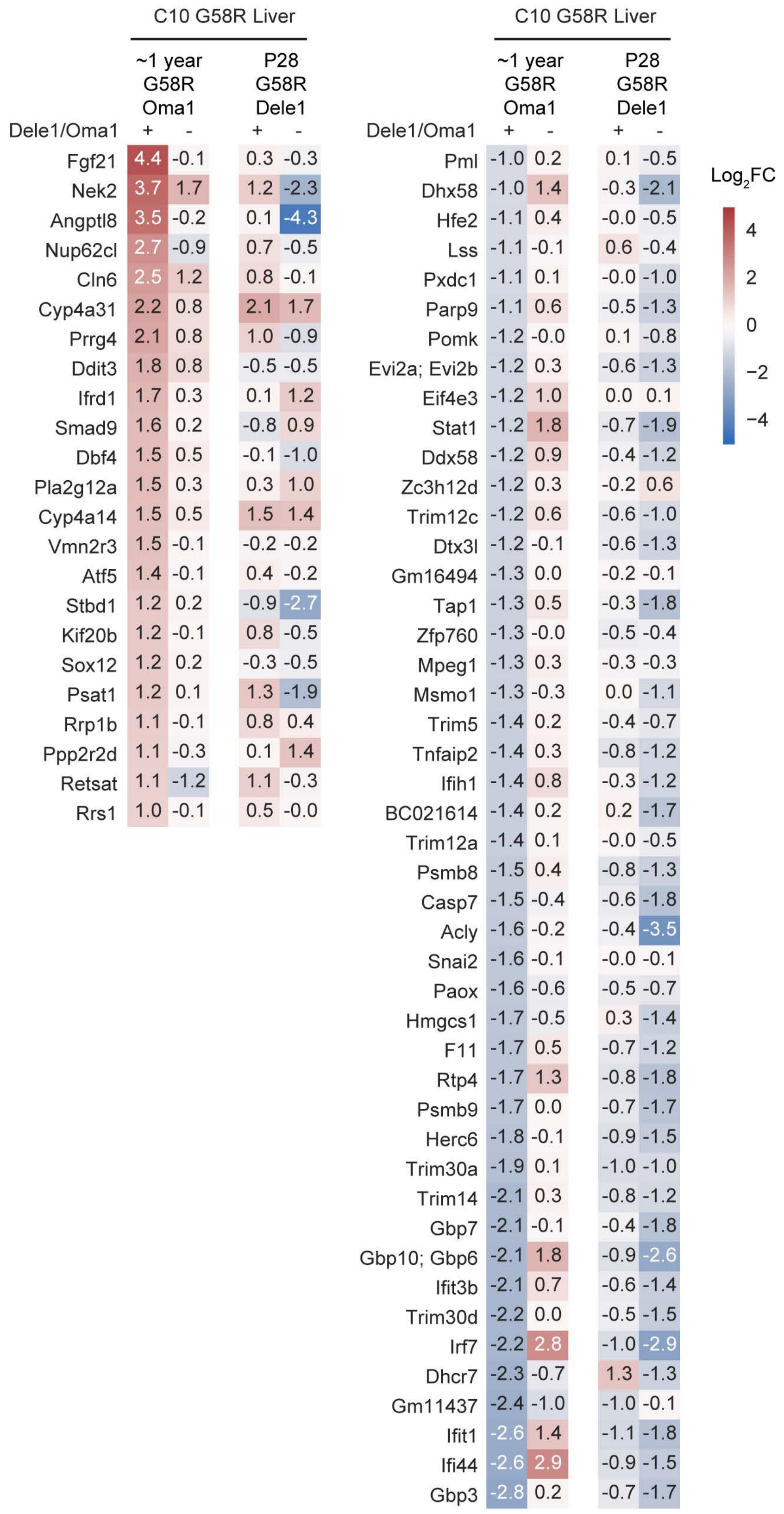

B

	Biological Process (Gene Ontology)			
GO-term	description	count in network	strength	false discovery rate
GO:0039528	Cytoplasmic pattern recognition receptor signaling pathwa in response to virus	3 of 10	2.0	0.0020
GO:0044790	Suppression of viral release by host	6 of 23	1.94	3.25e-07
GO:0031664	Regulation of lipopolysaccharide-mediated signaling pathway	5 of 28	1.77	3.57e-05
GO:0032727	Positive regulation of interferon-alpha production	4 of 26	1.71	0.00061

Appendix Figure S10. Transcriptomics from liver from C10 G58R mice with either *Dele1* KO (P28) or *Oma1* knockdown using an ASO (~1 year).

(A) Heat map depicts the OMA1-dependent DEGs detected in liver from ~1 year old C10 G58R mice compared to P28 C10 G58R mice. No DELE1-dependent DEGs were detected at P28. Data are from microarray-based transcriptomics and statistical analysis was performed as described in the methods for all microarray-based transcriptomics.

(B) Top gene ontology terms for the OMA1-dependent DEGs from livers of ~1 year old C10 G58R mice in (A).

Appendix Figure S11

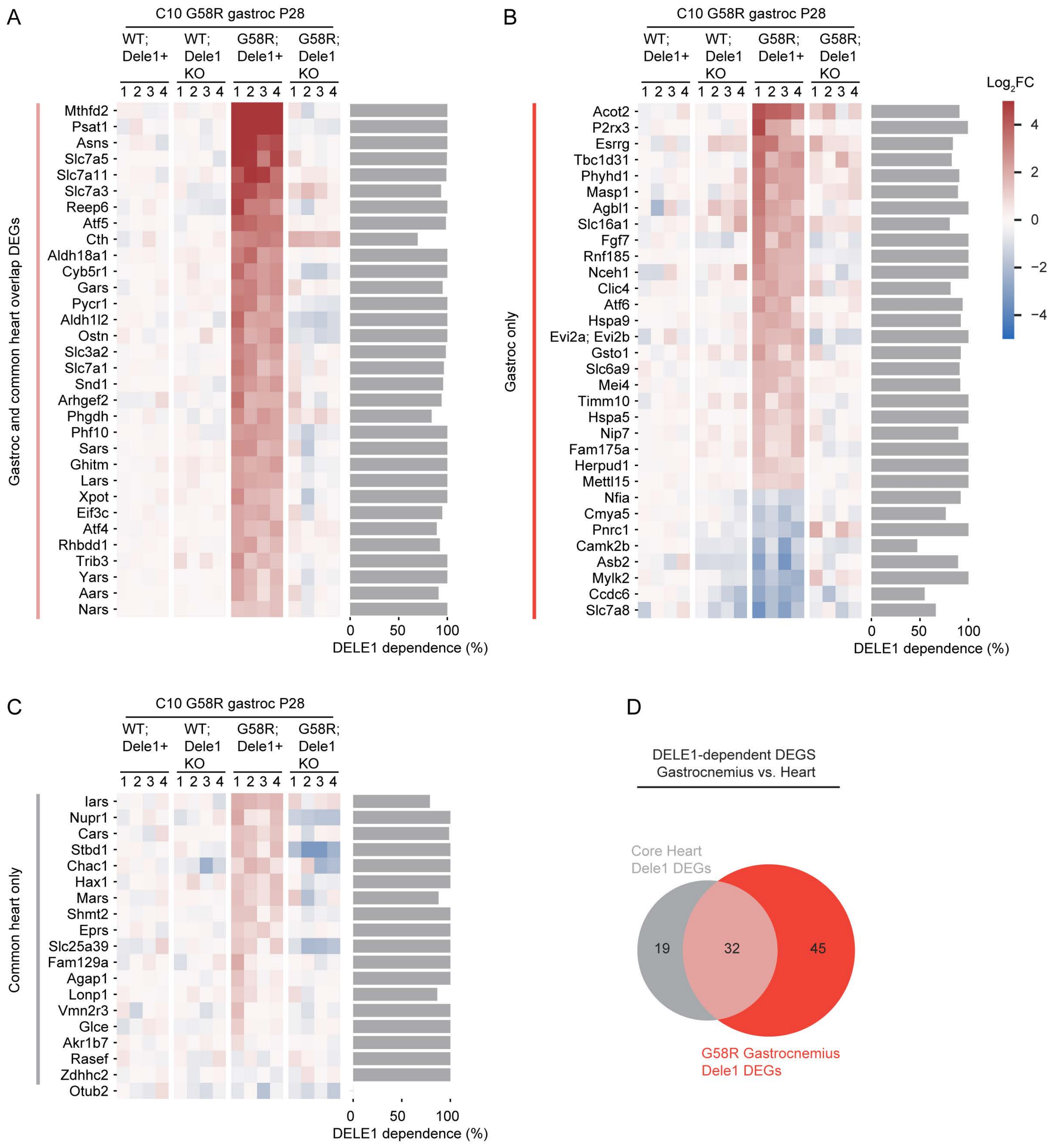

Appendix Figure S11. Transcriptomics from gastrocnemius skeletal muscle from P28 C10 G58R; *Dele1* KO mice and littermates.

(A) Heatmap of gene expression changes from gastrocnemius skeletal muscle for intersection of DELE1-dependent DEGs in gastrocnemius skeletal muscle and heart DELE1 mt-ISR signature. Bargraph on right represents percent DELE1 dependence.

(B) Heatmap of gene expression changes from gastrocnemius skeletal muscle for DELE1-dependent DEGs in gastrocnemius skeletal muscle that are not part of heart DELE1 mt-ISR signature. Bargraph on right represents percent DELE1 dependence.

(C) Heatmap of gene expression changes from gastrocnemius skeletal muscle for genes in the heart DELE1 mt-ISR signature that were not significantly DELE1-dependent in the gastrocnemius muscle. Bargraph on right represents percent DELE1 dependence.

(D) Venn diagram shows intersection of the heart DELE1 mt-ISR signature and C10 G58R DELE1-dependent DEGS from gastrocnemius skeletal muscle.

Appendix Figure S12

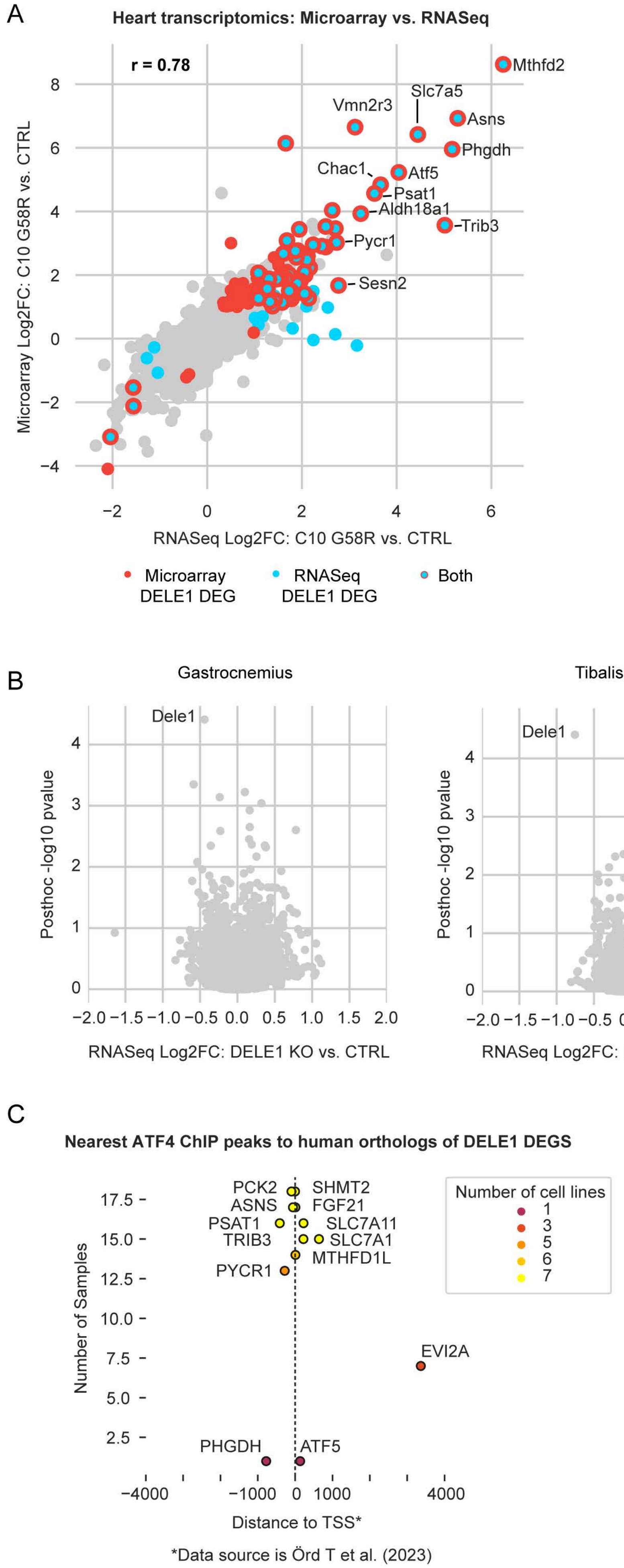

Appendix Figure S12. Cross-validation of microarray and RNA-Seq transcriptomics and analysis of Dele1 KO muscle tissues by RNA-Seq.

(A) Scatterplot shows the correlation between transcripts measured by microarray and RNA-Seq for the C10 G58R; Dele1+ vs. C10 WT; Dele1+ comparison in heart from P28 mice. Microarray data are also in Table S1 and RNA-Seq data in Table S9. Pearson correlation co-efficient was calculated using SciPy v1.14.0 library in Python.

(B) Volcano plots depict RNA-Seq data for C10 WT; Dele1 KO vs. C10 WT; Dele1 WT comparison from P28 mice. Black data points are significant (adjusted p-value < 0.05 and $|\log2\ FC| > 1$); gray data points are not significant. Statistical analysis was performed as described in methods for RNA-Seq transcriptomic data.

(C) Scatterplot depicts the nearest ATF4 peak from ChIP-Seq data (from (Örd *et al*, 2023)) for the 13 human orthologs of DELE1-dependent genes that are upregulated in skeletal muscle from MM patients in (Figure 6J).

Appendix Figure S13

DELE1 dependent DEGs common to gastroc, tibalis anterior, and heart

Female Gastrocnemius

C10 G58R	wt				wt			m			m		
Dele1	+				-			+			-		
	F1	F2	F3	F4	F1	F2	F3	F1	F2	F3	F1	F2	F3
Mthfd2	0.1	0.2	-0.2	-0.1	-0.2	0.0	-0.1	5.5	5.5	5.8	0.2	0.2	0.1
Asns	0.2	0.0	-0.1	-0.1	-0.1	0.0	-0.0	5.4	5.0	5.7	1.0	0.5	0.4
Trib3	0.0	0.0	0.0	0.0	0.1	0.0	0.0	5.6	5.5	6.3	0.6	0.3	0.2
Psat1	0.3	-0.0	0.2	-0.2	0.1	0.0	-0.1	5.4	5.0	5.5	0.3	0.2	0.1
Slc7a5	-0.0	0.1	-0.1	0.1	0.3	0.2	-0.2	4.4	3.6	4.9	0.9	0.1	0.5
Phgdh	0.6	-0.3	0.0	0.1	0.2	0.0	-0.5	3.6	2.8	3.7	0.2	0.3	0.6
Fgf21	0.0	0.0	0.0	0.0	0.0	0.0	0.0	5.0	4.1	6.1	0.0	0.0	0.0
Aldh18a1	-0.1	-0.0	-0.1	0.3	0.1	0.0	-0.4	3.3	2.9	3.7	-0.9	-0.8	-0.7
Atf5	0.3	-0.2	0.1	0.2	-0.1	-0.2	-0.2	3.0	2.7	3.2	0.3	0.2	0.6
N4bp2l1	-0.0	0.0	-0.0	-0.0	-0.0	0.0	-0.0	4.0	3.4	4.3	0.5	0.1	0.4
Pycr1	0.2	-0.1	-0.1	0.0	0.1	0.3	-0.2	3.0	2.2	3.2	-1.1	-0.3	-0.6
Pck2	0.1	-0.1	0.3	-0.1	0.1	-0.1	0.1	3.7	3.0	4.1	-0.3	-0.1	0.0
Sesn2	0.0	0.0	-0.1	-0.1	0.0	0.1	-0.1	2.5	3.1	4.1	-0.2	-0.2	-0.2
Nupr1	0.9	-0.4	0.3	-0.6	-0.2	0.3	0.4	2.2	2.5	3.1	-1.0	-1.0	-0.9
Snd1	-0.1	-0.2	0.1	0.4	-0.0	-0.1	-0.4	3.1	1.7	3.7	0.4	-0.1	0.3
Slc6a9	0.1	0.2	-0.1	0.1	0.4	0.1	-0.2	2.9	2.3	3.4	-0.3	-0.2	-0.5
Cyb5r1	-0.1	-0.0	0.0	-0.0	0.1	-0.1	-0.2	2.4	1.8	2.5	-0.2	0.4	-0.1
Tenm4	0.1	-0.0	-0.0	-0.0	-0.0	0.0	-0.0	3.3	1.5	3.1	-0.0	-0.0	-0.0
Aldh1l2	-0.1	-0.3	-0.5	0.3	0.3	0.0	-0.3	2.1	1.3	2.2	-0.4	-1.0	-0.4
Slc3a2	0.1	0.0	0.0	0.1	0.2	0.1	-0.1	2.4	2.0	2.6	0.5	0.4	0.9
Gadd45a	0.1	0.1	0.1	0.1	-0.0	0.4	-0.1	2.9	2.5	3.4	0.4	0.2	0.3
Gars	-0.1	0.2	-0.2	0.1	0.1	-0.1	-0.2	2.1	1.7	2.4	0.2	0.3	0.4
Soat2	0.0	0.0	0.0	0.0	0.0	0.0	0.0	3.2	1.9	3.2	0.0	0.0	0.0
Slc7a11	0.0	0.0	0.0	0.0	0.0	0.0	0.0	2.2	2.3	3.3	0.0	0.0	0.0
Arhgef2	-0.2	-0.4	0.2	0.0	-0.0	-0.1	0.3	1.9	1.8	2.2	-0.6	-0.5	-0.5
Brca2	0.0	0.0	0.0	0.0	0.0	0.0	0.0	1.8	0.7	1.7	0.0	0.0	0.1
Phf10	0.3	0.3	0.2	-0.3	-0.1	0.2	0.5	1.7	2.0	1.8	0.0	-0.3	-0.2
Ddit3	0.1	-0.5	0.5	-0.1	-0.6	-0.3	-0.0	1.7	2.3	2.3	-1.0	0.4	-1.1
Slc7a3	0.0	0.0	0.0	0.0	0.0	0.0	0.0	2.3	1.4	2.3	0.0	0.0	0.0
Ostn	0.5	0.4	-0.4	-0.4	0.6	0.4	-0.2	2.0	0.8	1.5	-0.4	-0.3	-0.6
Extl1	-0.2	-0.1	-0.2	0.0	-0.0	-0.2	-0.3	1.7	1.1	1.8	-0.1	-0.2	-0.1
Cebpg	-0.2	-0.3	0.1	0.1	-0.3	-0.5	0.3	1.5	1.2	1.7	-0.4	-0.3	-0.2
Xpot	-0.1	-0.0	-0.1	0.0	-0.0	-0.2	-0.1	1.6	1.0	1.4	-0.0	-0.2	-0.1
Yars	-0.3	-0.1	0.1	0.1	-0.1	-0.1	-0.3	1.4	1.1	1.7	-0.5	-0.3	-0.0
Adm2	0.0	0.0	0.0	0.0	0.0	0.0	0.0	1.3	0.9	1.6	0.0	0.0	0.0
Ghitm	-0.0	-0.0	-0.1	0.0	0.0	-0.1	-0.1	1.8	1.3	1.8	-0.0	-0.2	-0.1
Stbd1	-0.4	-0.4	0.3	0.2	-0.4	-0.2	0.0	1.4	1.0	1.4	-1.2	-0.2	-1.4
Stard5	-0.2	-0.2	0.1	-0.1	0.2	-0.3	-0.1	1.5	1.3	1.7	-0.2	-0.1	0.3
Slc25a39	-0.0	-0.1	0.0	-0.0	0.1	0.1	0.0	1.5	1.1	1.3	-0.5	-0.5	-0.4

Male Gastrocnemius

C10 G58R	wt		wt			m			m		
Dele1	+		-			+			-		
	M1	M2	M1	M2	M3	M1	M2	M3	M1	M2	M3
Mthfd2	0.1	-0.1	0.2	0.1	-0.1	5.8	5.9	5.6	0.3	-0.2	-0.4
Asns	-0.1	-0.1	-0.1	-0.1	-0.1	5.0	5.6	5.4	1.1	0.7	0.6
Trib3	0.0	0.0	0.0	0.0	0.0	4.6	5.0	5.1	0.5	0.1	0.8
Psat1	-0.0	-0.5	0.3	0.0	0.3	4.9	5.2	5.0	-0.4	-0.2	-0.4
Slc7a5	-0.1	0.0	0.1	-0.3	-0.2	4.0	4.0	3.9	0.0	-0.2	1.6
Phgdh	-0.4	-0.3	0.0	-0.3	-0.3	3.4	3.1	3.1	-0.0	-0.6	-0.5
Fgf21	0.0	0.0	0.0	0.0	0.0	3.6	4.9	5.4	0.0	0.0	0.0
Aldh18a1	-0.1	0.1	0.1	-0.2	0.1	3.7	3.5	3.4	-0.8	-0.6	-1.2
Atf5	-0.1	-0.3	-0.2	-0.2	-0.2	2.8	2.7	3.2	0.4	0.8	0.2
N4bp2l1	-0.0	0.0	-0.0	0.0	0.0	3.4	3.2	3.3	1.0	0.0	2.2
Pycr1	-0.0	-0.1	-0.1	-0.1	-0.1	2.7	2.7	2.3	-0.7	-0.1	-1.1
Pck2	-0.0	-0.3	0.5	0.6	0.4	3.6	3.5	3.1	0.2	0.0	2.2
Sesn2	-0.0	0.1	-0.1	-0.0	-0.0	2.7	2.5	3.1	0.1	-0.3	0.1
Nupr1	-0.3	-0.5	0.2	0.5	0.1	1.3	1.8	1.8	-0.8	-1.1	-1.1
Snd1	0.0	-0.3	-0.3	0.0	-0.0	2.9	2.0	1.9	0.7	0.1	0.7
Slc6a9	-0.1	-0.2	-0.1	-0.2	-0.4	2.8	2.5	2.3	-0.3	-0.2	-0.2
Cyb5r1	0.1	0.1	-0.0	-0.1	0.0	2.1	2.0	1.9	-0.2	0.1	-1.2
Tenm4	-0.0	-0.0	0.0	-0.0	-0.0	1.8	1.4	2.0	-0.0	-0.0	-0.0
Aldh1l2	0.1	0.3	-0.1	0.3	-0.4	2.1	1.8	1.7	-0.5	-0.8	-1.4
Slc3a2	-0.0	-0.3	-0.0	0.0	0.1	2.0	2.2	2.5	0.7	0.5	1.0
Gadd45a	-0.2	-0.3	0.3	0.4	0.6	2.5	2.9	3.3	1.5	0.1	0.4
Gars	0.0	-0.0	-0.0	-0.1	-0.1	2.2	2.2	1.9	0.4	0.1	0.8
Soat2	0.0	0.0	0.0	0.0	0.0	2.3	1.5	1.4	0.0	0.0	0.0
Slc7a11	0.0	0.0	0.0	0.0	0.0	2.8	3.2	2.6	0.0	0.0	0.0
Arhgef2	0.2	-0.0	0.1	-0.2	0.1	1.8	1.9	1.9	-0.8	-0.7	0.1
Brca2	0.0	0.0	0.0	0.0	0.0	1.1	0.9	0.7	0.0	0.0	0.0
Phf10	-0.2	-0.3	-0.0	-0.0	0.2	1.6	2.4	1.8	-0.4	0.0	0.3
Ddit3	-0.1	-0.1	-0.9	-0.4	-0.7	0.5	0.8	1.0	-1.2	0.3	-0.7
Slc7a3	0.0	0.0	0.0	0.0	0.0	1.9	1.7	1.4	0.0	0.0	0.0
Ostn	-0.2	-0.2	0.3	0.0	-0.7	1.2	1.7	1.2	-0.7	-0.4	-1.2
Extl1	0.1	0.3	-0.1	0.0	-0.0	1.8	1.6	1.6	-0.1	-0.1	-1.2
Cebpg	0.1	0.1	-0.1	-0.0	0.2	1.3	1.6	1.7	0.1	0.1	0.3
Xpot	0.1	0.1	-0.1	-0.1	-0.2	1.2	1.4	1.6	-0.4	-0.1	-0.5
Yars	0.2	0.0	0.1	-0.2	0.1	1.4	1.2	1.4	0.1	-0.3	-0.5
Adm2	0.0	0.0	0.0	0.0	0.0	1.0	1.0	1.0	0.0	0.0	0.0
Ghitm	0.1	-0.0	-0.1	-0.0	-0.1	1.5	1.3	1.4	0.1	-0.3	-0.5
Stbd1	0.1	0.1	-0.2	-0.4	0.2	0.9	1.2	1.0	-1.2	0.4	-1.9
Stard5	0.2	0.2	-0.2	0.4	-0.4	1.0	0.7	0.9	0.0	-0.2	-0.2
Slc25a39	-0.1	0.1	-0.1	-0.1	-0.0	1.0	1.0	1.2	-0.7	-0.3	-1.0

Log2 FC: 4, 2, 0, −2, −4

Appendix Figure S13. DELE1-dependent DEGs common skeletal and heart muscle show same pattern of activation in male and female gastrocnemius muscle from P28 CHCHD10 G58R mice. Heatmap shows log2 FC for DELE1-dependent genes separately for male and female animals. Values were unadjusted for sex by ANCOVA. RNA-Seq data are also in Table S7.

Appendix Figure S14

DELE1 dependent DEGs common to gastroc, tibalis anterior, and heart

Female Hearts

C10 G58R	wt				wt			m			m		
Dele1	+				-			+			-		
	F1	F2	F3	F4	F1	F2	F3	F1	F2	F3	F1	F2	F3
Mthfd2	-0.0	0.1	0.1	-0.1	0.1	-0.1	-0.1	6.2	6.2	6.4	3.0	2.2	2.2
Asns	-0.1	0.6	-0.2	-0.2	0.0	0.1	-0.1	5.2	5.0	5.4	2.4	1.5	1.6
Trib3	0.1	-0.2	-0.0	0.0	0.2	0.4	-0.2	5.4	5.0	5.3	1.7	1.8	1.2
Psat1	0.0	0.0	-0.0	-0.2	0.2	0.3	0.1	3.9	3.3	3.6	3.0	1.3	1.6
Slc7a5	-0.2	0.2	0.0	0.1	0.1	0.1	-0.2	4.8	4.4	5.0	2.0	1.7	1.7
Phgdh	0.2	0.1	-0.1	0.0	0.1	0.5	-0.2	5.3	5.2	5.4	0.6	0.2	0.5
Fgf21	0.0	0.0	0.0	0.0	0.0	0.0	0.0	3.2	1.9	2.3	0.0	0.0	0.0
Aldh18a1	0.1	-0.0	-0.2	-0.1	0.4	0.3	-0.4	3.5	3.2	3.5	0.6	1.0	0.6
Atf5	0.2	-0.1	-0.1	-0.2	0.2	0.1	-0.2	4.2	3.9	4.2	0.3	0.6	0.4
N4bp2l1	0.5	0.2	-0.1	-0.3	-0.2	-0.1	-0.3	3.0	2.3	2.2	-0.1	-0.2	-0.3
Pycr1	0.1	-0.0	-0.0	-0.0	0.0	0.4	-0.0	2.9	2.8	3.1	1.1	0.7	0.6
Pck2	0.1	-0.0	0.2	-0.3	0.3	-0.0	-0.0	1.7	1.5	2.0	-0.1	-0.3	-0.0
Sesn2	-0.0	-0.1	-0.1	0.1	0.2	0.2	0.0	3.1	2.7	3.0	0.1	0.7	0.6
Nupr1	-0.1	0.3	-0.2	0.2	0.0	0.0	-0.1	2.8	2.2	2.5	0.1	-0.3	0.4
Snd1	0.0	0.1	-0.1	-0.1	0.1	0.0	-0.1	3.1	2.4	2.6	0.7	0.7	0.5
Slc6a9	0.1	-0.1	-0.2	0.1	-0.1	0.2	-0.1	2.3	2.0	2.5	0.4	0.5	0.7
Cyb5r1	0.0	0.1	-0.1	-0.1	0.1	0.1	0.0	3.0	2.4	2.9	1.5	0.6	0.8
Tenm4	0.5	-0.2	-0.2	0.1	0.3	0.5	-0.2	3.7	1.8	3.2	-0.2	-0.2	-0.2
Aldh1l2	-0.3	-0.2	-0.0	0.3	0.0	0.1	-0.2	2.9	2.8	3.2	-0.8	-0.5	-0.8
Slc3a2	0.0	0.1	-0.0	-0.0	0.1	0.1	-0.0	2.2	2.0	2.2	1.0	0.7	1.2
Gadd45a	0.3	0.1	-0.0	-0.2	0.0	0.3	-0.2	1.2	1.6	1.4	-1.5	-1.1	-1.9
Gars	-0.0	0.1	0.0	0.0	0.1	-0.1	0.2	2.3	2.0	2.5	0.9	0.8	0.8
Soat2	0.0	0.0	0.0	0.0	0.0	0.0	0.0	2.8	1.6	2.1	0.0	0.0	0.0
Slc7a11	0.0	0.0	0.0	0.0	0.0	0.0	0.0	1.2	0.9	1.4	0.0	0.0	0.0
Arhgef2	-0.4	-0.2	-0.0	0.1	0.1	0.1	-0.3	2.0	1.7	2.1	0.1	0.2	0.1
Brca2	-0.1	-0.1	-0.1	-0.0	-0.1	0.3	-0.1	3.8	3.0	2.9	-0.1	-0.1	-0.1
Phf10	0.2	0.2	-0.0	-0.3	0.0	0.0	0.6	1.5	1.4	1.6	0.5	0.0	0.2
Ddit3	-0.1	-0.3	0.2	0.2	-0.2	-0.2	-0.0	2.2	2.1	2.4	0.5	0.5	0.1
Slc7a3	0.0	0.0	0.0	0.0	0.0	0.0	0.0	2.3	1.5	2.1	0.0	0.0	0.0
Ostn	0.0	0.0	0.0	0.0	0.0	0.0	0.0	3.2	1.6	2.7	0.0	0.0	0.0
Extl1	-0.2	-0.2	-0.1	-0.1	0.1	-0.1	-0.2	2.1	1.7	2.0	-1.2	-0.6	-1.2
Cebpg	0.3	0.0	-0.1	-0.2	0.2	-0.2	0.1	1.9	1.9	2.1	0.3	0.0	-0.2
Xpot	-0.1	-0.1	0.0	0.0	-0.2	0.1	-0.0	2.2	1.8	2.2	0.6	0.4	0.4
Yars	0.0	-0.1	-0.0	-0.0	-0.0	0.1	-0.0	2.0	1.9	2.1	0.7	0.6	0.5
Adm2	0.0	0.0	0.0	0.0	0.0	0.0	0.0	2.5	1.8	2.3	0.0	0.0	0.0
Ghitm	0.0	0.0	-0.0	0.0	-0.1	0.0	0.1	1.4	1.2	1.4	-0.0	-0.2	-0.1
Stbd1	0.1	0.3	-0.4	0.2	0.0	0.1	-0.7	2.2	1.5	2.4	0.2	-0.6	-0.0
Stard5	0.4	-0.2	-0.2	0.1	-0.2	-0.3	0.0	1.8	1.6	2.2	-0.4	-0.2	0.4
Slc25a39	0.2	-0.0	-0.2	-0.1	0.1	0.1	-0.0	1.7	1.2	1.4	0.2	0.2	0.3

Male Hearts

C10 G58R	wt		wt			m			m		
Dele1	+		-			+			-		
	M1	M2	M1	M2	M3	M1	M2	M3	M1	M2	M3
Mthfd2	-0.0	-0.0	0.1	0.3	0.3	6.1	6.5	6.0	3.1	1.5	-0.0
Asns	-0.2	-0.1	0.0	-0.2	-0.2	5.0	5.9	5.1	2.3	0.9	0.0
Trib3	-0.3	0.3	-0.1	0.1	-0.2	5.0	4.7	4.5	1.4	0.9	2.2
Psat1	0.2	-0.1	0.5	0.4	0.1	3.3	4.1	2.9	2.4	0.7	-0.0
Slc7a5	-0.2	-0.1	0.2	0.4	-0.0	4.2	3.9	4.2	1.8	1.3	1.5
Phgdh	-0.2	-0.0	-0.1	-0.2	-0.1	5.2	5.1	4.5	0.7	0.0	-0.3
Fgf21	0.0	0.0	0.0	0.0	0.0	1.4	1.5	2.4	0.0	0.0	0.0
Aldh18a1	0.2	0.0	-0.2	-0.0	-0.3	3.0	2.8	3.2	0.6	0.6	-0.1
Atf5	-0.1	0.2	0.0	-0.0	0.0	3.9	3.9	4.1	0.4	0.5	1.8
N4bp2l1	-0.2	-0.2	0.2	-0.3	-0.3	3.1	2.6	2.0	0.2	-0.3	2.3
Pycr1	-0.0	-0.0	-0.0	-0.0	-0.0	2.5	2.6	2.3	0.6	0.6	0.2
Pck2	0.1	-0.2	0.6	0.3	0.2	1.8	1.9	1.3	0.2	-0.7	0.2
Sesn2	-0.0	0.1	0.0	0.2	-0.0	3.0	2.4	2.4	0.5	0.4	0.4
Nupr1	-0.2	-0.2	-0.0	0.1	-0.0	2.2	2.8	2.5	0.2	-0.4	-0.4
Snd1	-0.1	0.1	0.0	0.0	0.2	3.1	1.7	2.1	0.9	0.5	0.5
Slc6a9	0.0	0.1	0.1	0.2	0.1	2.4	2.0	2.2	0.4	0.1	1.6
Cyb5r1	-0.0	0.1	0.1	0.1	-0.2	2.5	2.7	2.5	0.9	0.5	-0.4
Tenm4	-0.2	-0.2	-0.2	-0.2	-0.2	2.1	2.5	2.5	-0.2	-0.2	-0.2
Aldh1l2	0.2	-0.0	-0.6	0.1	0.2	2.6	1.6	2.2	-1.0	-1.1	-1.5
Slc3a2	-0.1	-0.0	0.4	0.2	0.1	1.9	2.0	2.1	0.9	0.8	1.0
Gadd45a	-0.1	-0.3	0.2	-0.4	-0.6	1.4	2.0	1.7	-0.9	-1.2	-0.1
Gars	-0.1	-0.1	-0.0	-0.1	-0.1	2.1	2.3	2.2	1.1	0.6	0.7
Soat2	0.0	0.0	0.0	0.0	0.0	1.7	1.6	1.7	0.0	0.0	0.0
Slc7a11	0.0	0.0	0.0	0.0	0.0	1.1	1.2	0.7	0.0	0.0	0.0
Arhgef2	0.3	0.1	0.4	0.3	0.4	1.6	1.6	1.5	0.1	0.5	-0.3
Brca2	-0.1	0.3	-0.1	-0.1	-0.1	3.2	3.2	2.7	-0.1	-0.1	-0.1
Phf10	-0.0	-0.1	-0.1	-0.1	-0.1	1.4	2.6	1.6	0.3	0.2	0.1
Ddit3	-0.2	0.1	-0.3	-0.0	0.1	1.8	2.2	1.5	0.0	0.3	0.5
Slc7a3	0.0	0.0	0.0	0.0	0.0	1.5	1.0	1.5	0.0	0.0	0.0
Ostn	0.0	0.0	0.0	0.0	0.0	1.0	1.4	1.1	0.0	0.0	0.0
Extl1	0.1	0.4	-0.1	0.2	-0.2	1.6	1.3	1.8	-0.9	-0.4	-1.0
Cebpg	0.2	-0.2	0.5	0.2	0.4	1.6	2.0	1.8	0.1	0.1	0.6
Xpot	-0.0	0.1	-0.3	-0.0	-0.1	1.7	1.7	1.6	0.5	0.6	0.2
Yars	0.0	0.1	0.1	0.1	-0.1	1.7	1.9	1.8	0.8	0.7	0.7
Adm2	0.0	0.0	0.0	0.0	0.0	1.5	1.7	1.7	0.0	0.0	0.0
Ghitm	0.0	-0.0	-0.0	0.1	-0.0	1.3	1.3	1.1	-0.2	-0.1	-0.2
Stbd1	0.1	-0.3	-0.1	0.3	0.5	1.2	1.8	1.4	-0.1	-0.9	-0.6
Stard5	-0.0	-0.2	-0.2	-0.1	0.1	1.4	0.9	1.1	-0.2	-0.5	-0.5
Slc25a39	0.1	0.0	-0.0	-0.1	-0.0	1.3	1.2	1.1	0.2	0.2	-0.2

Log2 FC
4
2
0
−2
−4

Appendix Figure S14. DELE1-dependent DEGs common skeletal and heart muscle show same pattern of activation in male and female hearts from P28 CHCHD10 G58R mice. Heatmap shows log2 FC for DELE1-dependent genes separately for male and female animals. Values were unadjusted for sex by ANCOVA. RNA-Seq data are also in Table S9.

Appendix Figure S15

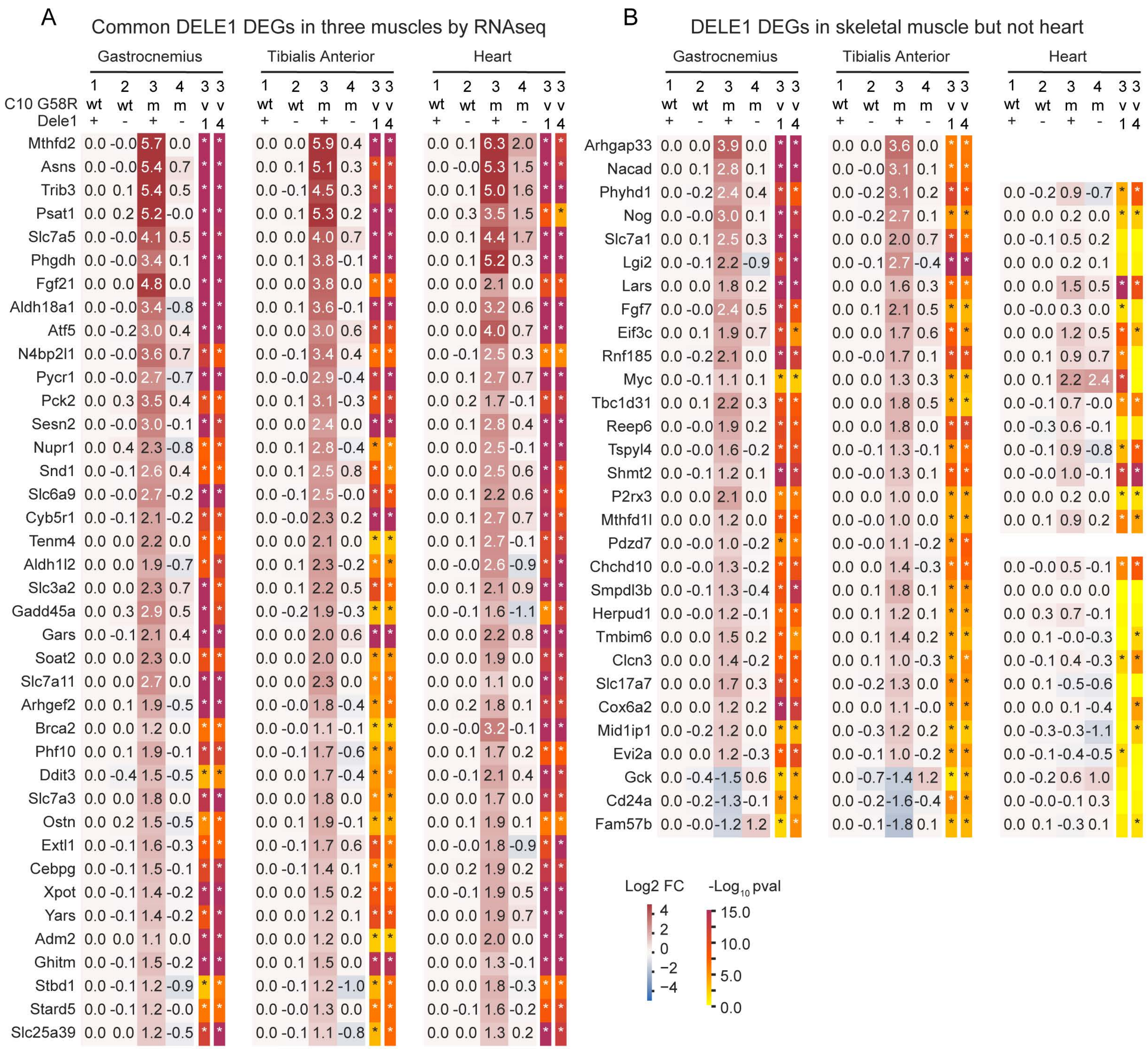

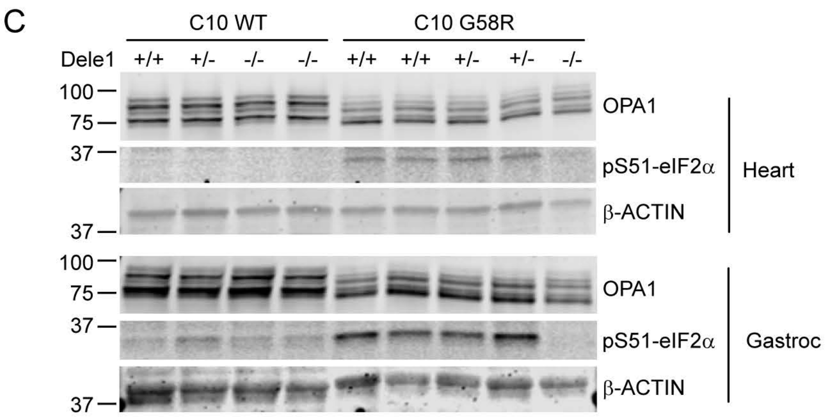

Appendix Figure S15. DELE1-dependent DEGs shared between skeletal and heart muscle and unique to skeletal muscle from P28 CHCHD10 G58R mice.

(A and B) Heat maps show shows $\log_2$ fold changes (FC) and adjusted $-\log_{10}$ p-values for DELE1-dependent DEGs shared between skeletal and heart muscle (A) or unique to skeletal muscle (B). Transcriptomic data are from P28 CHCHD10 G58R mice measured by RNA-Seq and are also in Tables S7 – 9. Statistical analysis was performed as described in the methods for RNA-Seq based transcriptomics.

(C) Immunoblot of heart (top) and gastrocnemius muscle (bottom) from P28 C10 G58R; *Dele1* KO mice and their littermates, showing DELE1-dependent phosphorylation of eIF2α.

Appendix Figure S16

C10 G58R; Dele1 KO gastrocnemius muscle

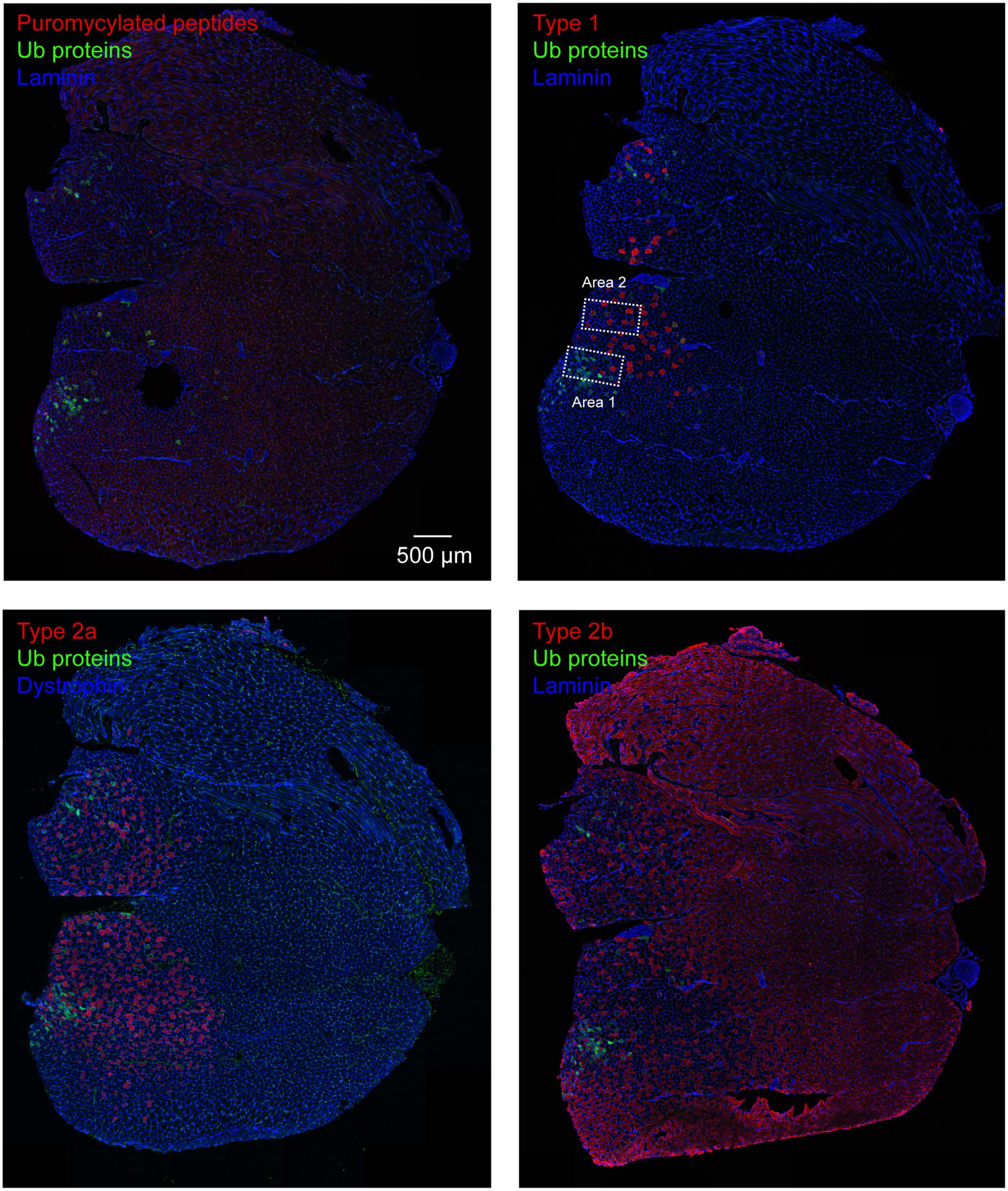

Appendix Figure S16. Muscle fibers with aggregates of ubiquitinated proteins affect each muscle fiber. Stitched 20X confocal images of consecutive whole sections from C10 G58R; Dele1 KO gastrocnemius muscle. Sections are the same that appear in Figure 7I; Areas 1 and 2 shown in Figure 7I are indicated with boxes in upper right image. Scale bar = 500 µm.

Appendix Figure S17

Breeding scheme

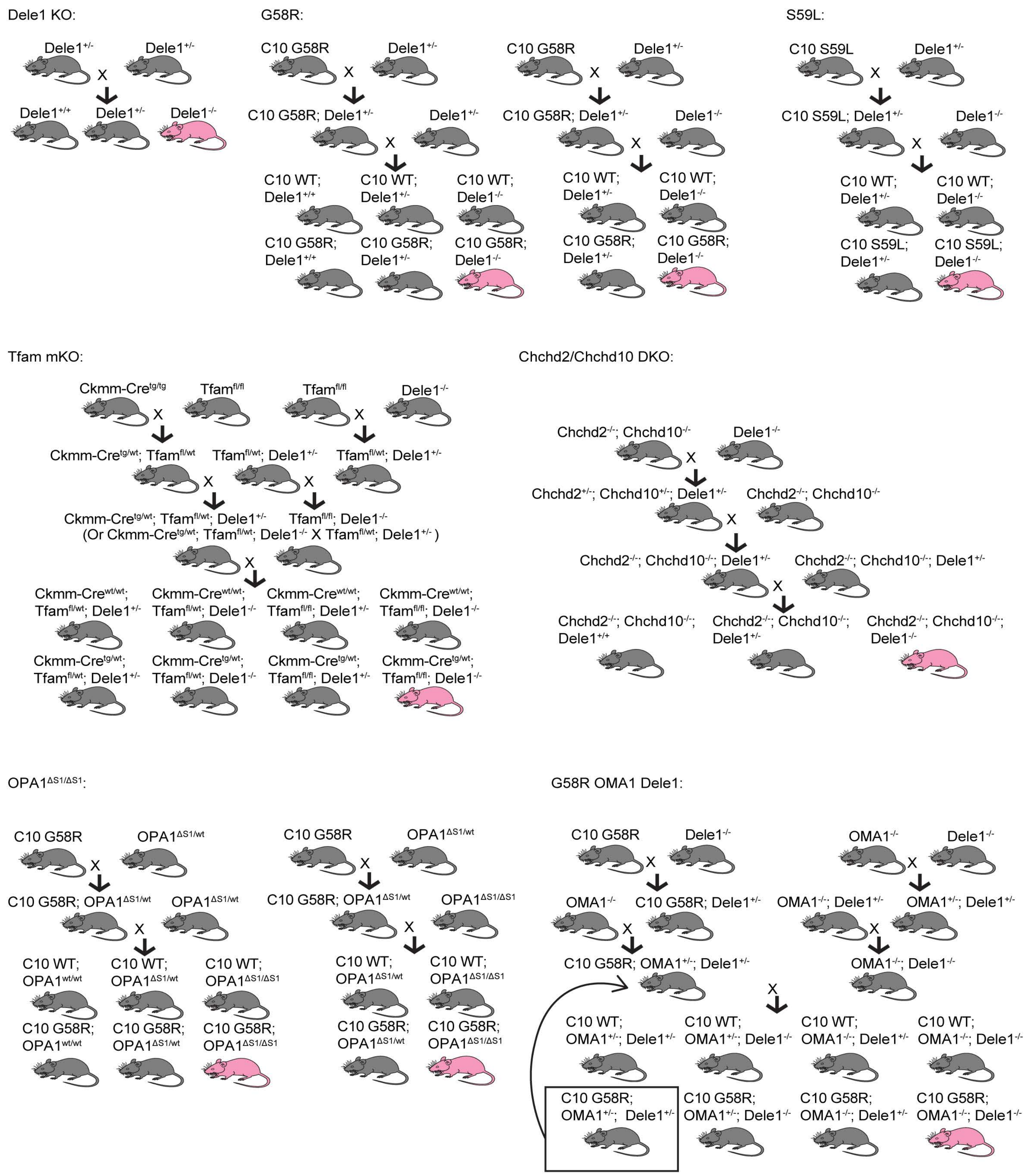

Appendix Figure S17. Crosses used in study. Schematic shows all crosses used in experiments.
