# Supplementary figures and images for: DELE1 maintains muscle proteostasis to promote growth and survival in mitochondrial myopathy (part 1 of 2)
Source: EMBO J. 2024 Oct 8;43(22):5548–85. doi: 10.1038/s44318-024-00242-x (PMC11574132; doi:10.1038/s44318-024-00242-x)

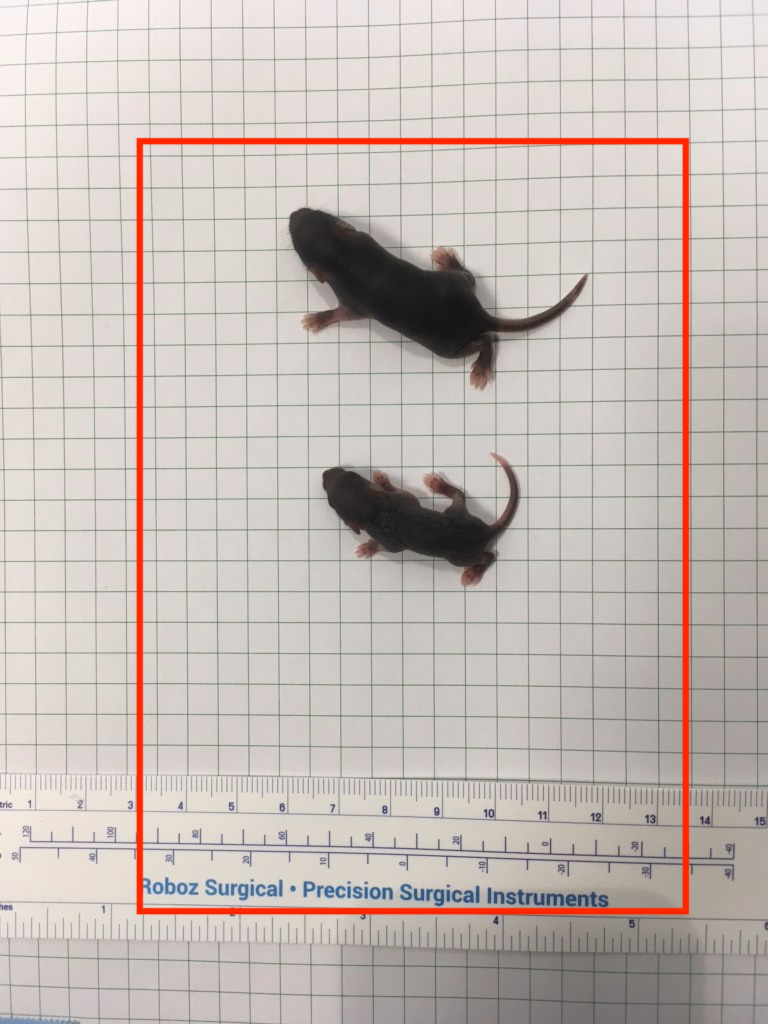

Supplement: Supplementary file 12 — Source data Fig. 1 [file 44318_2024_242_MOESM12_ESM.zip › Fig1/A/1A.tiff]

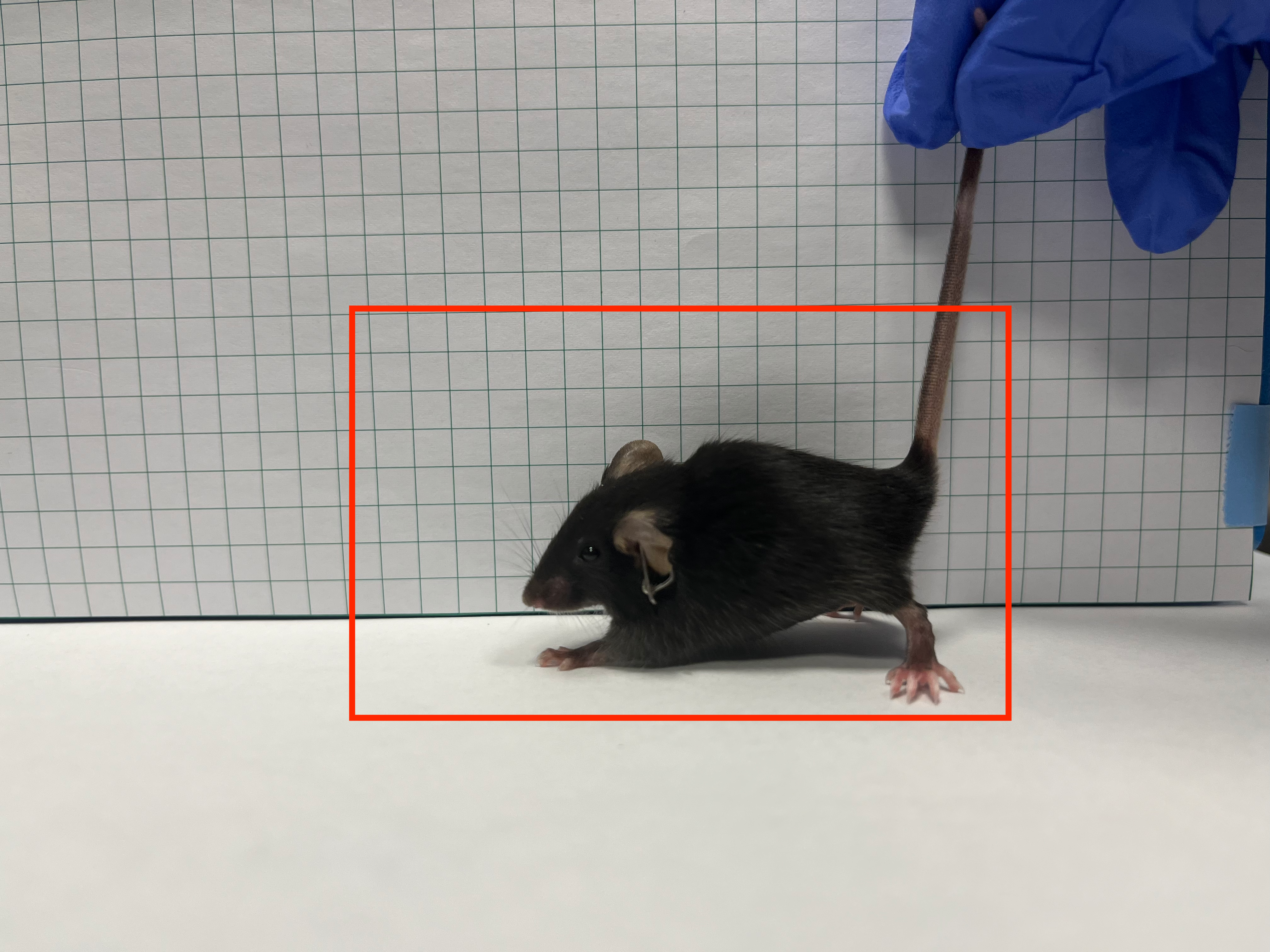

Supplement: Supplementary file 12 — Source data Fig. 1 [file 44318_2024_242_MOESM12_ESM.zip › Fig1/D/1D_Dele1+.tiff]

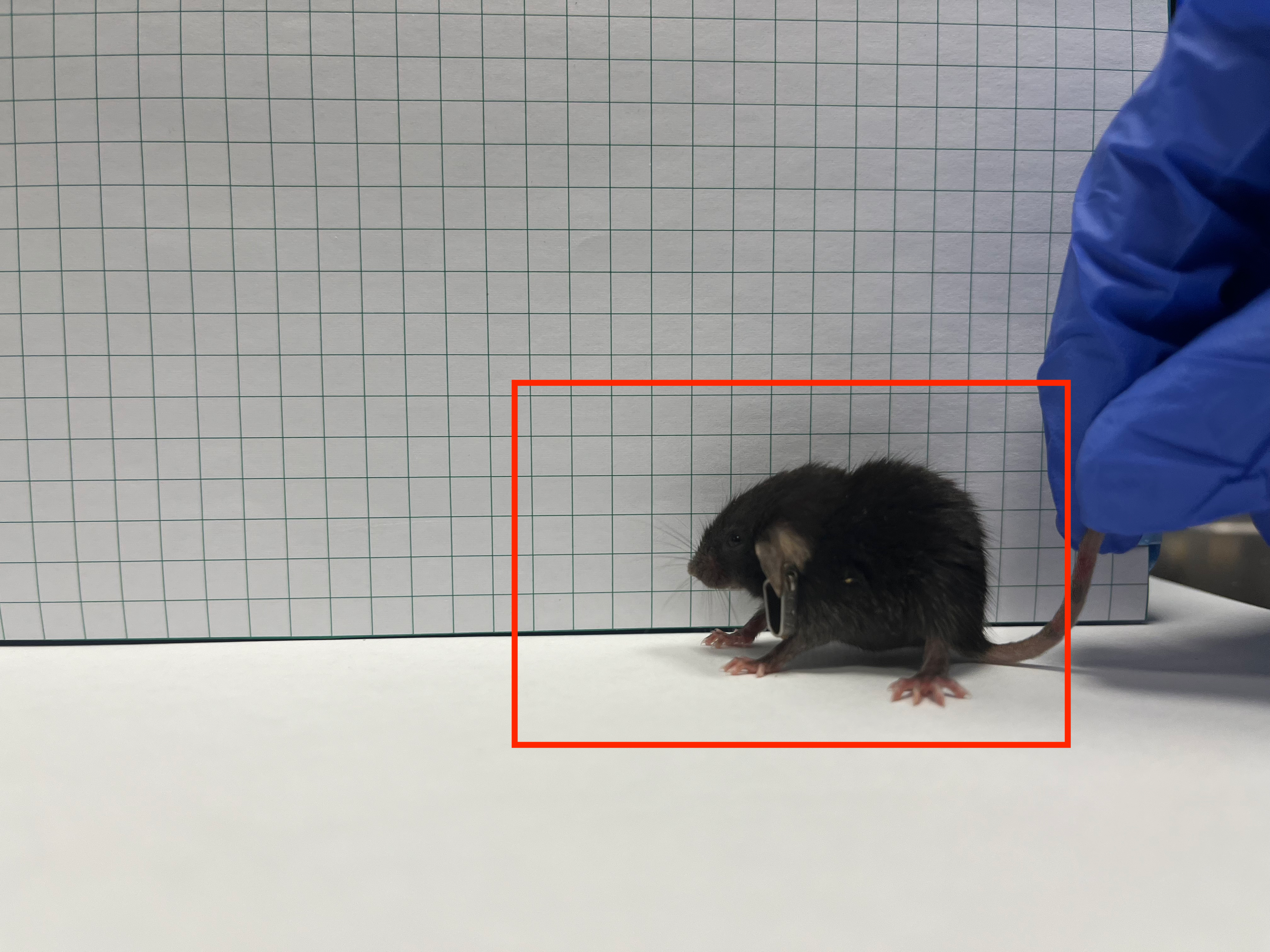

Supplement: Supplementary file 12 — Source data Fig. 1 [file 44318_2024_242_MOESM12_ESM.zip › Fig1/D/1D_Dele1KO.tiff]

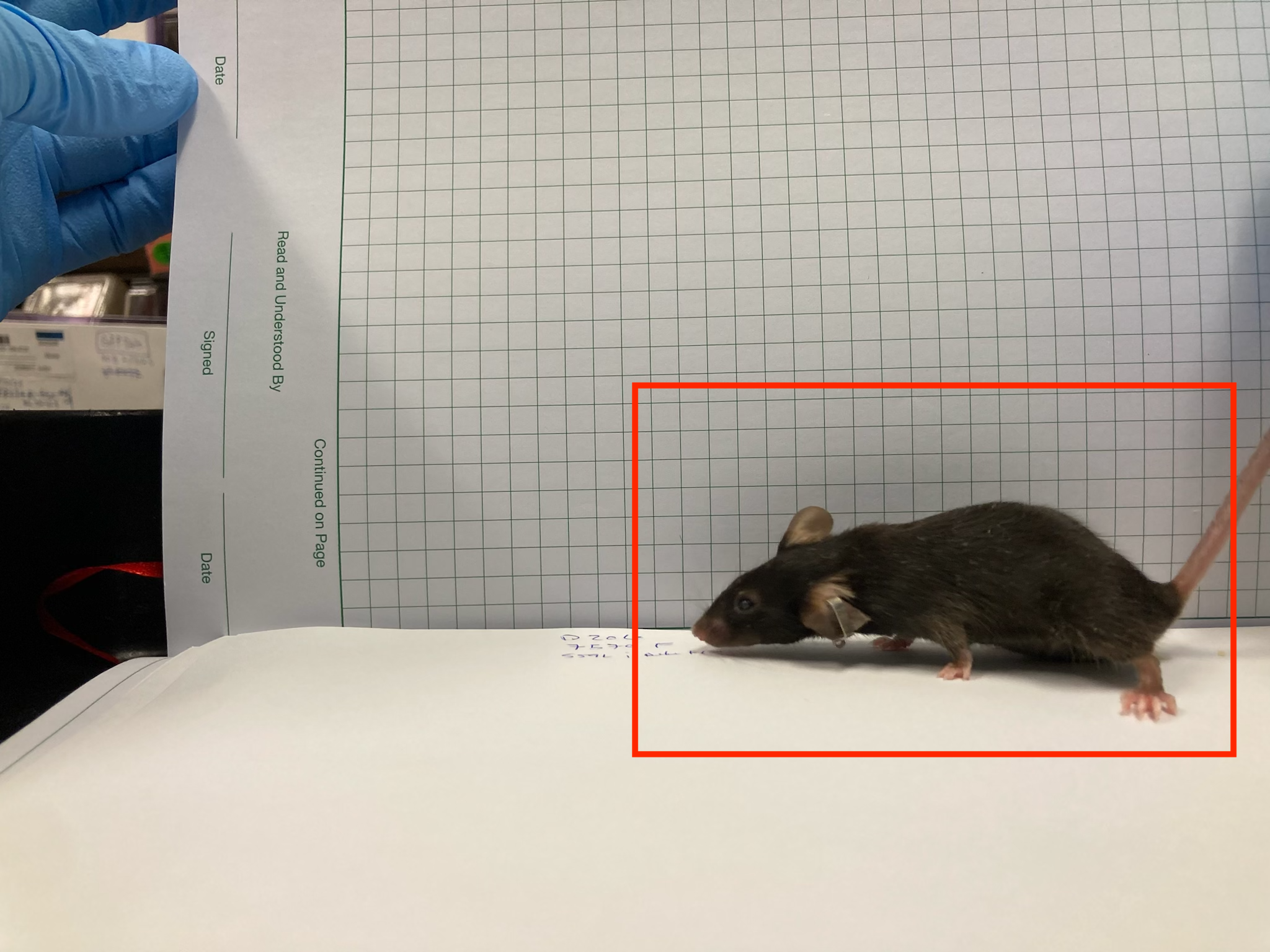

Supplement: Supplementary file 12 — Source data Fig. 1 [file 44318_2024_242_MOESM12_ESM.zip › Fig1/G/1G_Dele1+.tiff]

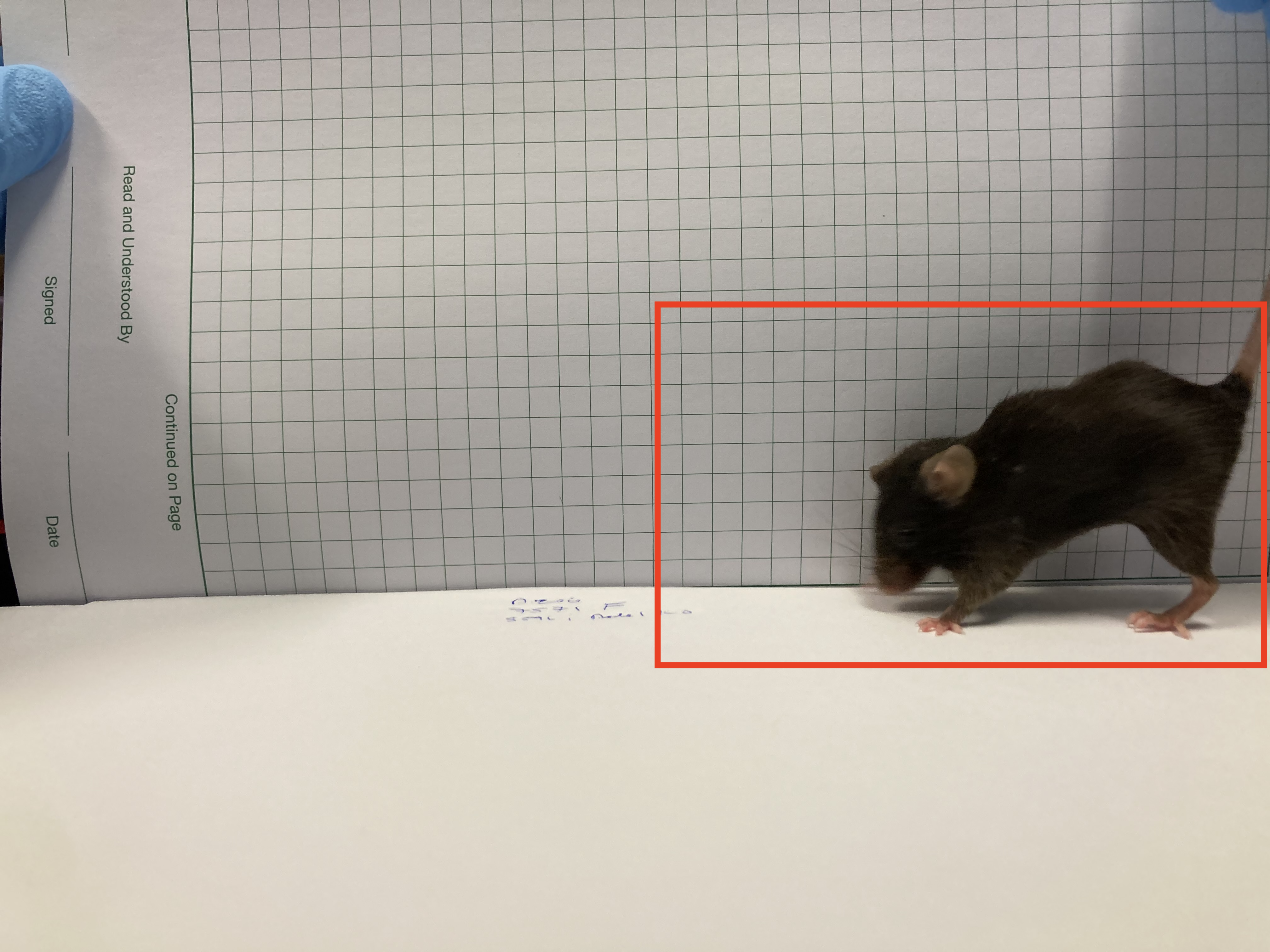

Supplement: Supplementary file 12 — Source data Fig. 1 [file 44318_2024_242_MOESM12_ESM.zip › Fig1/G/1G_Dele1KO.tiff]

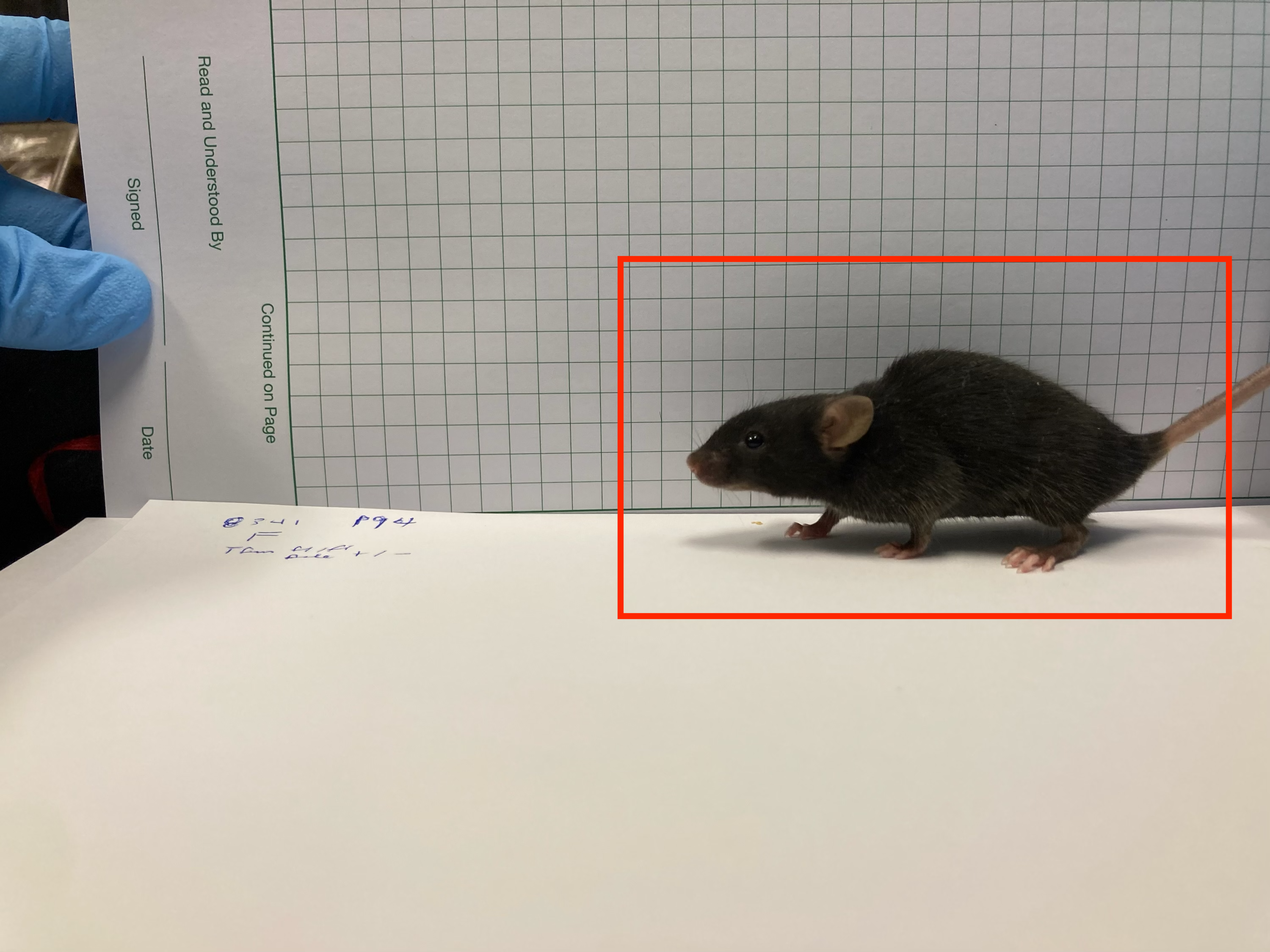

Supplement: Supplementary file 12 — Source data Fig. 1 [file 44318_2024_242_MOESM12_ESM.zip › Fig1/J/1J_Dele1+.tiff]

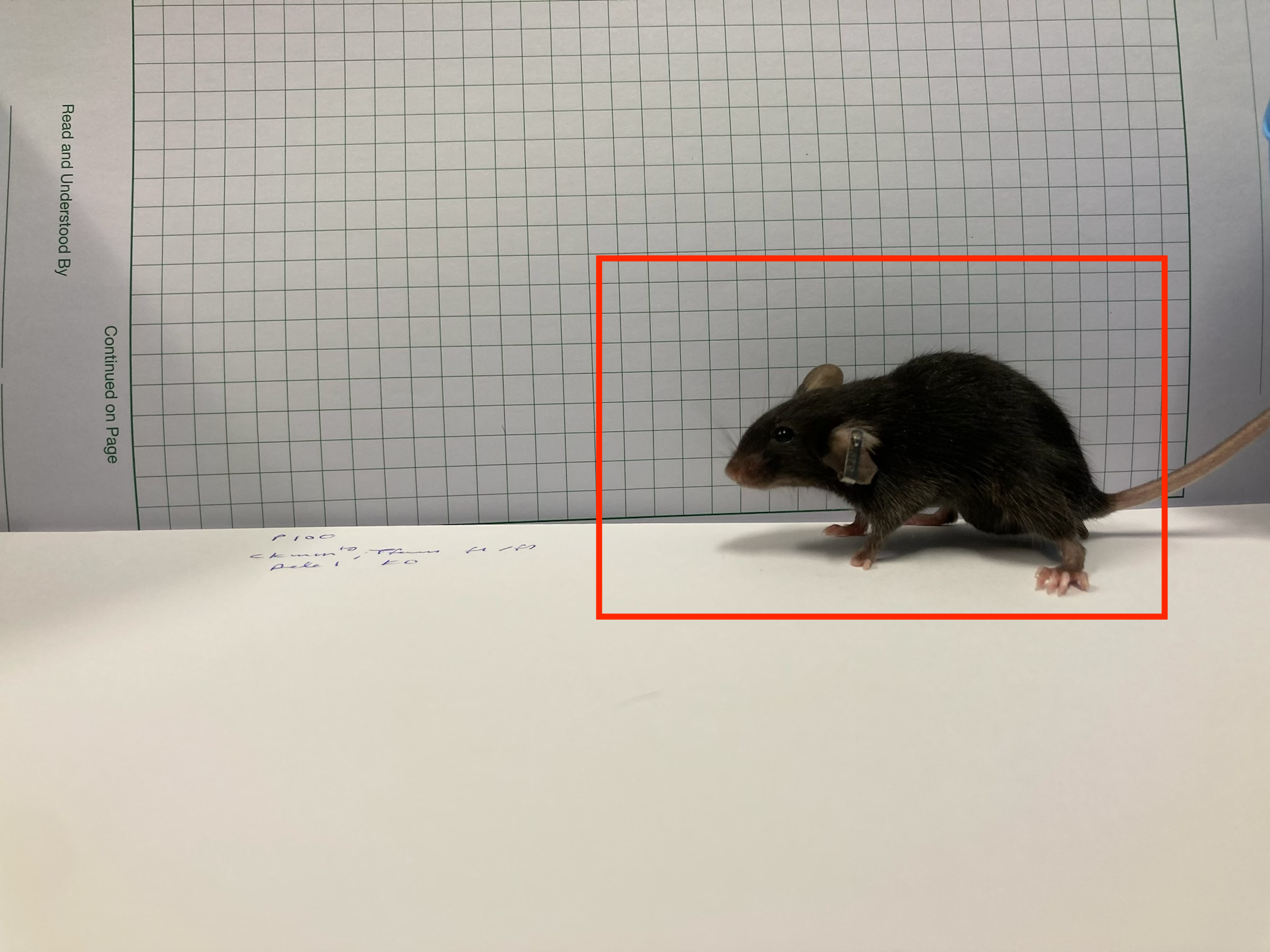

Supplement: Supplementary file 12 — Source data Fig. 1 [file 44318_2024_242_MOESM12_ESM.zip › Fig1/J/1J_Dele1KO.tiff]

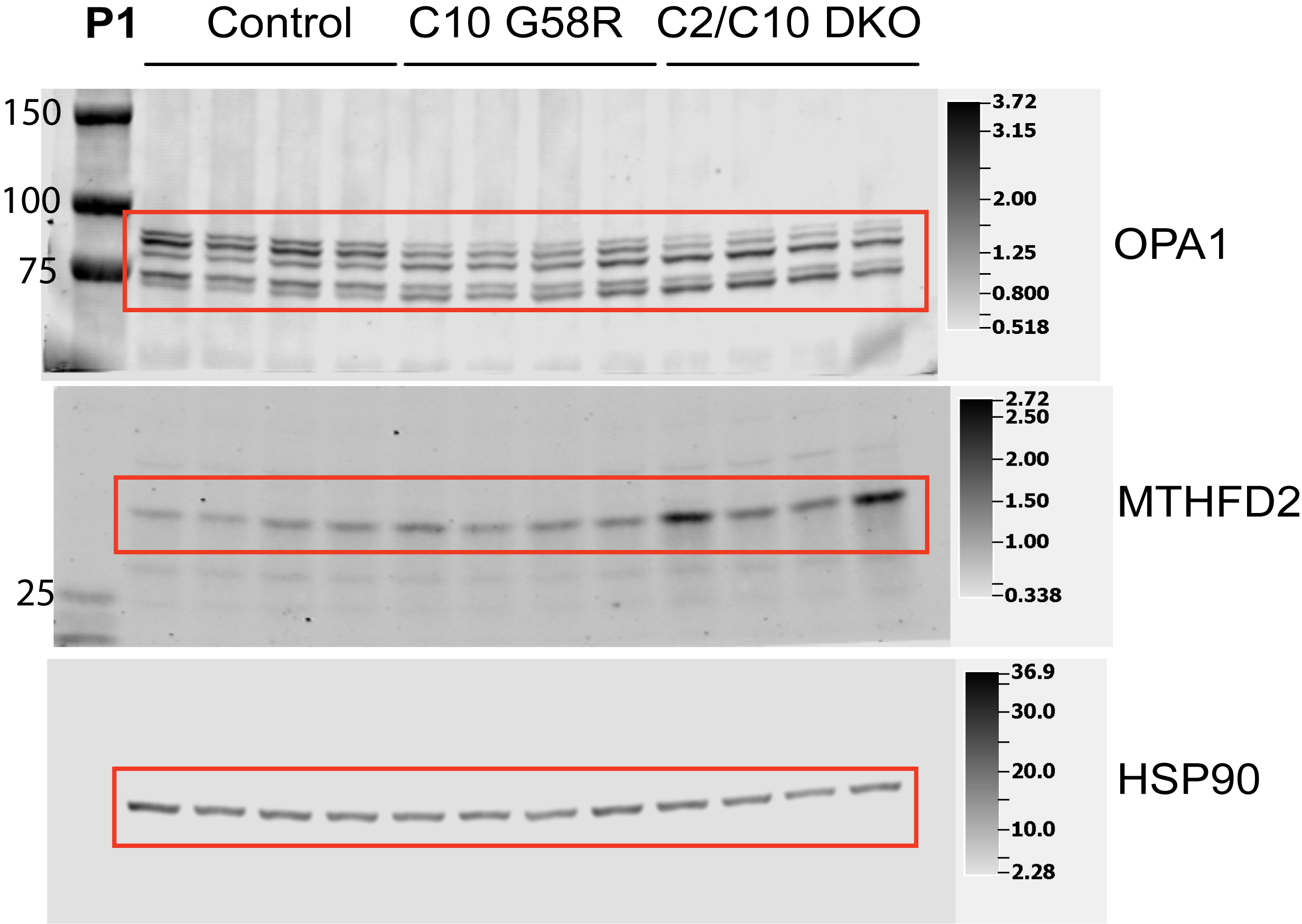

Supplement: Supplementary file 12 — Source data Fig. 1 [file 44318_2024_242_MOESM12_ESM.zip › Fig1/M/1M_P1.tif]

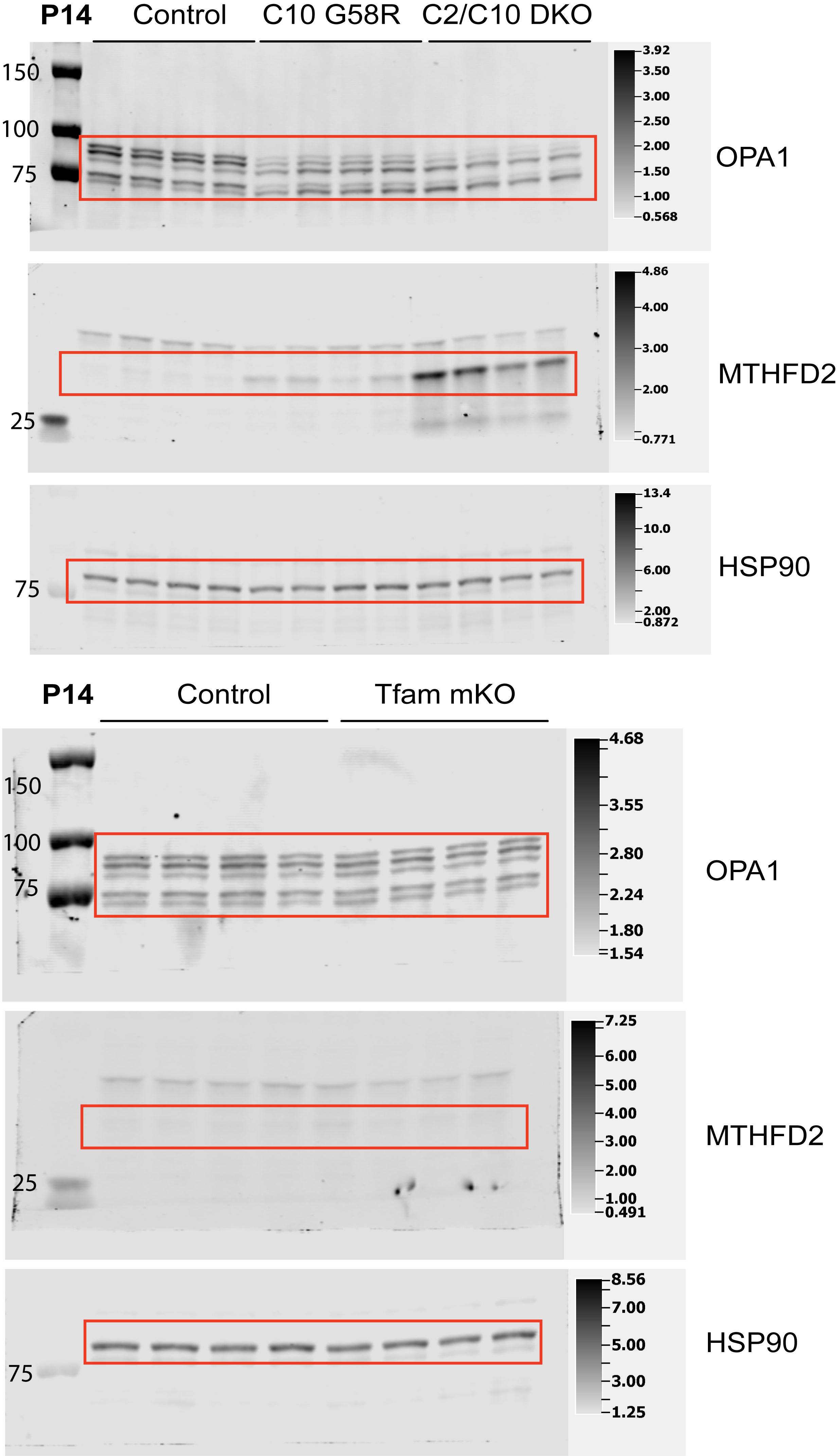

Supplement: Supplementary file 12 — Source data Fig. 1 [file 44318_2024_242_MOESM12_ESM.zip › Fig1/M/1M_P14.tif]

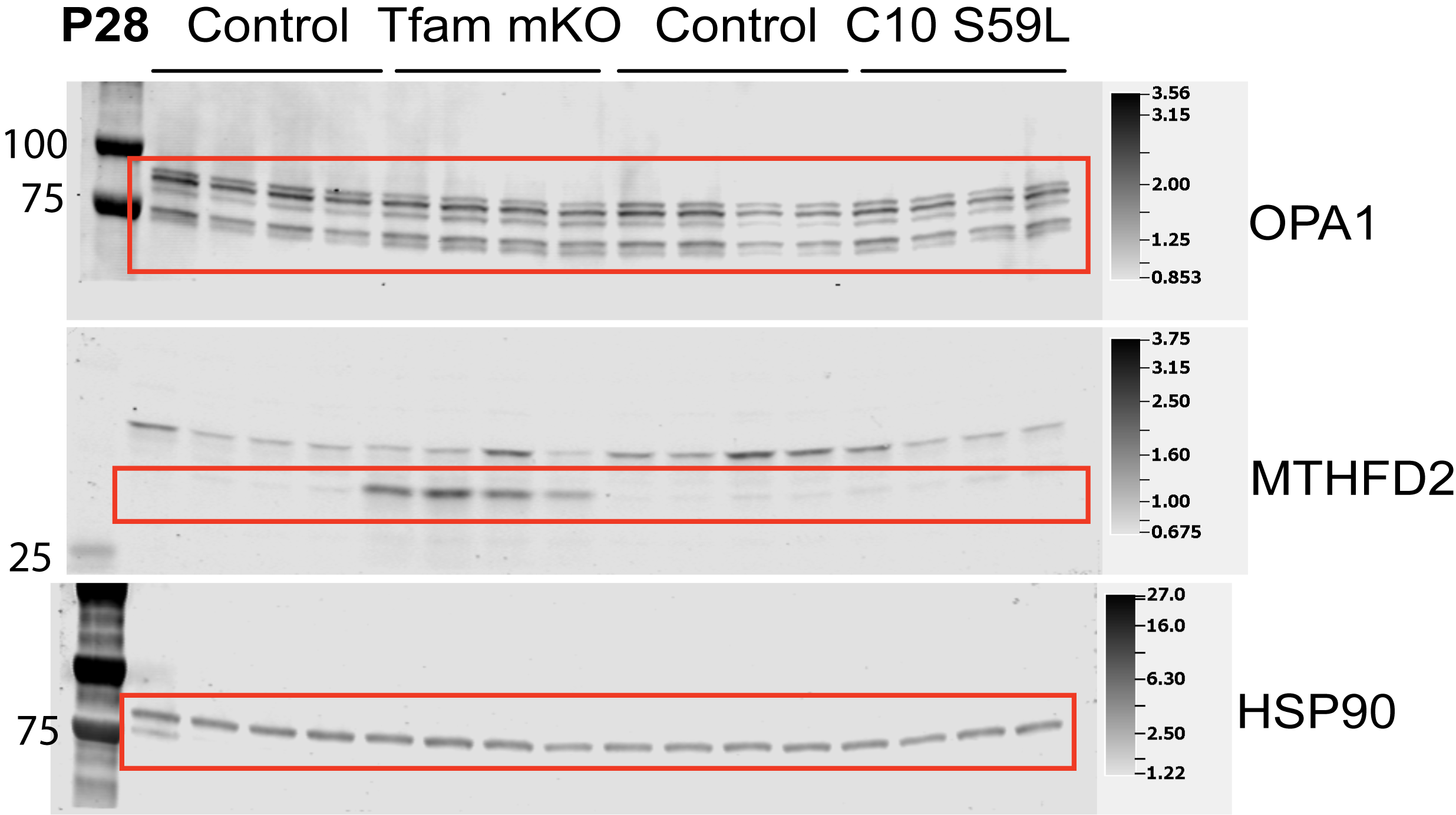

Supplement: Supplementary file 12 — Source data Fig. 1 [file 44318_2024_242_MOESM12_ESM.zip › Fig1/M/1M_P28.tif]

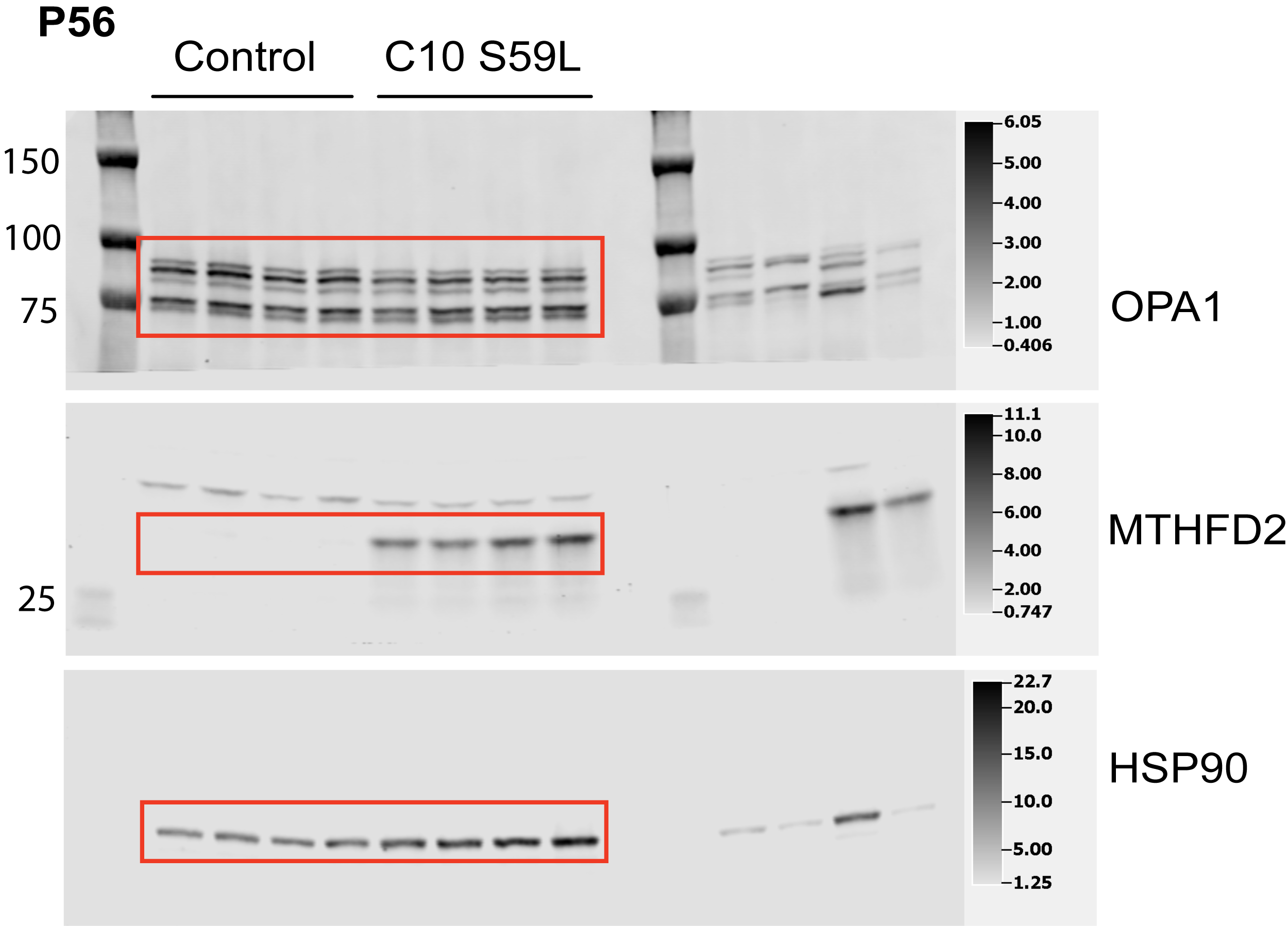

Supplement: Supplementary file 12 — Source data Fig. 1 [file 44318_2024_242_MOESM12_ESM.zip › Fig1/M/1M_P56.tif]

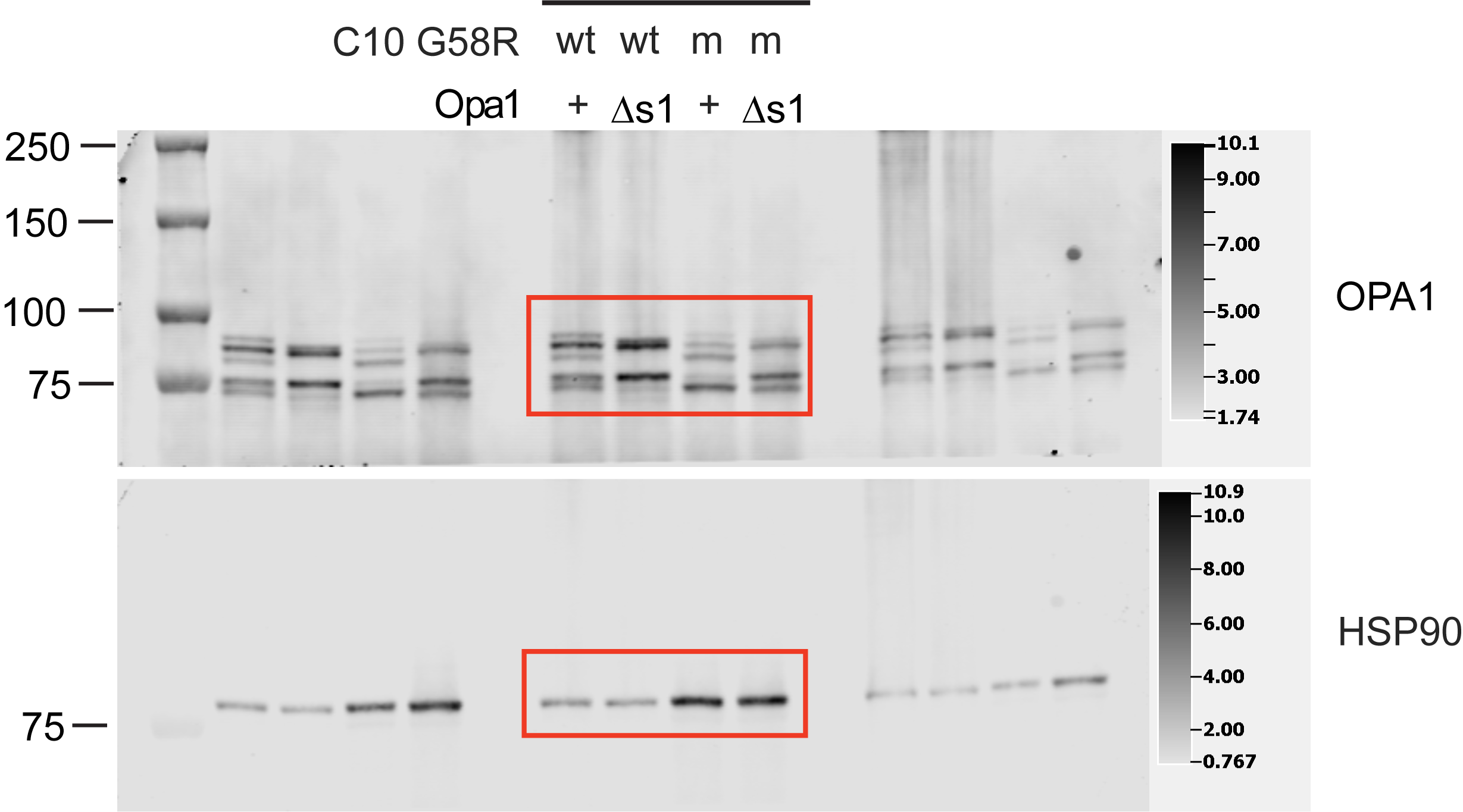

Supplement: Supplementary file 13 — Source data Fig. 2 [file 44318_2024_242_MOESM13_ESM.zip › Fig2/B/2B.tif]

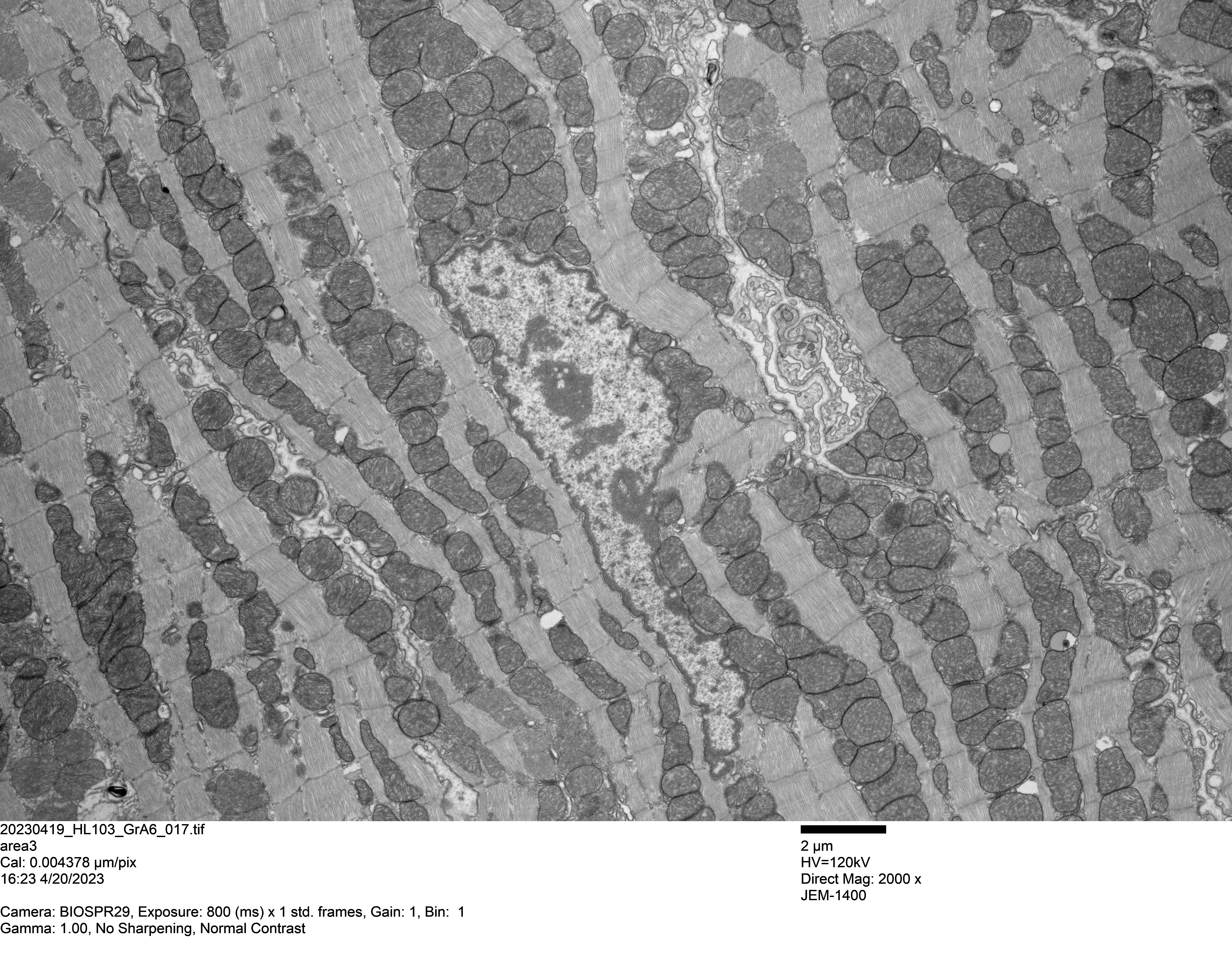

Supplement: Supplementary file 14 — Source data Fig. 3A [file 44318_2024_242_MOESM14_ESM.zip › Fig3A/2000x_view.tif]

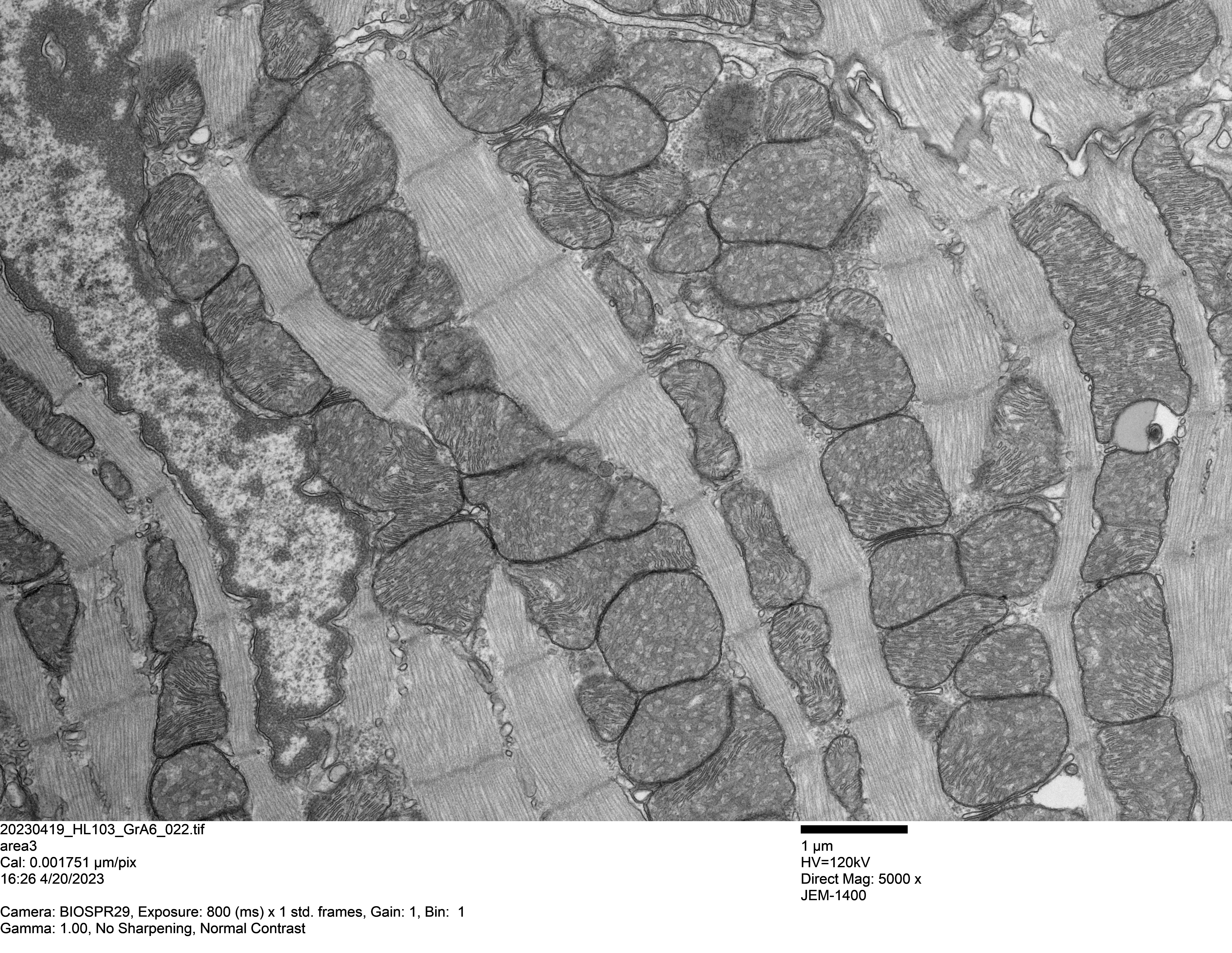

Supplement: Supplementary file 14 — Source data Fig. 3A [file 44318_2024_242_MOESM14_ESM.zip › Fig3A/5000x_view1.tif]

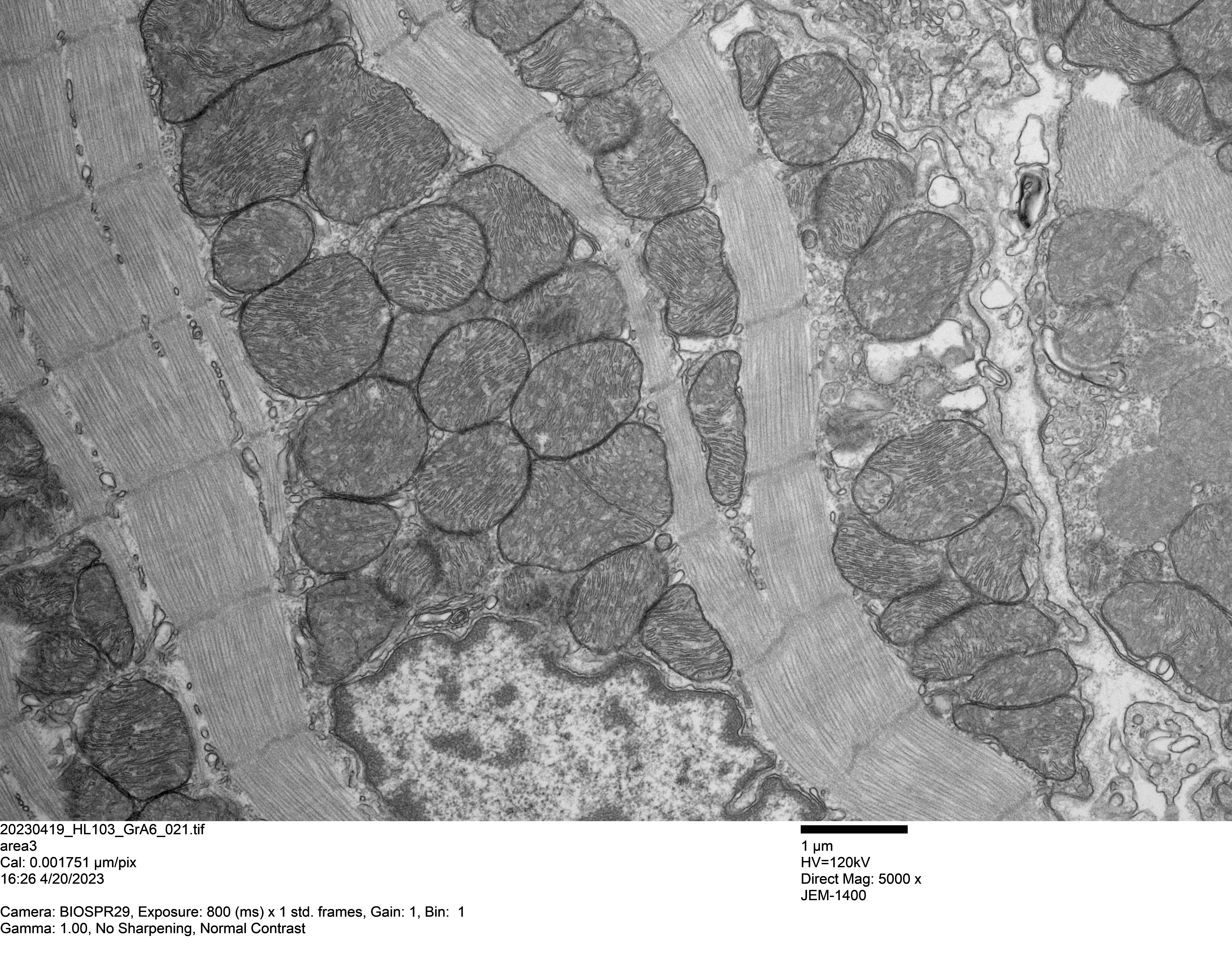

Supplement: Supplementary file 14 — Source data Fig. 3A [file 44318_2024_242_MOESM14_ESM.zip › Fig3A/5000x_view2.tif]

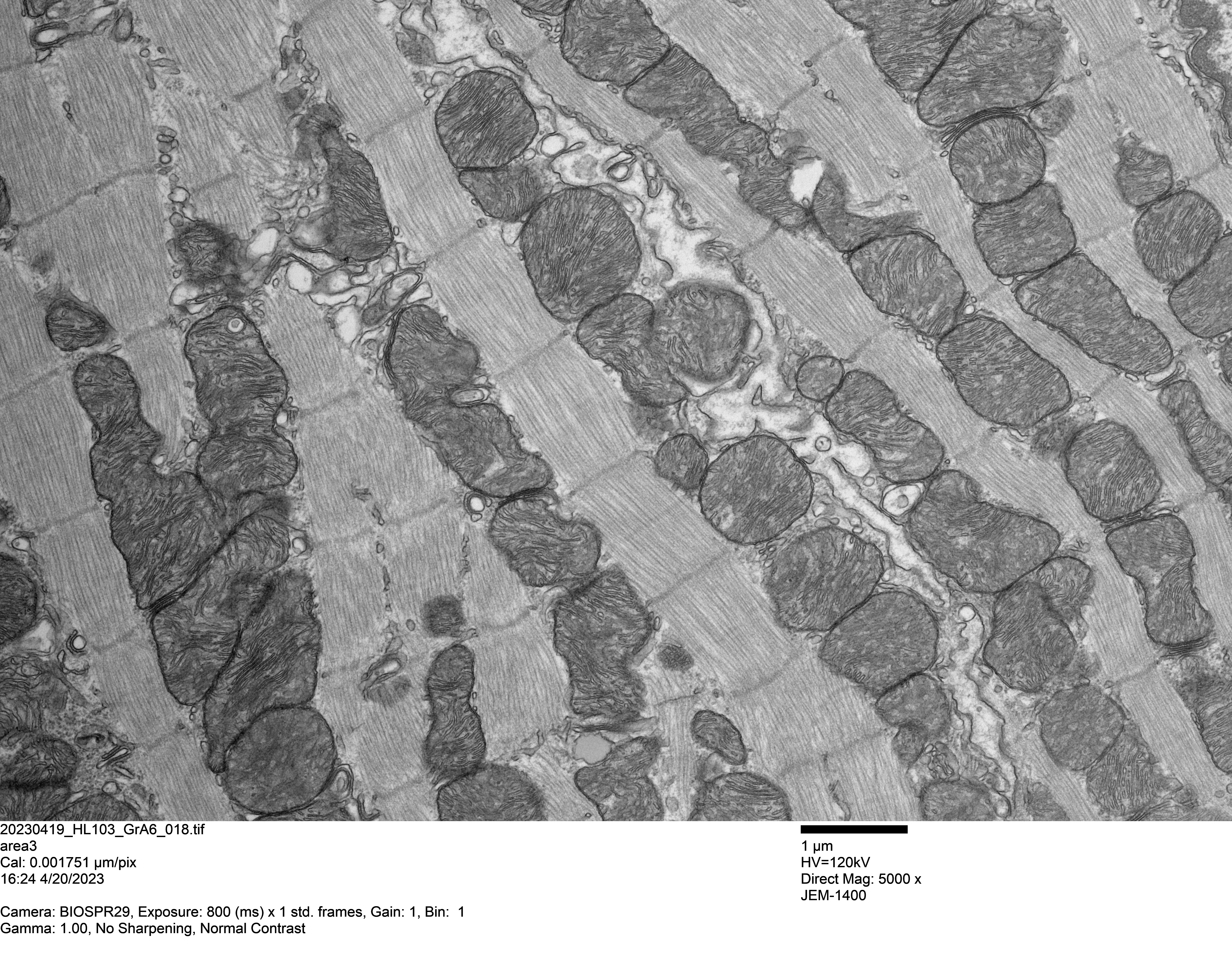

Supplement: Supplementary file 14 — Source data Fig. 3A [file 44318_2024_242_MOESM14_ESM.zip › Fig3A/5000x_view3.tif]

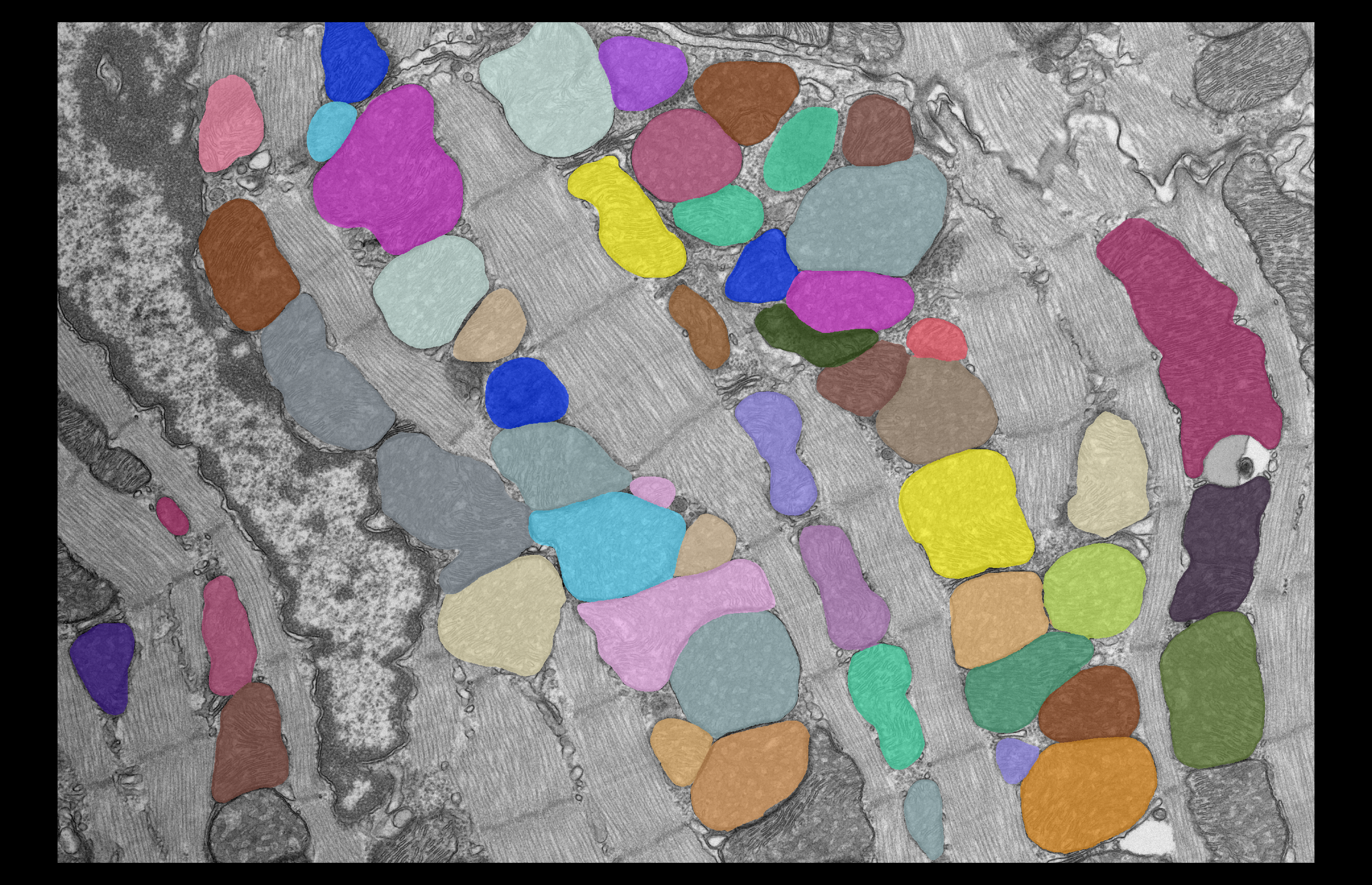

Supplement: Supplementary file 14 — Source data Fig. 3A [file 44318_2024_242_MOESM14_ESM.zip › Fig3A/Labels1.png]

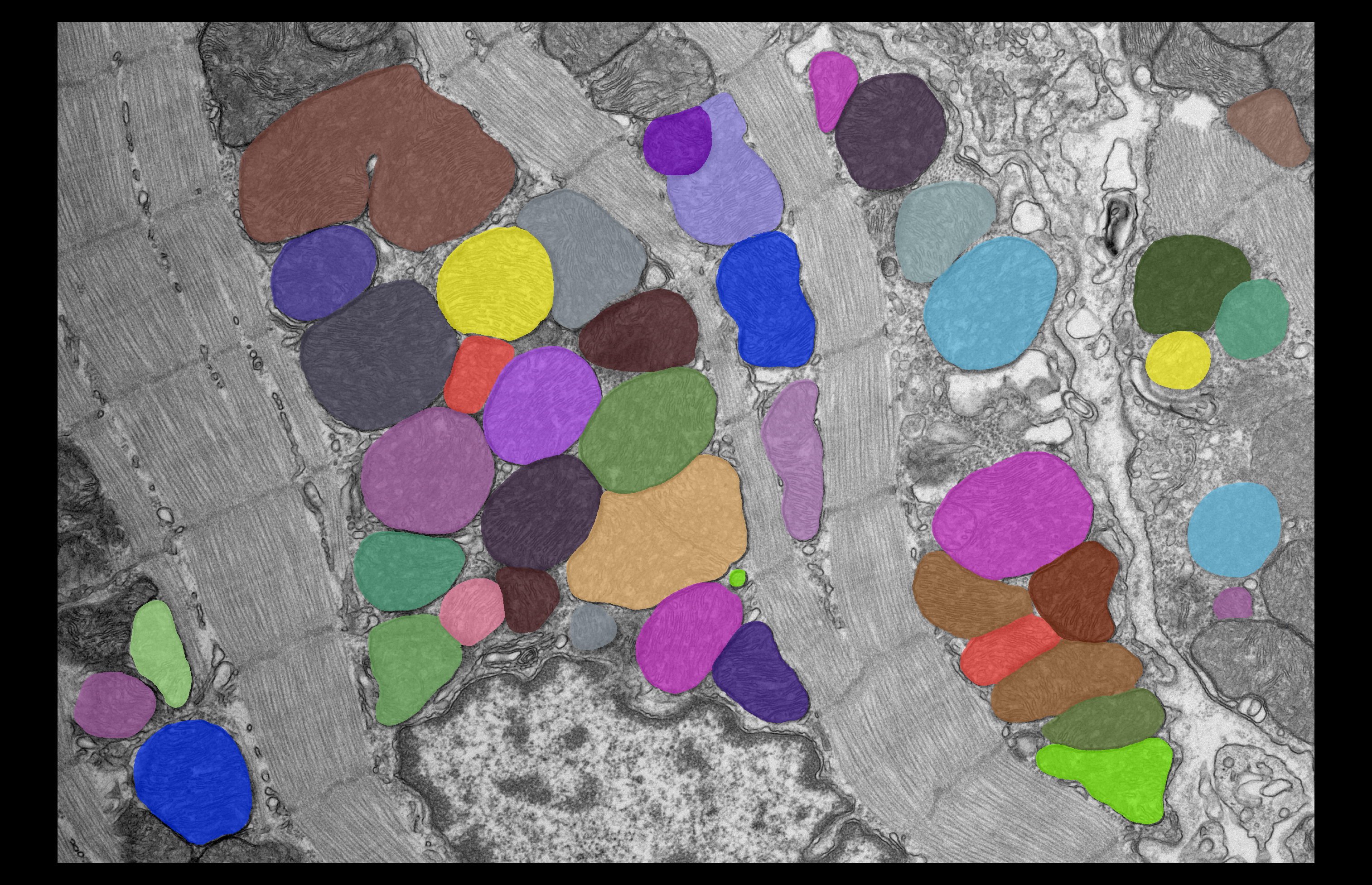

Supplement: Supplementary file 14 — Source data Fig. 3A [file 44318_2024_242_MOESM14_ESM.zip › Fig3A/Labels2.png]

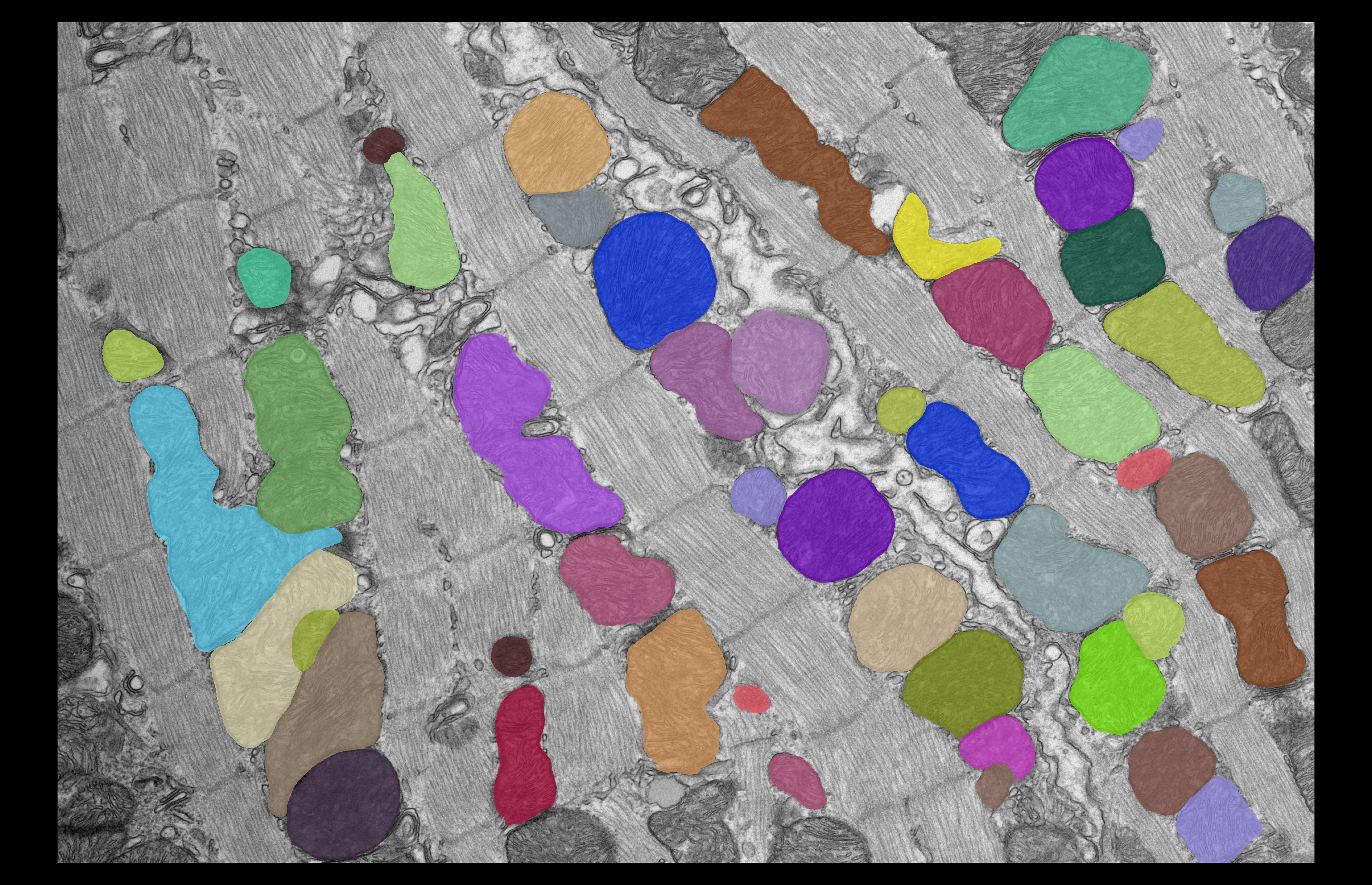

Supplement: Supplementary file 14 — Source data Fig. 3A [file 44318_2024_242_MOESM14_ESM.zip › Fig3A/Labels3.png]

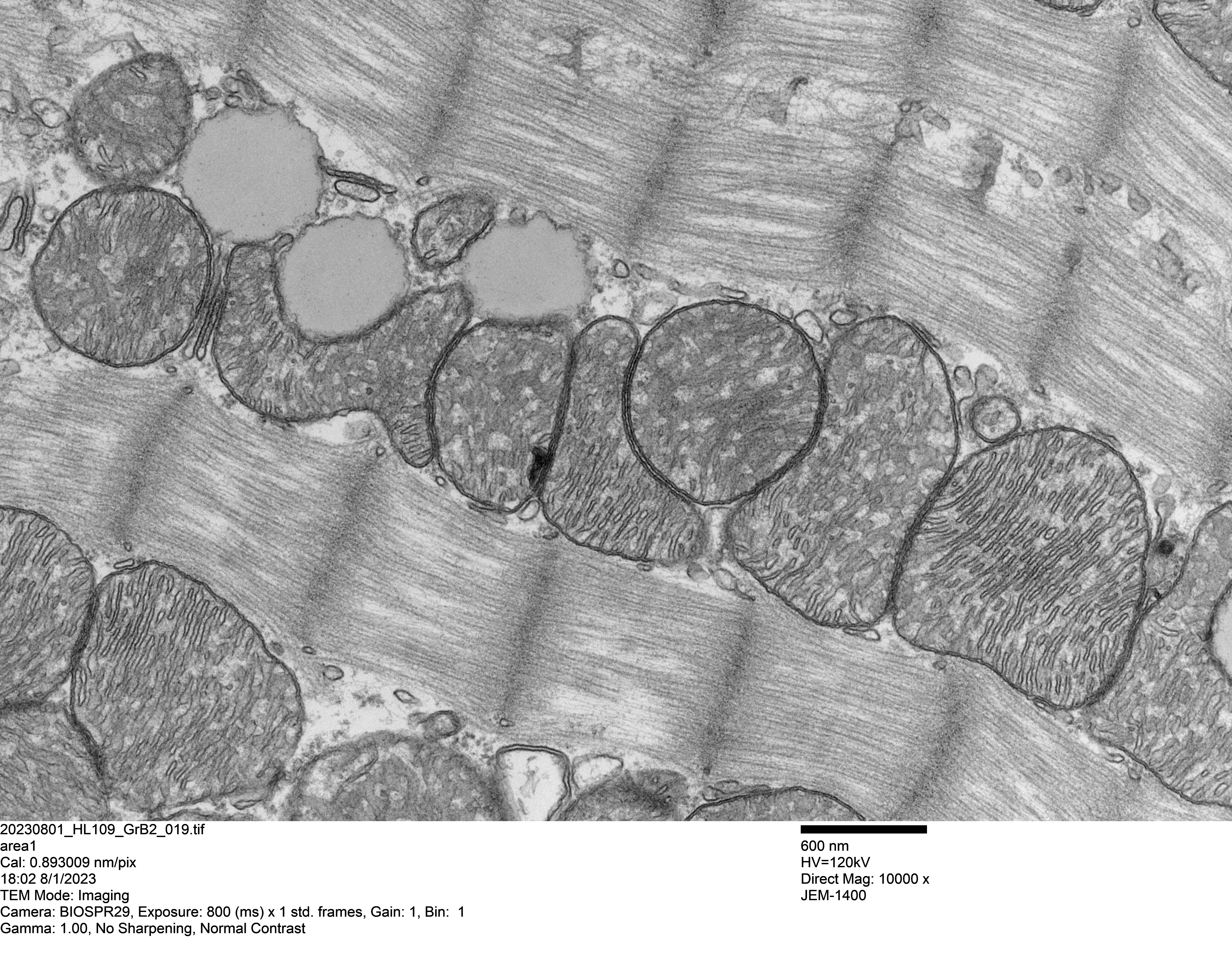

Supplement: Supplementary file 15 — Source data Fig. 3B [file 44318_2024_242_MOESM15_ESM.zip › Fig3B/Fig3B_WT_bottom.tif]

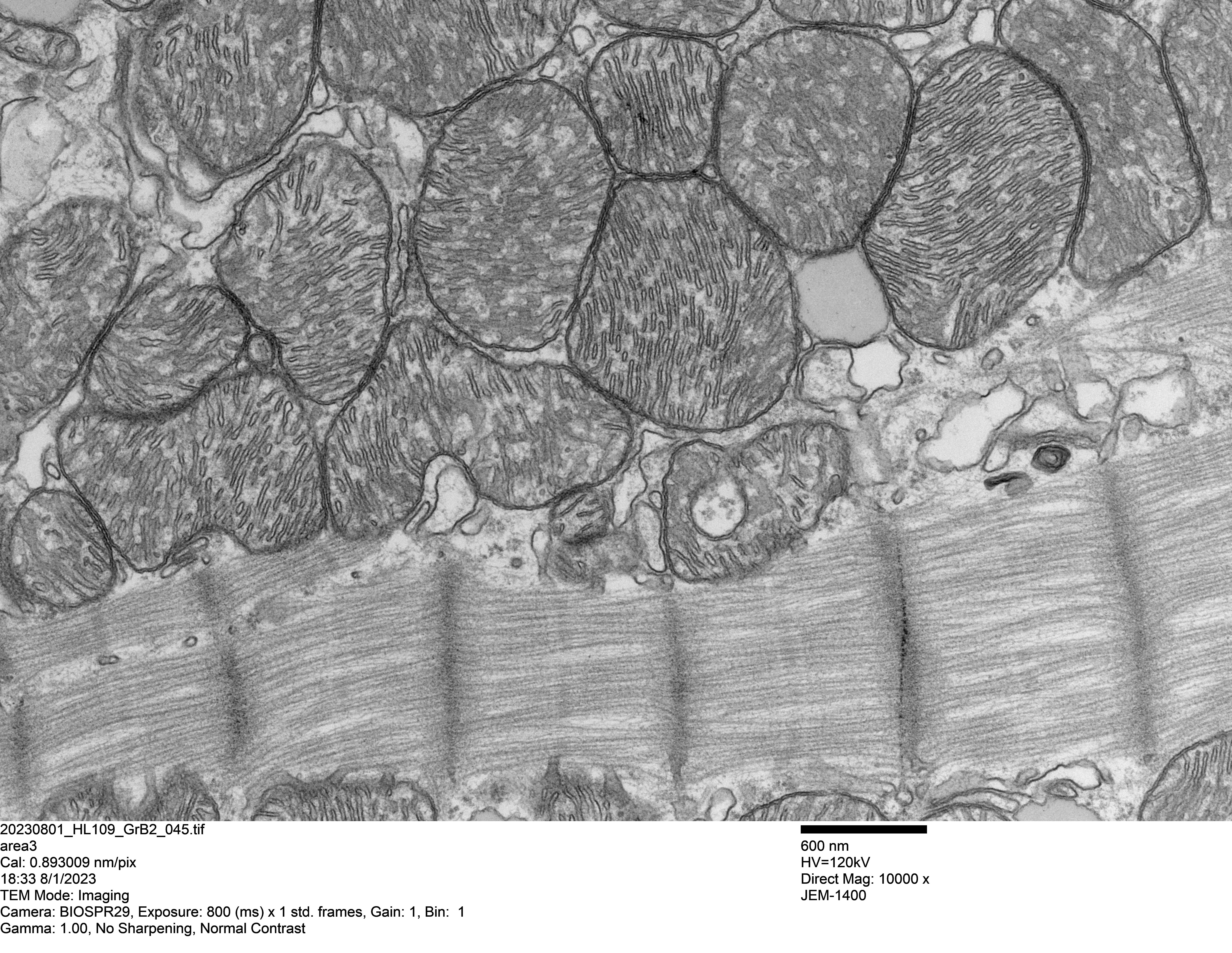

Supplement: Supplementary file 15 — Source data Fig. 3B [file 44318_2024_242_MOESM15_ESM.zip › Fig3B/Fig3B_WT_middle.tif]

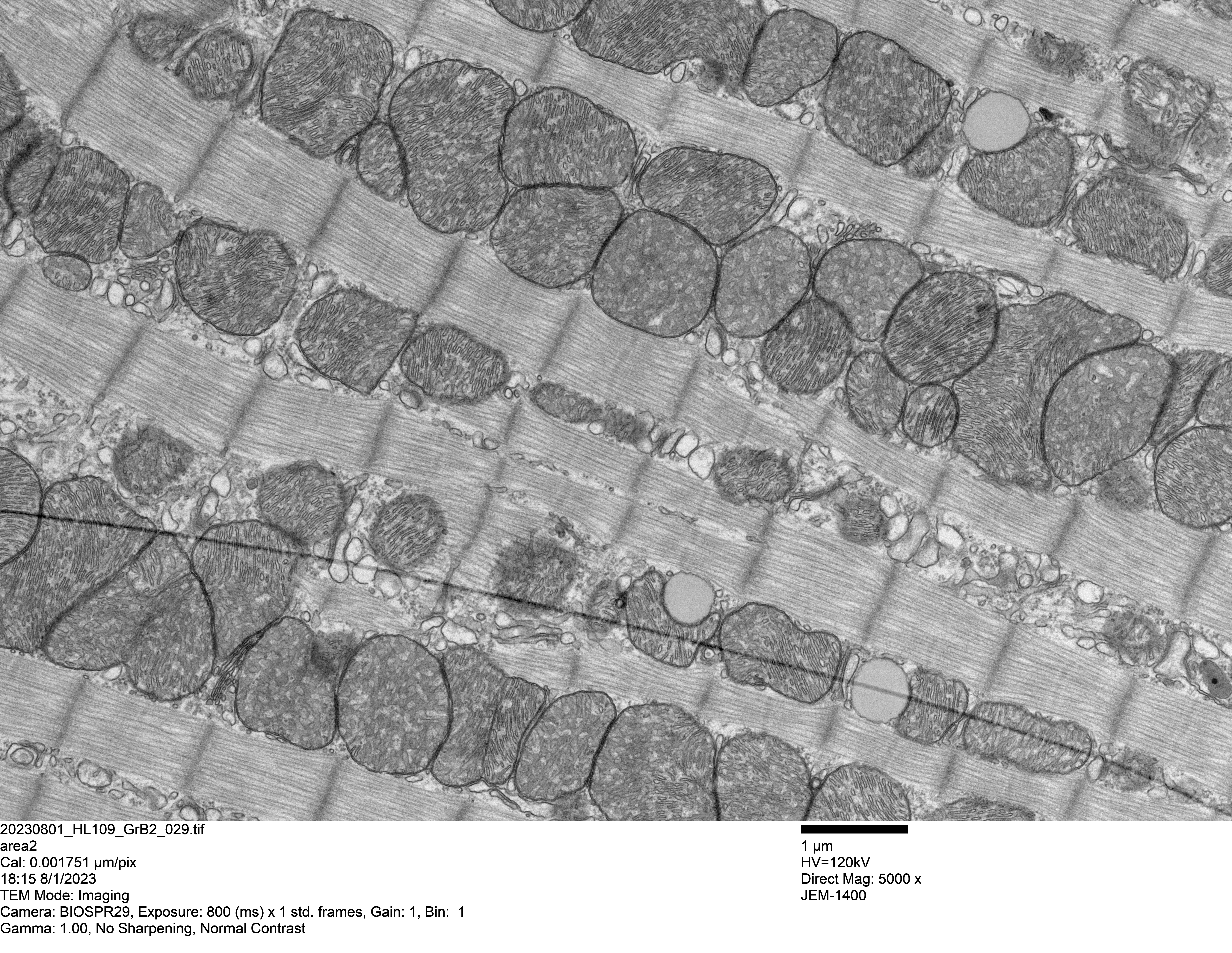

Supplement: Supplementary file 15 — Source data Fig. 3B [file 44318_2024_242_MOESM15_ESM.zip › Fig3B/Fig3B_WT_top.tif]

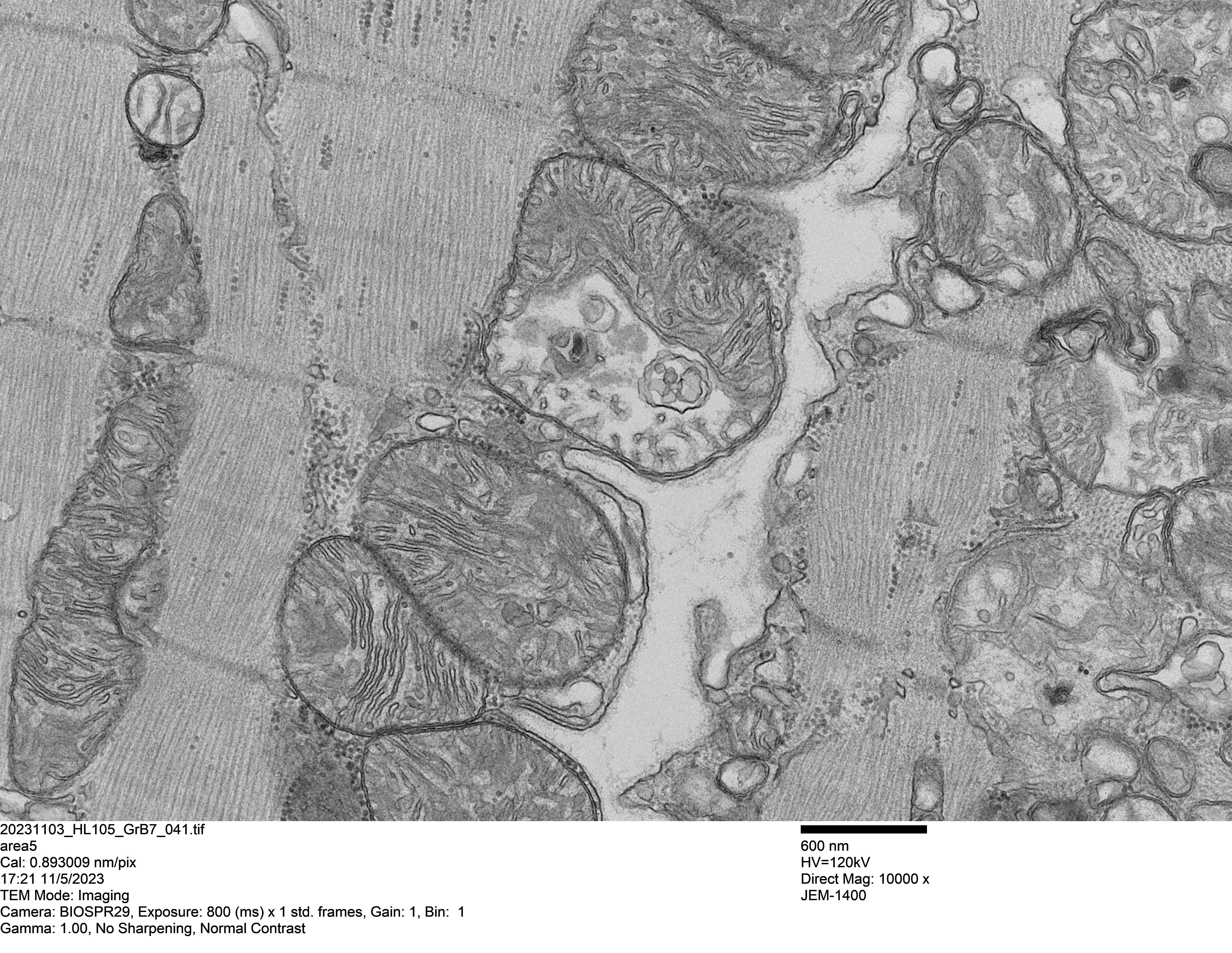

Supplement: Supplementary file 16 — Source data Fig. 3C [file 44318_2024_242_MOESM16_ESM.zip › Fig3C/Fig3C_G58R_bottom.tif]

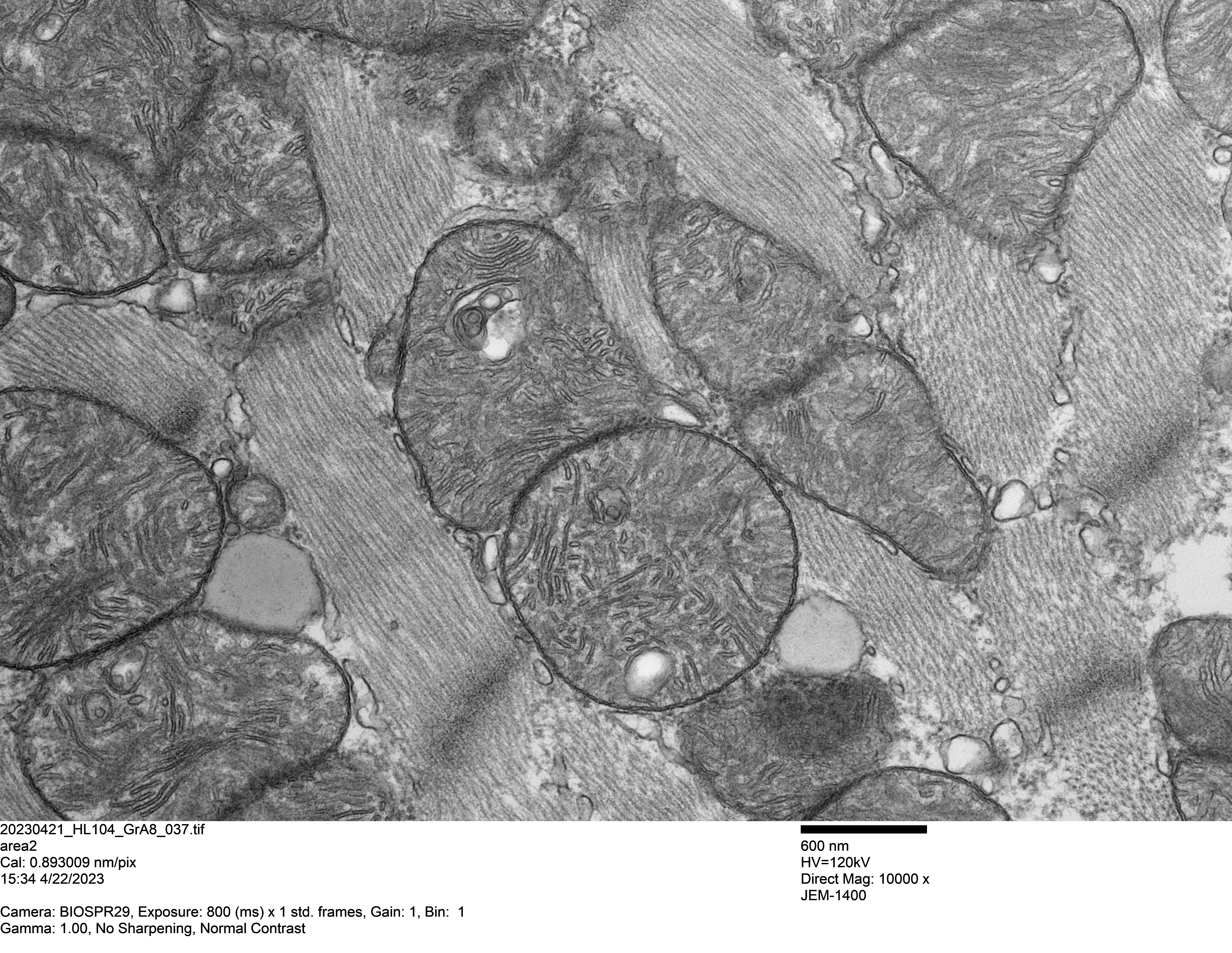

Supplement: Supplementary file 16 — Source data Fig. 3C [file 44318_2024_242_MOESM16_ESM.zip › Fig3C/Fig3C_G58R_middle.tif]

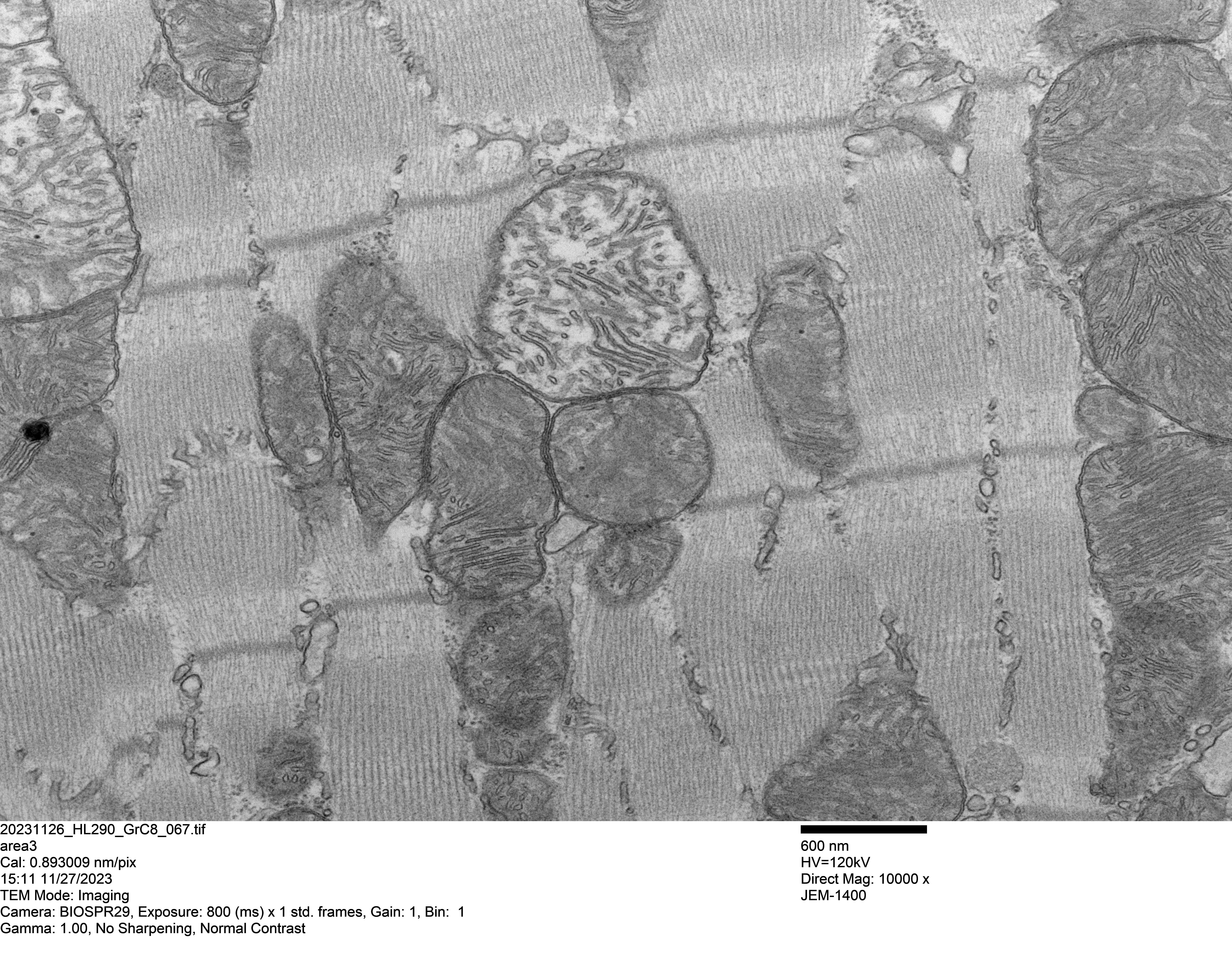

Supplement: Supplementary file 17 — Source data Fig. 3D [file 44318_2024_242_MOESM17_ESM.zip › Fig3D/Fig3D_DKO_bottom.tif]

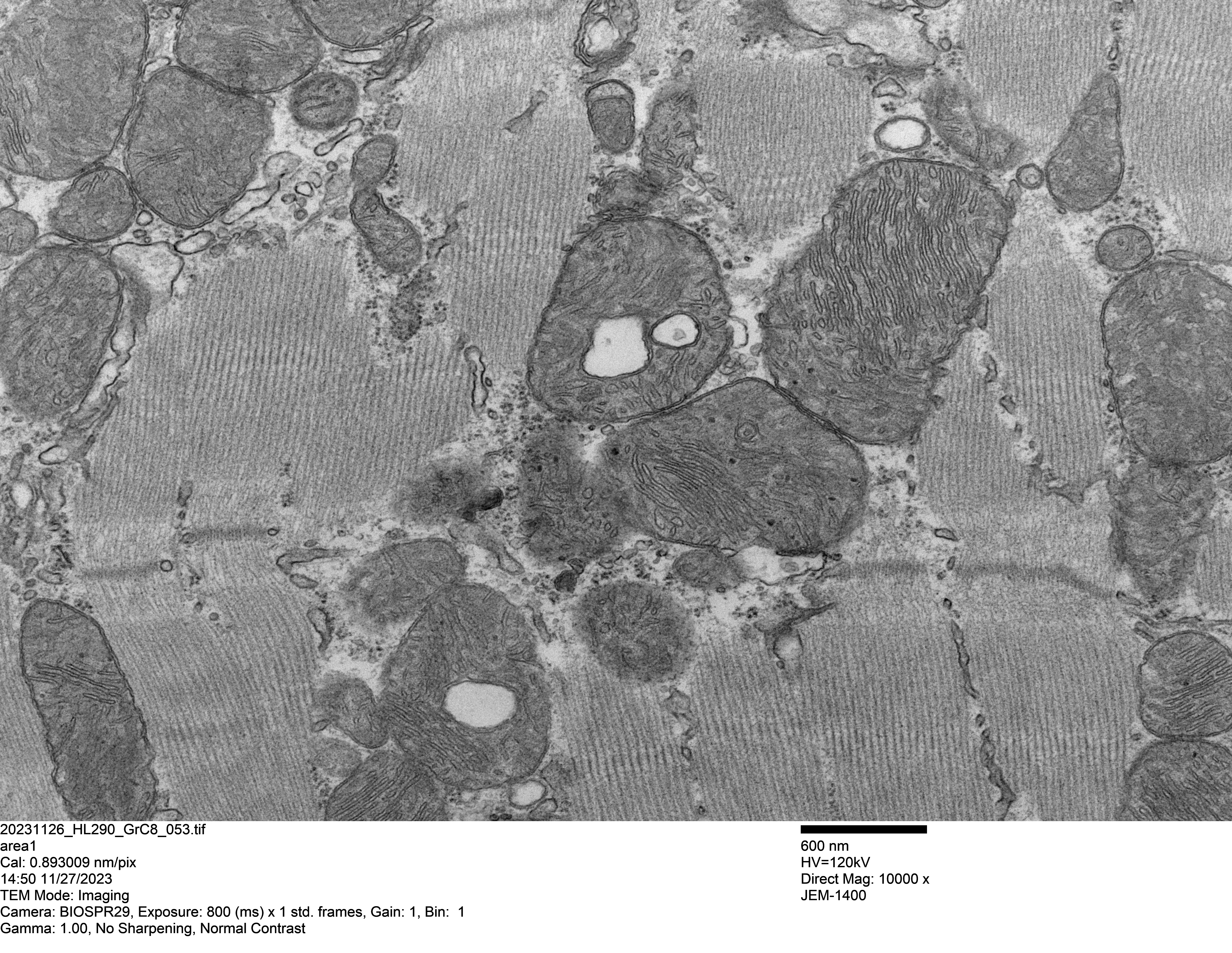

Supplement: Supplementary file 17 — Source data Fig. 3D [file 44318_2024_242_MOESM17_ESM.zip › Fig3D/Fig3D_DKO_middle.tif]

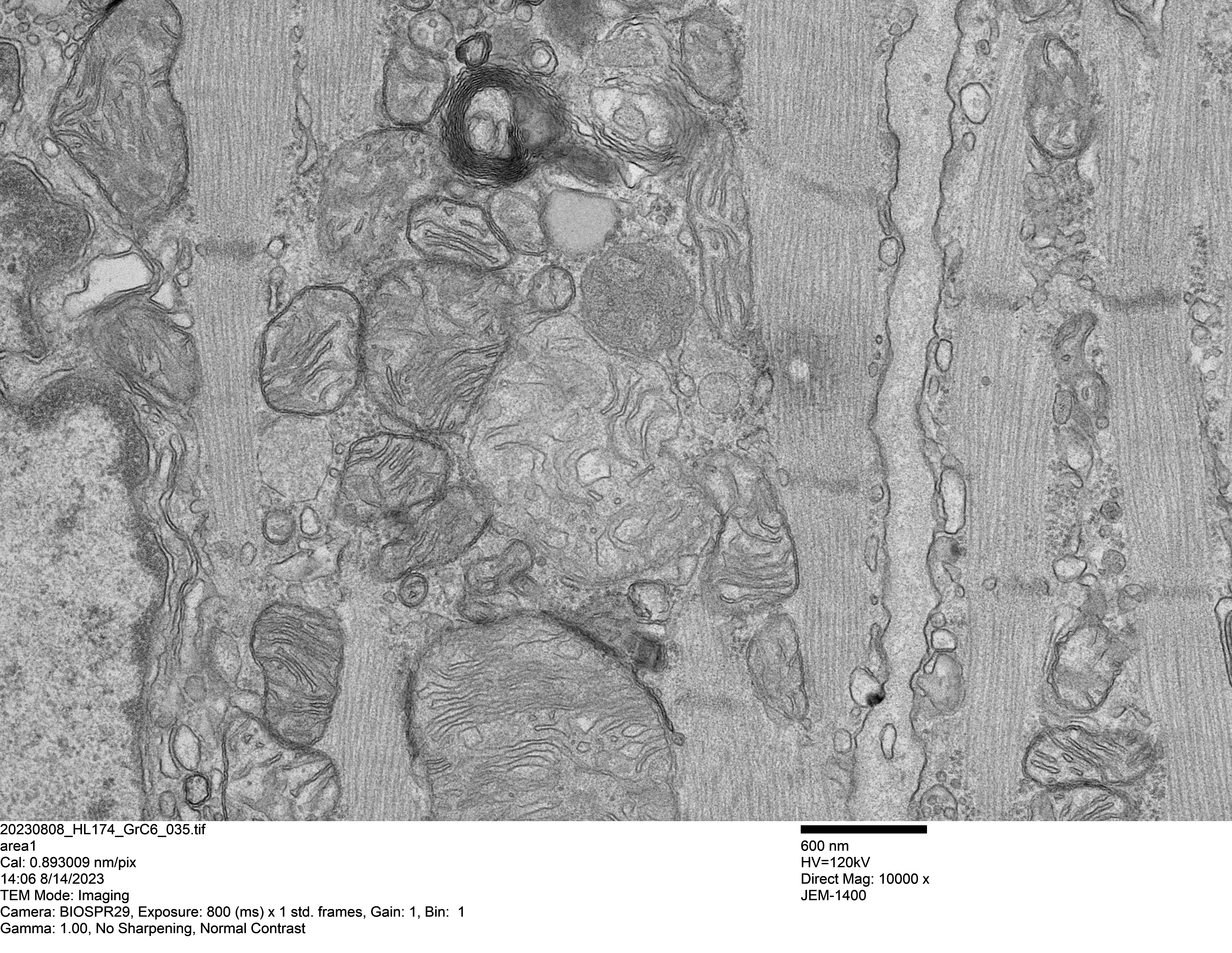

Supplement: Supplementary file 18 — Source data Figure 3E [file 44318_2024_242_MOESM18_ESM.zip › Fig3E/Fig3E_S59L_bottom.tif]

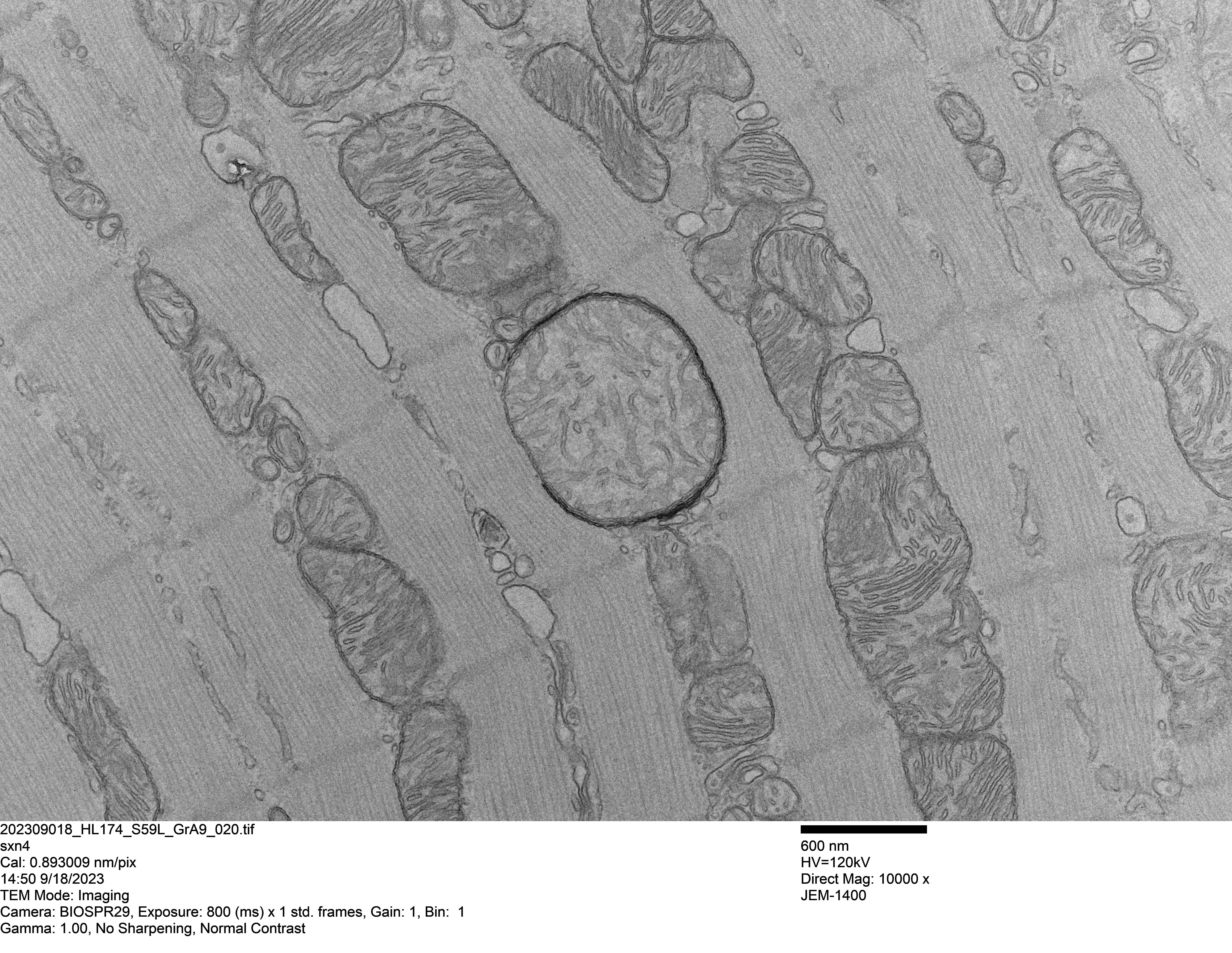

Supplement: Supplementary file 18 — Source data Figure 3E [file 44318_2024_242_MOESM18_ESM.zip › Fig3E/Fig3E_S59L_center.tif]

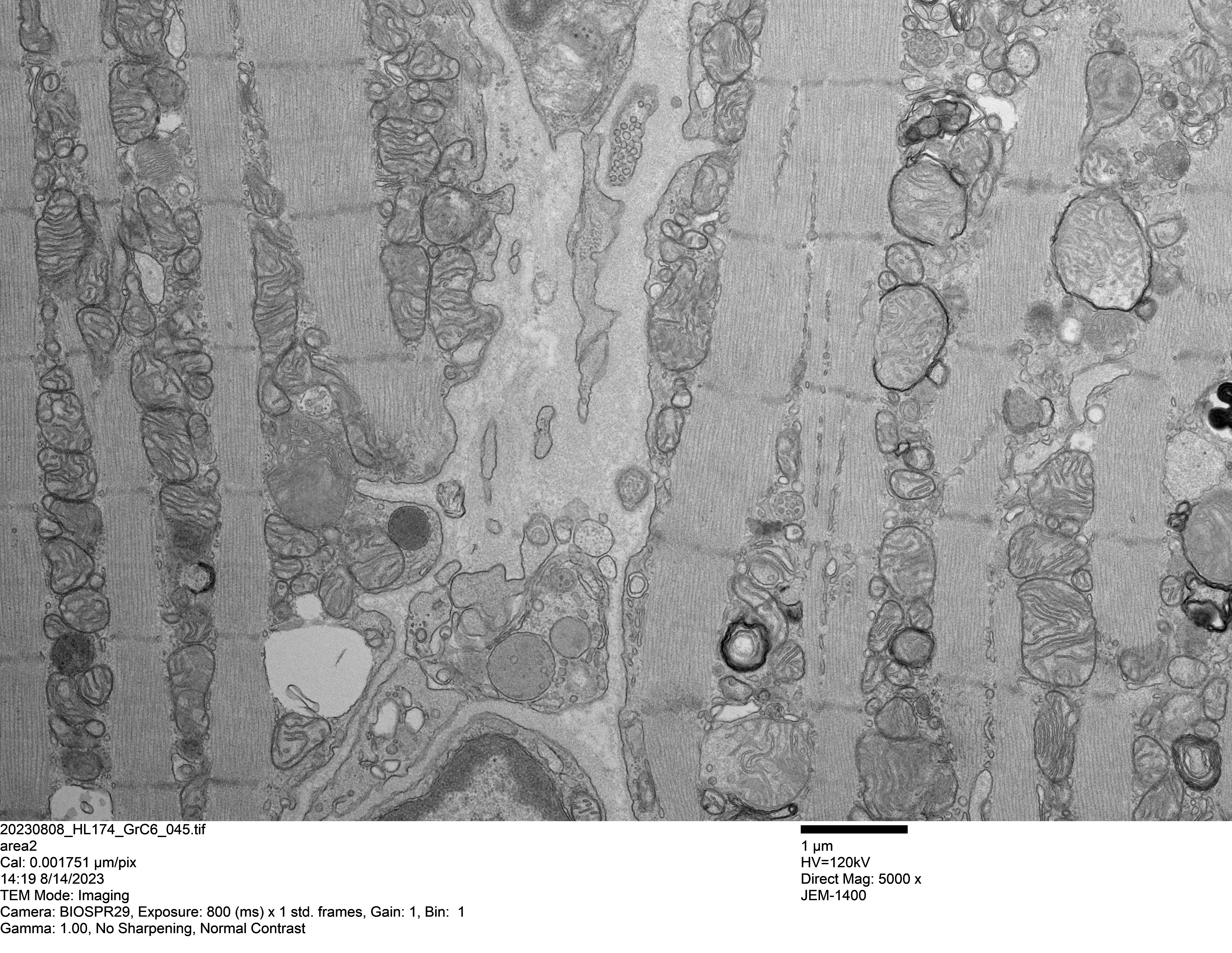

Supplement: Supplementary file 18 — Source data Figure 3E [file 44318_2024_242_MOESM18_ESM.zip › Fig3E/Fig3E_S59L_top.tif]

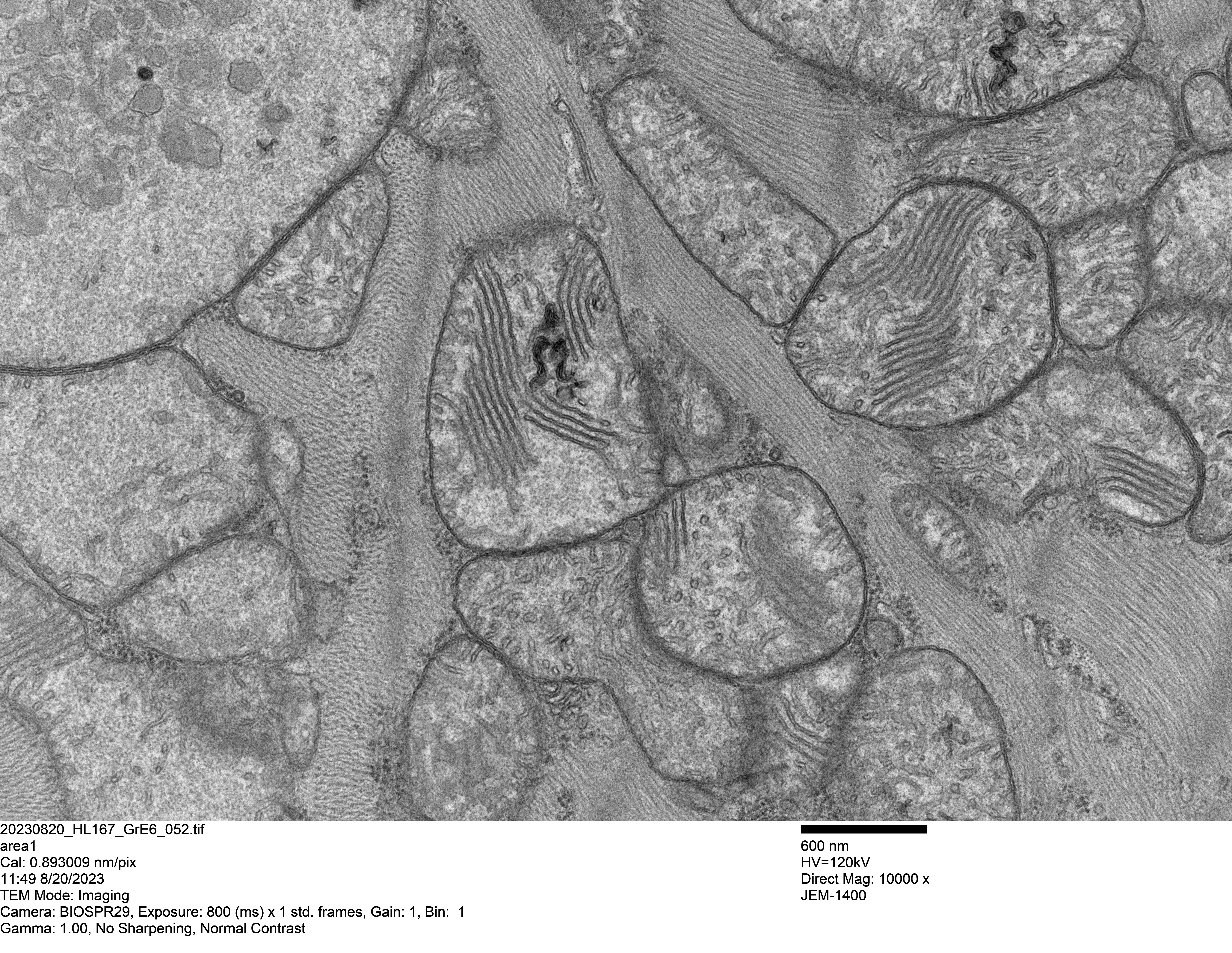

Supplement: Supplementary file 19 — Source data Fig. 3F [file 44318_2024_242_MOESM19_ESM.zip › Fig3F/Fig3F_Tfam_bottom.tif]

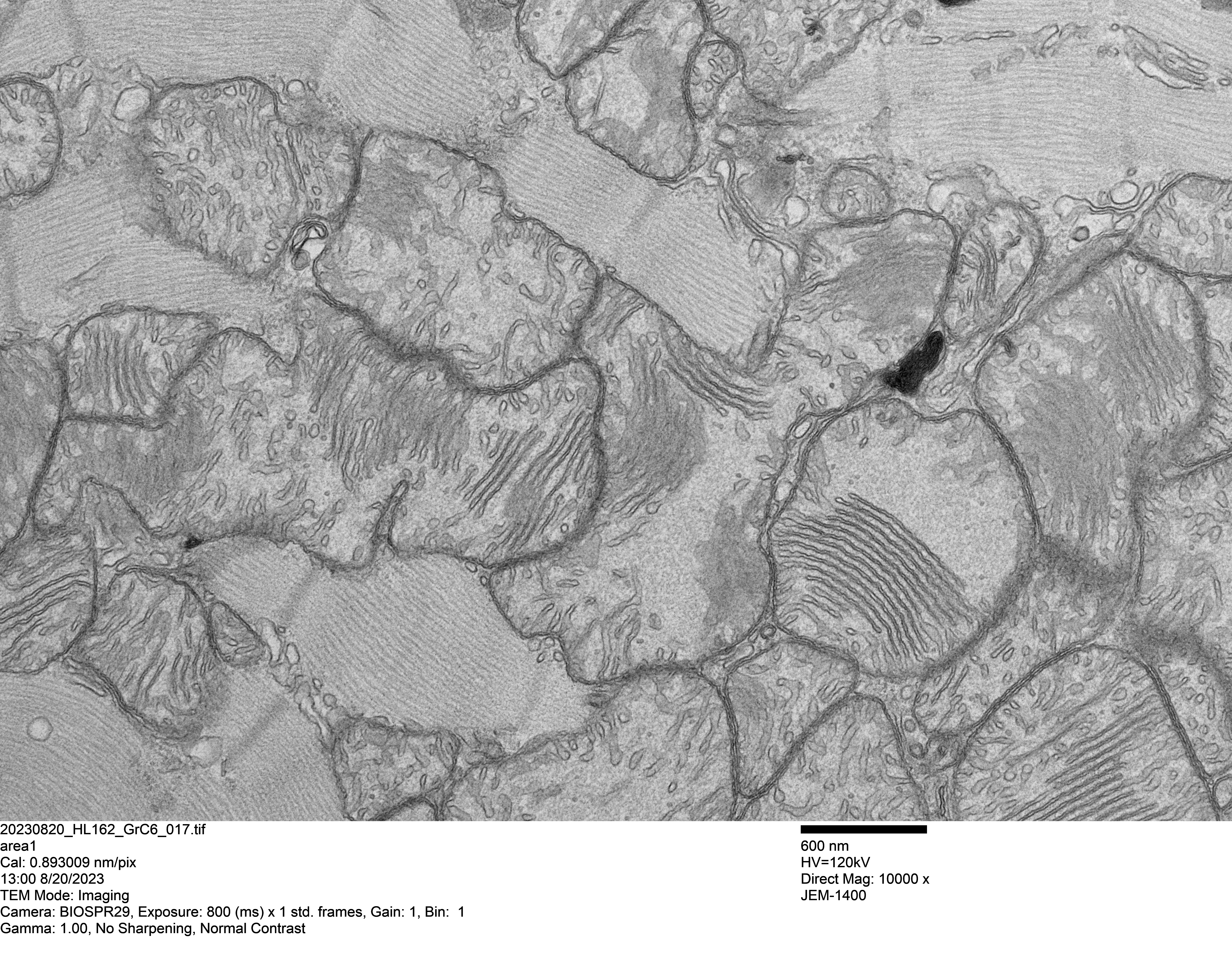

Supplement: Supplementary file 19 — Source data Fig. 3F [file 44318_2024_242_MOESM19_ESM.zip › Fig3F/Fig3F_Tfam_middle.tif]

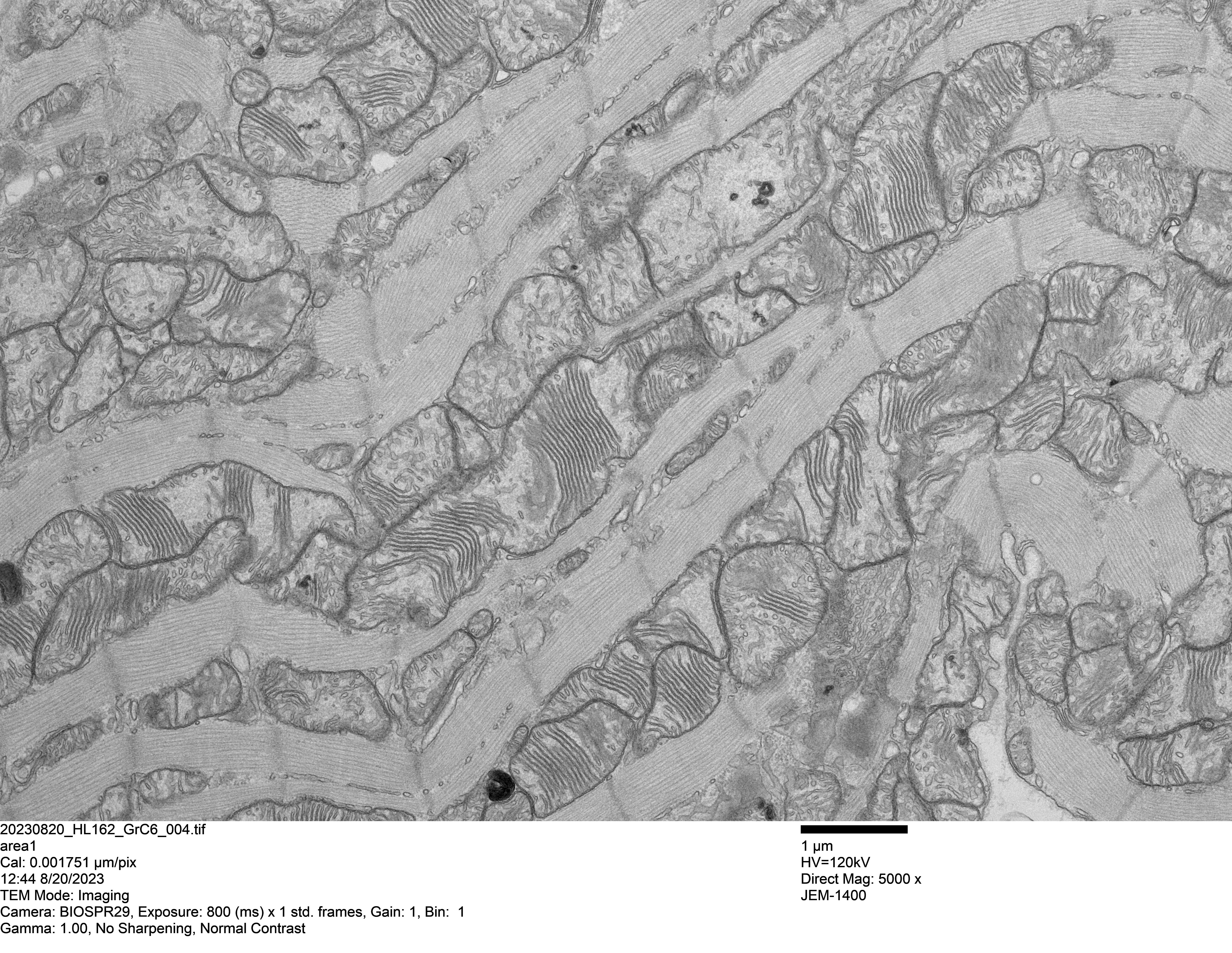

Supplement: Supplementary file 19 — Source data Fig. 3F [file 44318_2024_242_MOESM19_ESM.zip › Fig3F/Fig3F_Tfam_top.tif]

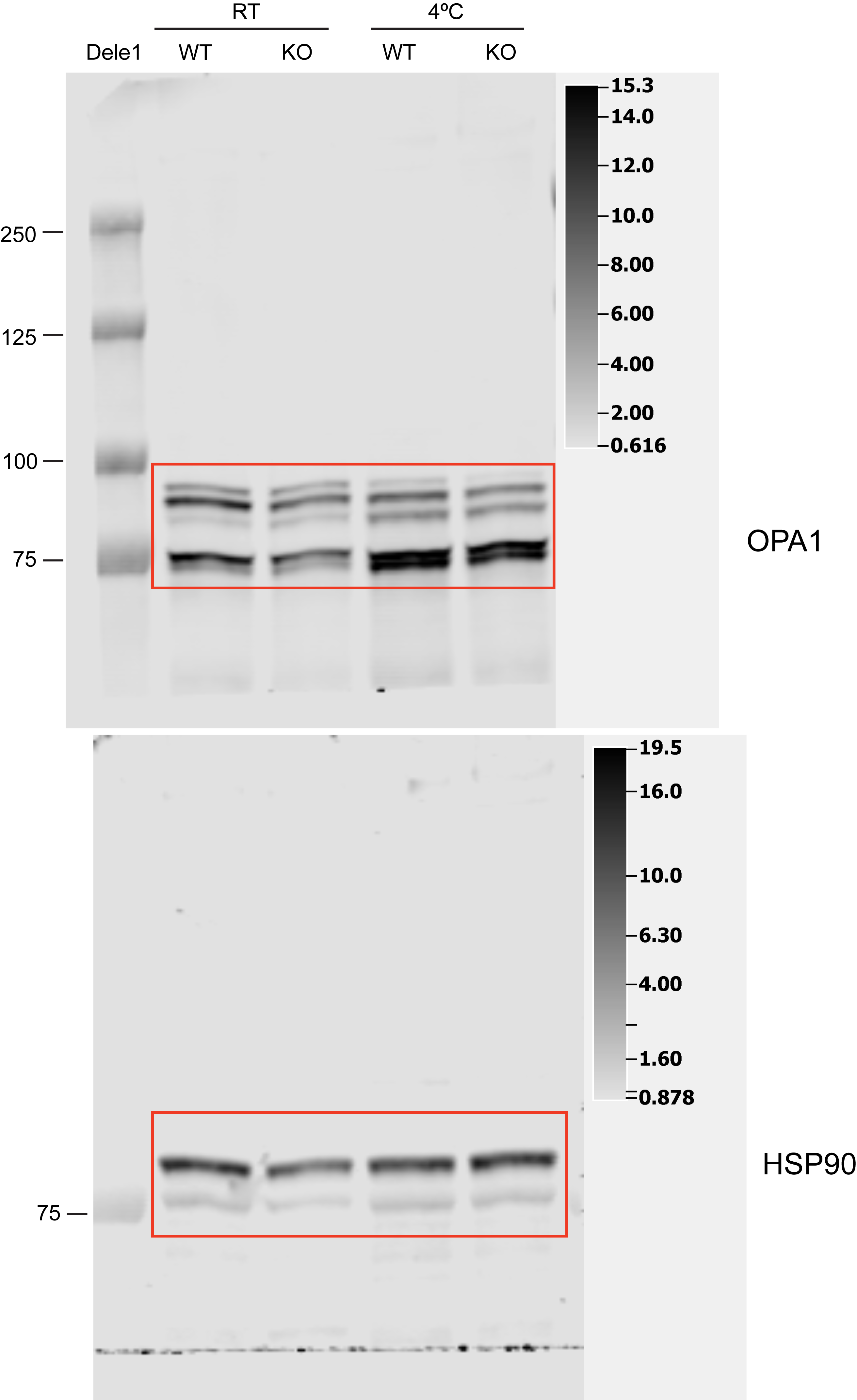

Supplement: Supplementary file 23 — Source data Fig. 6 [file 44318_2024_242_MOESM23_ESM.zip › Figure 6/A/6A.tif]

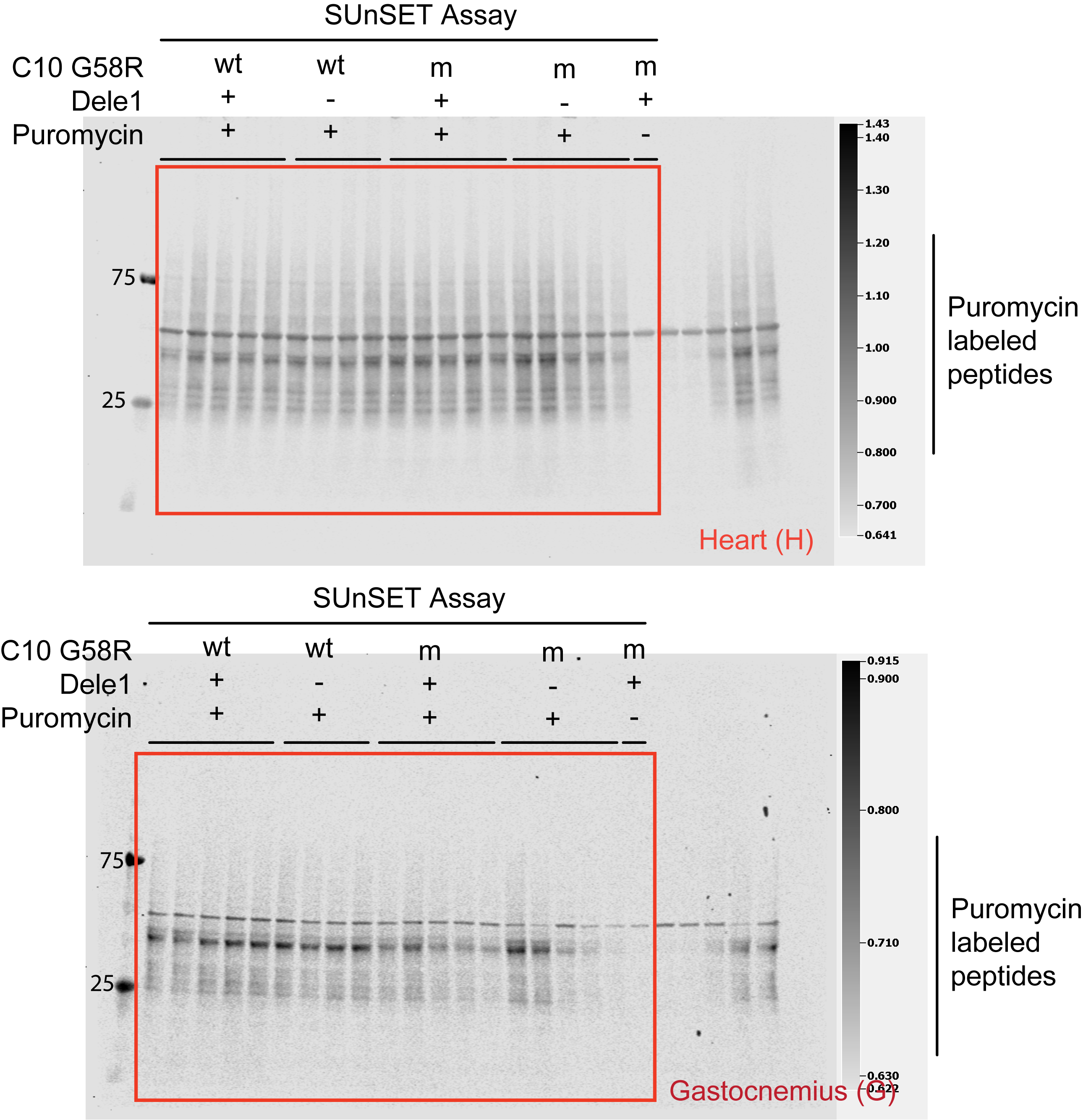

Supplement: Supplementary file 24 — Source data Fig. 7 [file 44318_2024_242_MOESM24_ESM.zip › Figure 7/B/7B.tif]

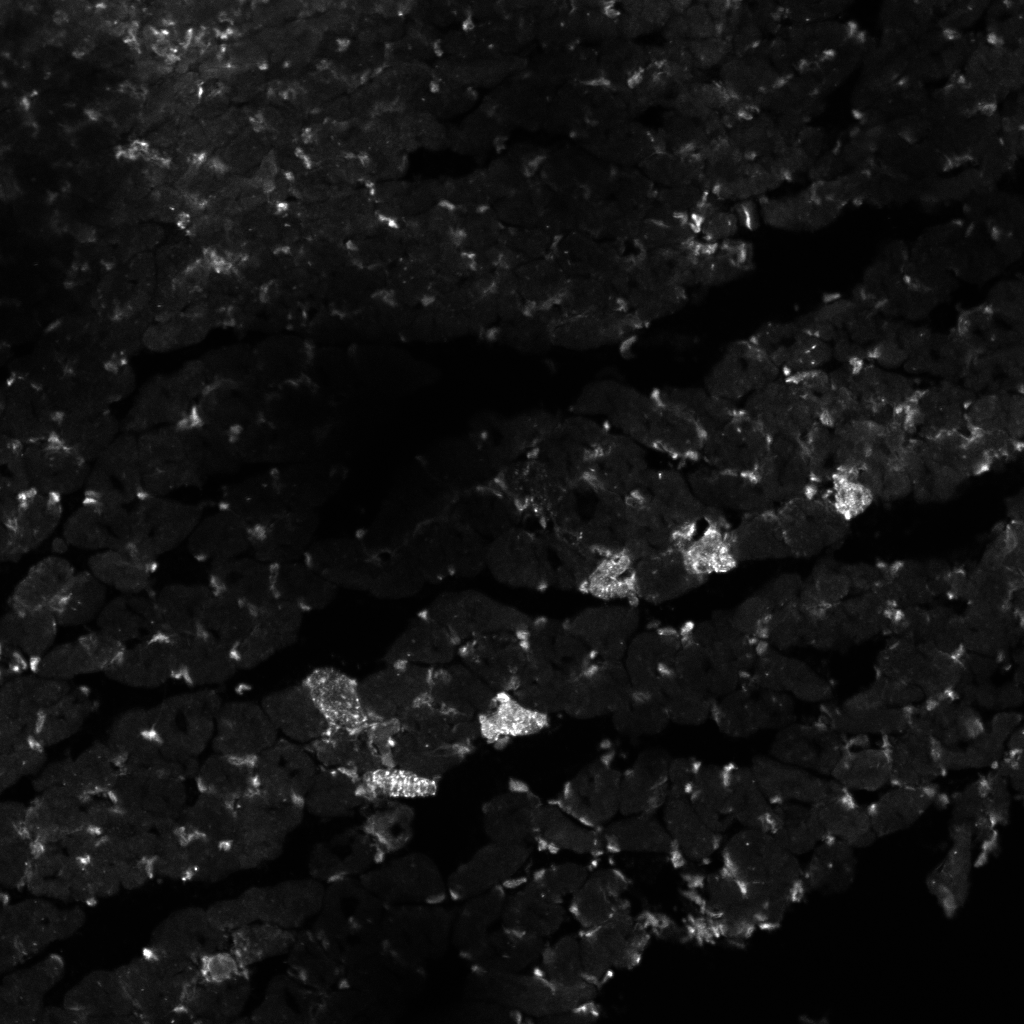

Supplement: Supplementary file 24 — Source data Fig. 7 [file 44318_2024_242_MOESM24_ESM.zip › Figure 7/D/Fig7D_C10 G58R; Dele1KO_FK2.tif]

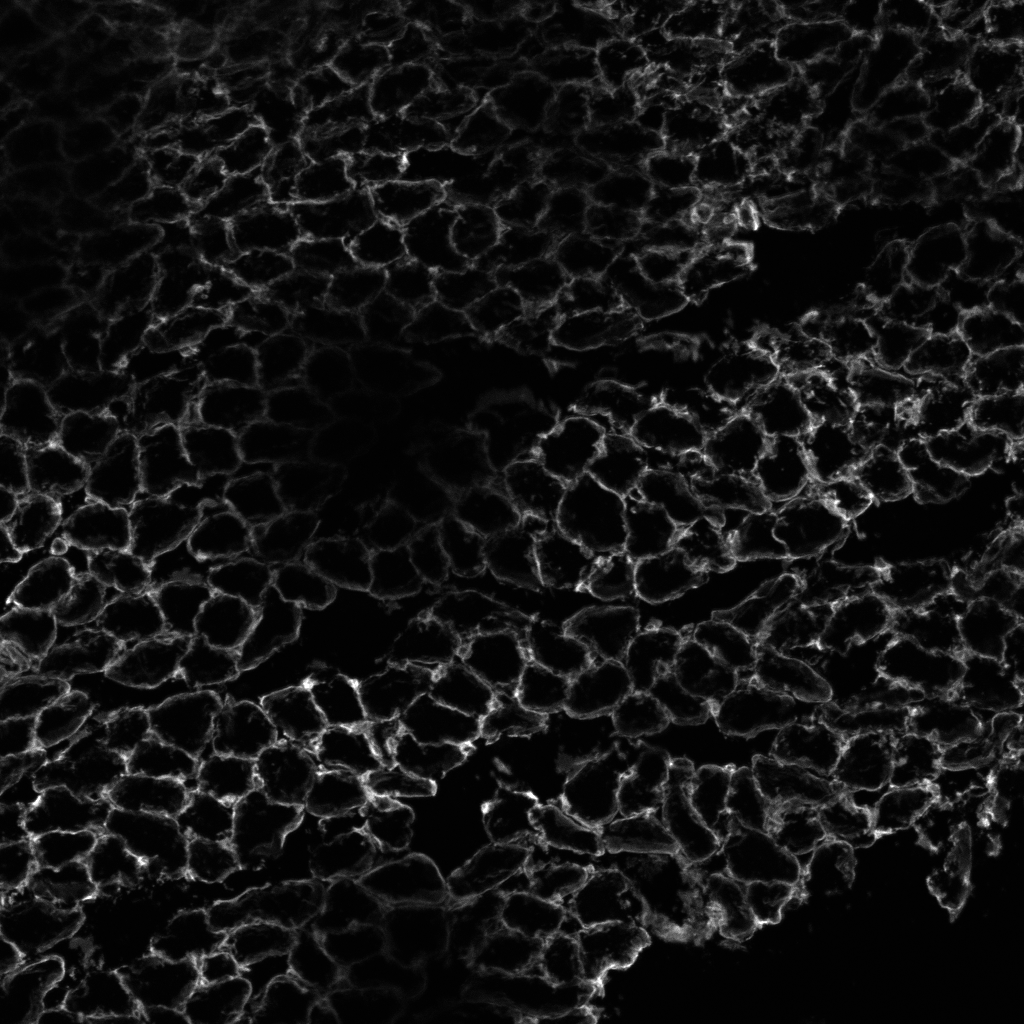

Supplement: Supplementary file 24 — Source data Fig. 7 [file 44318_2024_242_MOESM24_ESM.zip › Figure 7/D/Fig7D_C10 G58R; Dele1KO_Laminin.tif]

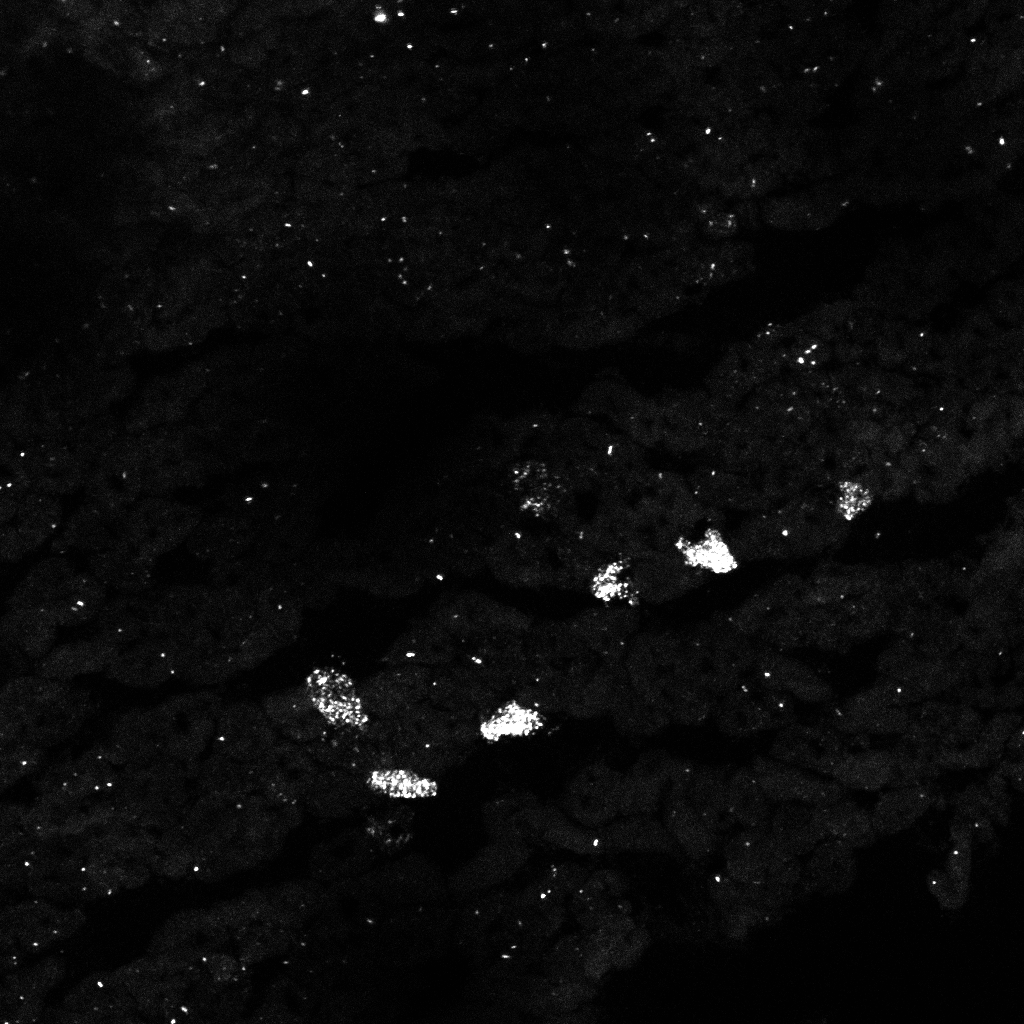

Supplement: Supplementary file 24 — Source data Fig. 7 [file 44318_2024_242_MOESM24_ESM.zip › Figure 7/D/Fig7D_C10 G58R; Dele1KO_P62.tif]

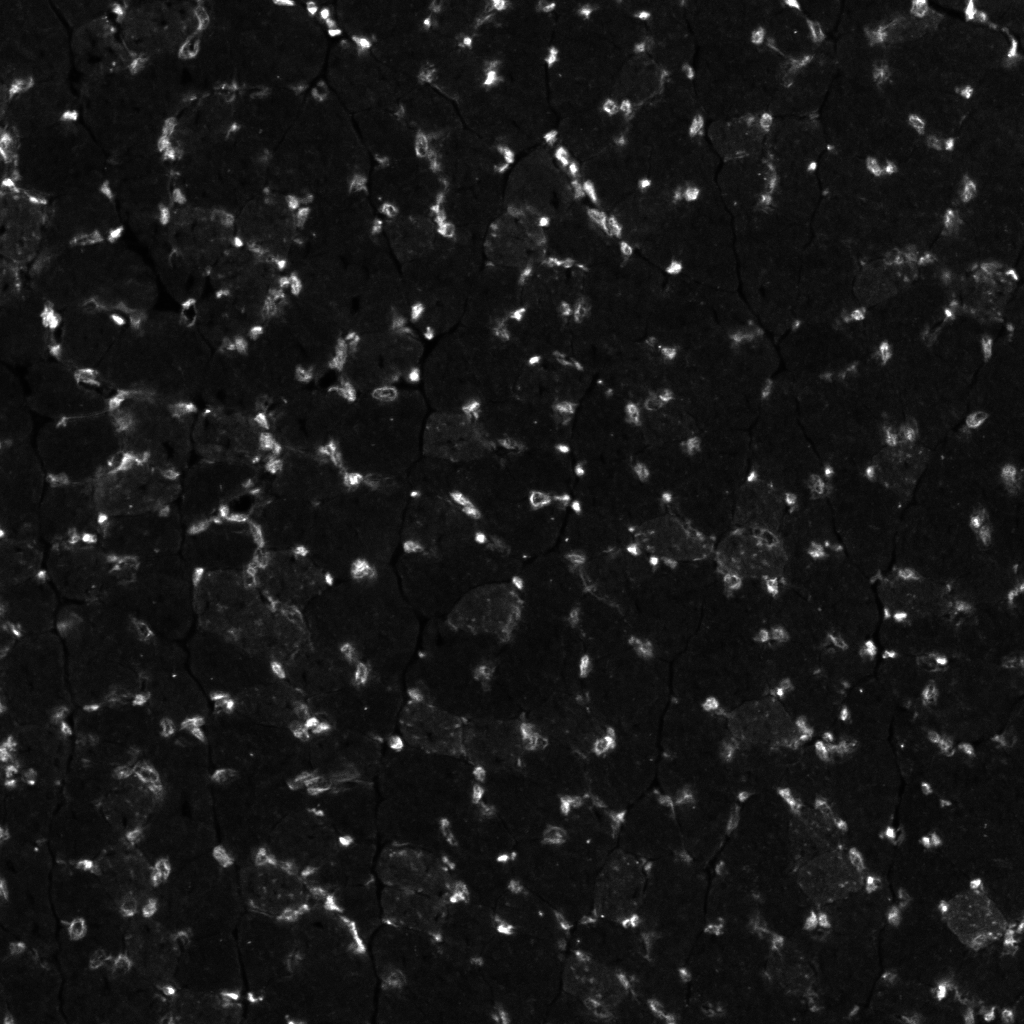

Supplement: Supplementary file 24 — Source data Fig. 7 [file 44318_2024_242_MOESM24_ESM.zip › Figure 7/D/Fig7D_C10 G58R; Dele1plus_FK2.tif]

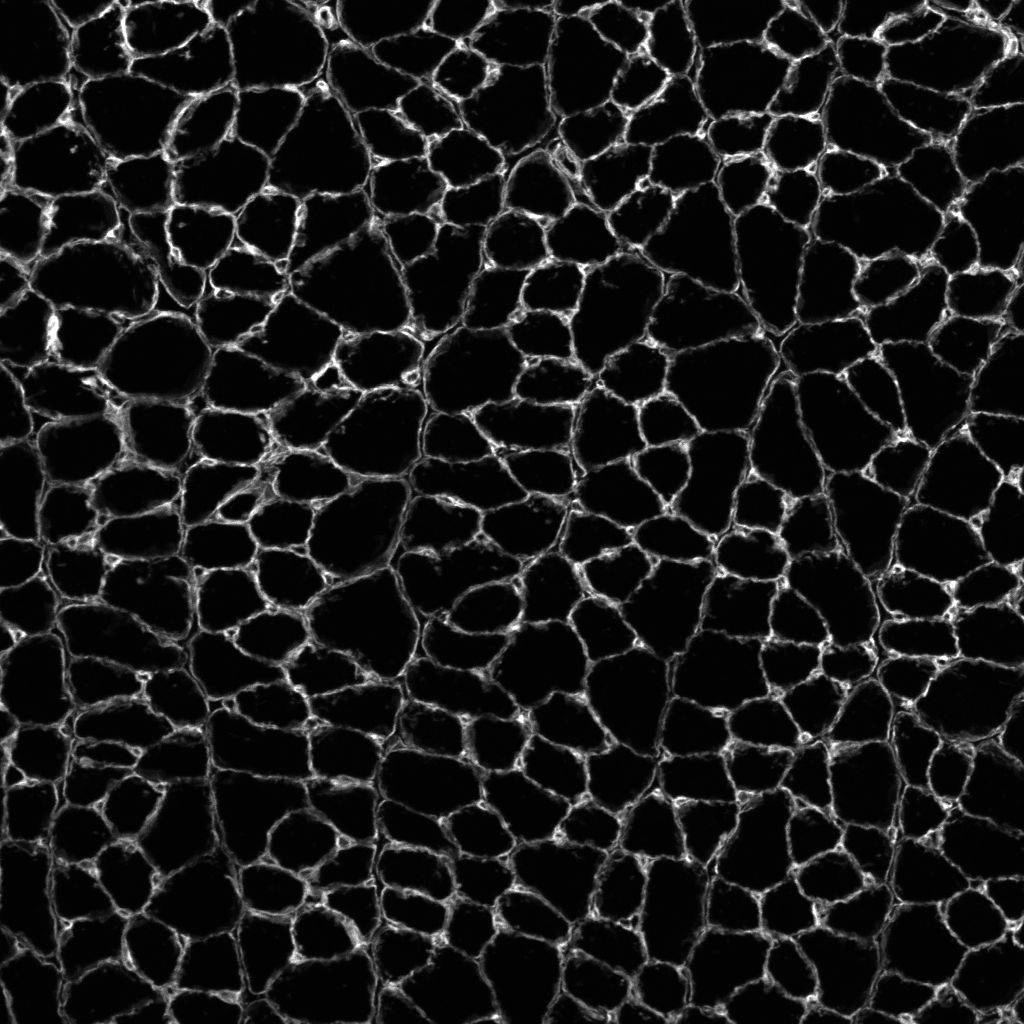

Supplement: Supplementary file 24 — Source data Fig. 7 [file 44318_2024_242_MOESM24_ESM.zip › Figure 7/D/Fig7D_C10 G58R; Dele1plus_Laminin.tif]

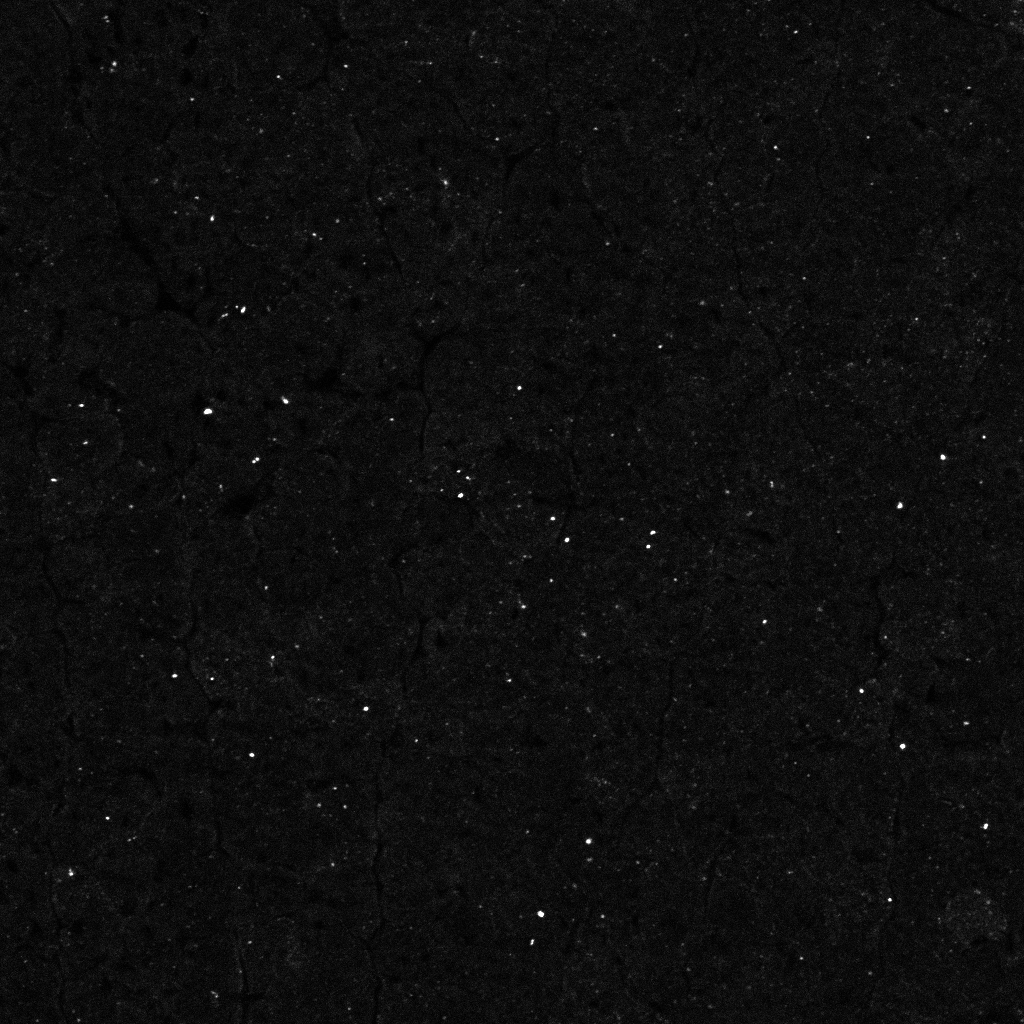

Supplement: Supplementary file 24 — Source data Fig. 7 [file 44318_2024_242_MOESM24_ESM.zip › Figure 7/D/Fig7D_C10 G58R; Dele1plus_P62.tif]

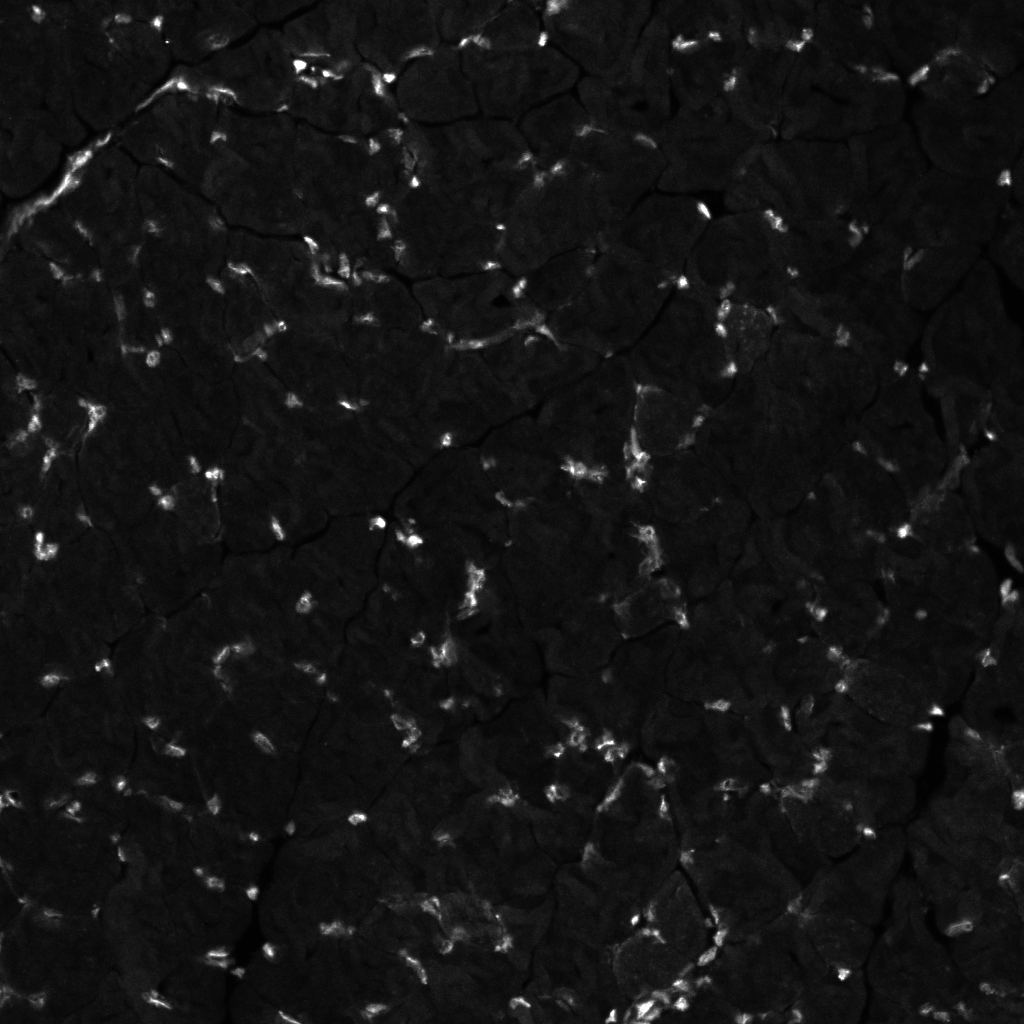

Supplement: Supplementary file 24 — Source data Fig. 7 [file 44318_2024_242_MOESM24_ESM.zip › Figure 7/D/Fig7D_C10 WT; Dele1KO_FK2.tif]

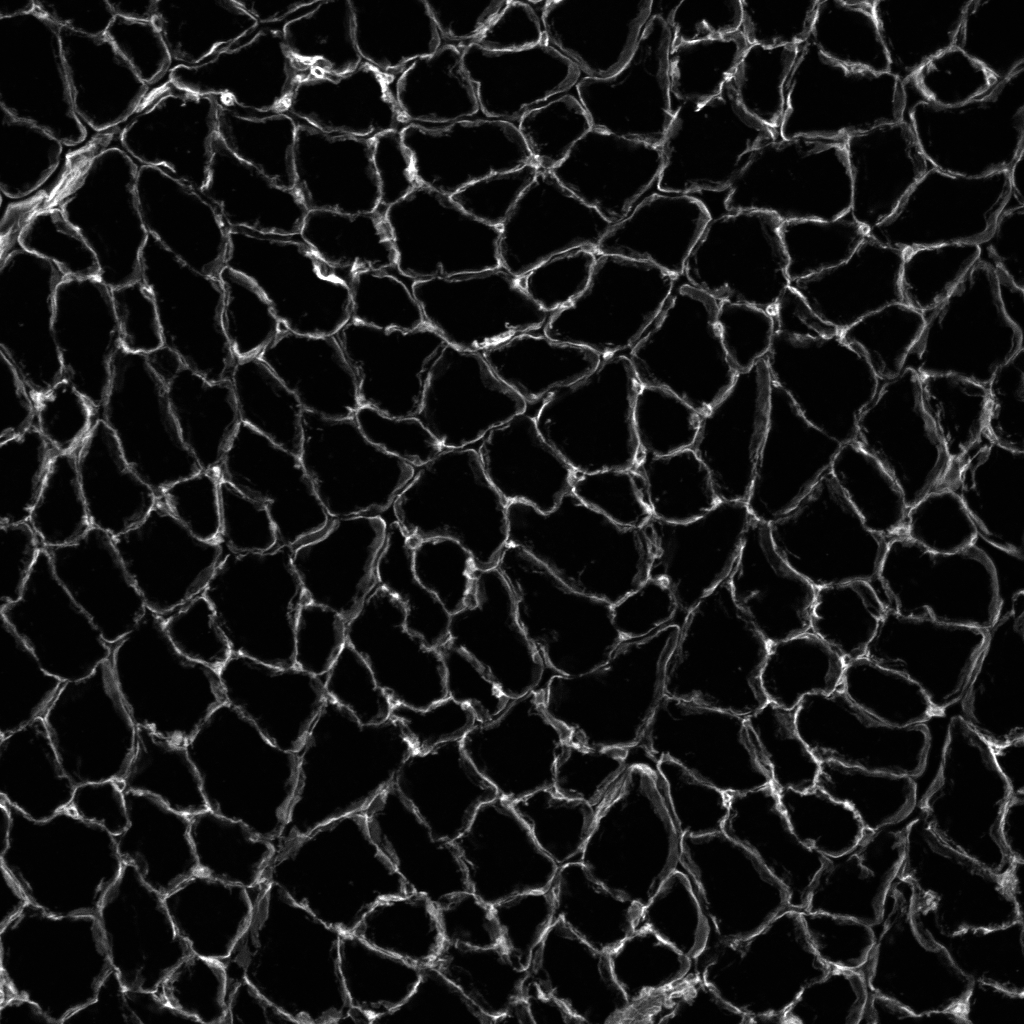

Supplement: Supplementary file 24 — Source data Fig. 7 [file 44318_2024_242_MOESM24_ESM.zip › Figure 7/D/Fig7D_C10 WT; Dele1KO_Laminin.tif]

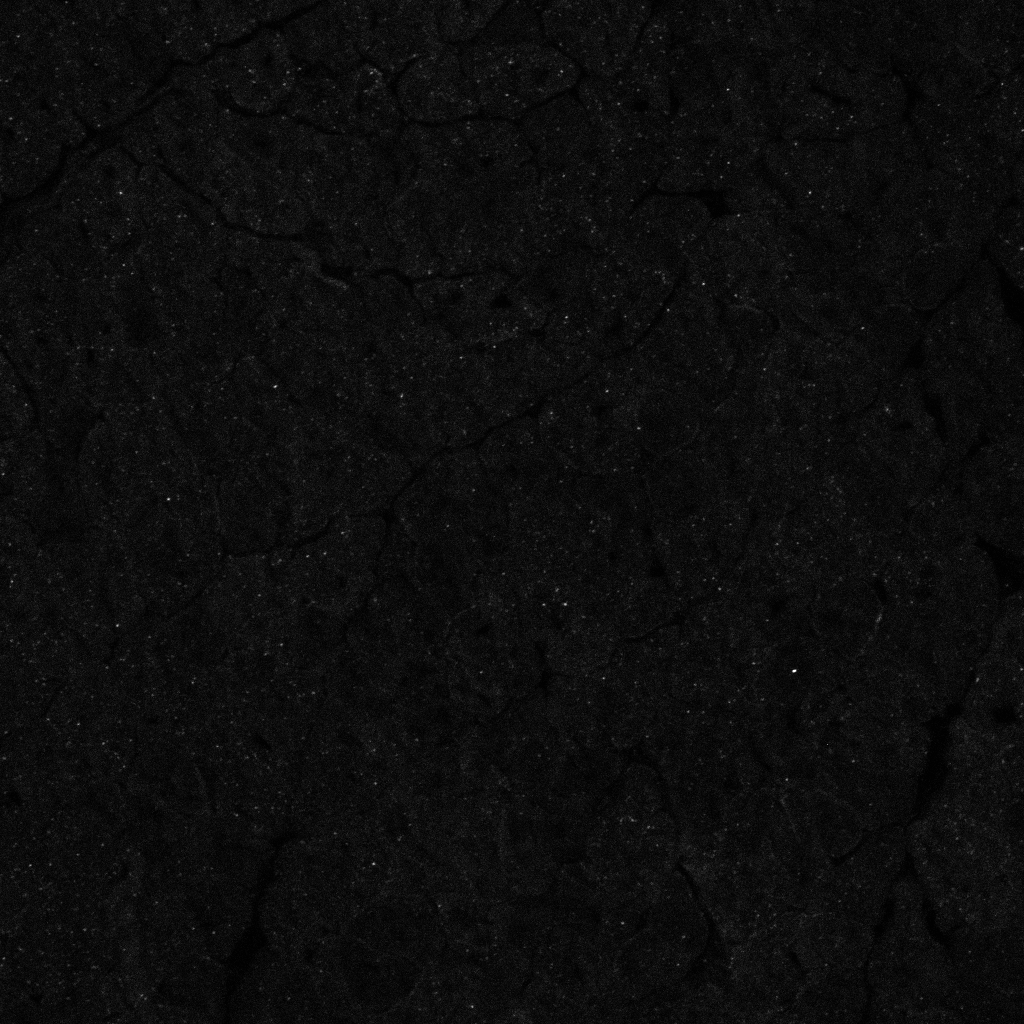

Supplement: Supplementary file 24 — Source data Fig. 7 [file 44318_2024_242_MOESM24_ESM.zip › Figure 7/D/Fig7D_C10 WT; Dele1KO_P62.tif]

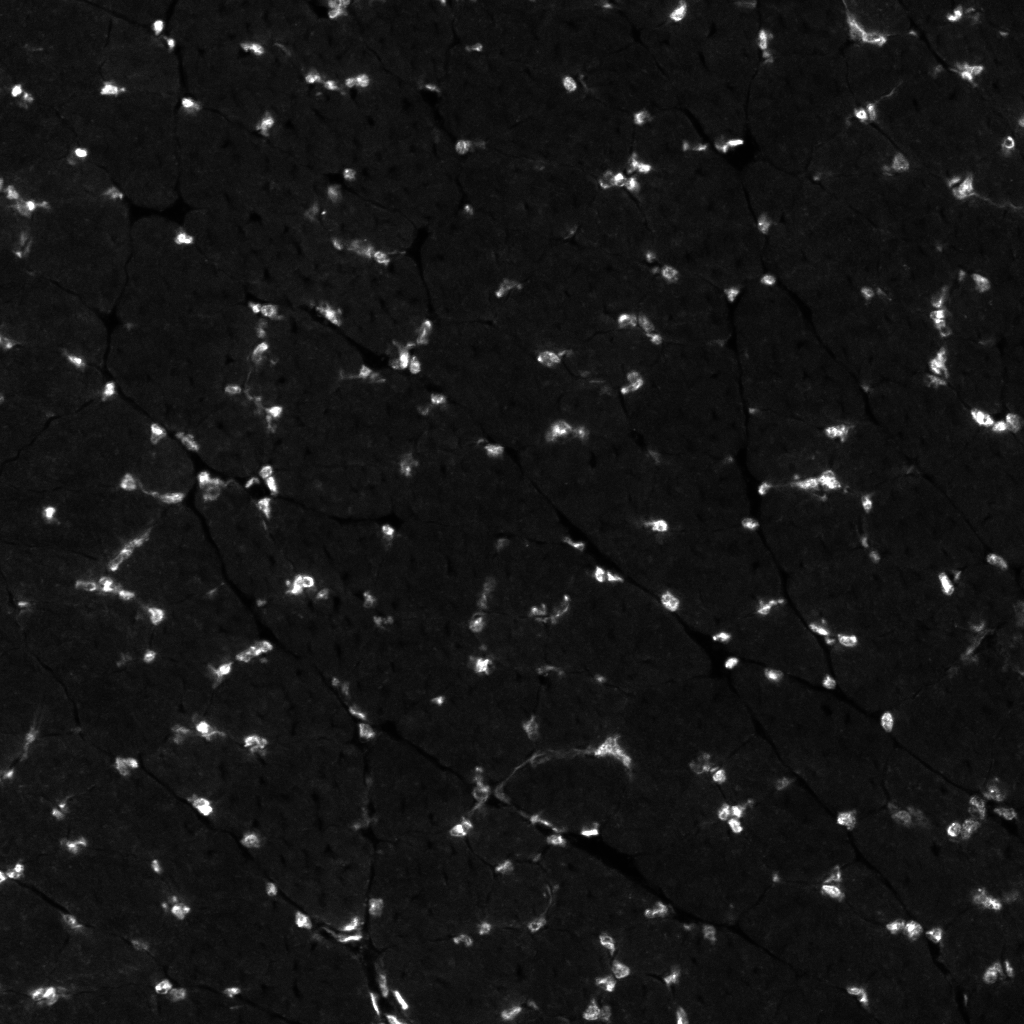

Supplement: Supplementary file 24 — Source data Fig. 7 [file 44318_2024_242_MOESM24_ESM.zip › Figure 7/D/Fig7D_C10 WT; Dele1plus_FK2.tif]

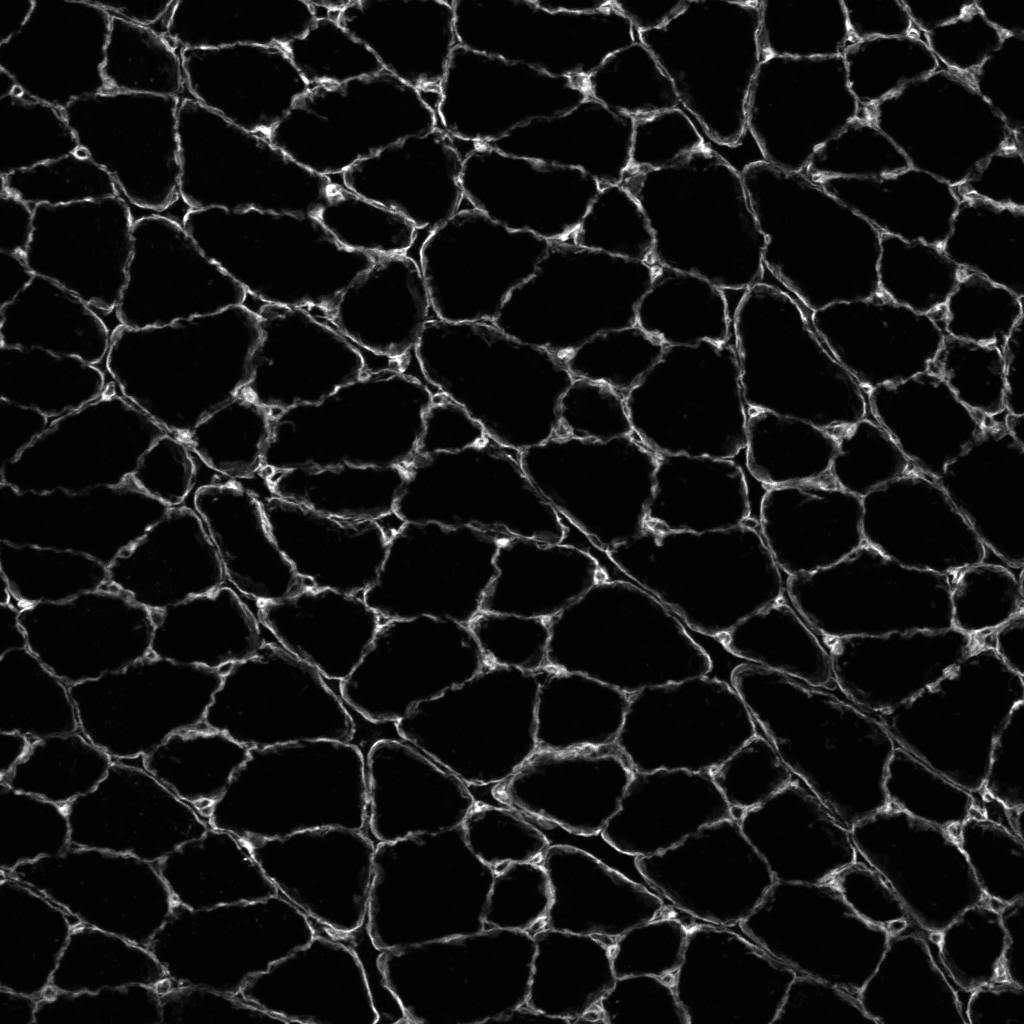

Supplement: Supplementary file 24 — Source data Fig. 7 [file 44318_2024_242_MOESM24_ESM.zip › Figure 7/D/Fig7D_C10 WT; Dele1plus_Laminin.tif]

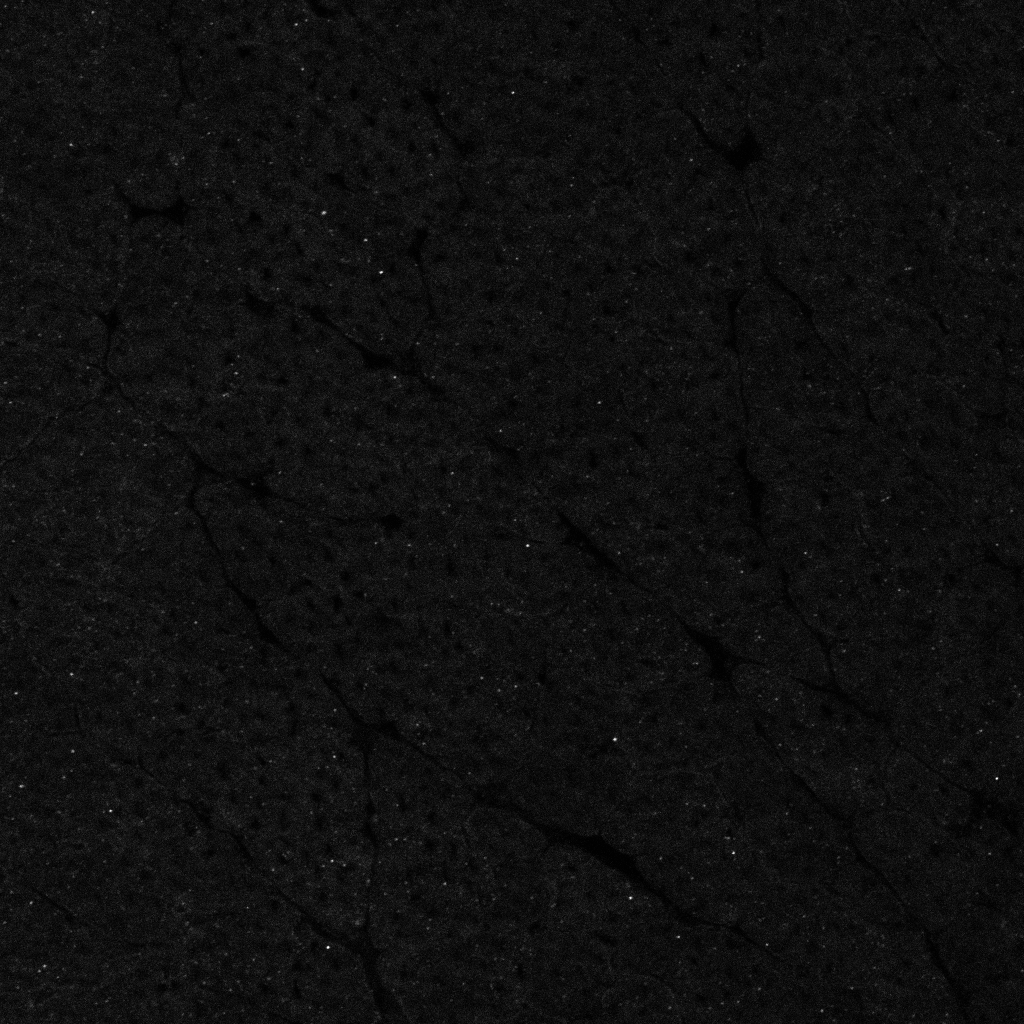

Supplement: Supplementary file 24 — Source data Fig. 7 [file 44318_2024_242_MOESM24_ESM.zip › Figure 7/D/Fig7D_C10 WT; Dele1plus_P62.tif]

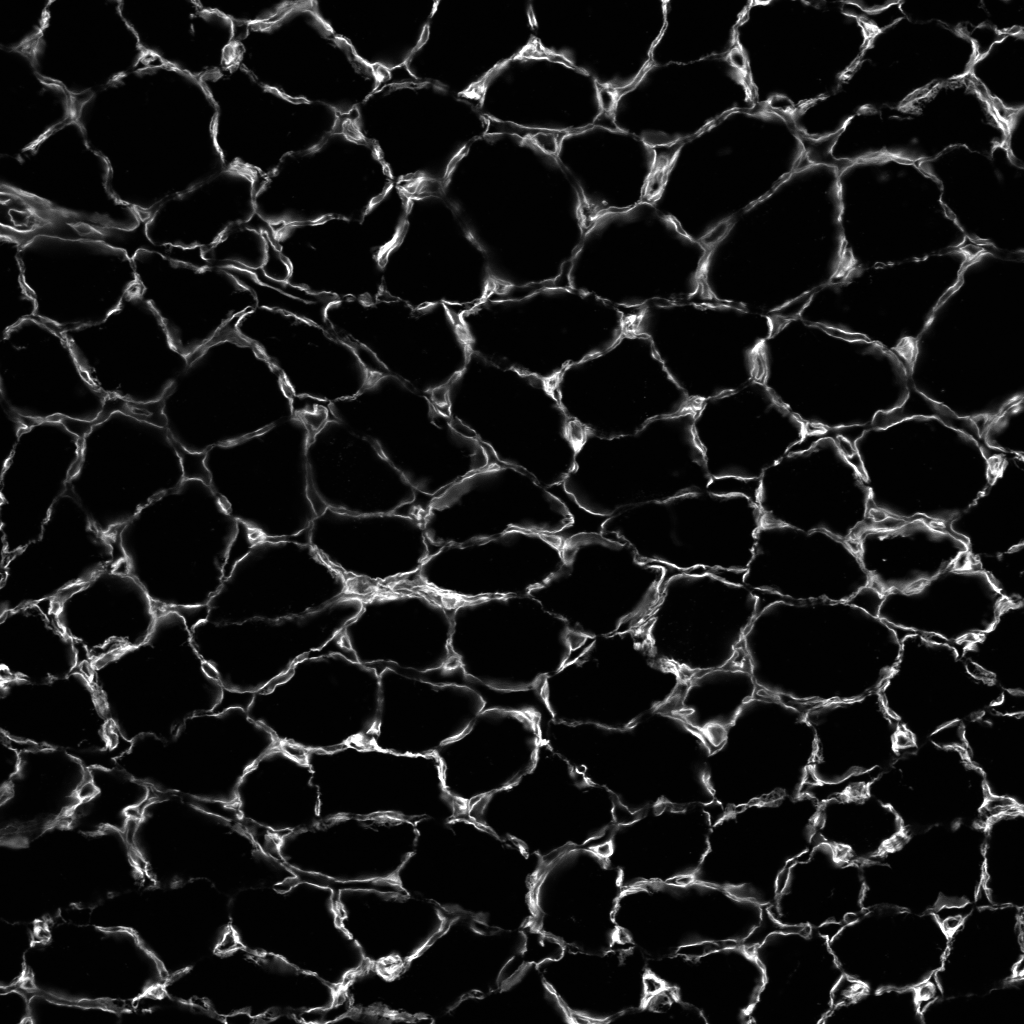

Supplement: Supplementary file 24 — Source data Fig. 7 [file 44318_2024_242_MOESM24_ESM.zip › Figure 7/F/Fig7F_G58R_Dele1KO_Laminin_60x.tif]

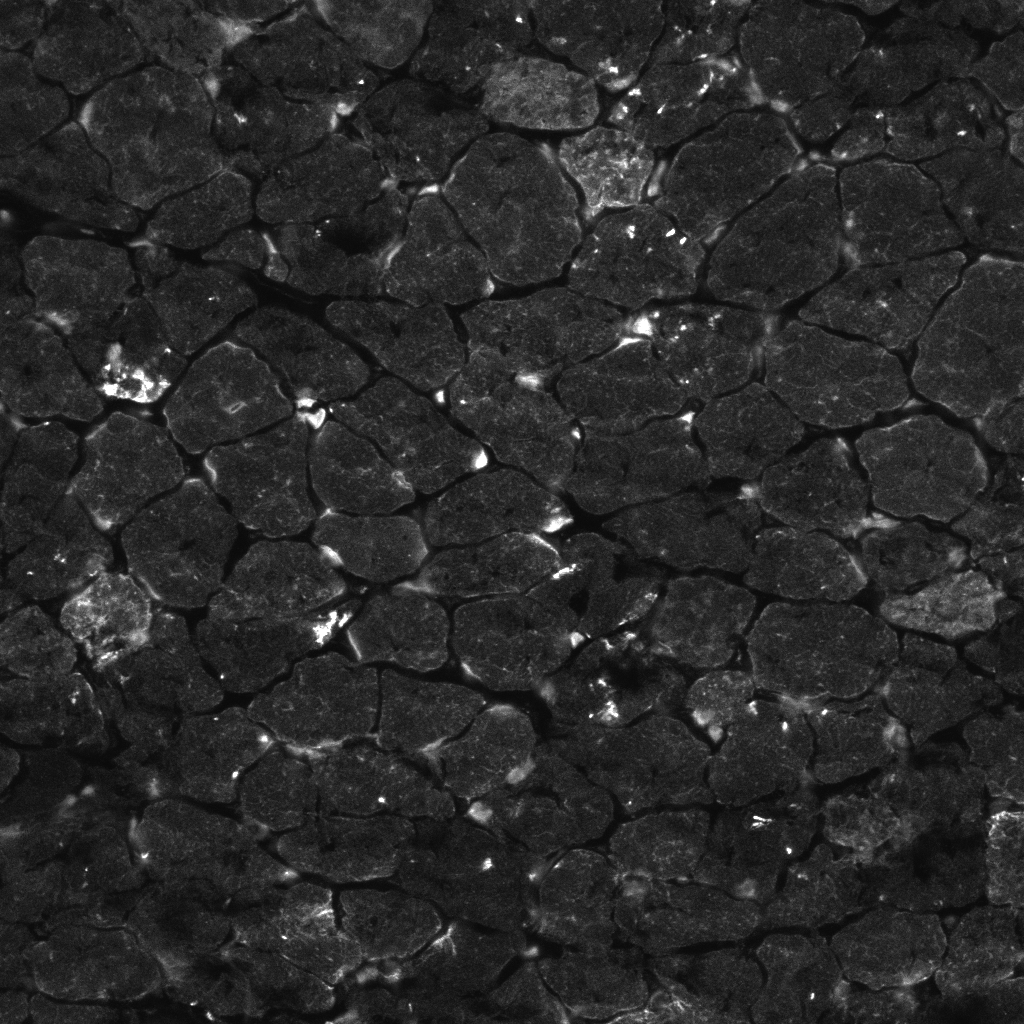

Supplement: Supplementary file 24 — Source data Fig. 7 [file 44318_2024_242_MOESM24_ESM.zip › Figure 7/F/Fig7F_G58R_Dele1KO_Puro_60x.tif]

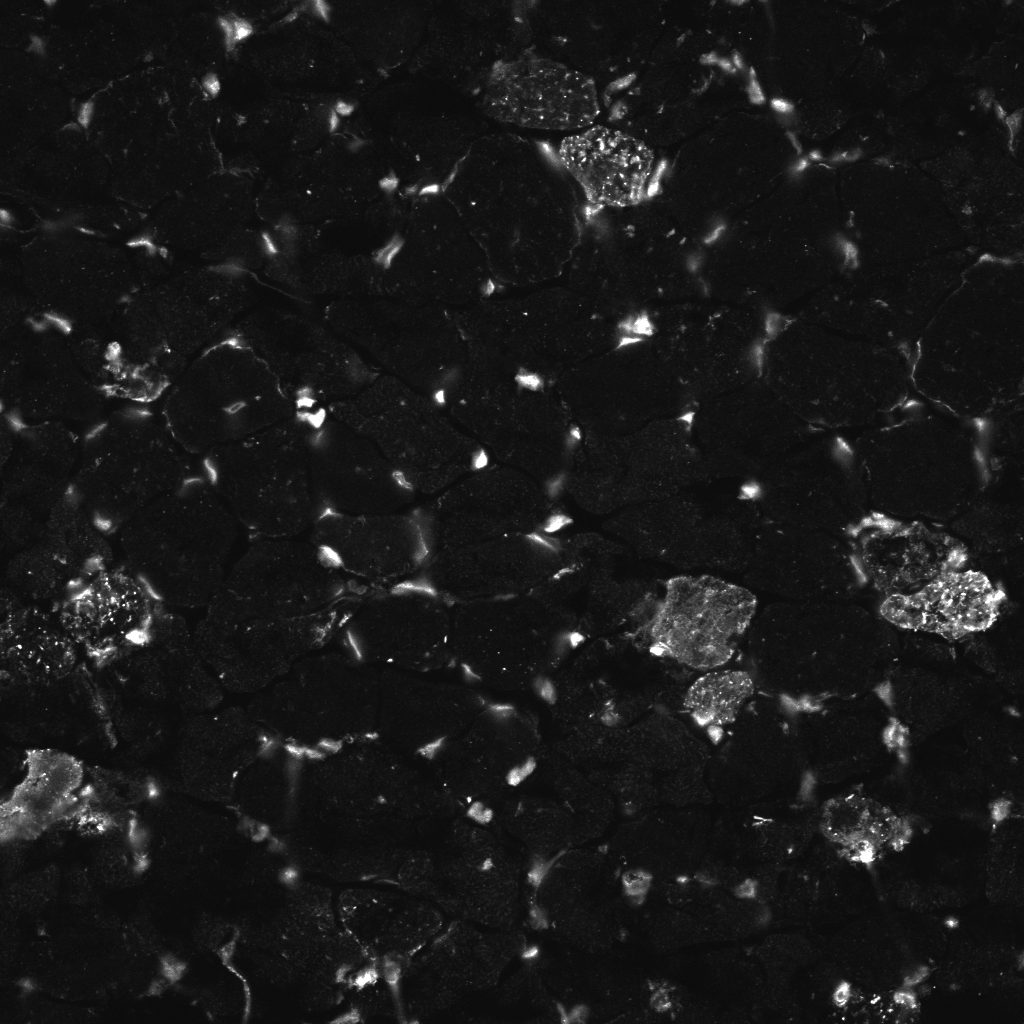

Supplement: Supplementary file 24 — Source data Fig. 7 [file 44318_2024_242_MOESM24_ESM.zip › Figure 7/F/Fig7F_G58R_Dele1KO_Ubiquitin_60x.tif]

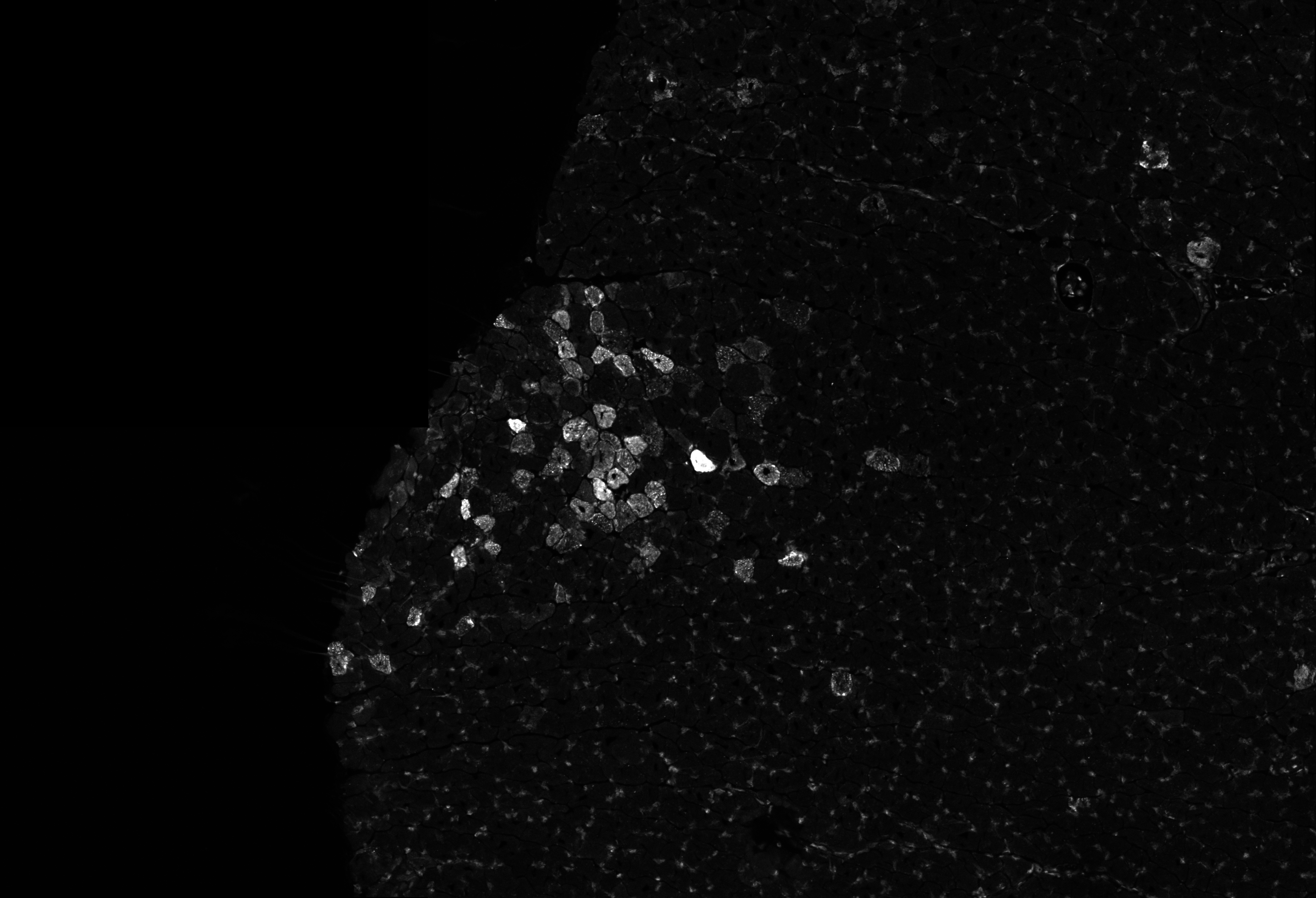

Supplement: Supplementary file 24 — Source data Fig. 7 [file 44318_2024_242_MOESM24_ESM.zip › Figure 7/I/Fig7I_G58R_Dele1KO_left_FK2_20x.tif.tif]

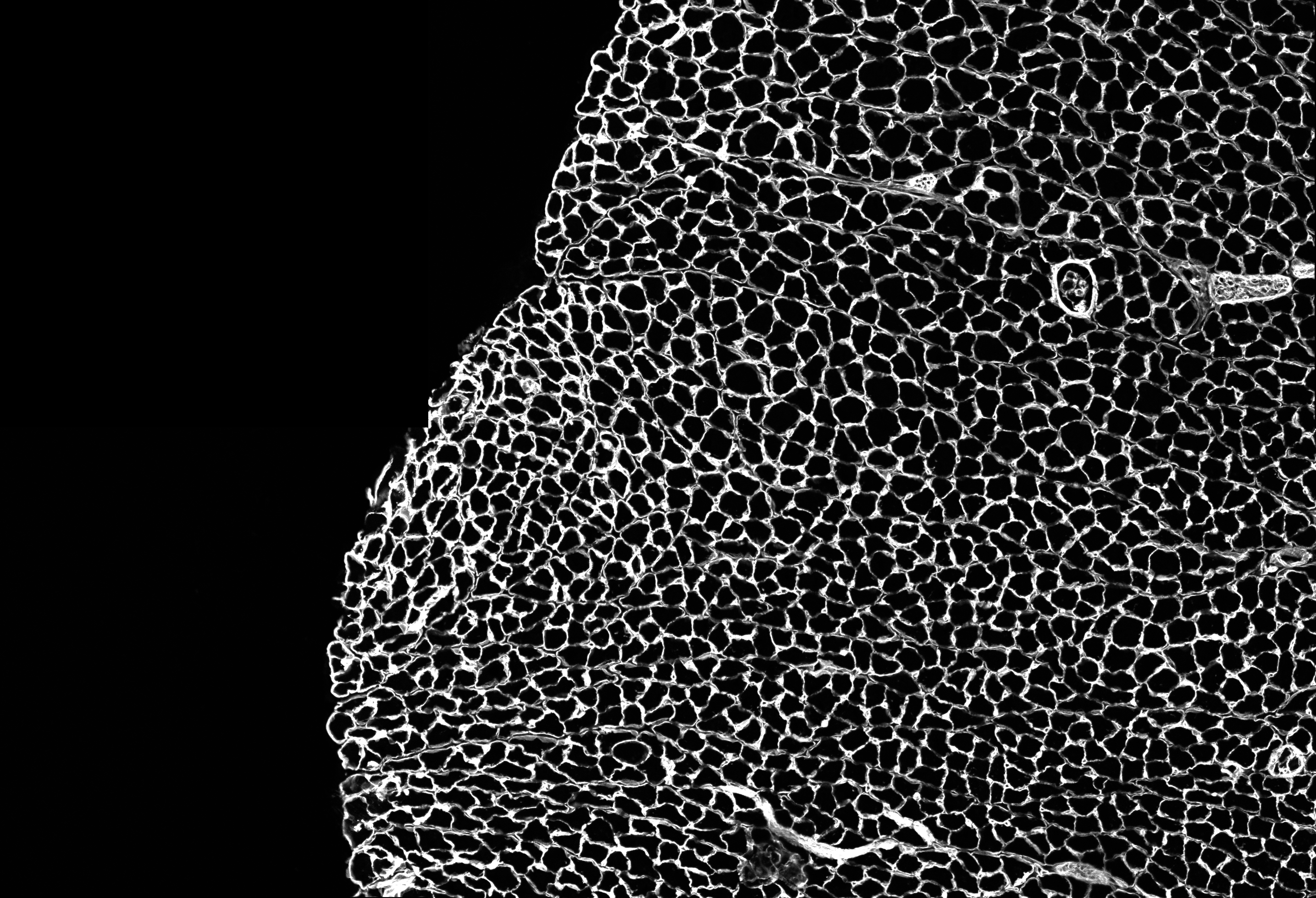

Supplement: Supplementary file 24 — Source data Fig. 7 [file 44318_2024_242_MOESM24_ESM.zip › Figure 7/I/Fig7I_G58R_Dele1KO_left_Laminin_20x.tif.tif]

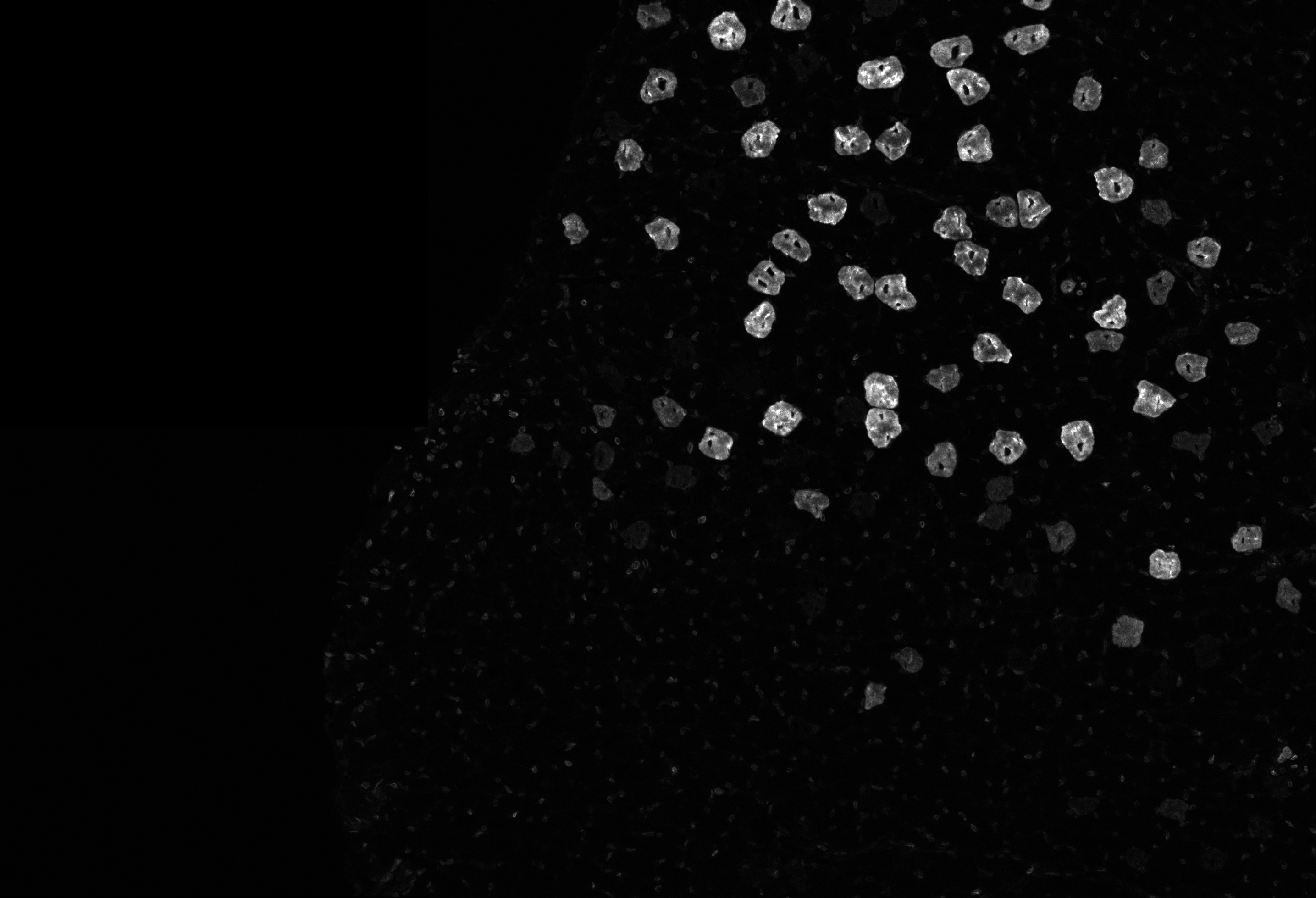

Supplement: Supplementary file 24 — Source data Fig. 7 [file 44318_2024_242_MOESM24_ESM.zip › Figure 7/I/Fig7I_G58R_Dele1KO_left_type1_20x.tif.tif]

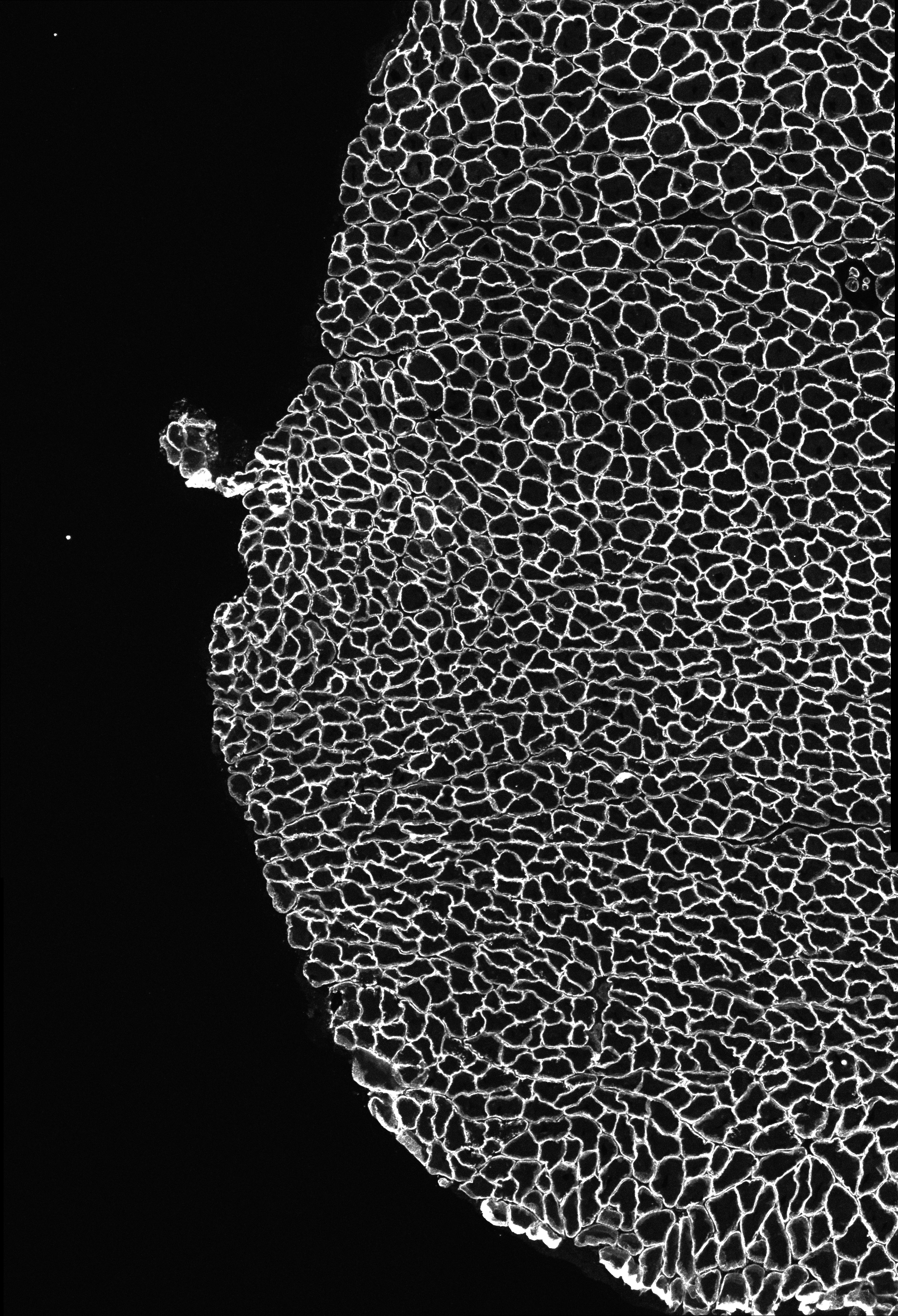

Supplement: Supplementary file 24 — Source data Fig. 7 [file 44318_2024_242_MOESM24_ESM.zip › Figure 7/I/Fig7I_G58R_Dele1KO_middle_dystrophin_20x.tif.tif]

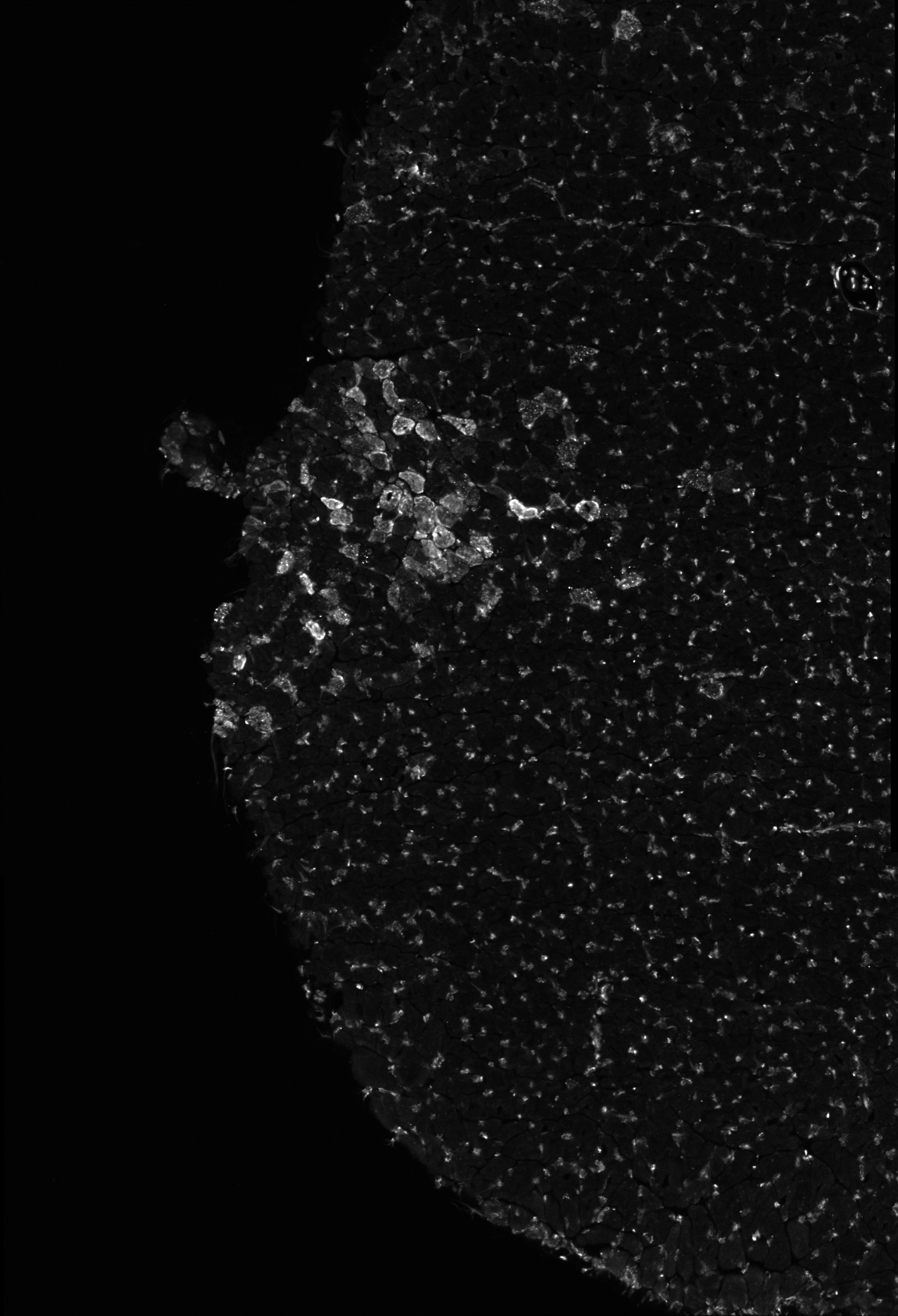

Supplement: Supplementary file 24 — Source data Fig. 7 [file 44318_2024_242_MOESM24_ESM.zip › Figure 7/I/Fig7I_G58R_Dele1KO_middle_FK2_20x.tif.tif]

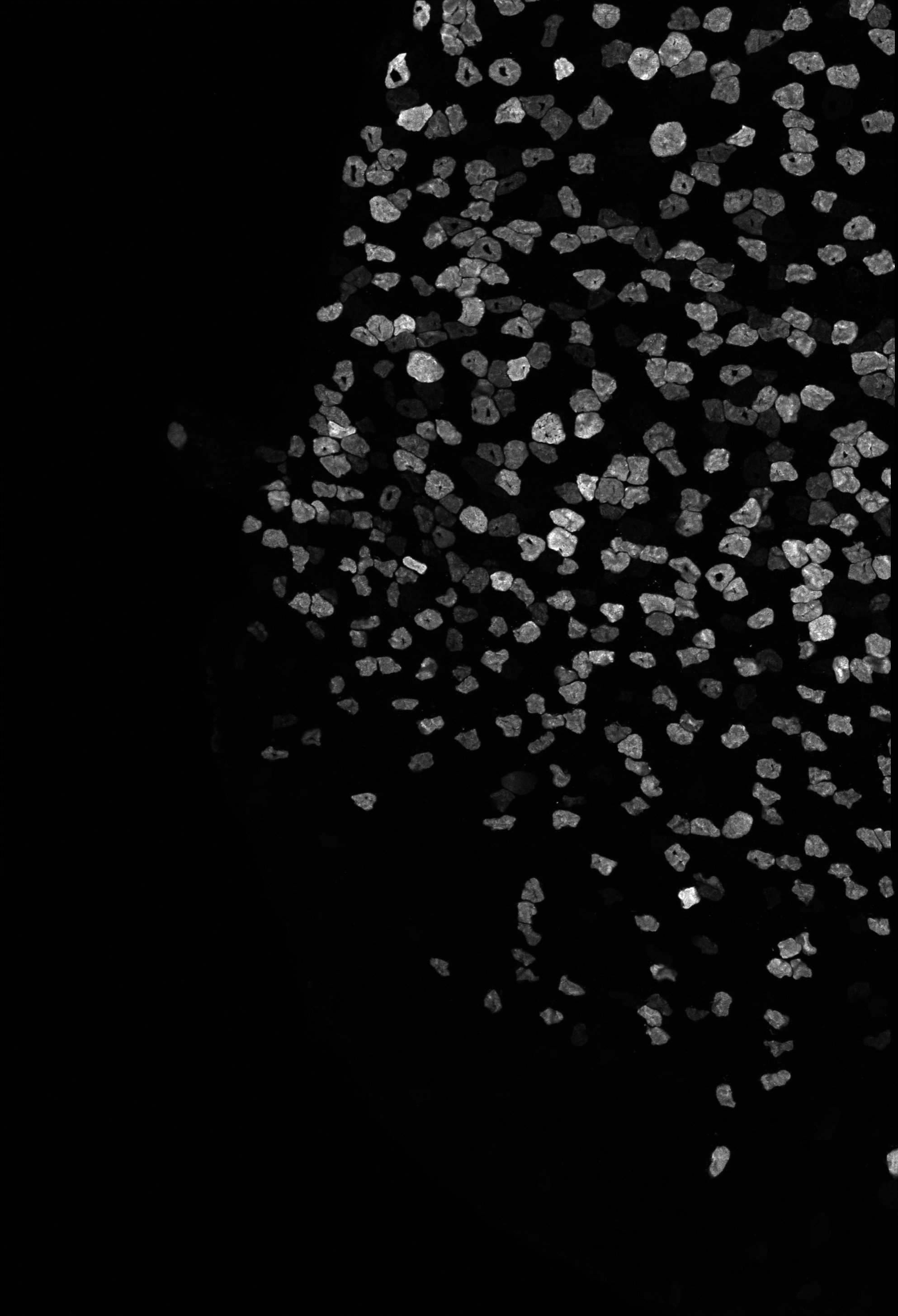

Supplement: Supplementary file 24 — Source data Fig. 7 [file 44318_2024_242_MOESM24_ESM.zip › Figure 7/I/Fig7I_G58R_Dele1KO_middle_type2a_20x.tif.tif]

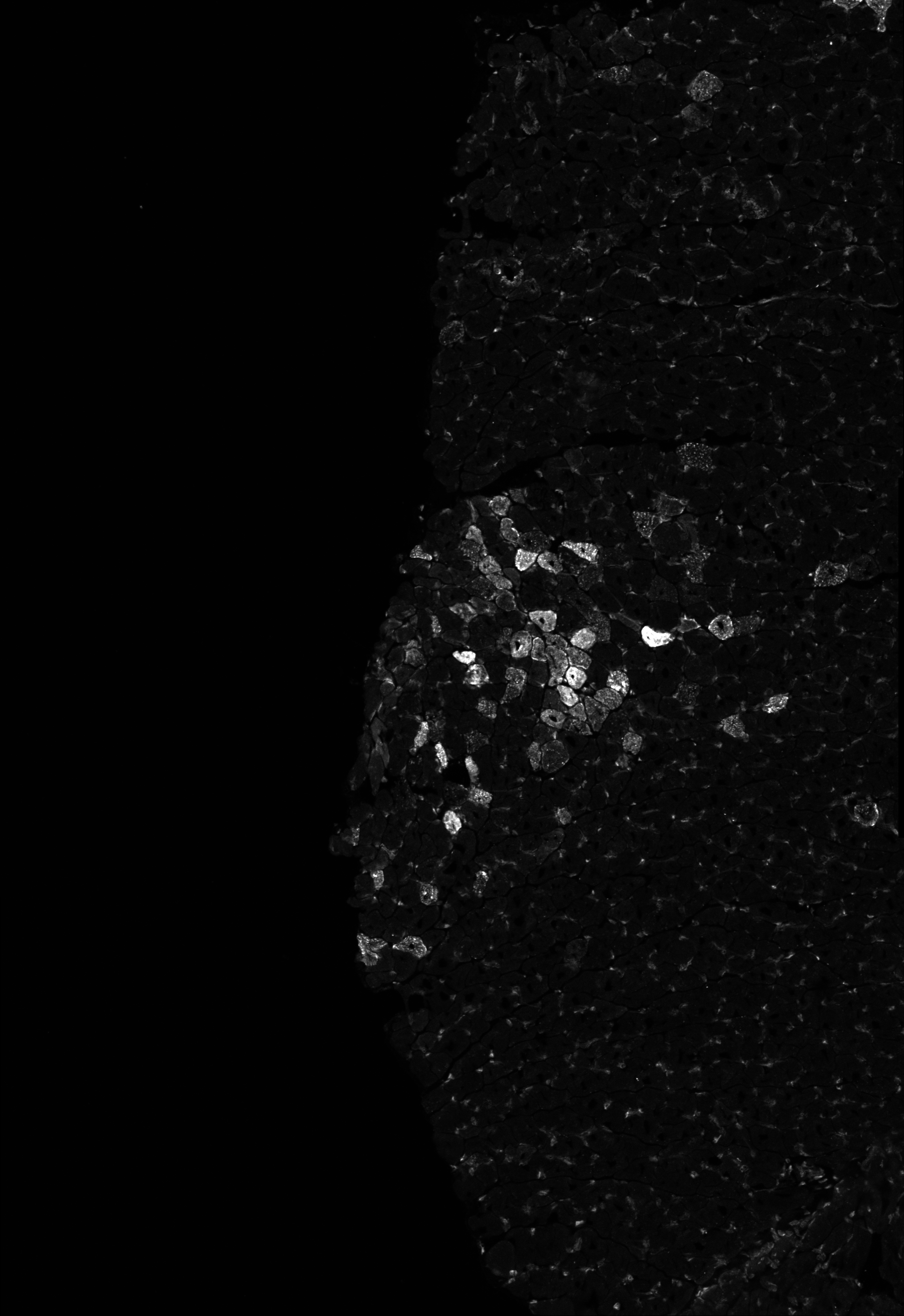

Supplement: Supplementary file 24 — Source data Fig. 7 [file 44318_2024_242_MOESM24_ESM.zip › Figure 7/I/Fig7I_G58R_Dele1KO_right_FK2_20x.tif.tif]

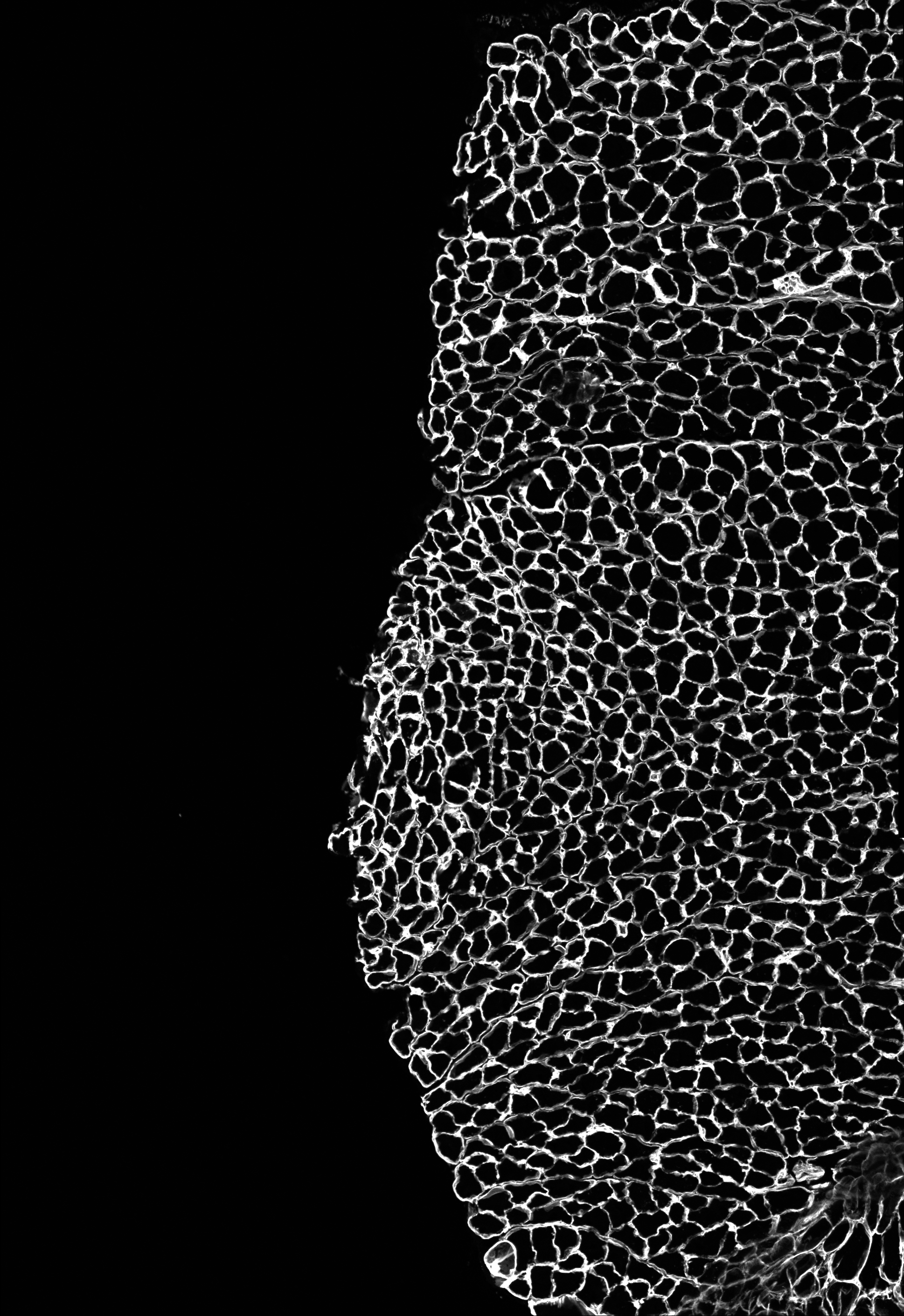

Supplement: Supplementary file 24 — Source data Fig. 7 [file 44318_2024_242_MOESM24_ESM.zip › Figure 7/I/Fig7I_G58R_Dele1KO_right_Laminin_20x.tif.tif]

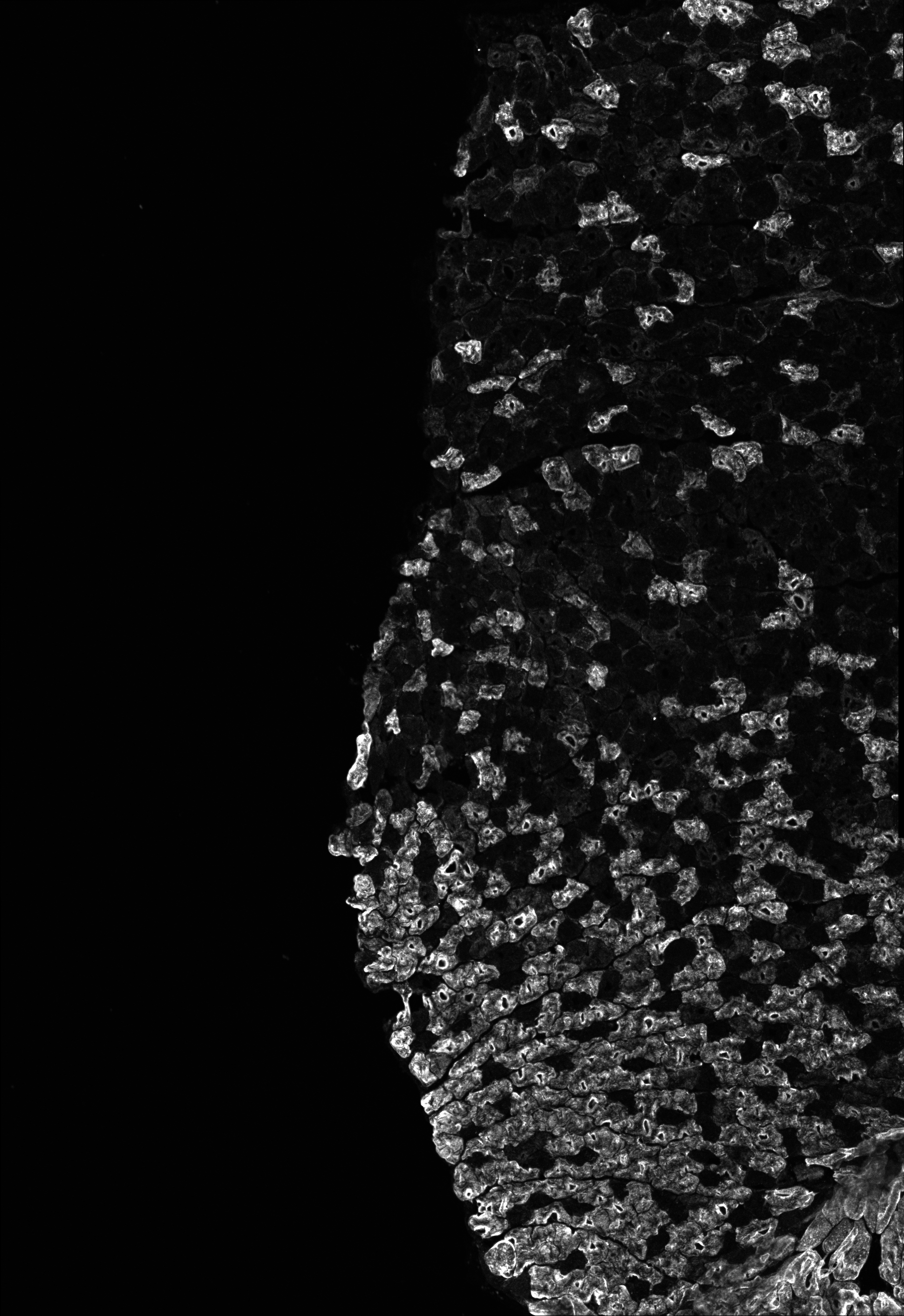

Supplement: Supplementary file 24 — Source data Fig. 7 [file 44318_2024_242_MOESM24_ESM.zip › Figure 7/I/Fig7I_G58R_Dele1KO_right_type2b_20x.tif.tif]

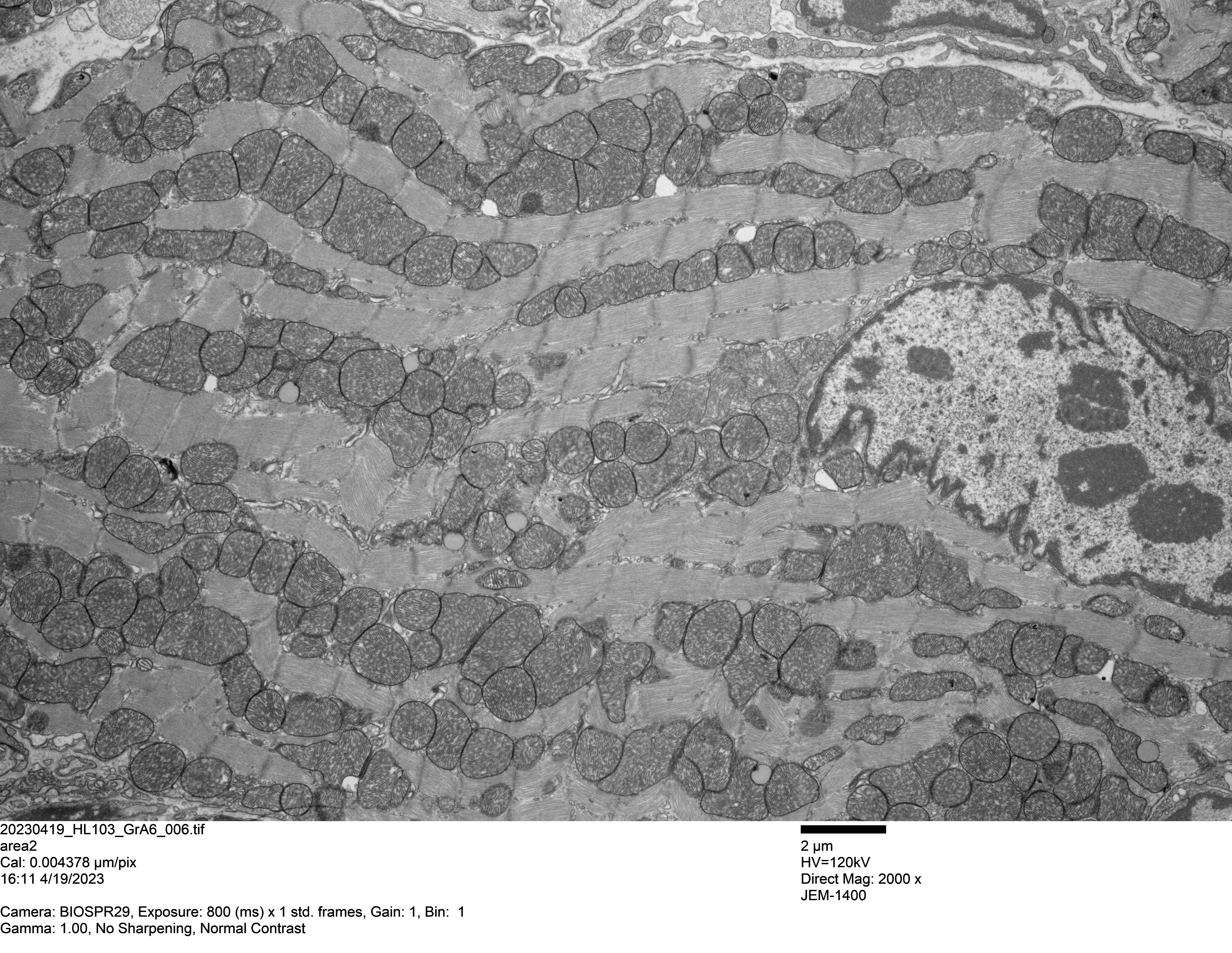

Supplement: Supplementary file 25 — Figure EV1CDEF Source Data [file 44318_2024_242_MOESM25_ESM.zip › EV1CDEF/EV1C.tif]

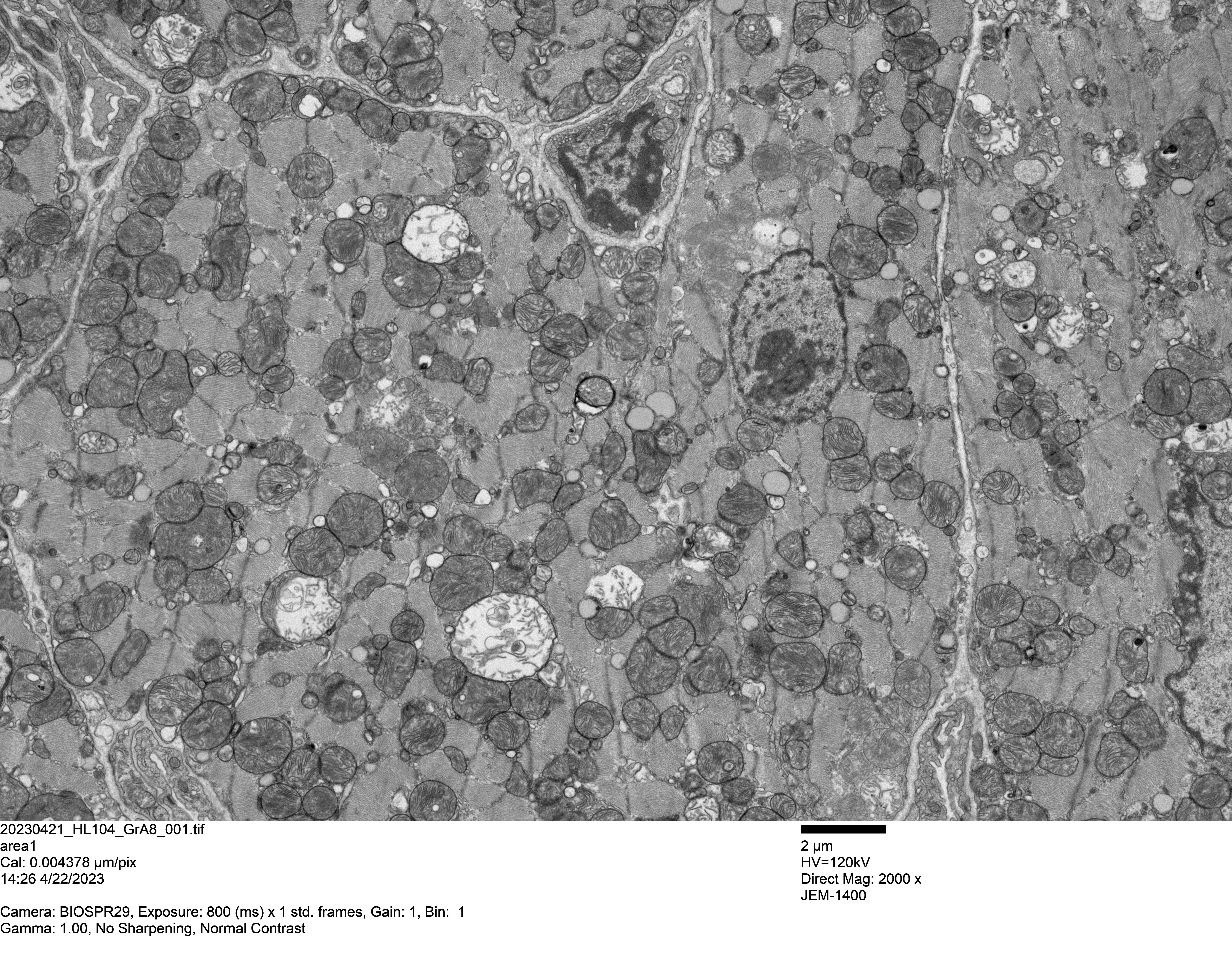

Supplement: Supplementary file 25 — Figure EV1CDEF Source Data [file 44318_2024_242_MOESM25_ESM.zip › EV1CDEF/EV1D.tif]

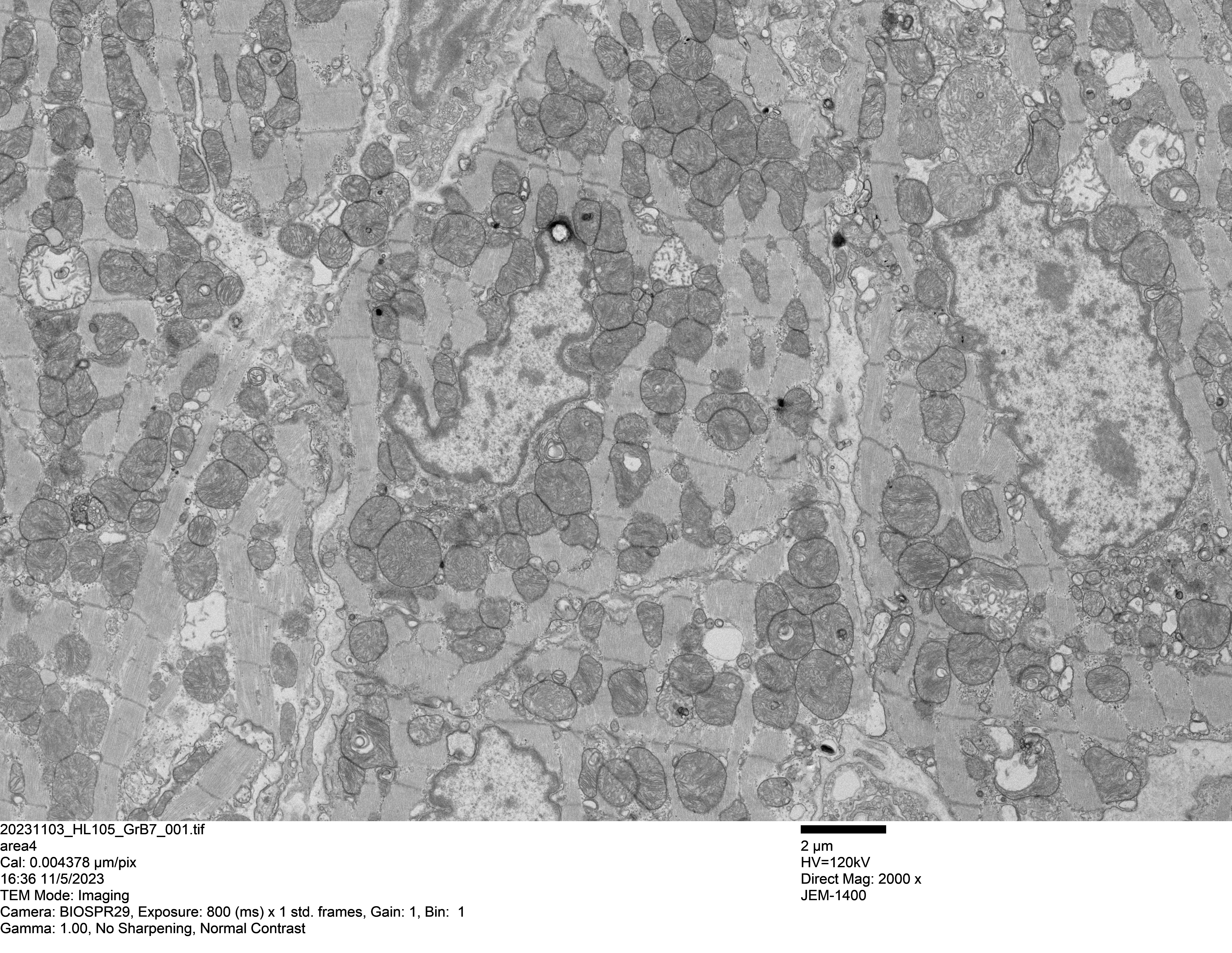

Supplement: Supplementary file 25 — Figure EV1CDEF Source Data [file 44318_2024_242_MOESM25_ESM.zip › EV1CDEF/EV1E.tif]

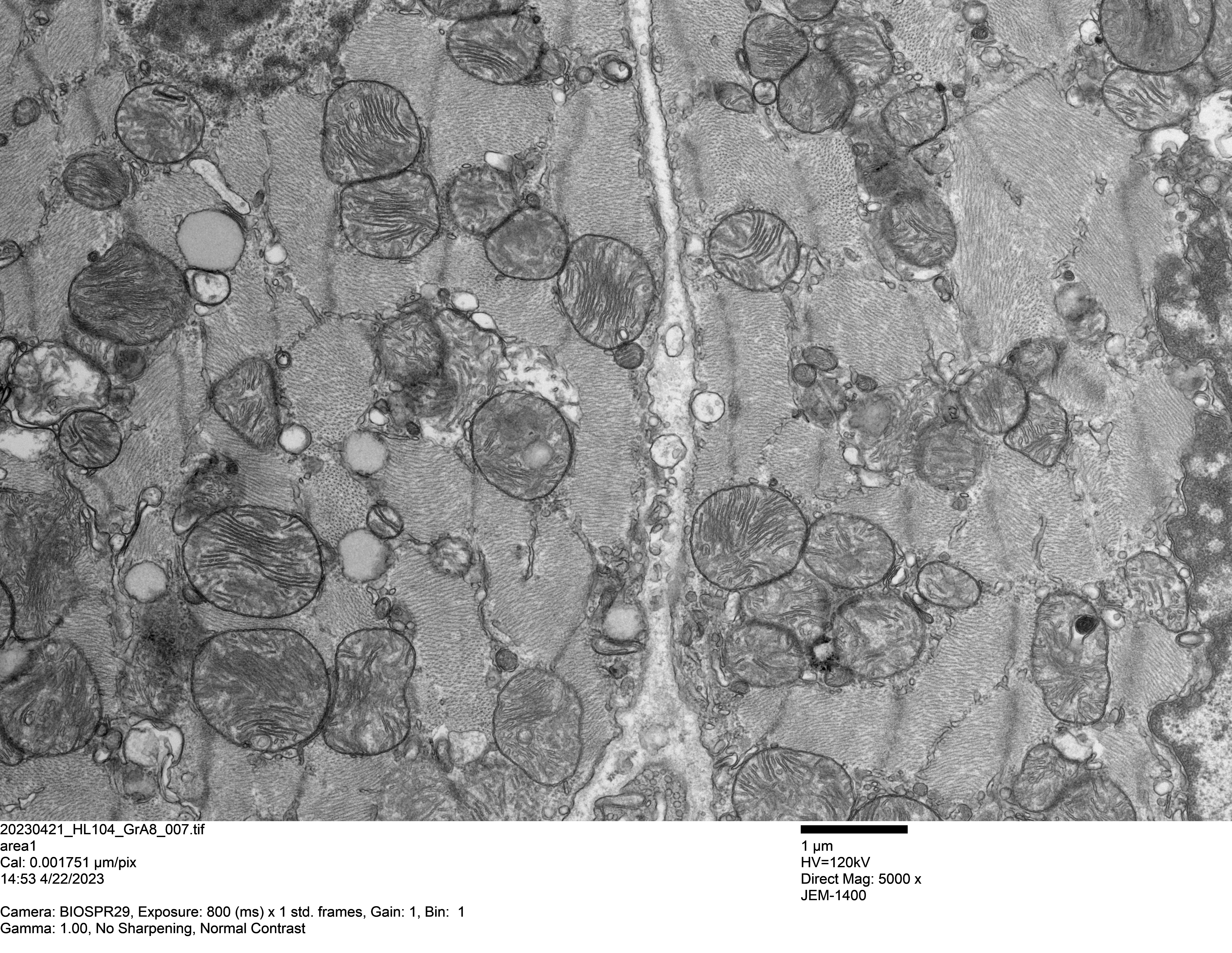

Supplement: Supplementary file 25 — Figure EV1CDEF Source Data [file 44318_2024_242_MOESM25_ESM.zip › EV1CDEF/EV1F.tif]

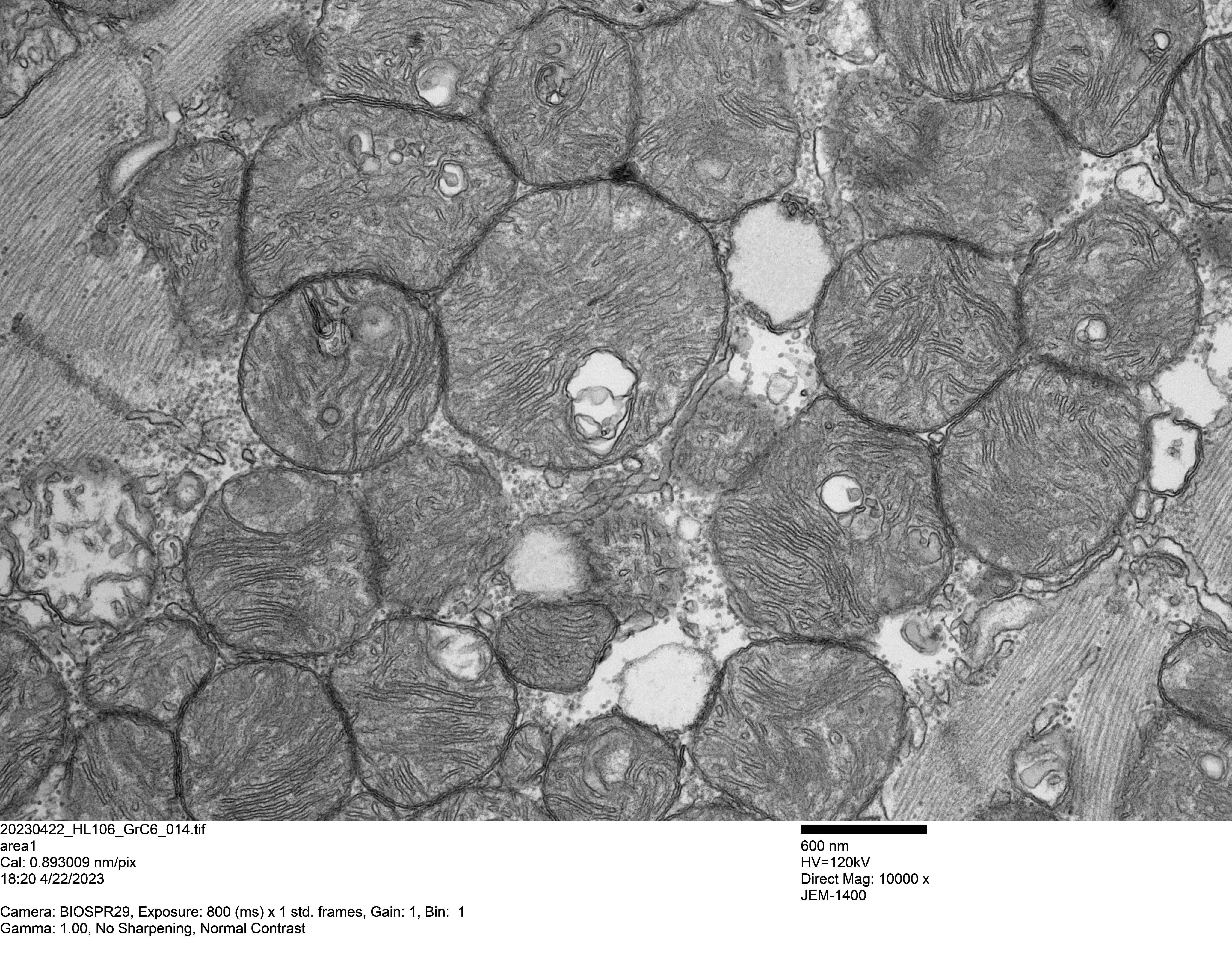

Supplement: Supplementary file 26 — Figure EV1G Source Data [file 44318_2024_242_MOESM26_ESM.zip › EV1G/EV1G_bottom_left.tif]

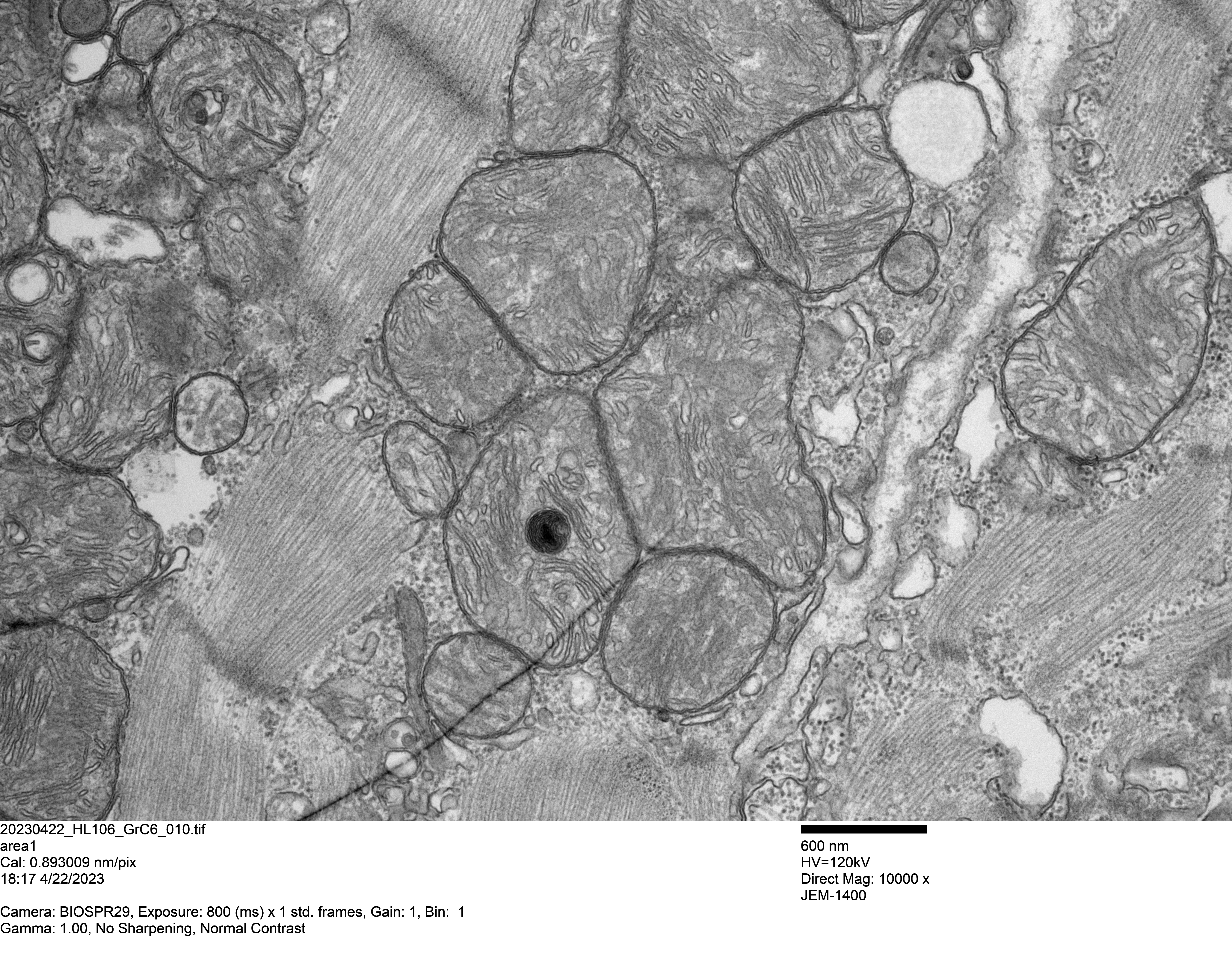

Supplement: Supplementary file 26 — Figure EV1G Source Data [file 44318_2024_242_MOESM26_ESM.zip › EV1G/EV1G_bottom_right.tif]

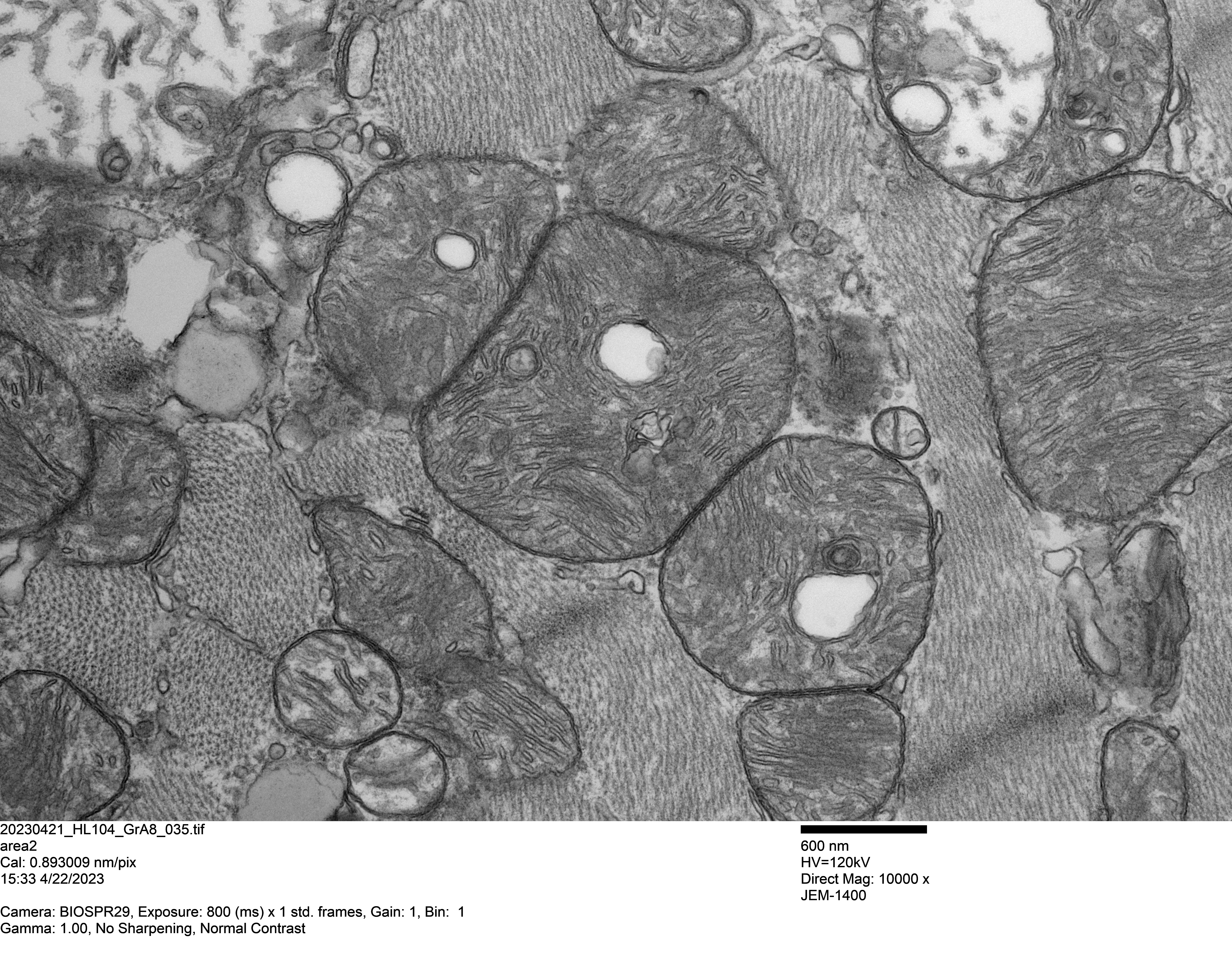

Supplement: Supplementary file 26 — Figure EV1G Source Data [file 44318_2024_242_MOESM26_ESM.zip › EV1G/EV1G_top_left.tif]

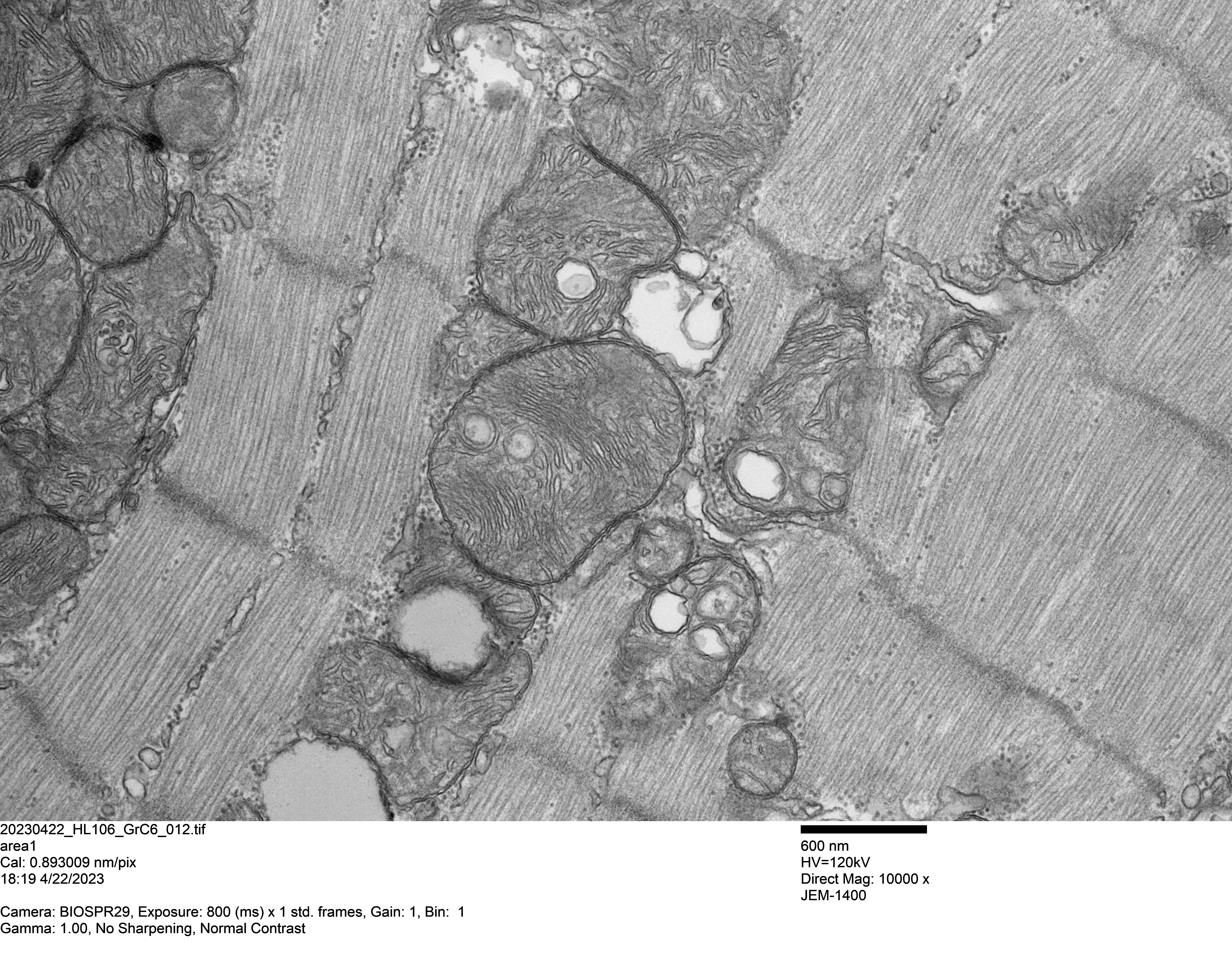

Supplement: Supplementary file 26 — Figure EV1G Source Data [file 44318_2024_242_MOESM26_ESM.zip › EV1G/EV1G_top_right.tif]

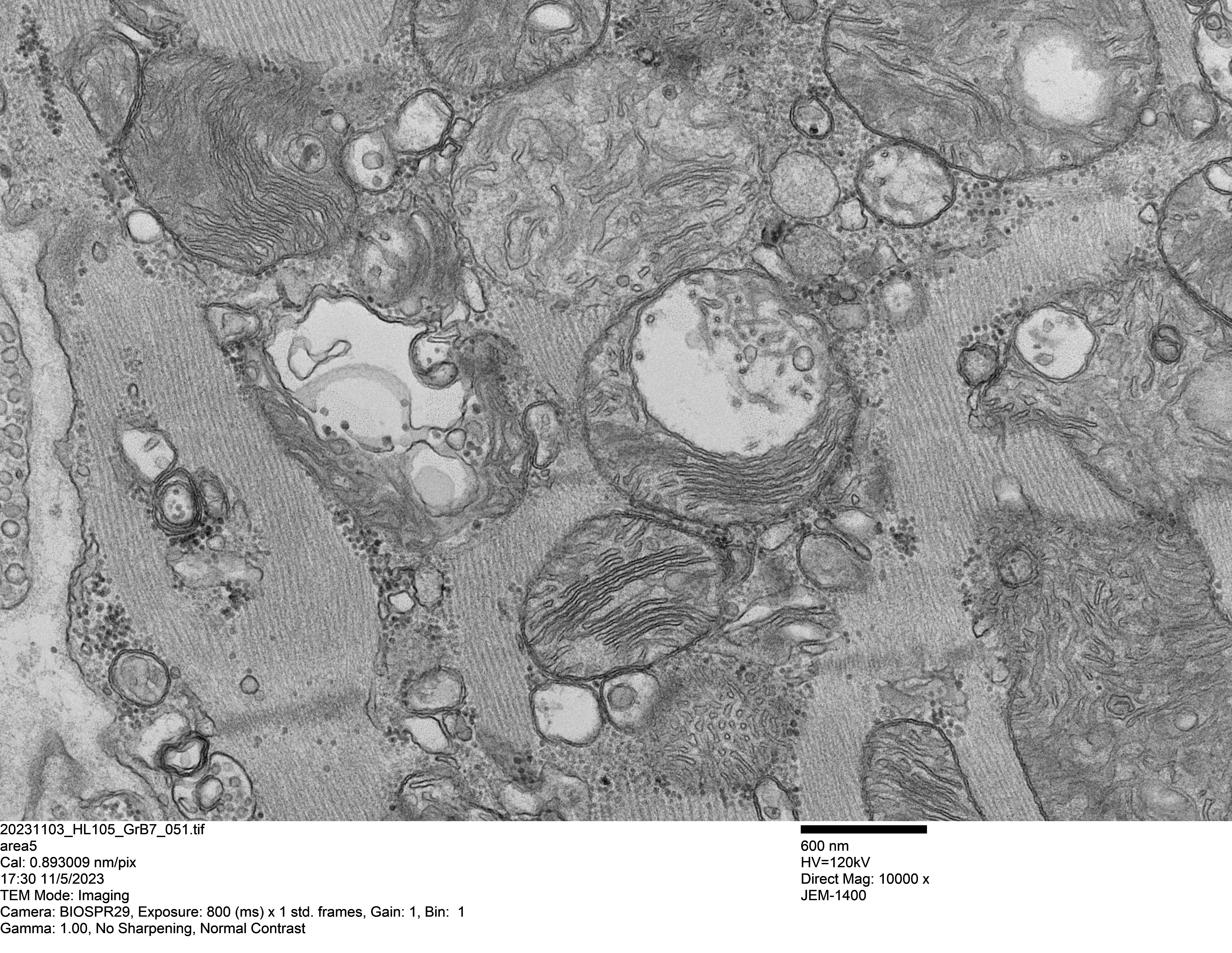

Supplement: Supplementary file 27 — Figure EV1H Source Data [file 44318_2024_242_MOESM27_ESM.zip › EV1H/EV1H_bottom_and_J_top.tif]

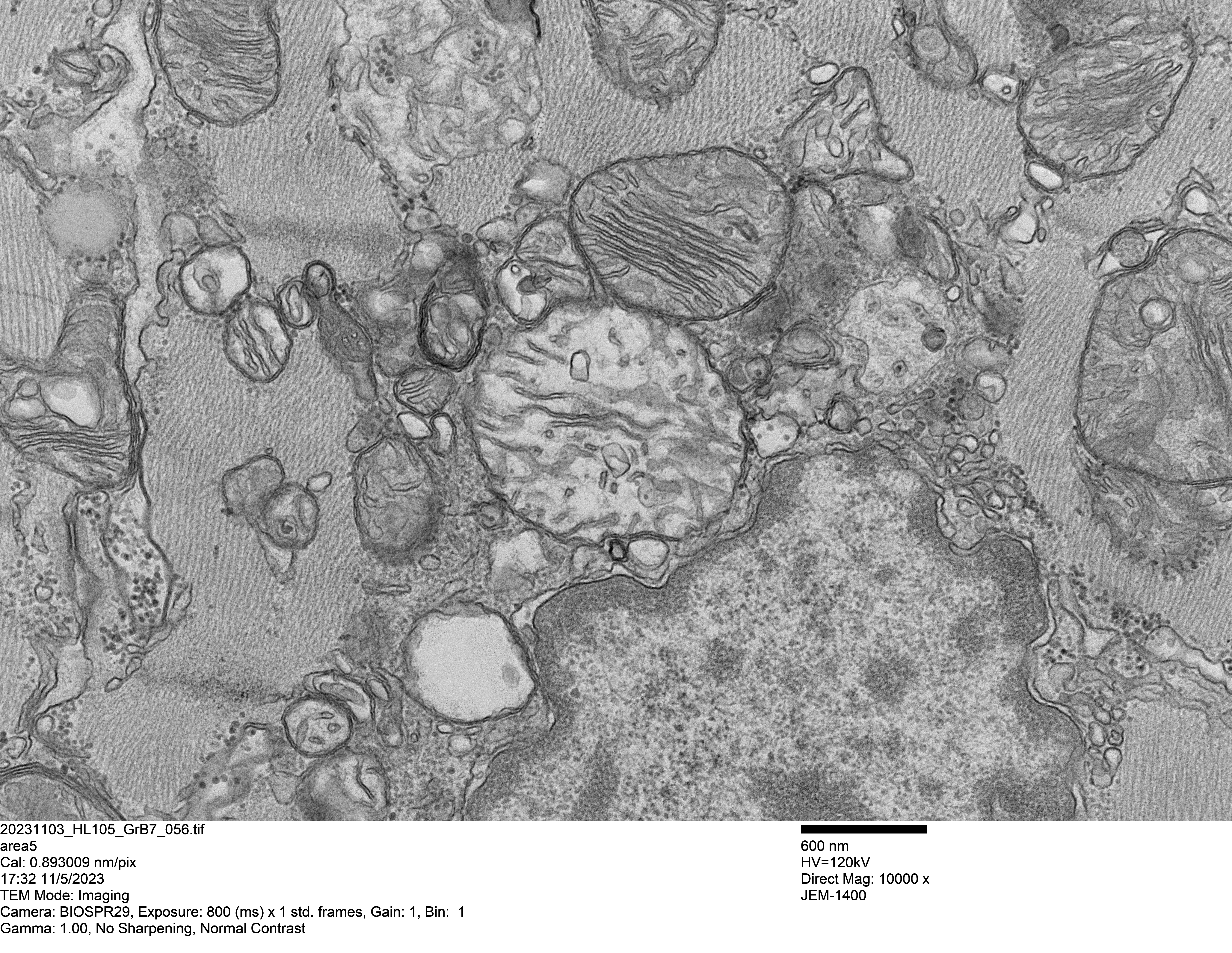

Supplement: Supplementary file 27 — Figure EV1H Source Data [file 44318_2024_242_MOESM27_ESM.zip › EV1H/EV1H_top.tif]

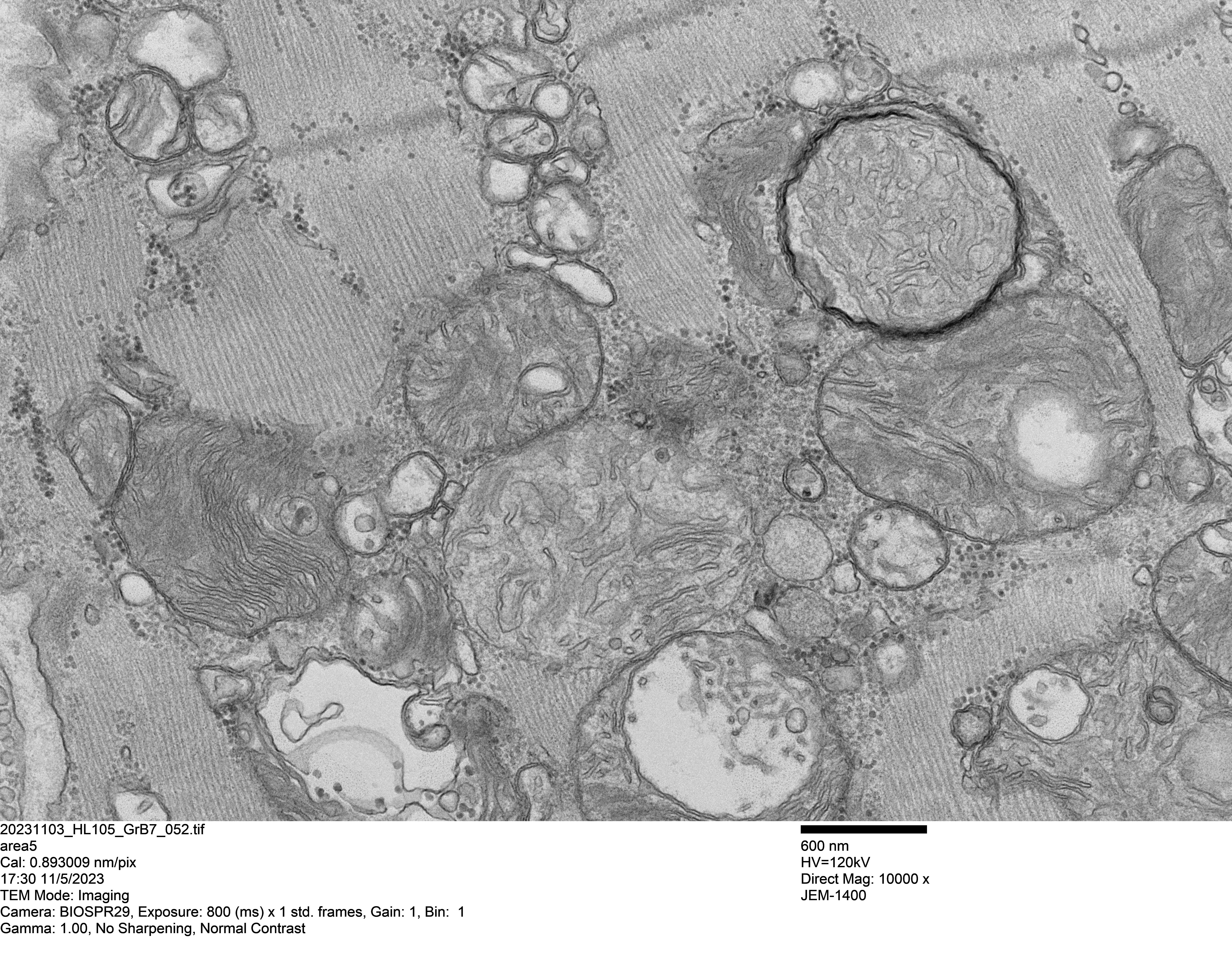

Supplement: Supplementary file 28 — Figure EV1HIJ Source Data [file 44318_2024_242_MOESM28_ESM.zip › EV1HIJ/EV1I_and_J_top.tif]

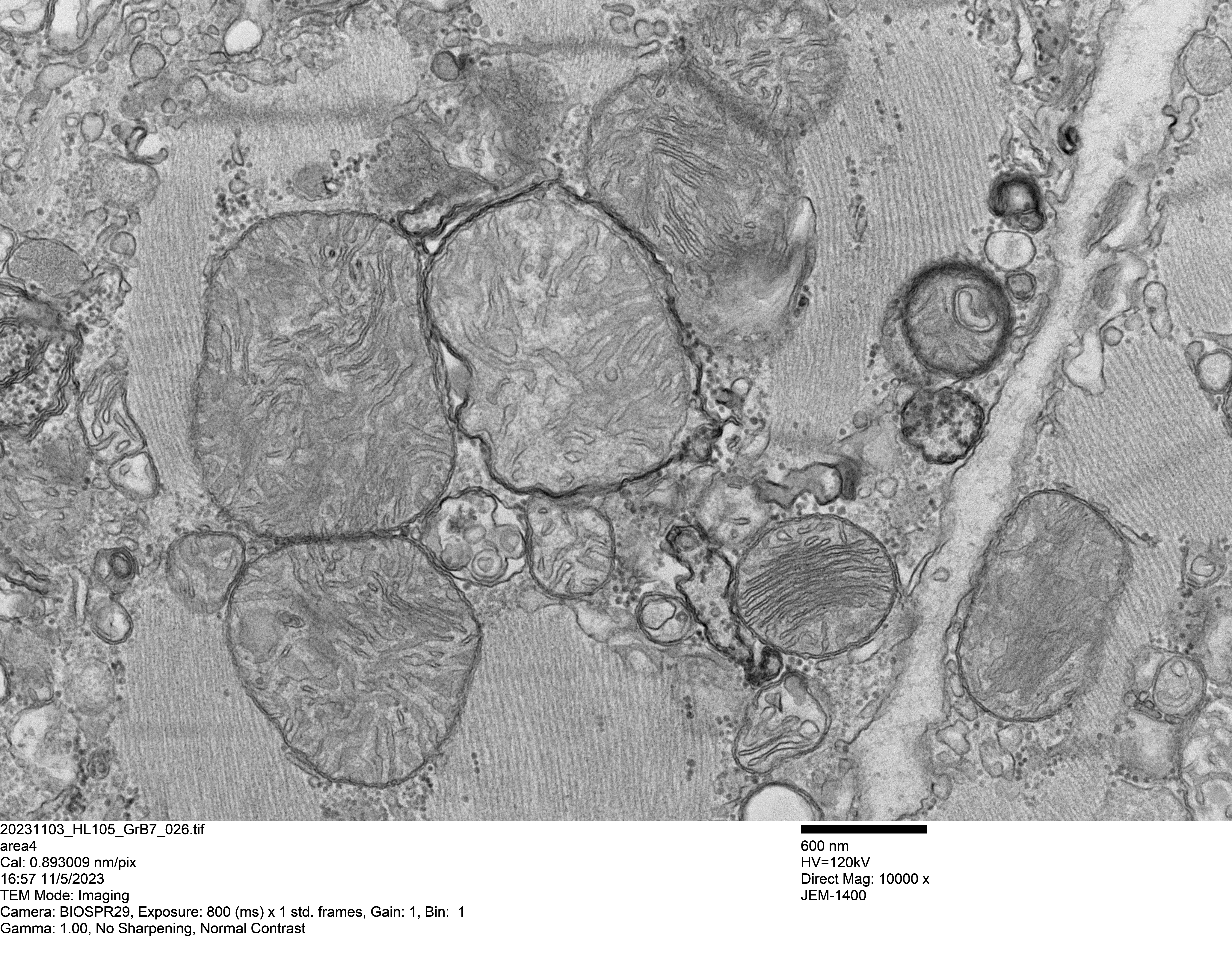

Supplement: Supplementary file 28 — Figure EV1HIJ Source Data [file 44318_2024_242_MOESM28_ESM.zip › EV1HIJ/EV1I_bottom.tif]

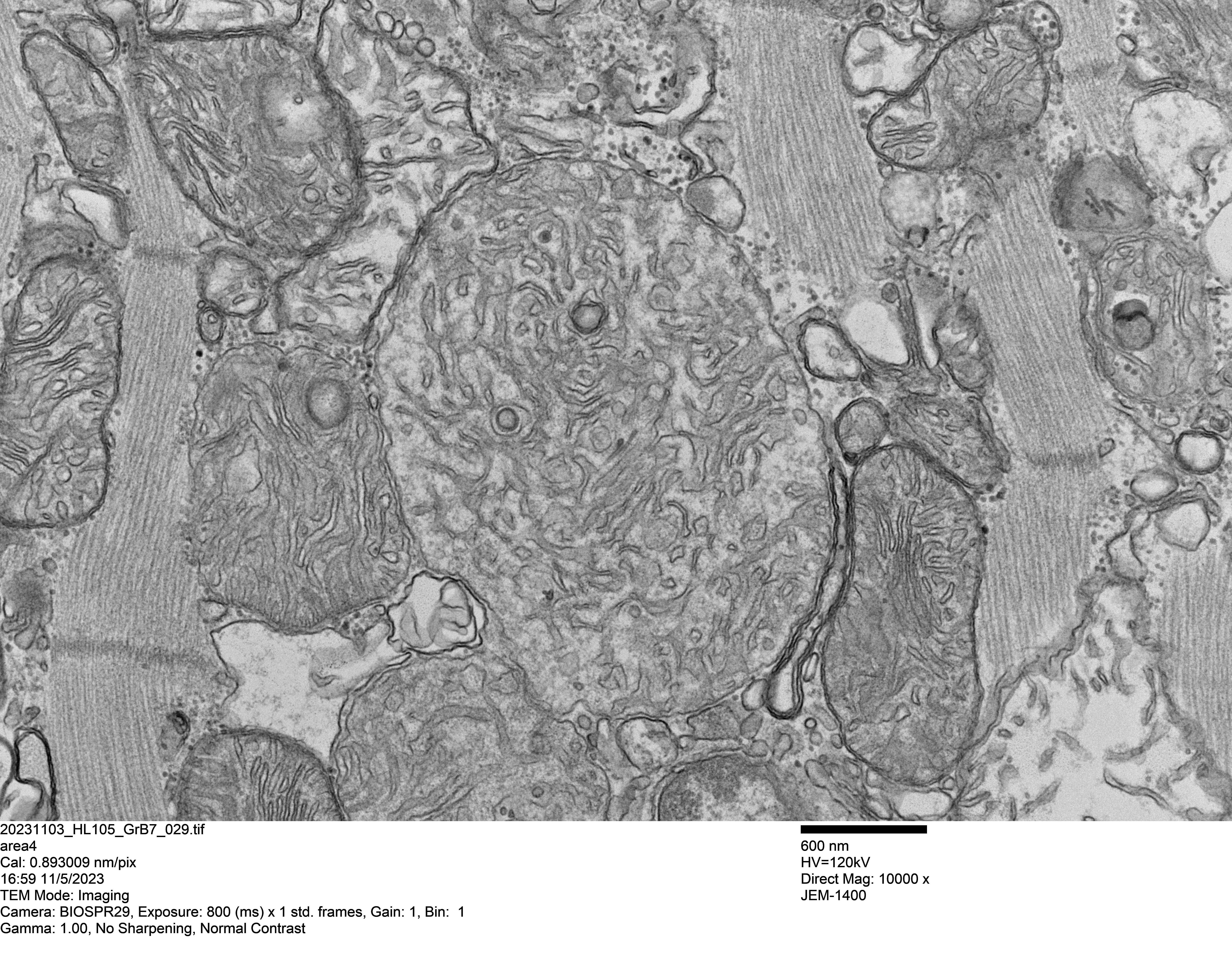

Supplement: Supplementary file 28 — Figure EV1HIJ Source Data [file 44318_2024_242_MOESM28_ESM.zip › EV1HIJ/EV1J_bottom.tif]

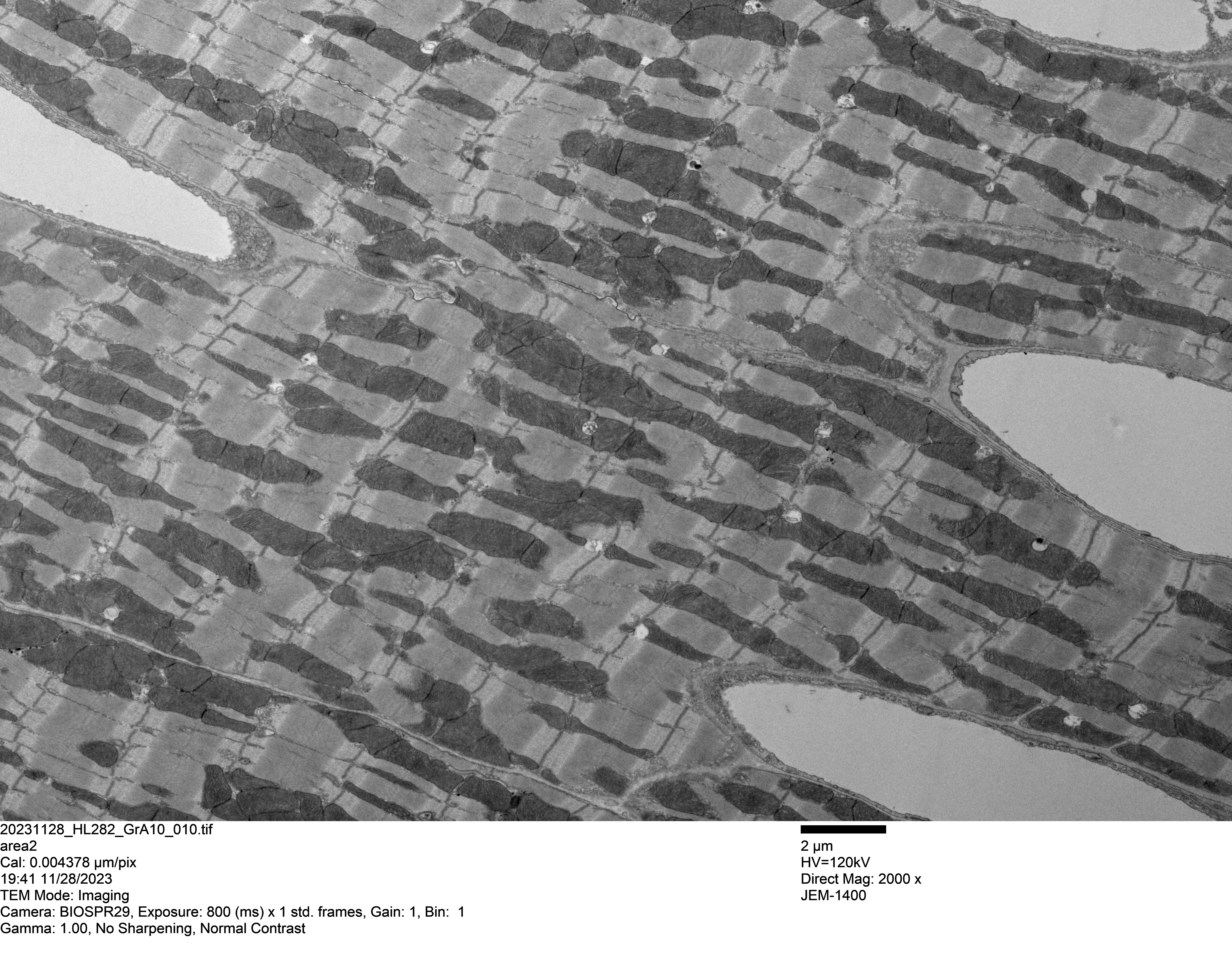

Supplement: Supplementary file 29 — Figure EV2ABC Source Data [file 44318_2024_242_MOESM29_ESM.zip › EV2ABC/EV2A.tif]

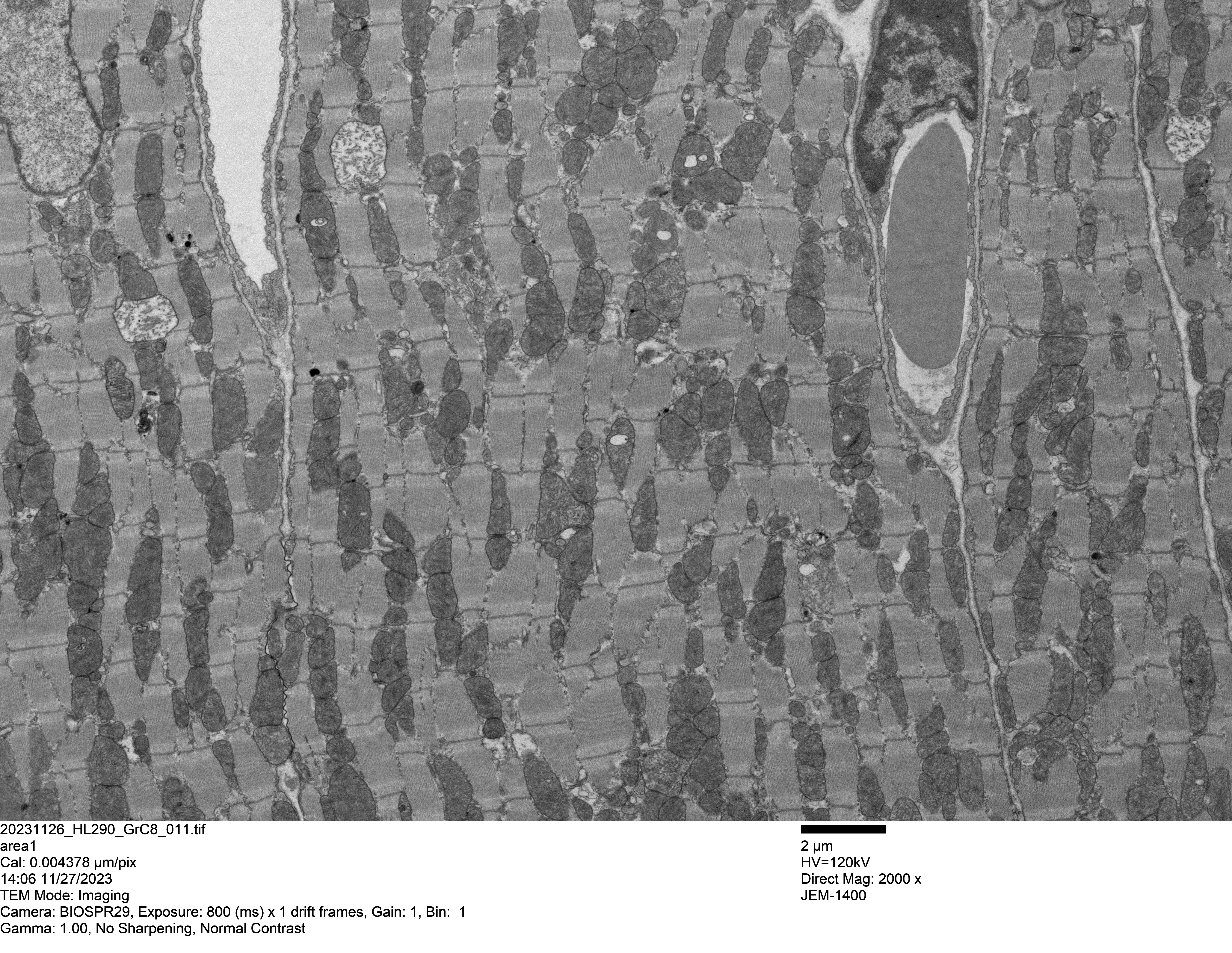

Supplement: Supplementary file 29 — Figure EV2ABC Source Data [file 44318_2024_242_MOESM29_ESM.zip › EV2ABC/EV2B.tif]

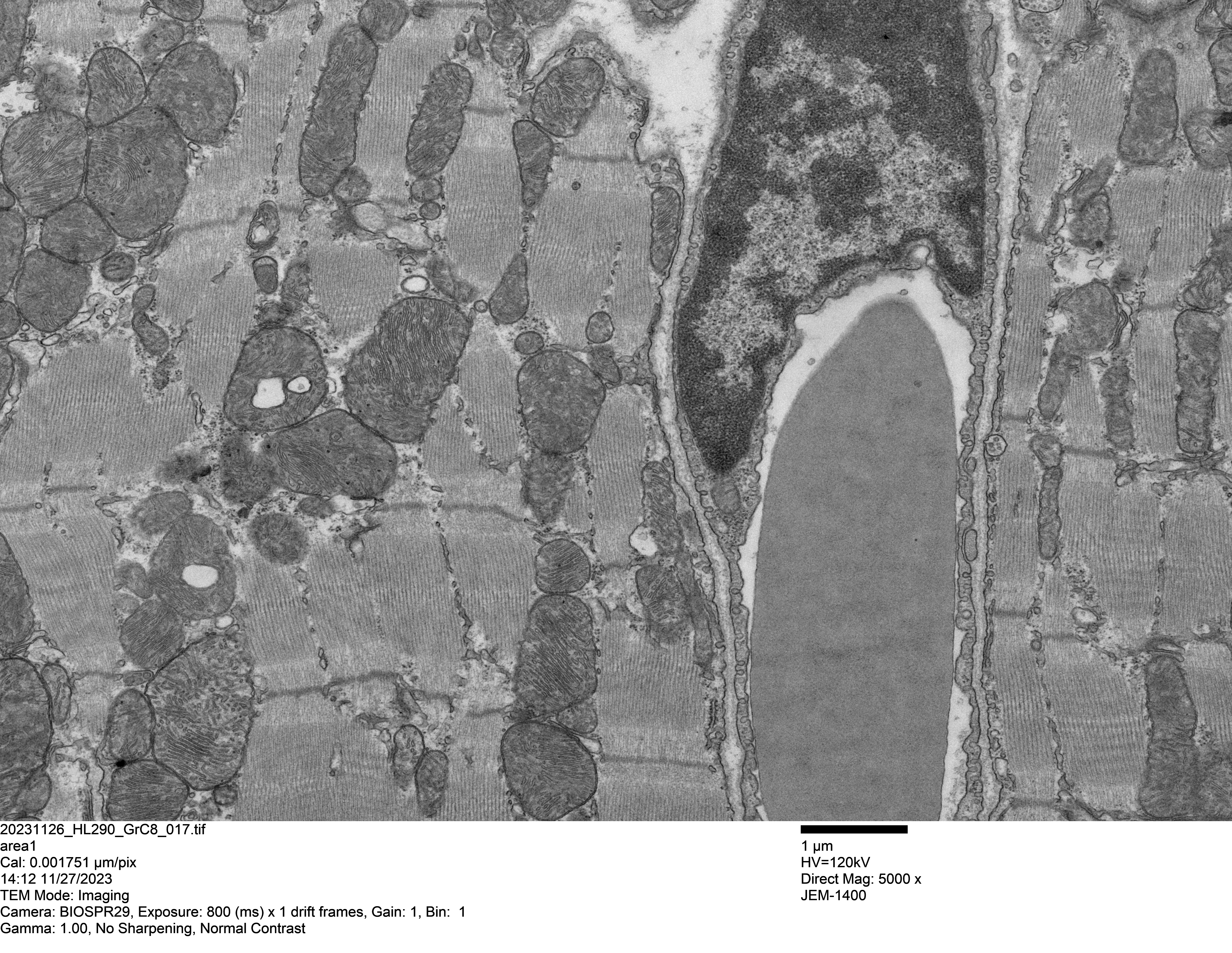

Supplement: Supplementary file 29 — Figure EV2ABC Source Data [file 44318_2024_242_MOESM29_ESM.zip › EV2ABC/EV2C.tif]

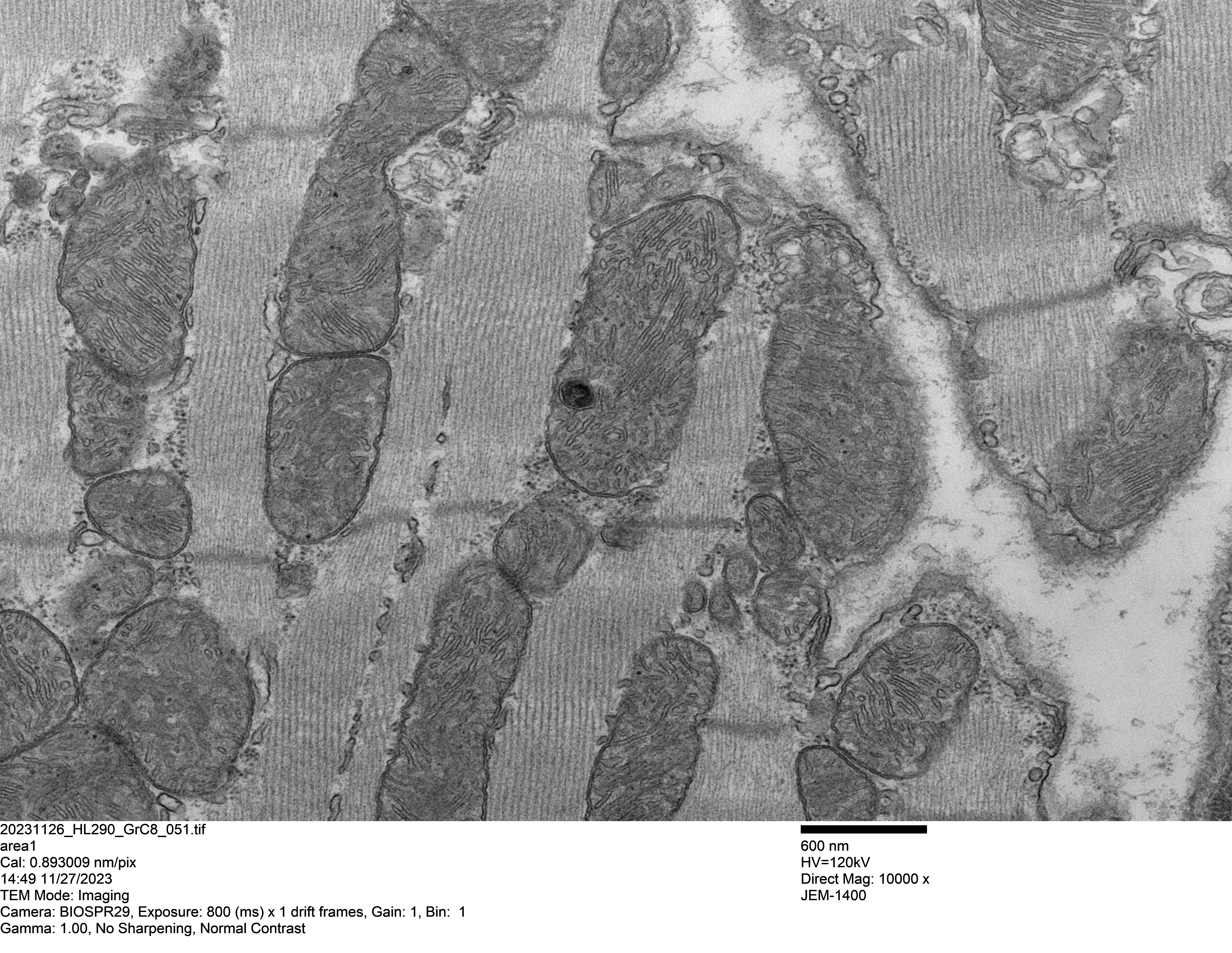

Supplement: Supplementary file 30 — Figure EV2D bottom Source Data [file 44318_2024_242_MOESM30_ESM.zip › EV2D_bottom_ middle/EV2D_bottom_left.tif]

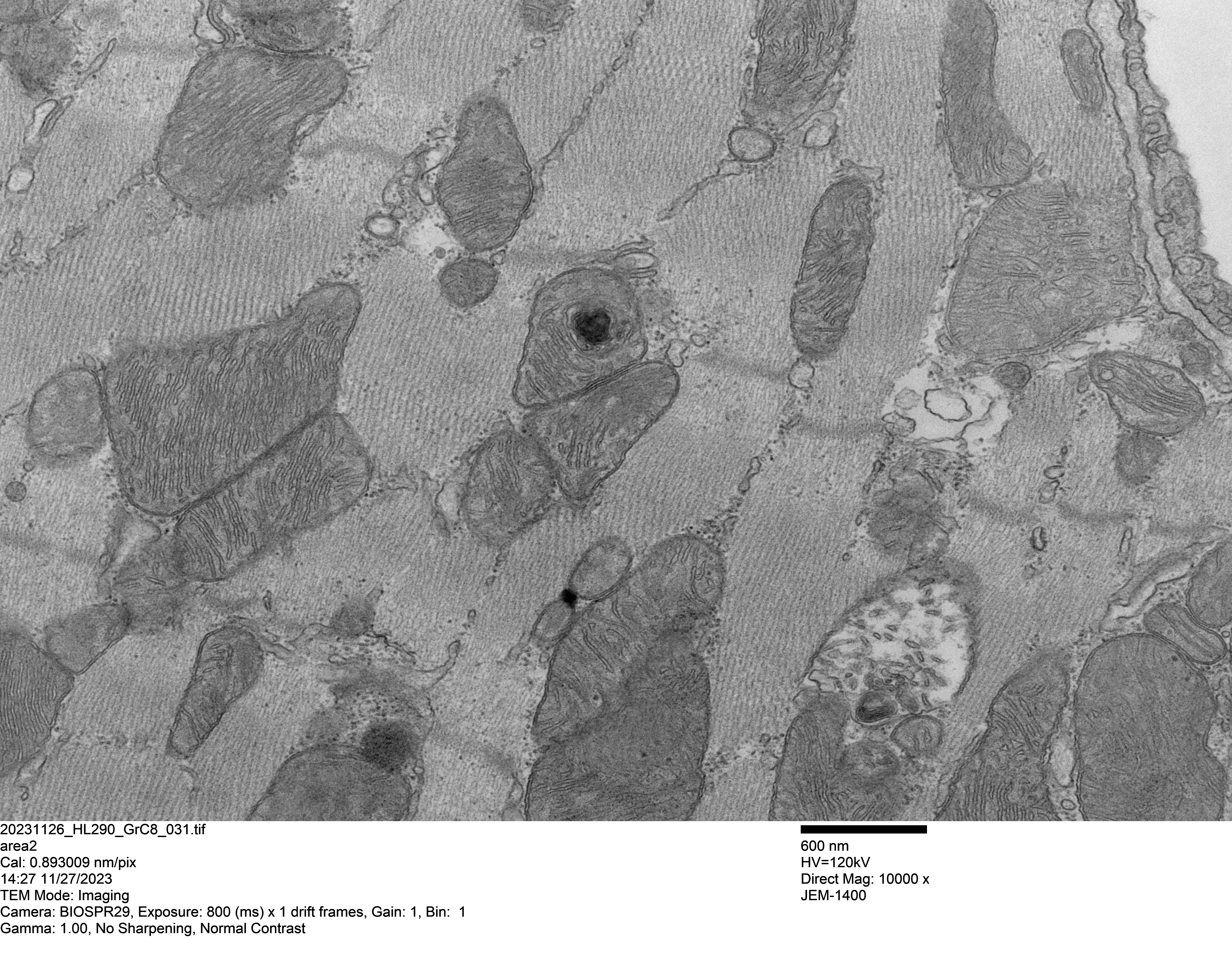

Supplement: Supplementary file 30 — Figure EV2D bottom Source Data [file 44318_2024_242_MOESM30_ESM.zip › EV2D_bottom_ middle/EV2D_bottom_right.tif]

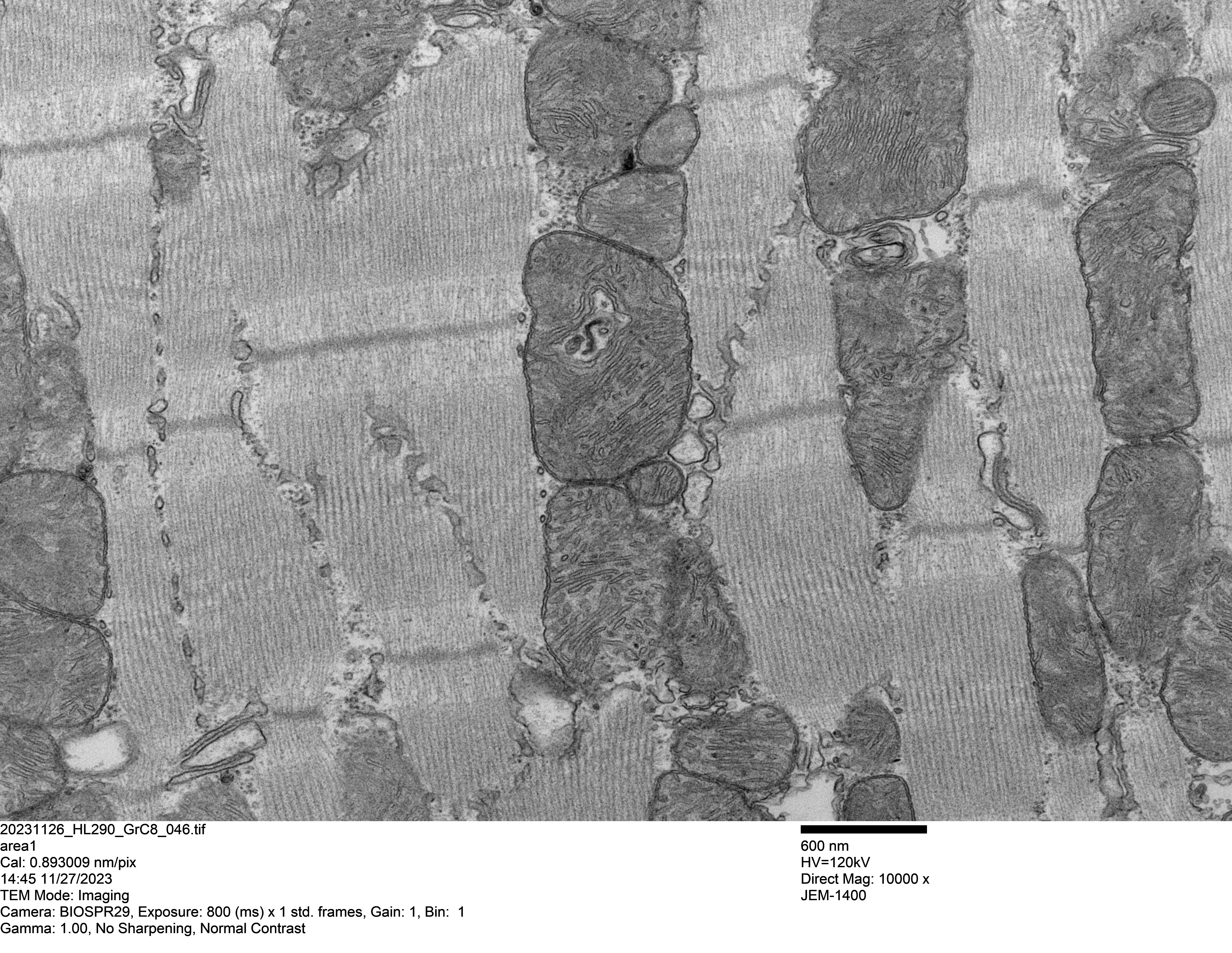

Supplement: Supplementary file 30 — Figure EV2D bottom Source Data [file 44318_2024_242_MOESM30_ESM.zip › EV2D_bottom_ middle/EV2D_middle_left.tif]

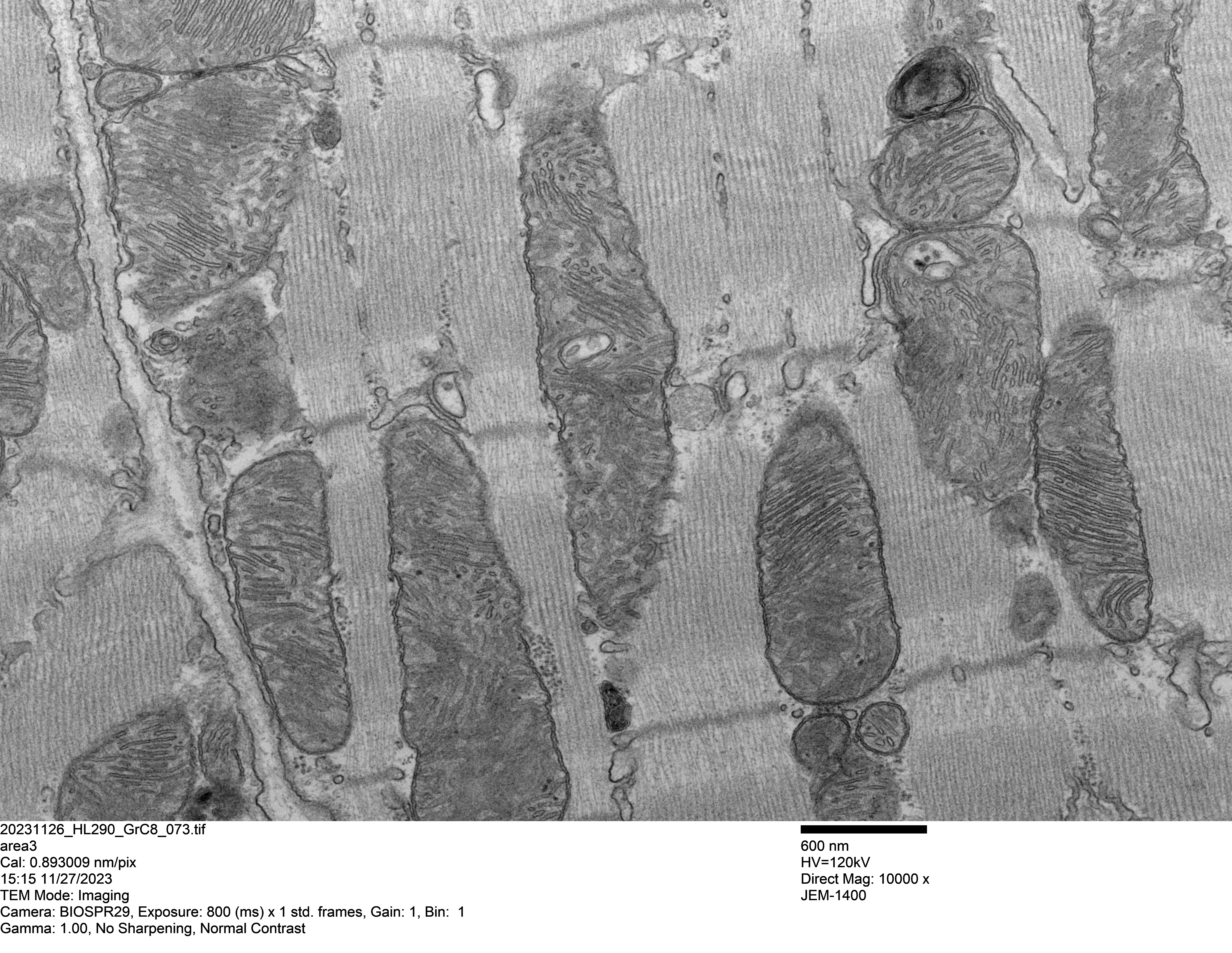

Supplement: Supplementary file 30 — Figure EV2D bottom Source Data [file 44318_2024_242_MOESM30_ESM.zip › EV2D_bottom_ middle/EV2D_middle_right.tif]

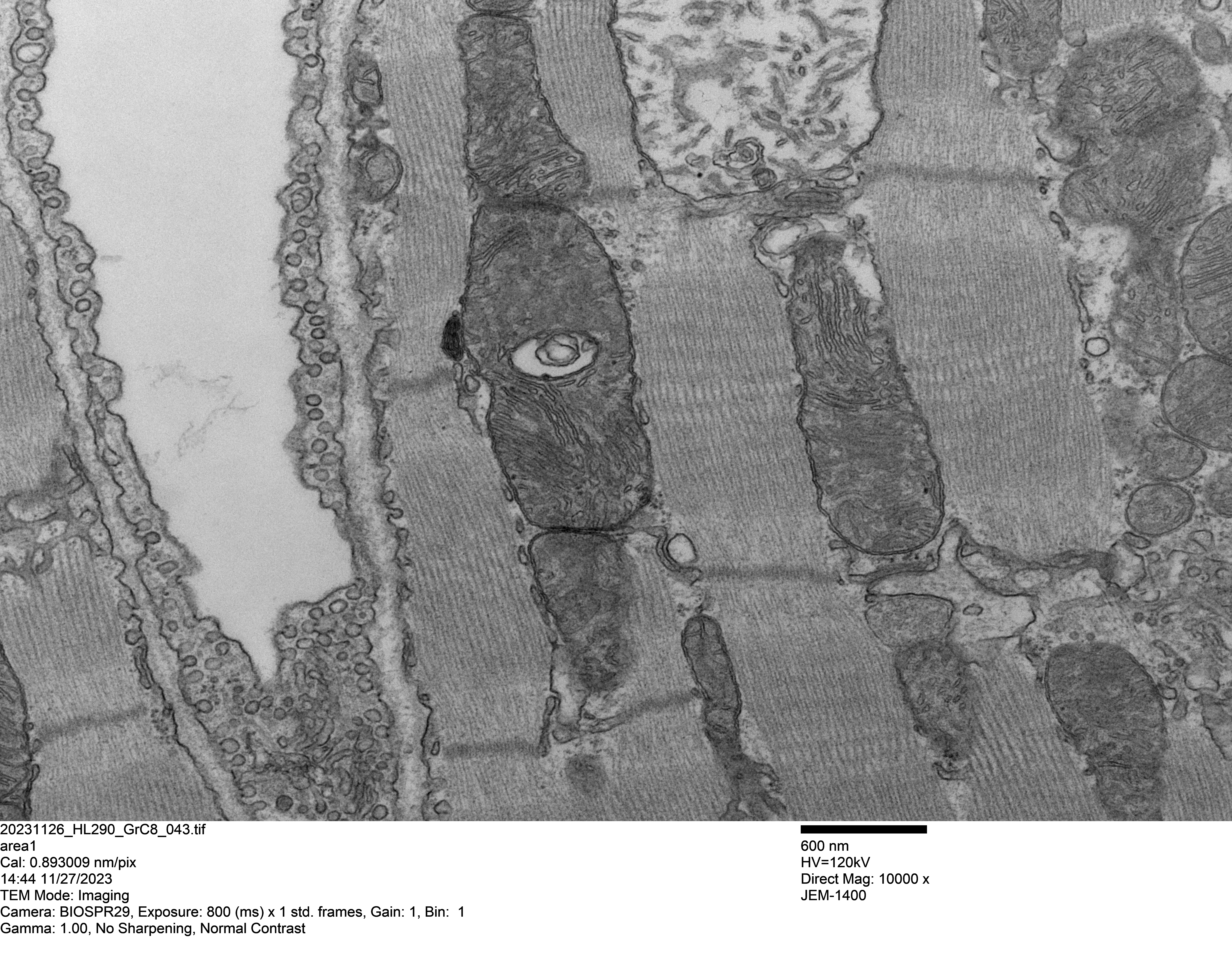

Supplement: Supplementary file 31 — Figure EV2D top Source Data [file 44318_2024_242_MOESM31_ESM.zip › EV2D_top/EV2D_top_right.tif]

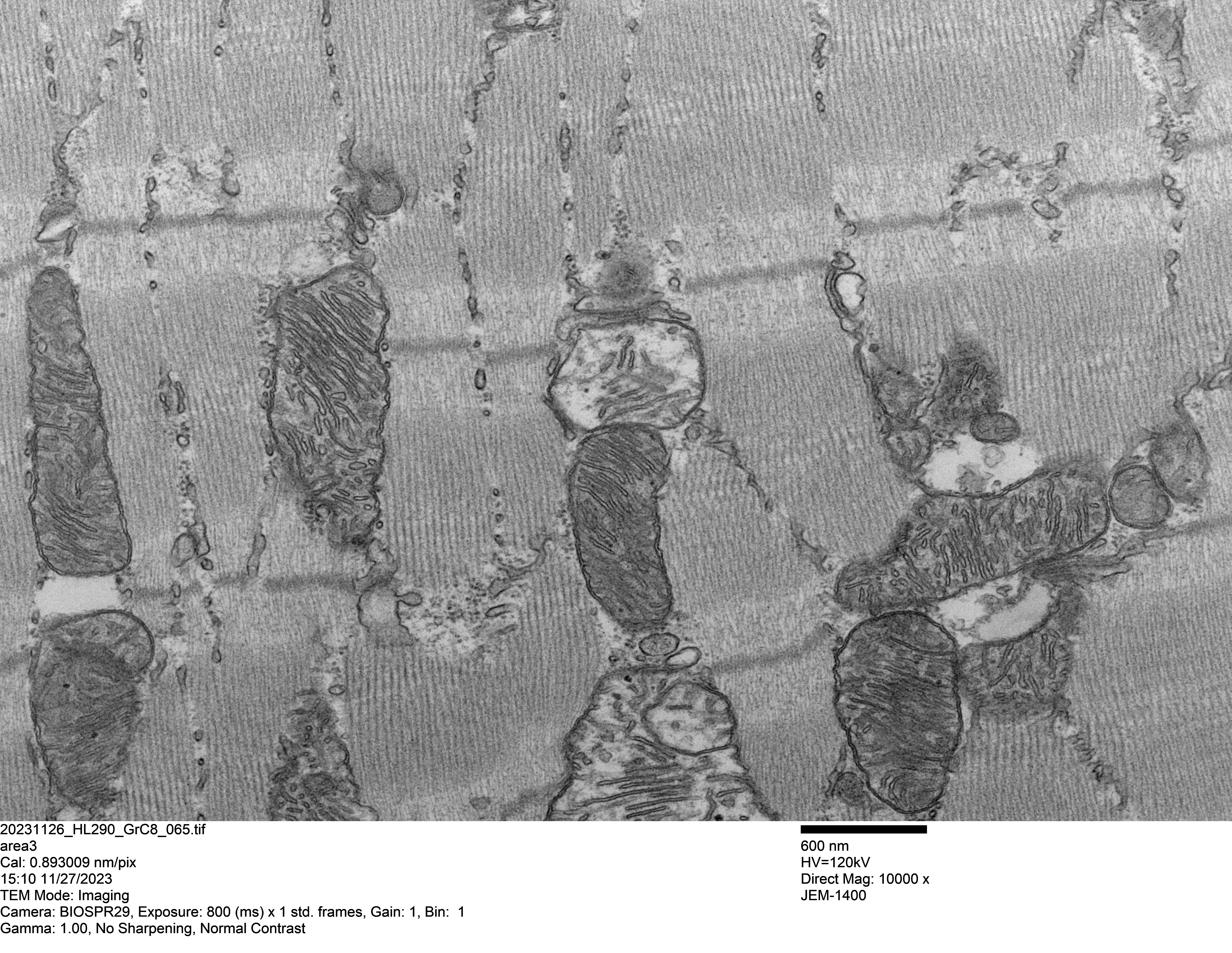

Supplement: Supplementary file 32 — Figure EV2E Source Data [file 44318_2024_242_MOESM32_ESM.zip › EV2E/EV2E_left.tif]

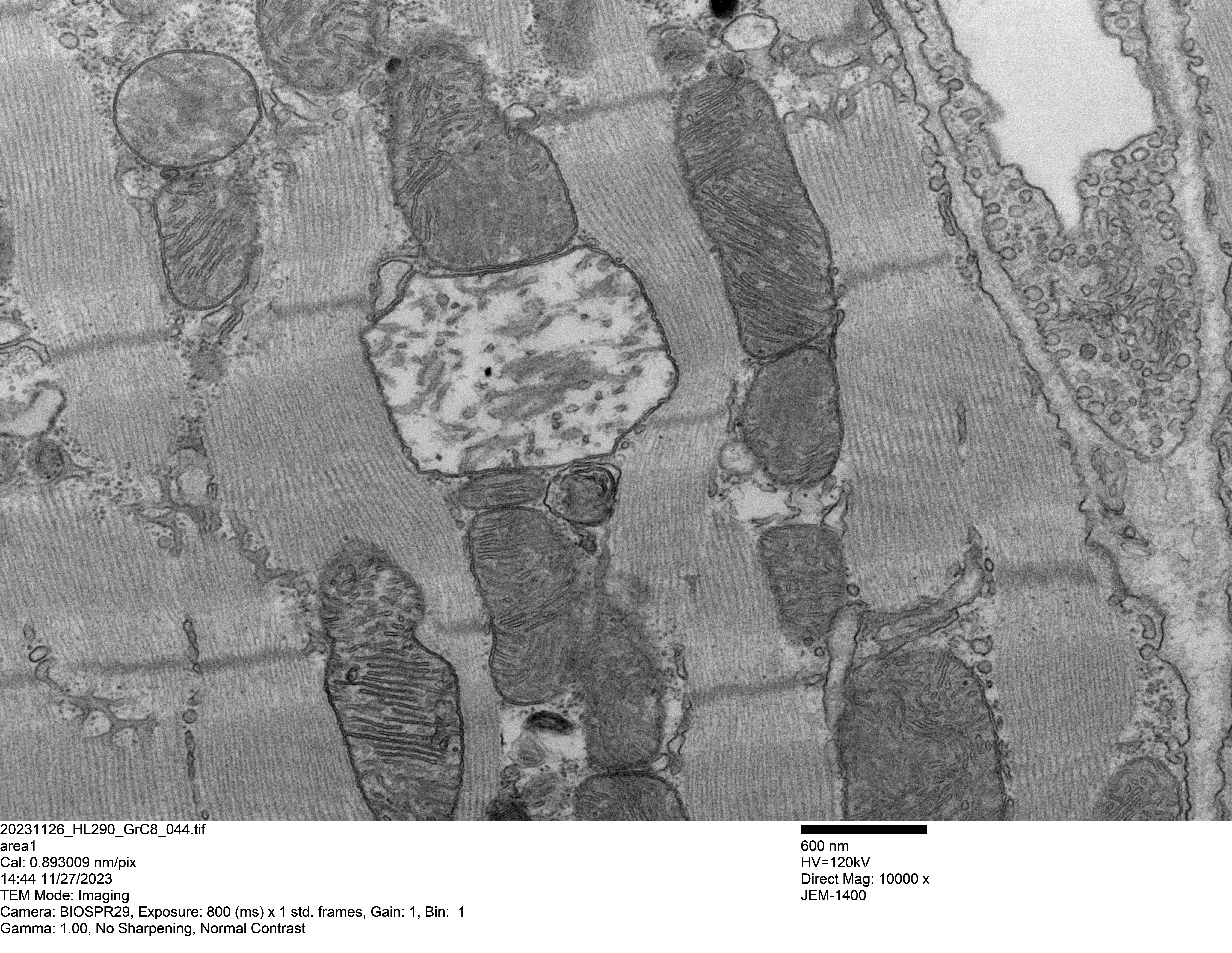

Supplement: Supplementary file 32 — Figure EV2E Source Data [file 44318_2024_242_MOESM32_ESM.zip › EV2E/EV2E_middle.tif]

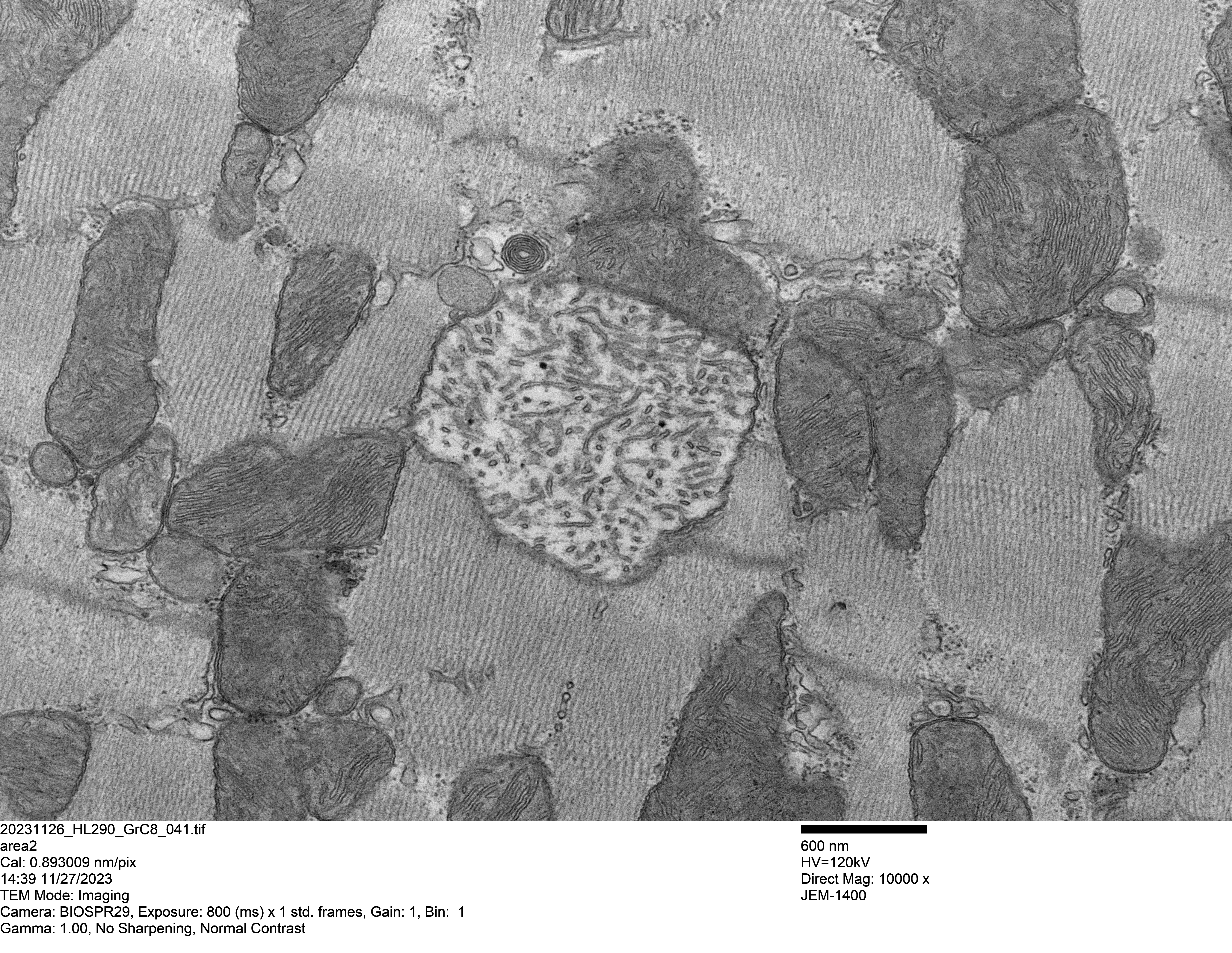

Supplement: Supplementary file 32 — Figure EV2E Source Data [file 44318_2024_242_MOESM32_ESM.zip › EV2E/EV2E_right.tif]

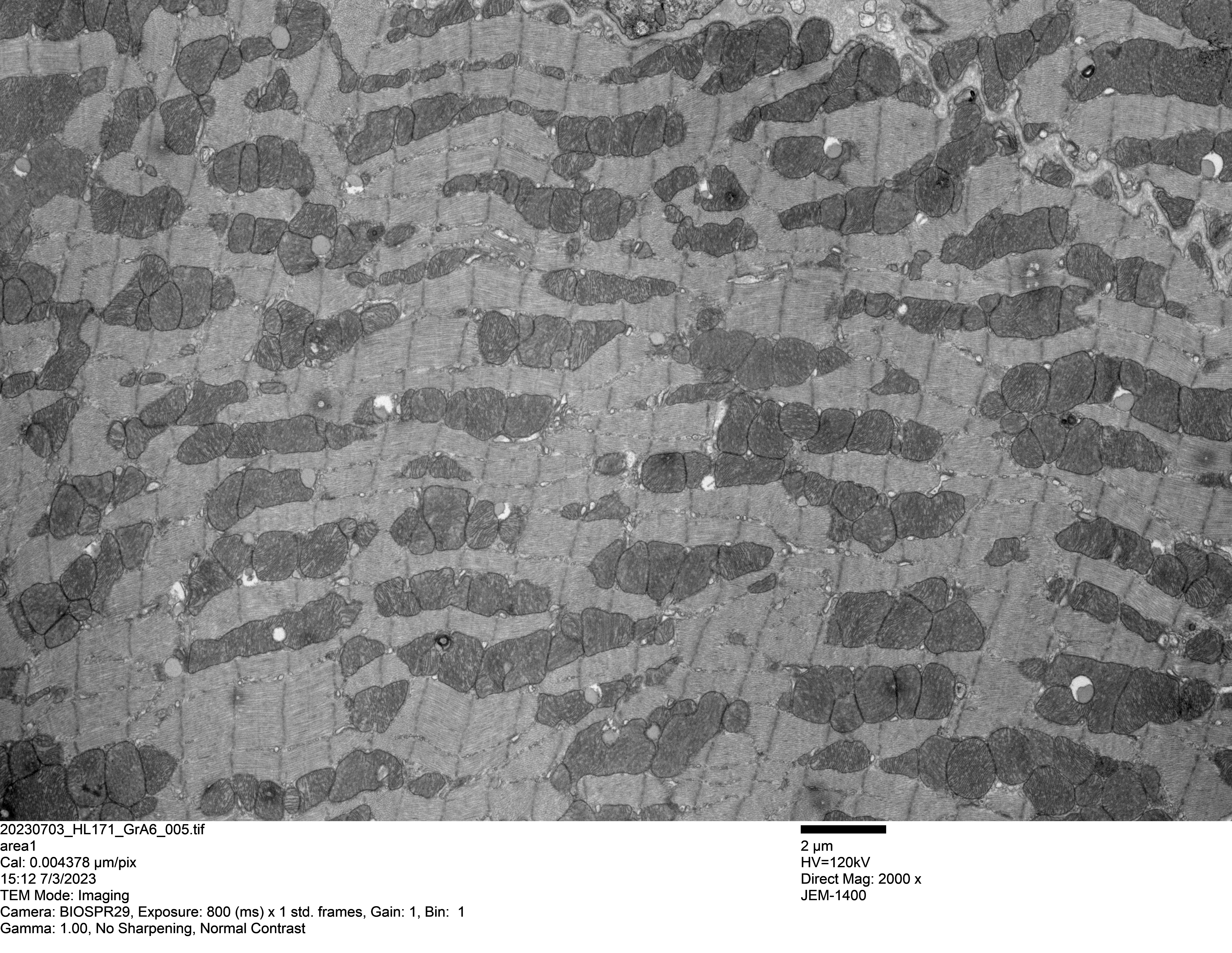

Supplement: Supplementary file 33 — Figure EV3ABCD Source Data [file 44318_2024_242_MOESM33_ESM.zip › EV3ABCD/EV3A.tif]

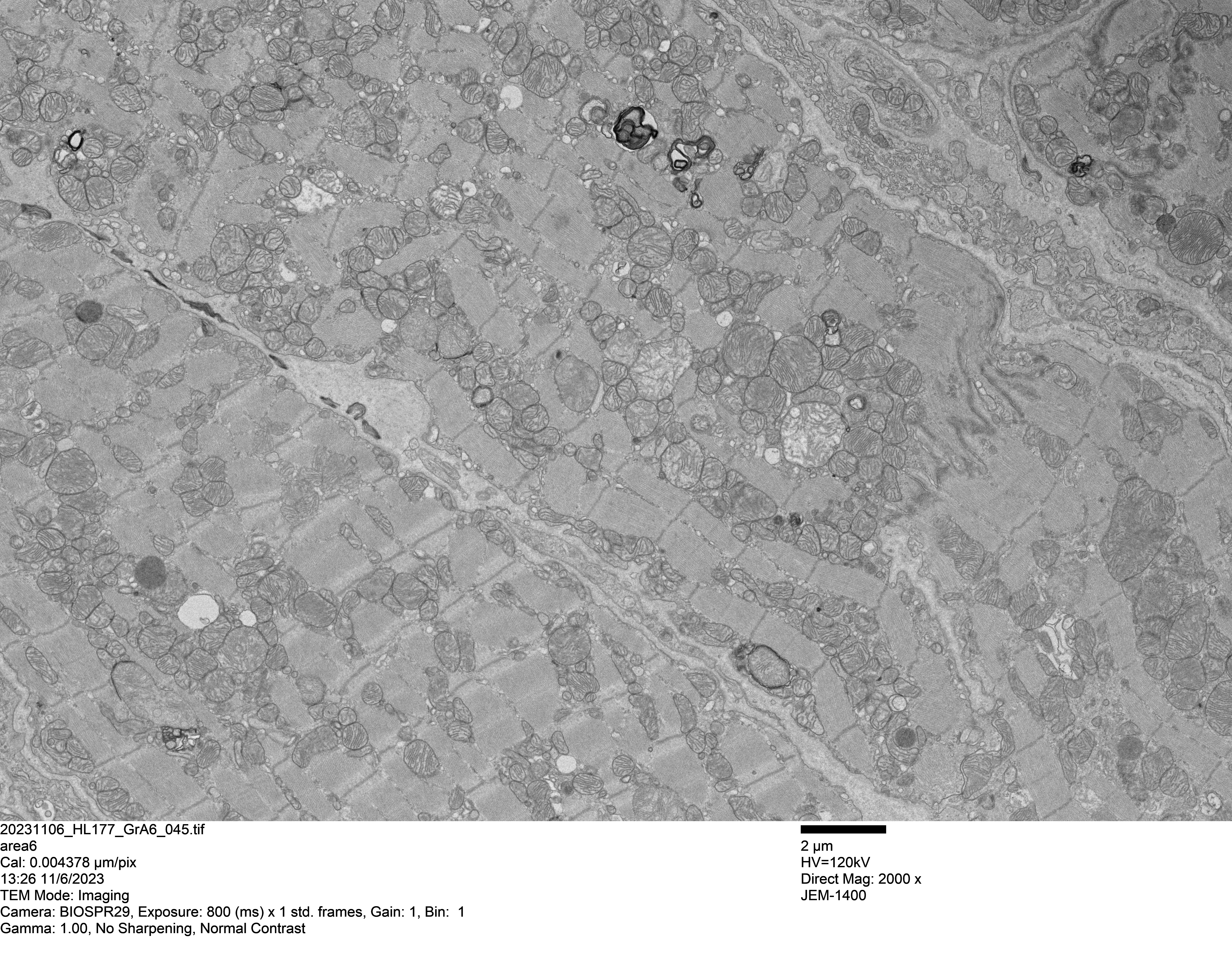

Supplement: Supplementary file 33 — Figure EV3ABCD Source Data [file 44318_2024_242_MOESM33_ESM.zip › EV3ABCD/EV3B.tif]

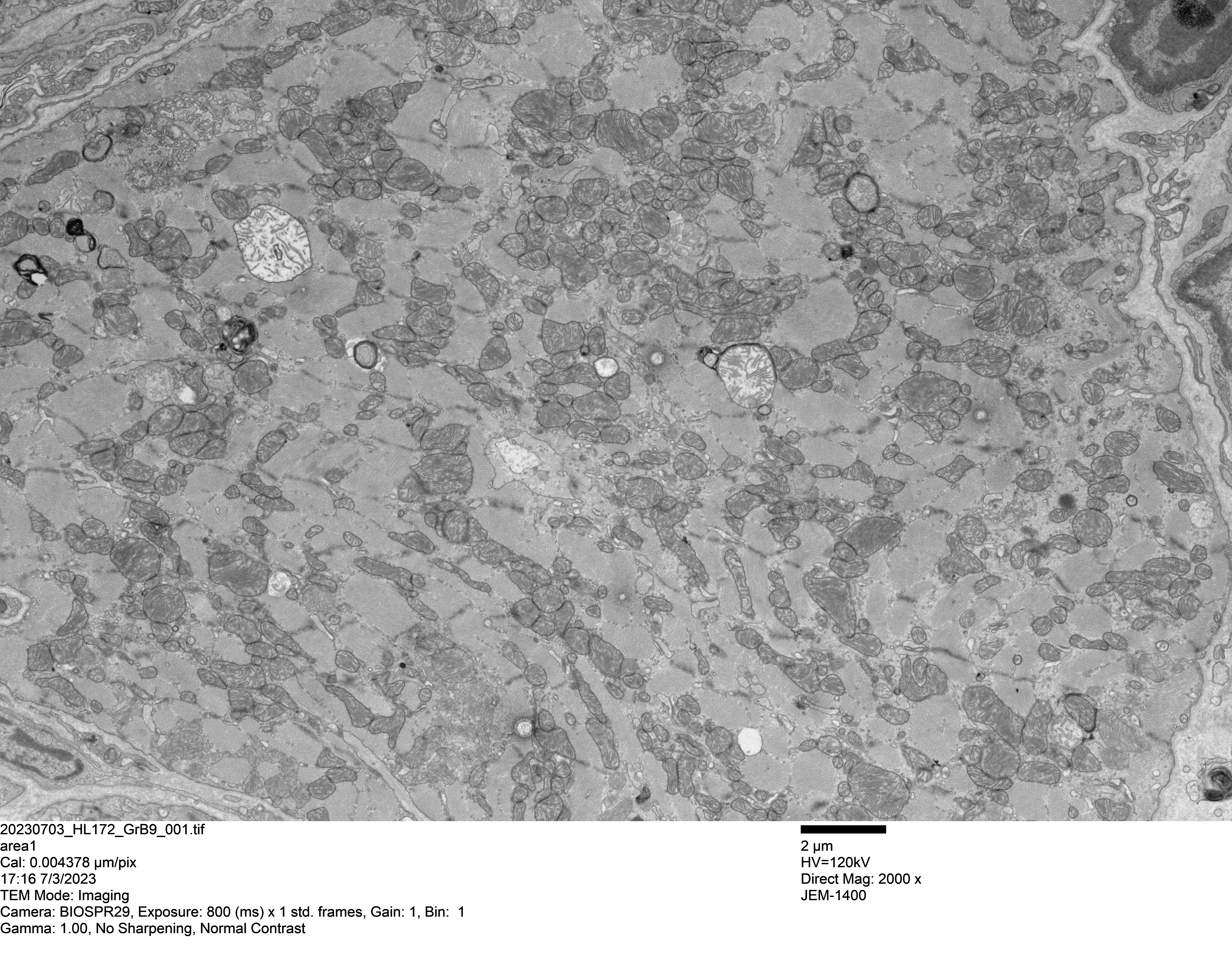

Supplement: Supplementary file 33 — Figure EV3ABCD Source Data [file 44318_2024_242_MOESM33_ESM.zip › EV3ABCD/EV3C.tif]

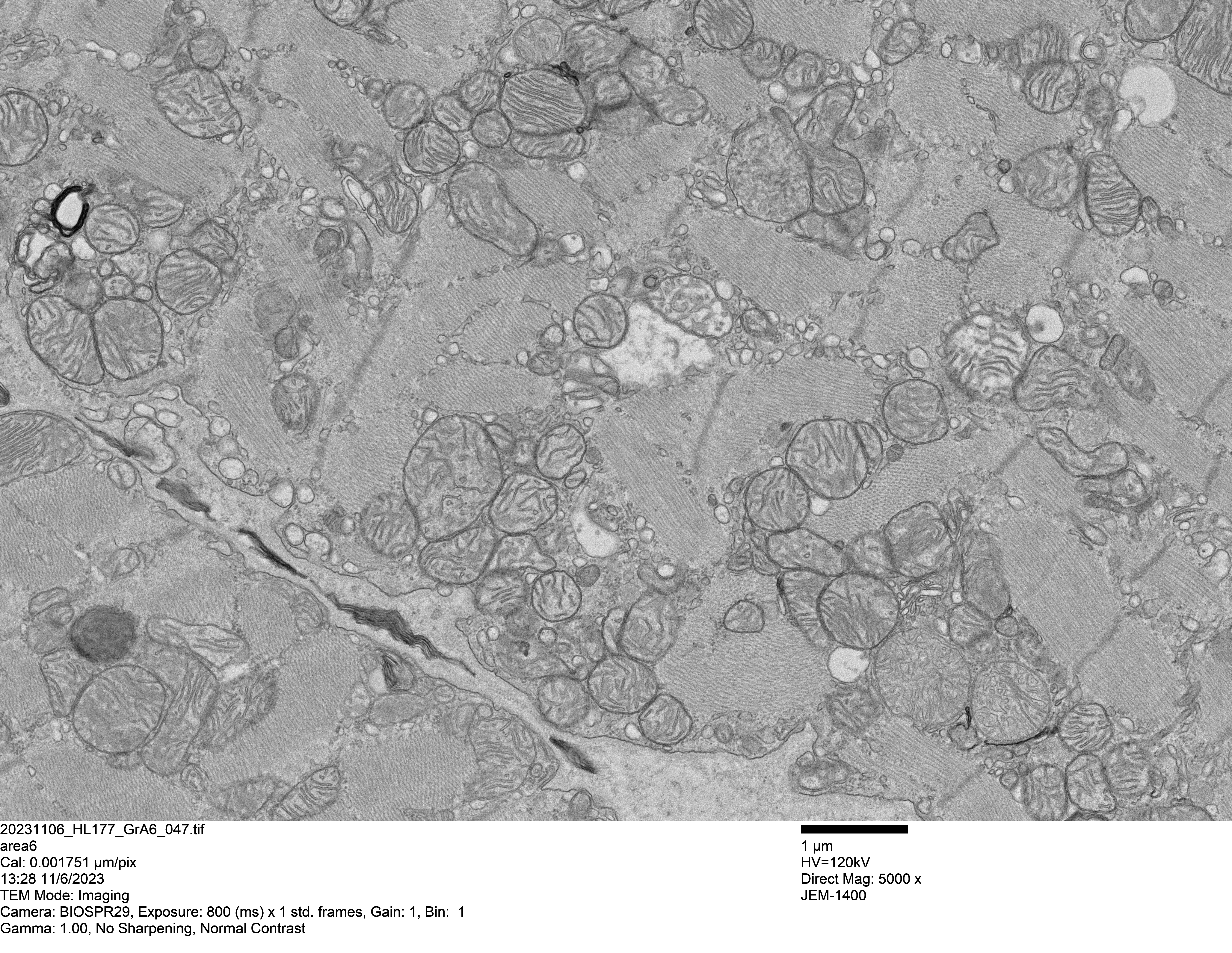

Supplement: Supplementary file 33 — Figure EV3ABCD Source Data [file 44318_2024_242_MOESM33_ESM.zip › EV3ABCD/EV3D.tif]

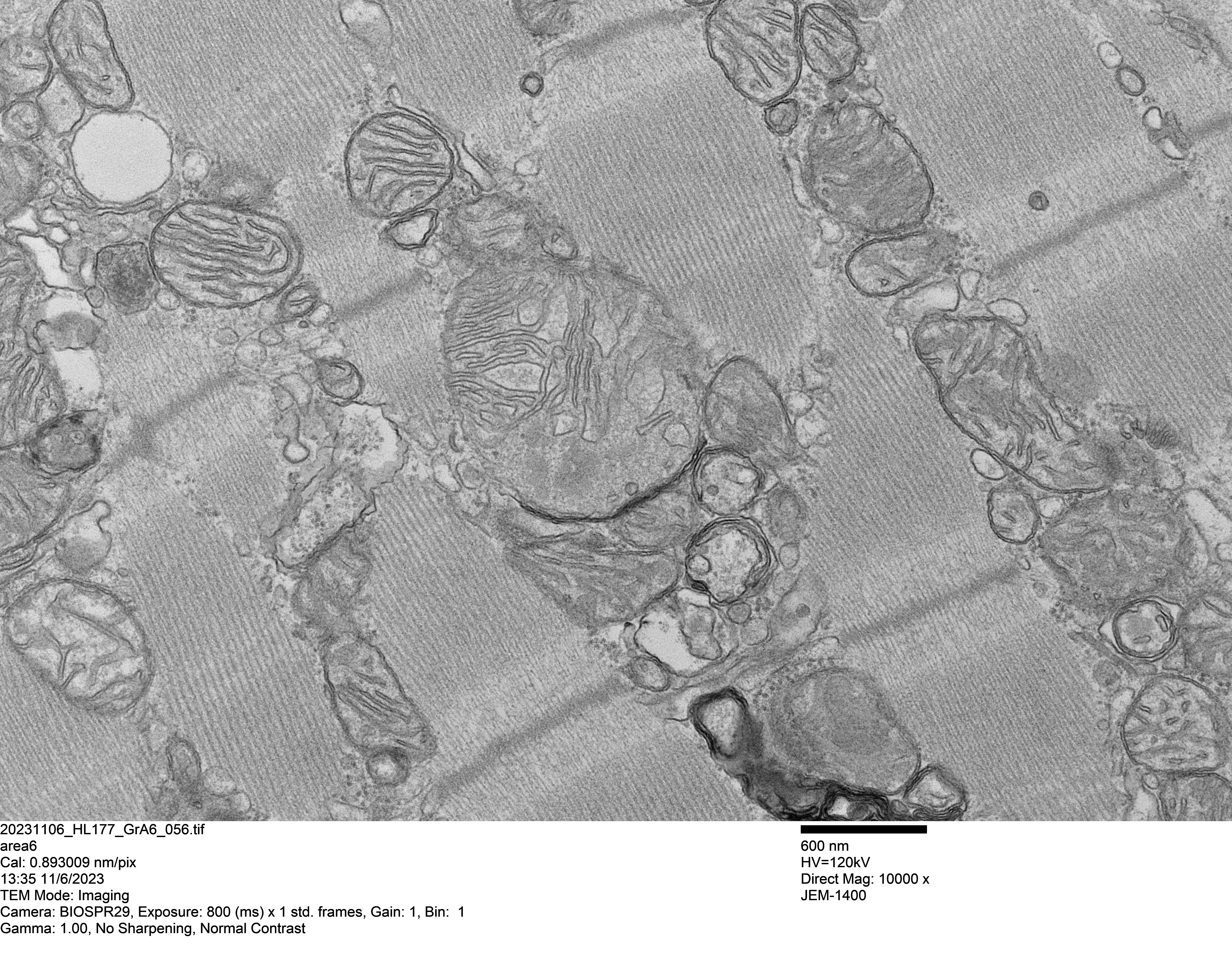

Supplement: Supplementary file 34 — Figure EV3E Source Data [file 44318_2024_242_MOESM34_ESM.zip › EV3E/EV3E_bottom_left.tif]

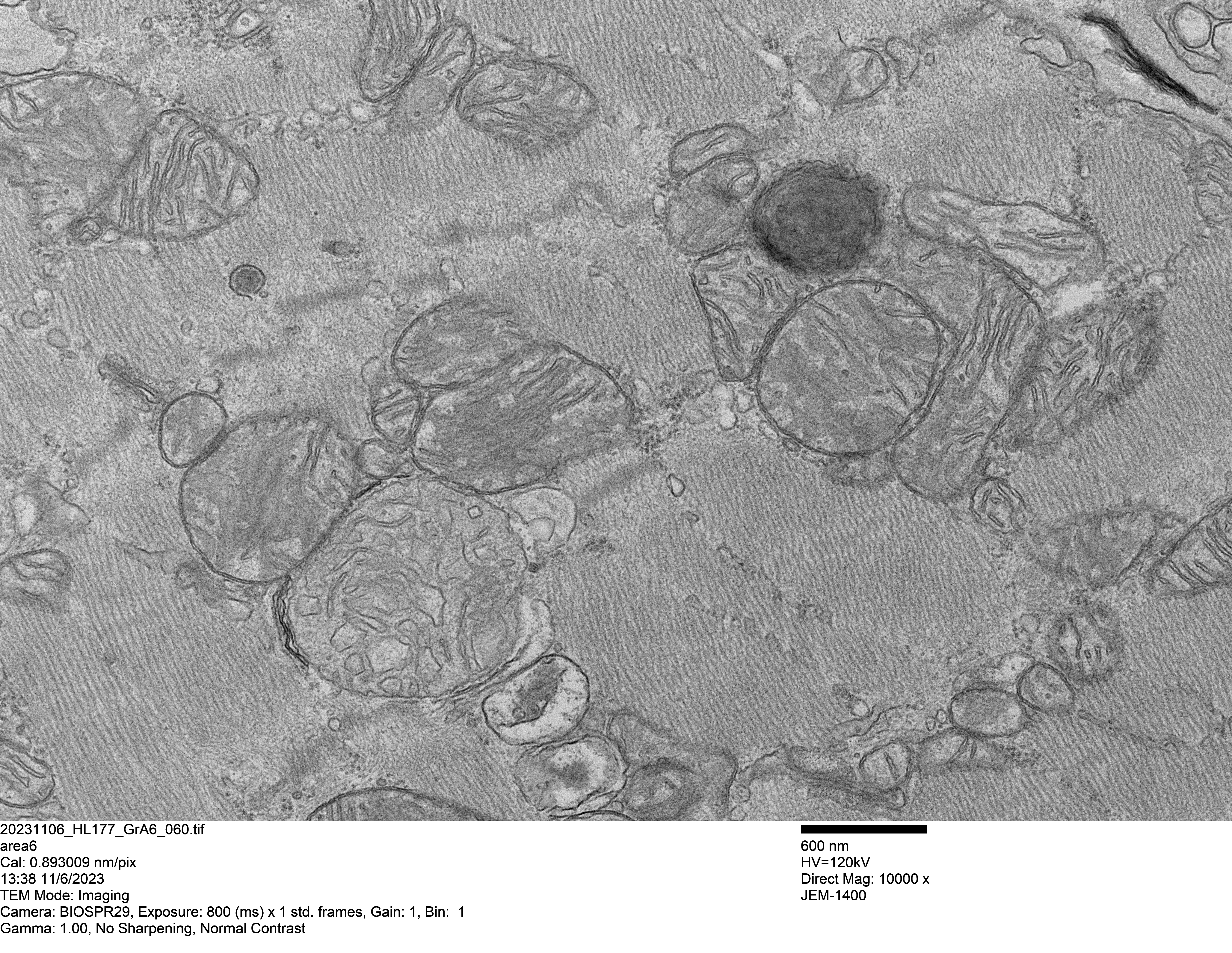

Supplement: Supplementary file 34 — Figure EV3E Source Data [file 44318_2024_242_MOESM34_ESM.zip › EV3E/EV3E_bottom_right.tif]

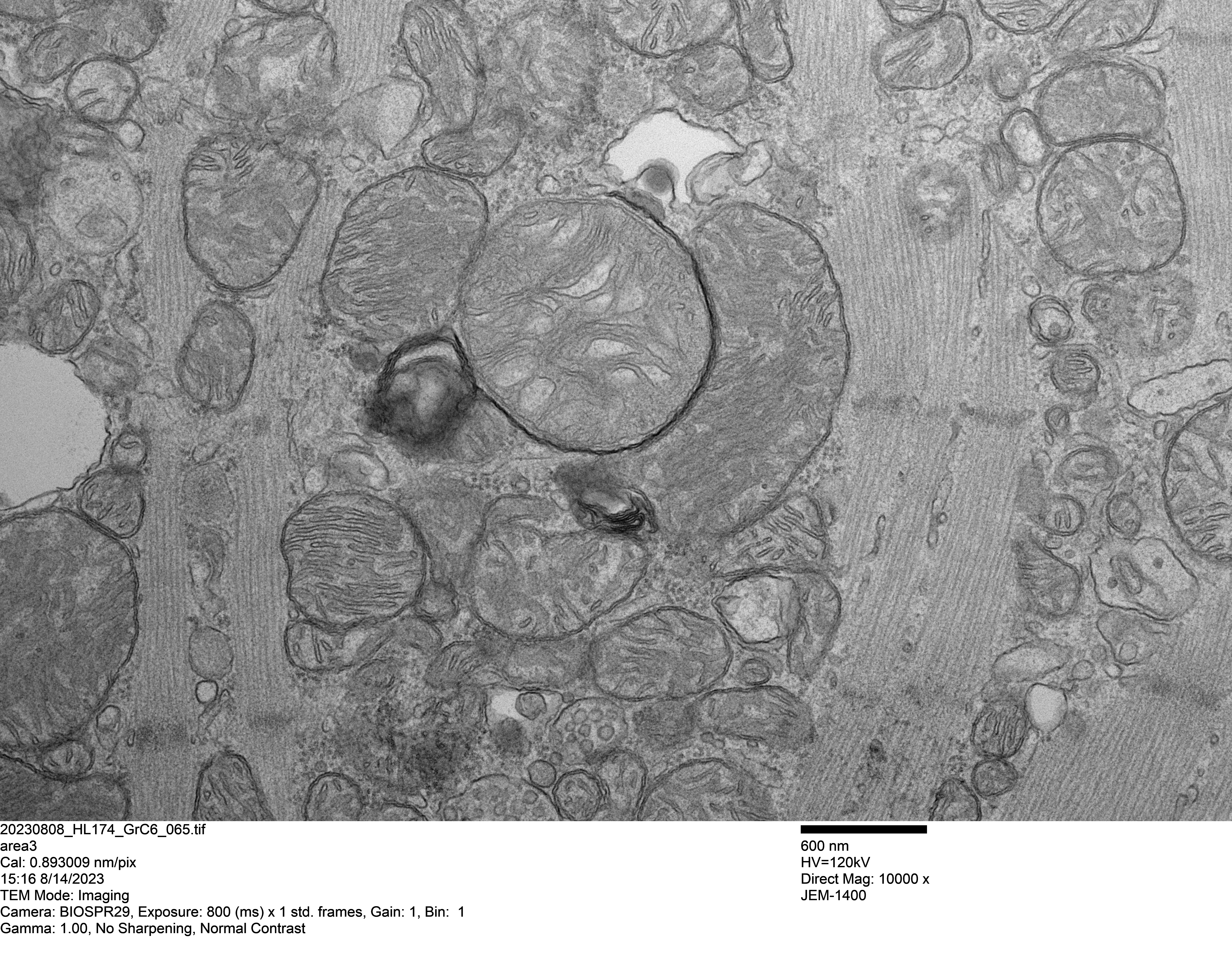

Supplement: Supplementary file 34 — Figure EV3E Source Data [file 44318_2024_242_MOESM34_ESM.zip › EV3E/EV3E_top_left.tif]

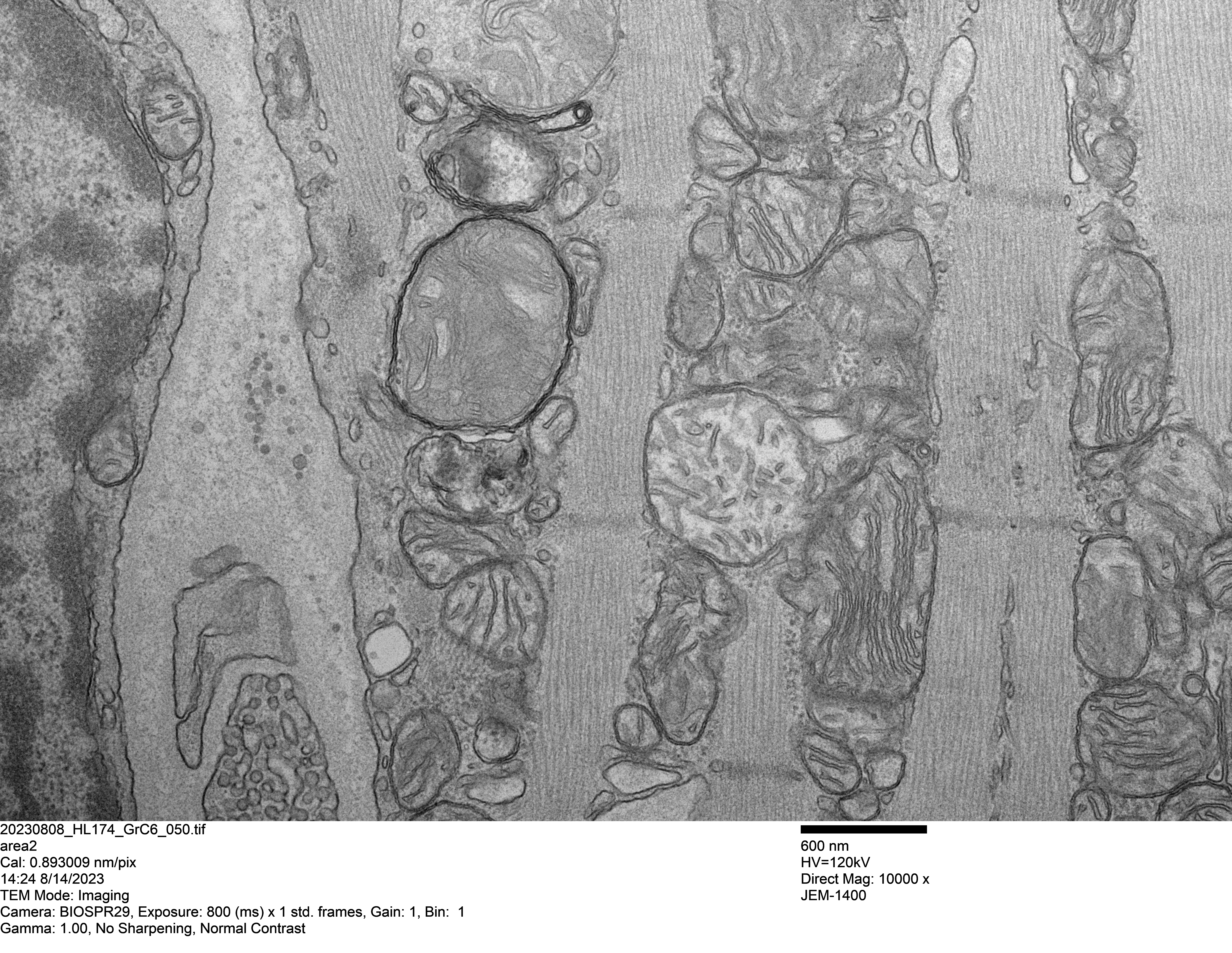

Supplement: Supplementary file 34 — Figure EV3E Source Data [file 44318_2024_242_MOESM34_ESM.zip › EV3E/EV3E_top_right.tif]

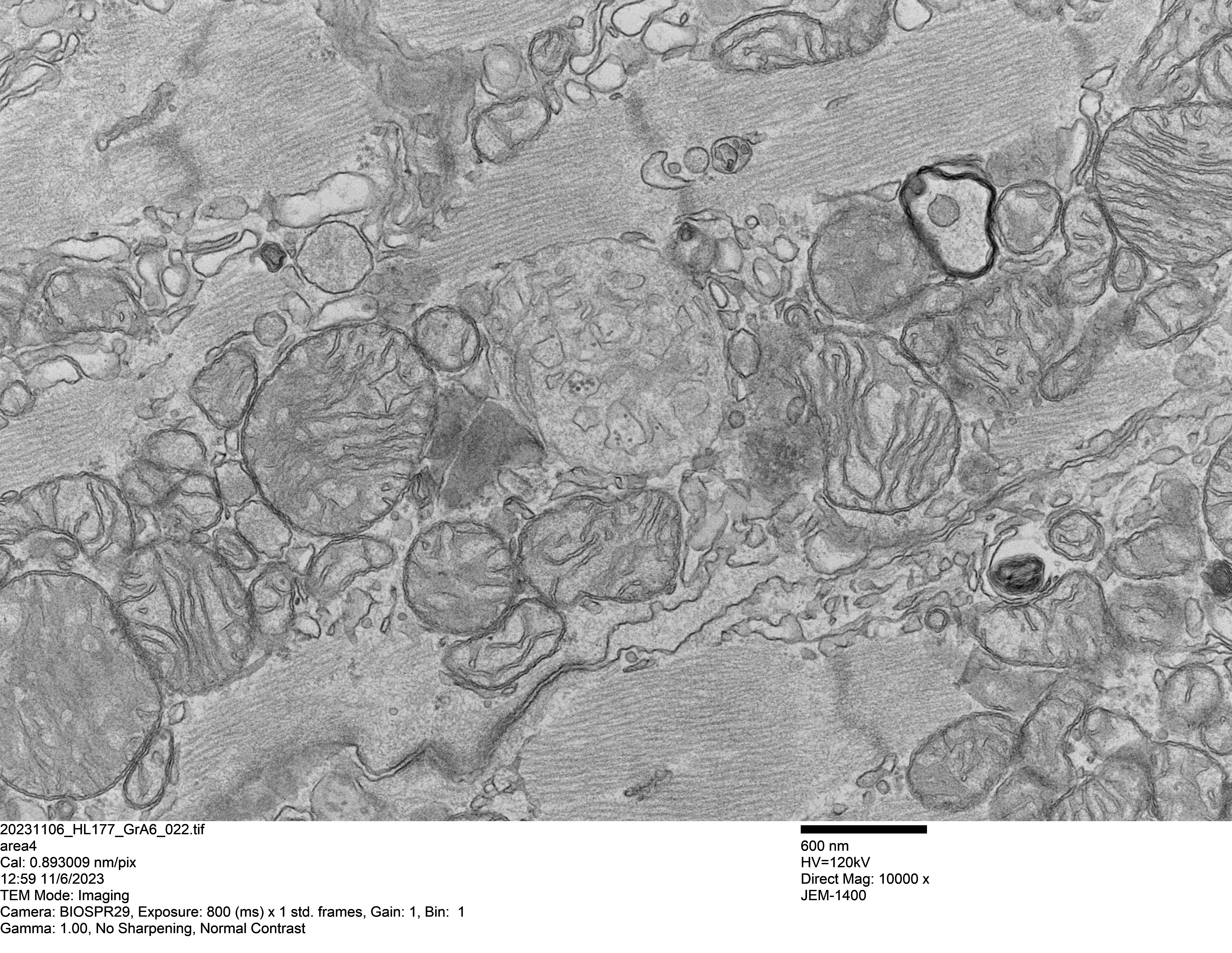

Supplement: Supplementary file 35 — Figure EV3F Source Data [file 44318_2024_242_MOESM35_ESM.zip › EV3F/EV3F_left.tif]

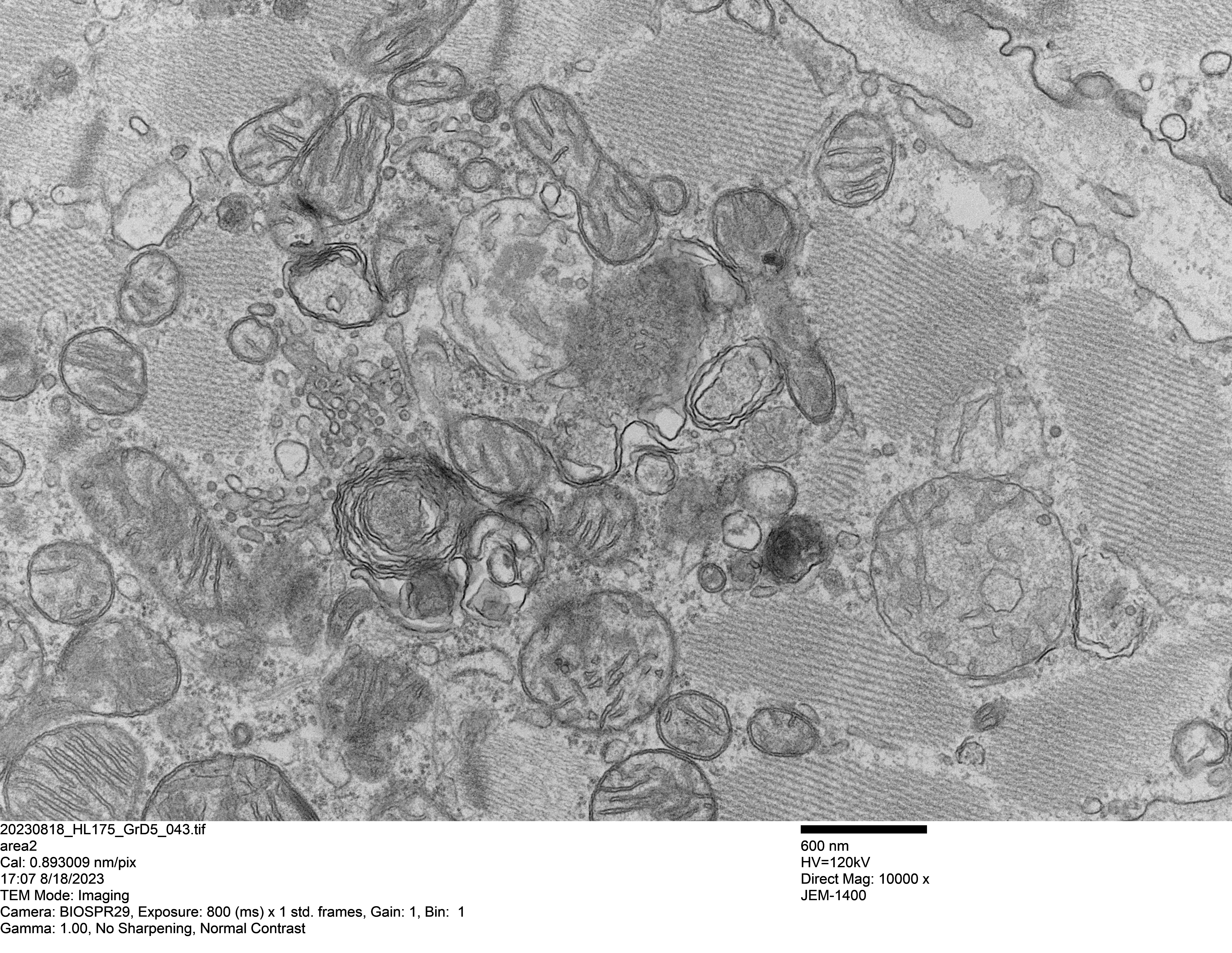

Supplement: Supplementary file 35 — Figure EV3F Source Data [file 44318_2024_242_MOESM35_ESM.zip › EV3F/EV3F_right.tif]

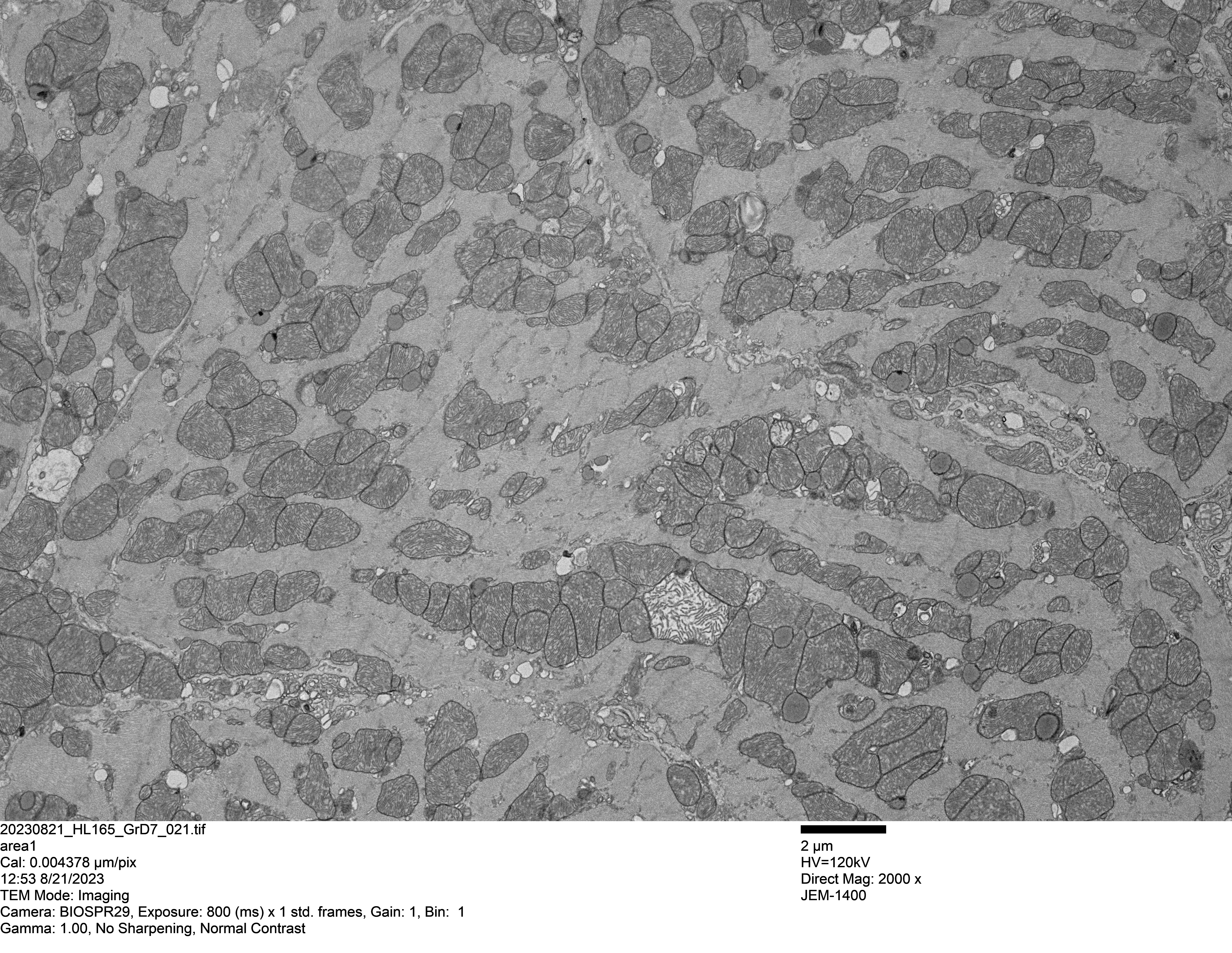

Supplement: Supplementary file 40 — Figure EV4ABCD Source Data [file 44318_2024_242_MOESM40_ESM.zip › EV4ABCD/EV4A.tif]

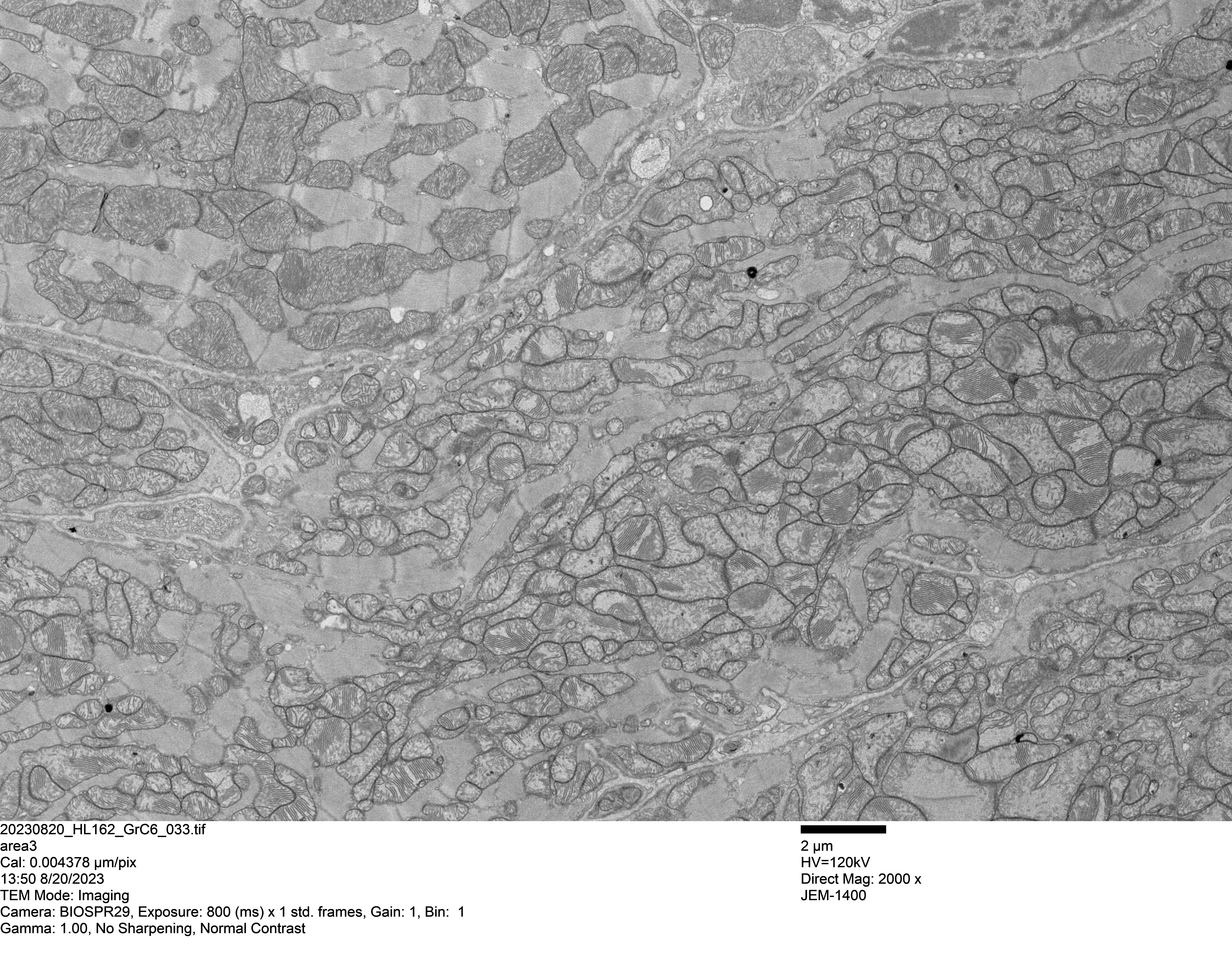

Supplement: Supplementary file 40 — Figure EV4ABCD Source Data [file 44318_2024_242_MOESM40_ESM.zip › EV4ABCD/EV4B.tif]

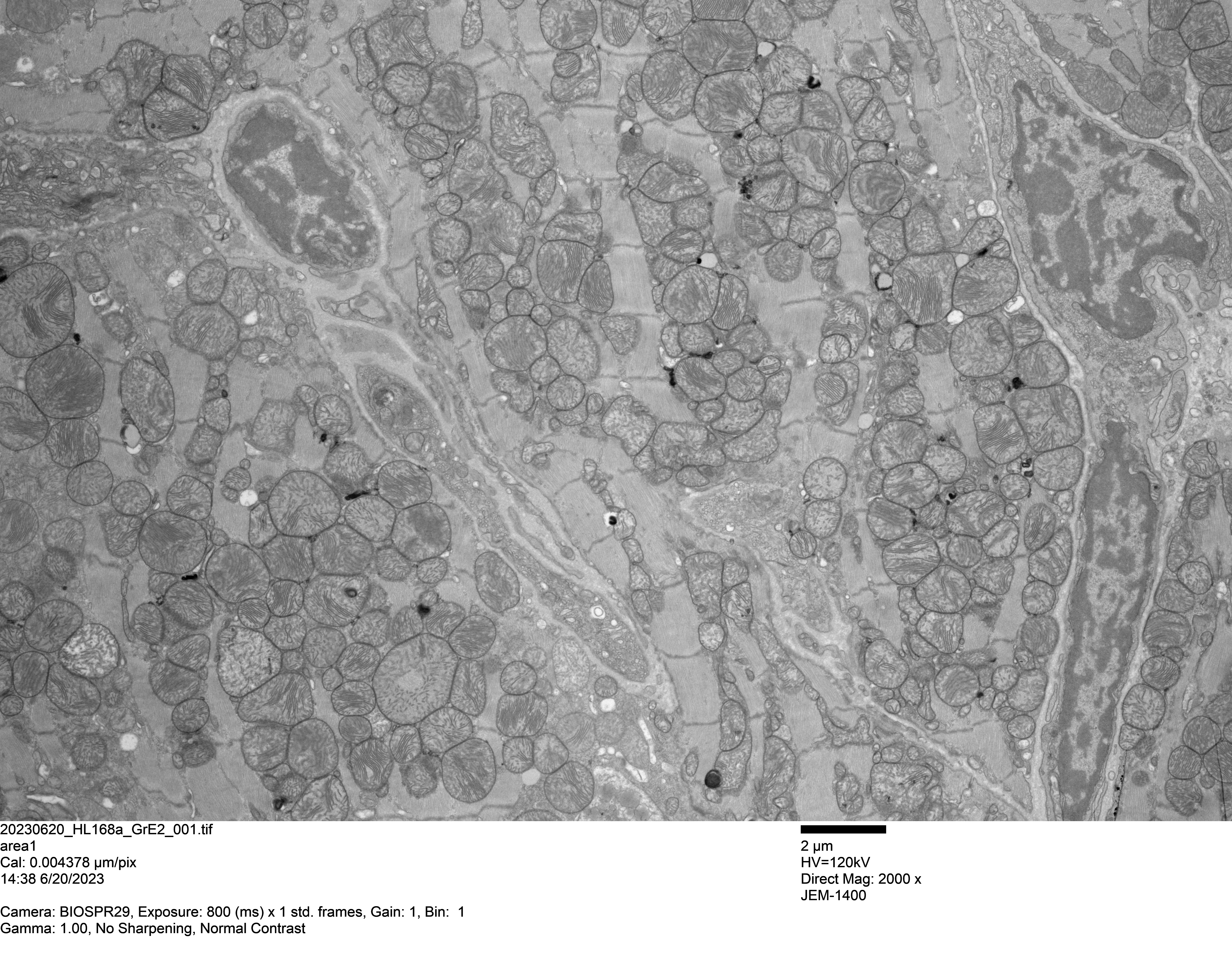

Supplement: Supplementary file 40 — Figure EV4ABCD Source Data [file 44318_2024_242_MOESM40_ESM.zip › EV4ABCD/EV4C.tif]

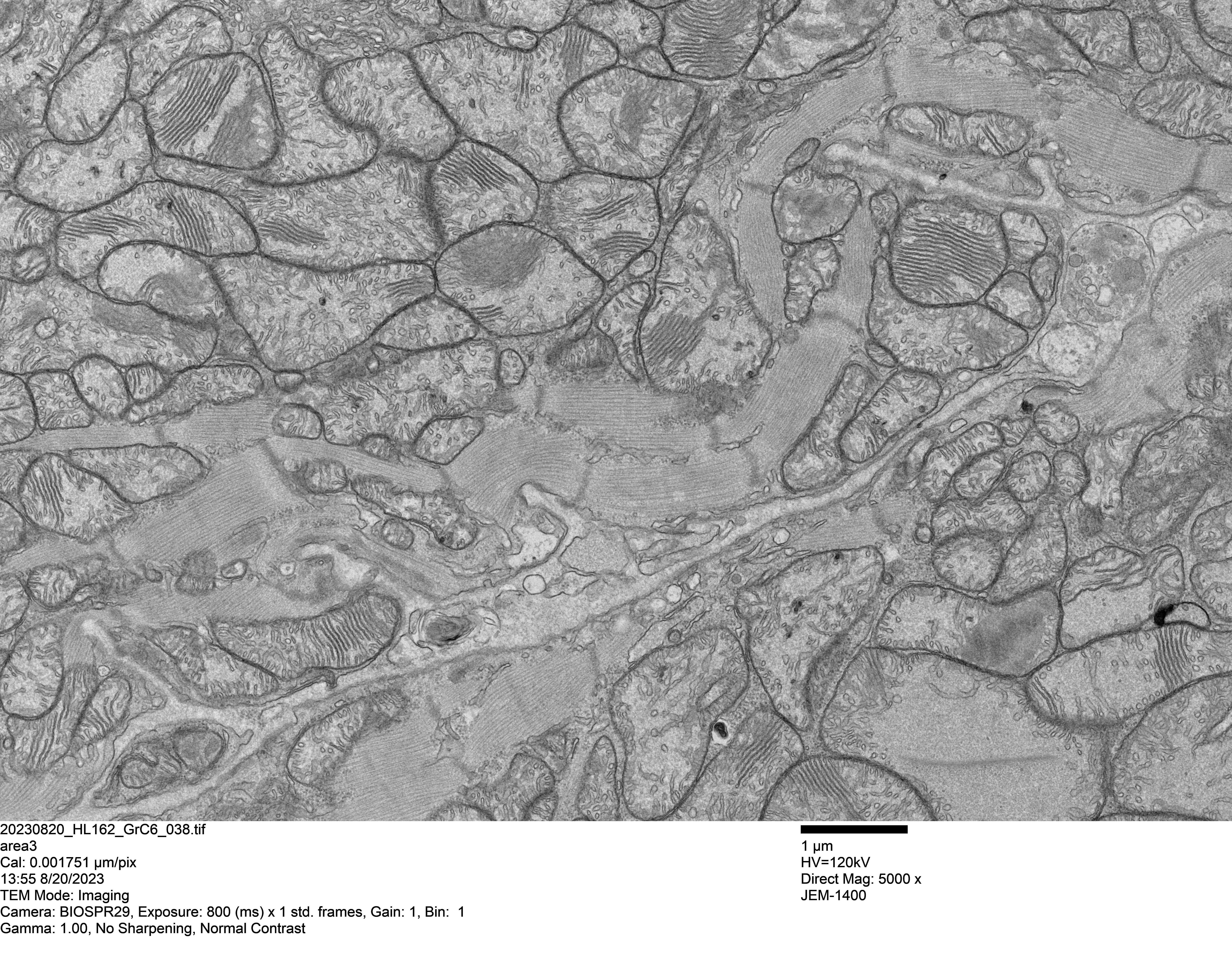

Supplement: Supplementary file 40 — Figure EV4ABCD Source Data [file 44318_2024_242_MOESM40_ESM.zip › EV4ABCD/EV4D.tif]

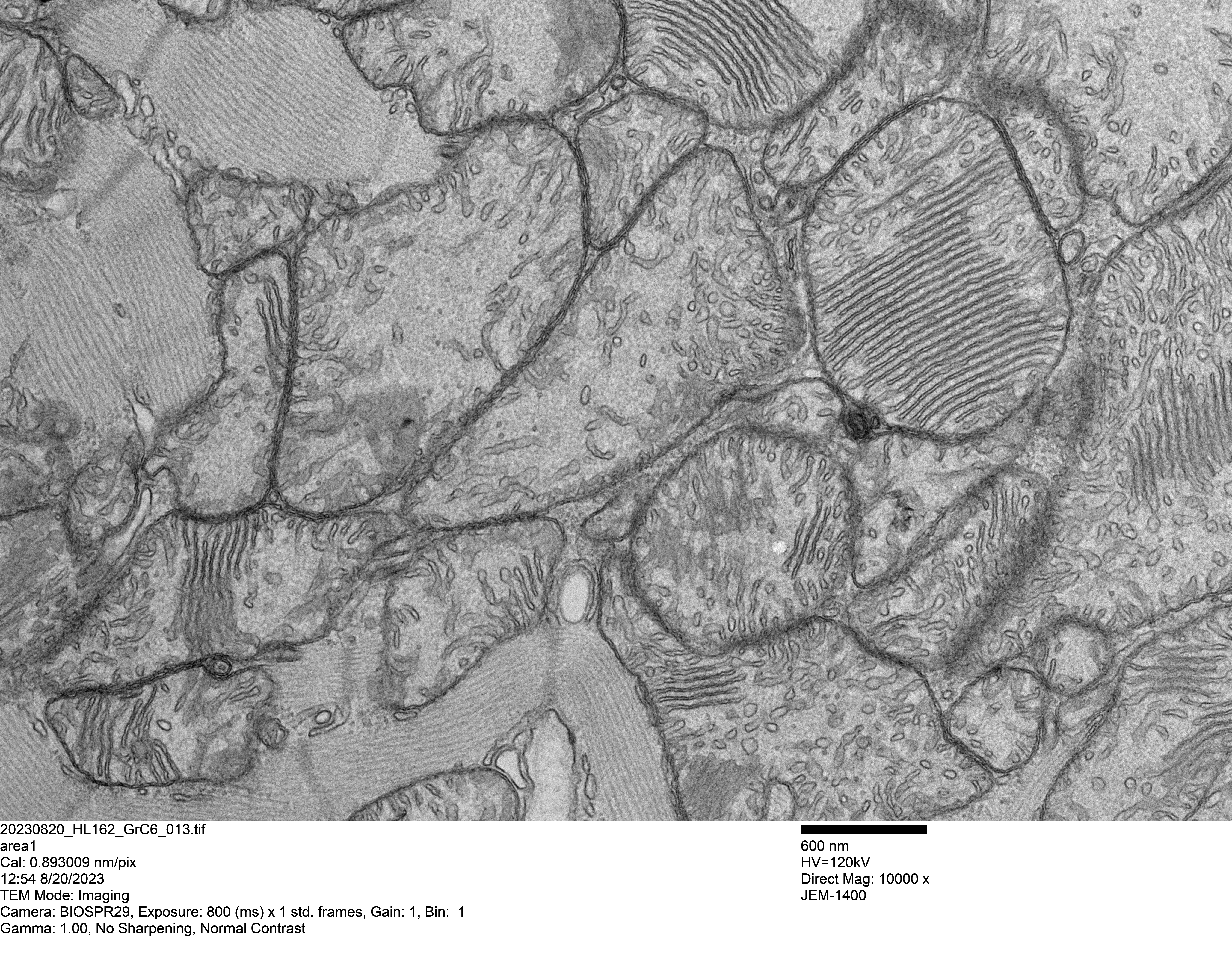

Supplement: Supplementary file 41 — Figure EV4E Source Data [file 44318_2024_242_MOESM41_ESM.zip › EV4E/EV4E_bottom_left.tif]

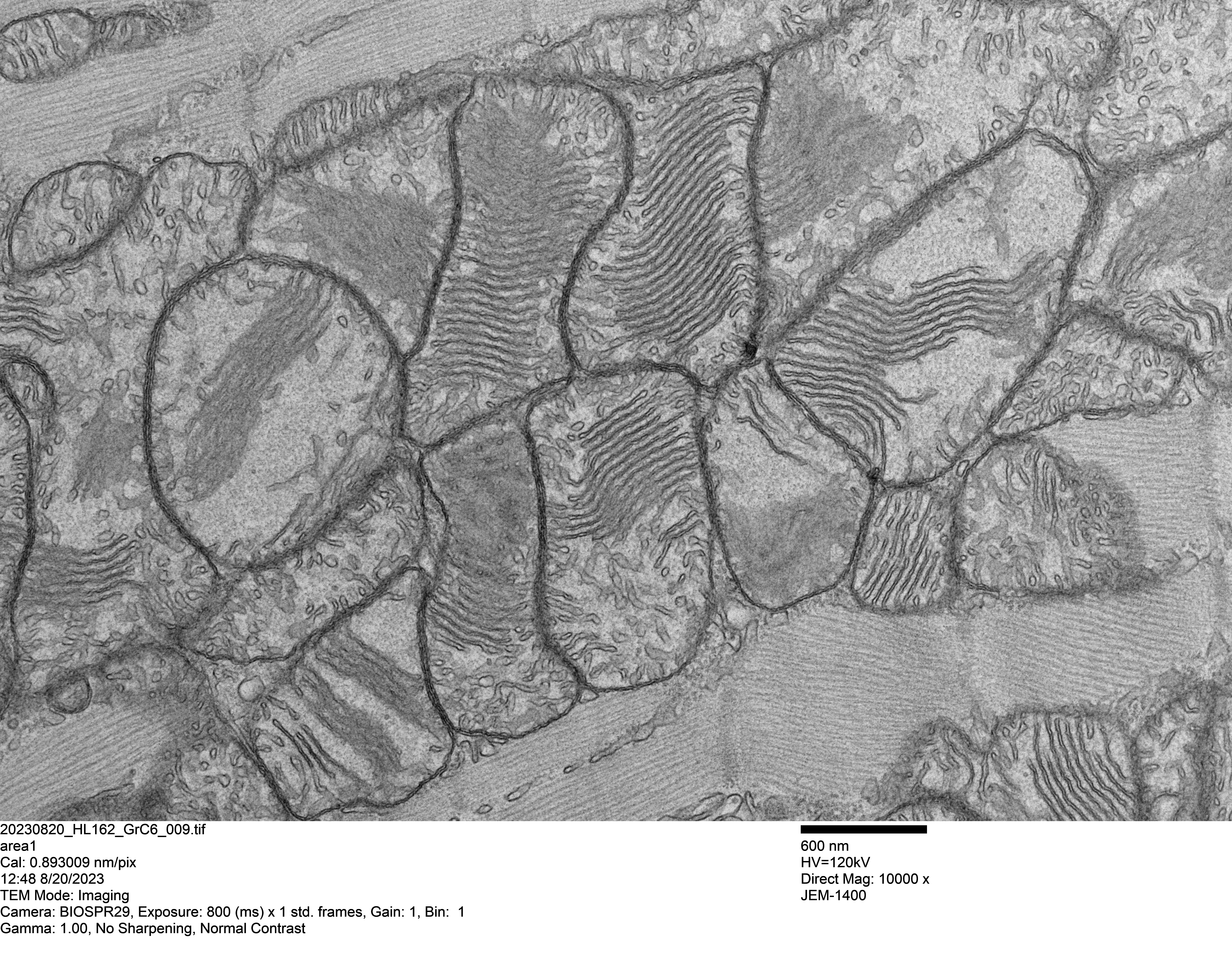

Supplement: Supplementary file 41 — Figure EV4E Source Data [file 44318_2024_242_MOESM41_ESM.zip › EV4E/EV4E_top_and_bottom_right.tif]

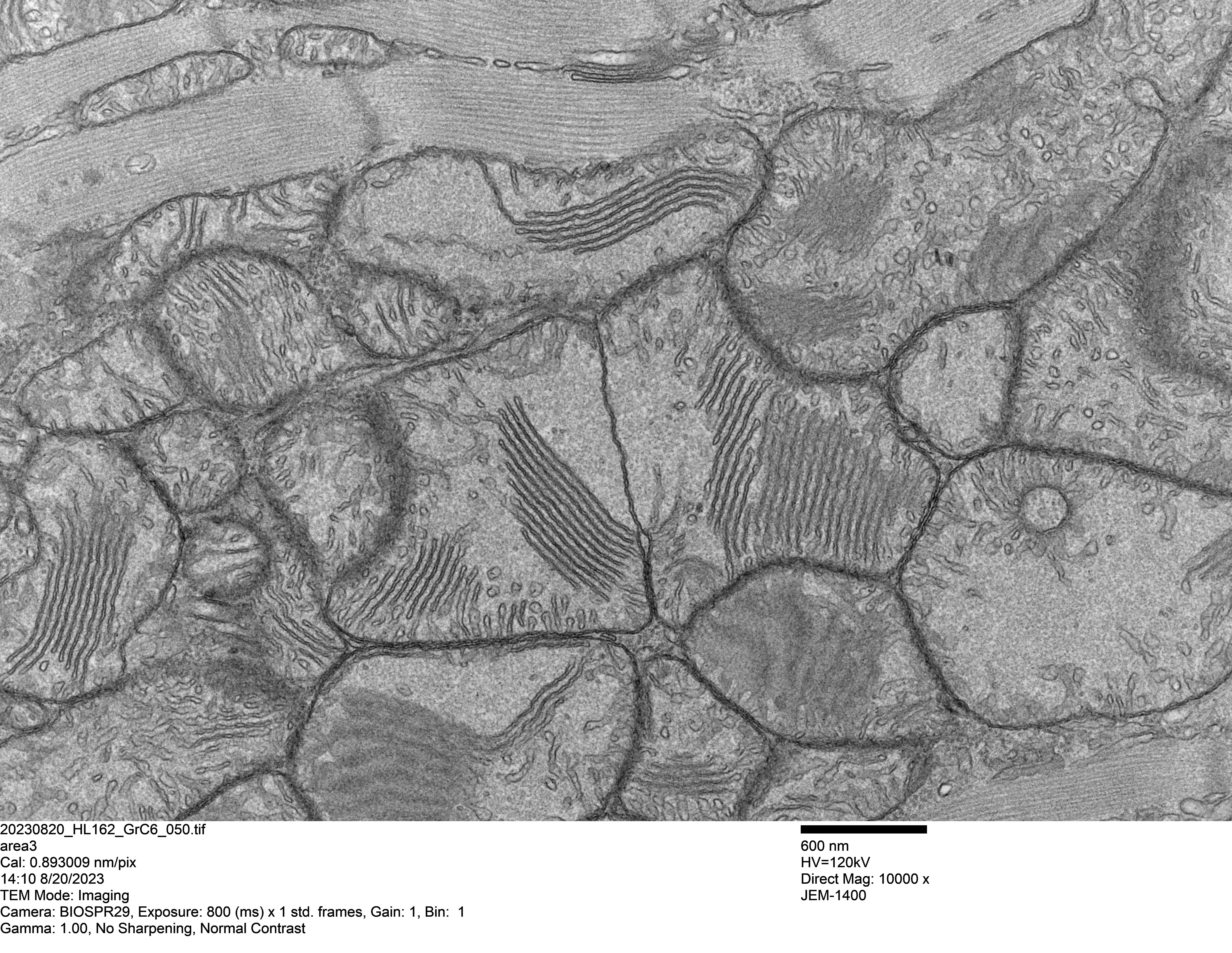

Supplement: Supplementary file 41 — Figure EV4E Source Data [file 44318_2024_242_MOESM41_ESM.zip › EV4E/EV4E_top_right.tif]

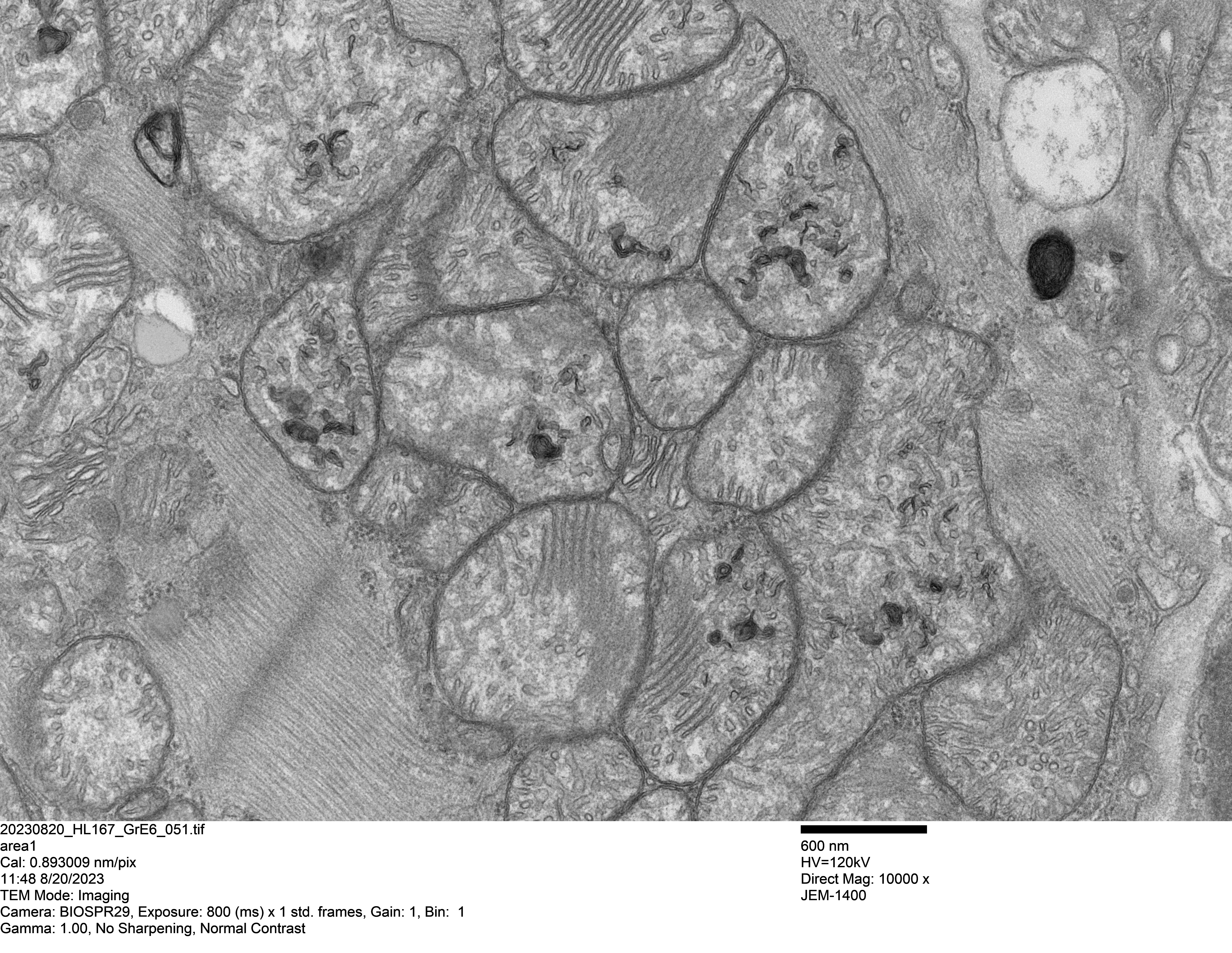

Supplement: Supplementary file 42 — Figure EV4F Source Data [file 44318_2024_242_MOESM42_ESM.zip › EV4F/EV4F_bottom_right.tif]
