# Supplementary figures and images for: DELE1 maintains muscle proteostasis to promote growth and survival in mitochondrial myopathy (part 2 of 2)
Source: EMBO J. 2024 Oct 8;43(22):5548–85. doi: 10.1038/s44318-024-00242-x (PMC11574132; doi:10.1038/s44318-024-00242-x)

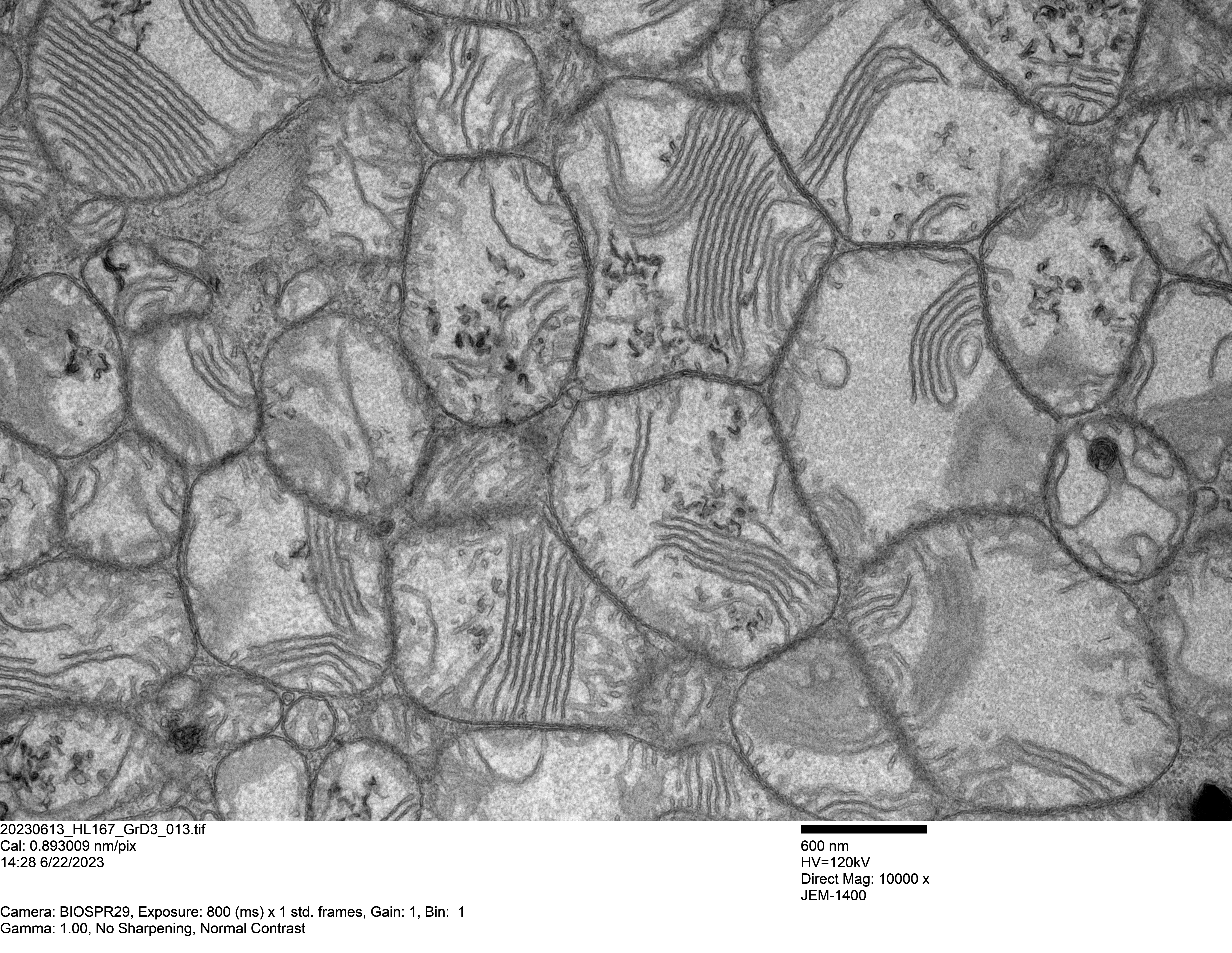

Supplement: Supplementary file 42 — Figure EV4F Source Data [file 44318_2024_242_MOESM42_ESM.zip › EV4F/EV4F_top_and_bottom_left.tif]

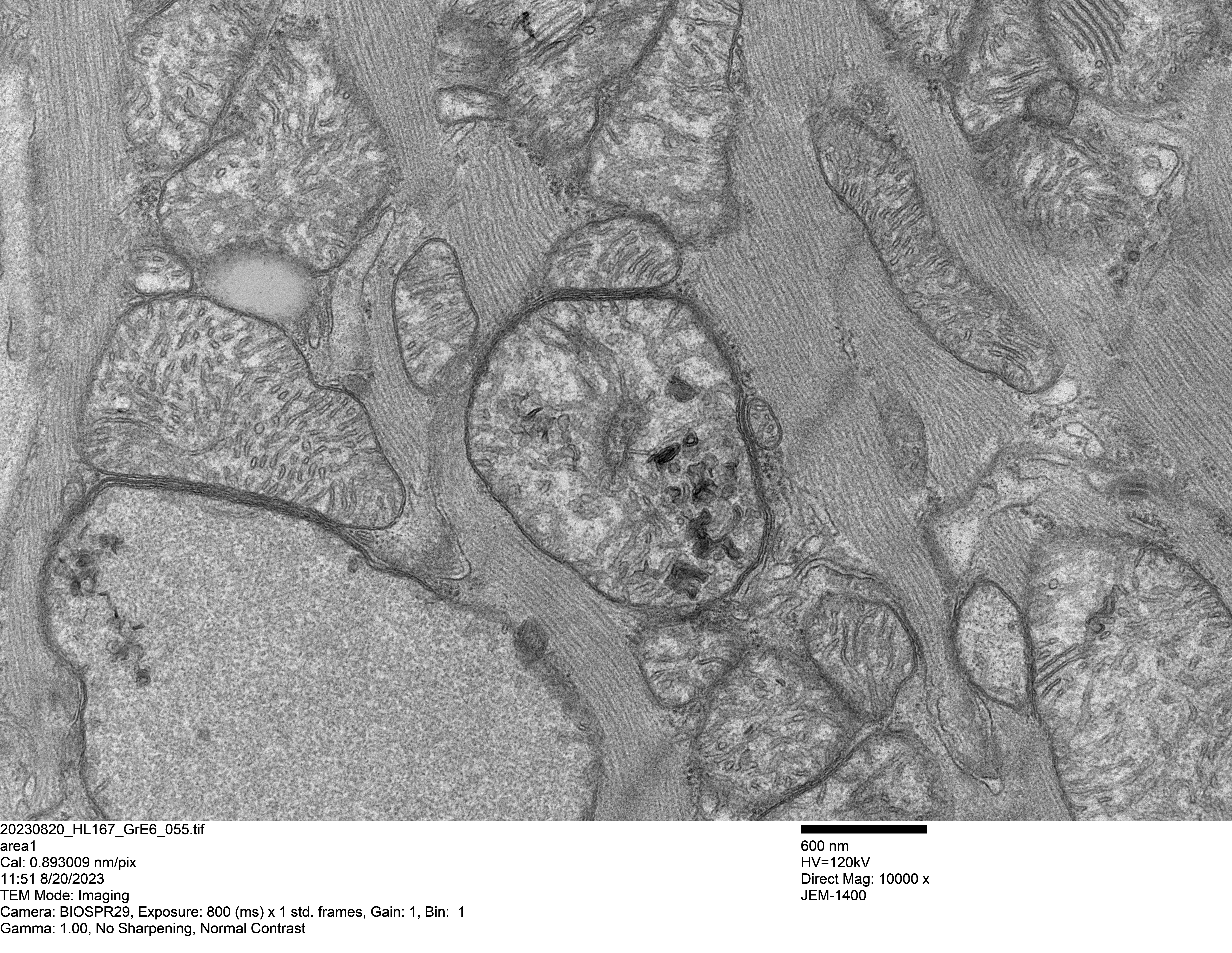

Supplement: Supplementary file 42 — Figure EV4F Source Data [file 44318_2024_242_MOESM42_ESM.zip › EV4F/EV4F_top_right.tif]
