# Supplementary material for: DELE1 maintains muscle proteostasis to promote growth and survival in mitochondrial myopathy
Source: EMBO J. 2024 Oct 8;43(22):5548–85. doi: 10.1038/s44318-024-00242-x (PMC11574132; doi:10.1038/s44318-024-00242-x)
Supplement: Supplementary file 43 — Expanded View Figures [file 44318_2024_242_MOESM43_ESM.pdf]

Expanded View Figures

Figure EV1. TEM of myocardium and ultrastructural features of mitochondria in C10 G58R on *Dele1*$^{+/-}$ and *Dele1* KO backgrounds.

(**A**) Kernel density plots showing distribution of mitochondrial areas for indicated genotypes, measured from TEM images of heart mitochondria. Median values and, in parentheses, interquartile ranges are reported adjacent to curves. $N = 2$ animals per genotype except for *Tfam* mKO; *Dele1* KO, where only 1 animal was available. >600 mitochondria were measured per animal. *P* value was <0.0001. Bar and error bars represent mean and SD, respectively. (**B**) Bar graph comparing the areas of segmented and non-segmented types of electrolucent mitochondria that were obtained from analysis of C10 G58R animals and littermates in (**A**). Statistics were performed using Mann–Whitney test, as the data had a non-parametric distribution. **** indicates $p \le 0.0001$. (**C–E**) Representative TEM images acquired at 2000× direct magnification show areas of myocardium of indicated genotype used for analysis of mitochondria. Scale bar = 5 µm. (**F**) Image of the subarea boxed yellow in (**D**), acquired at 5000× direct magnification and representative of the images used to quantify ultrastructural features of mitochondria detailed in Fig. 3. Scale bar = 2.5 µm. (**G**) Examples of inclusions observed in C10 G58R mutant mitochondria (black arrows). Scale bar = 500 nm. (**H**) Examples of two types of electrolucent mitochondria characterized by an enlarged matrix area absent of electron-dense substance and fewer cristae. (Top) A uniformly electrolucent mitochondrion. (Bottom) a segmented mitochondrion that has an electrolucent part (white arrow) separated from a portion of normal-looking matrix and cristae by a cut-through cristae. Open black arrowhead indicates the junction between electrolucent and normal portions of the segmented mitochondria. Scale bar = 500 nm. Please note: mitochondrion in (**H**, bottom) also appears in bottom right corner of image (**J**, top). (**I**) Mitochondria that are fully wrapped by electron-dense phagosome membranes (black arrows). Scale bar = 500 nm. (**J**) Mitochondria with ruptured OMMs. Open white arrowheads indicate sites where the intact IMM is visible, but OMM is absent. Scale bar = 500 nm. Please note: mitochondrion in image (**H**, bottom) also appears in the lower right corner of (**J**, top). Source data are available online for this figure.

This is a U.S. Government work and not under copyright protection in the US; foreign copyright protection may apply

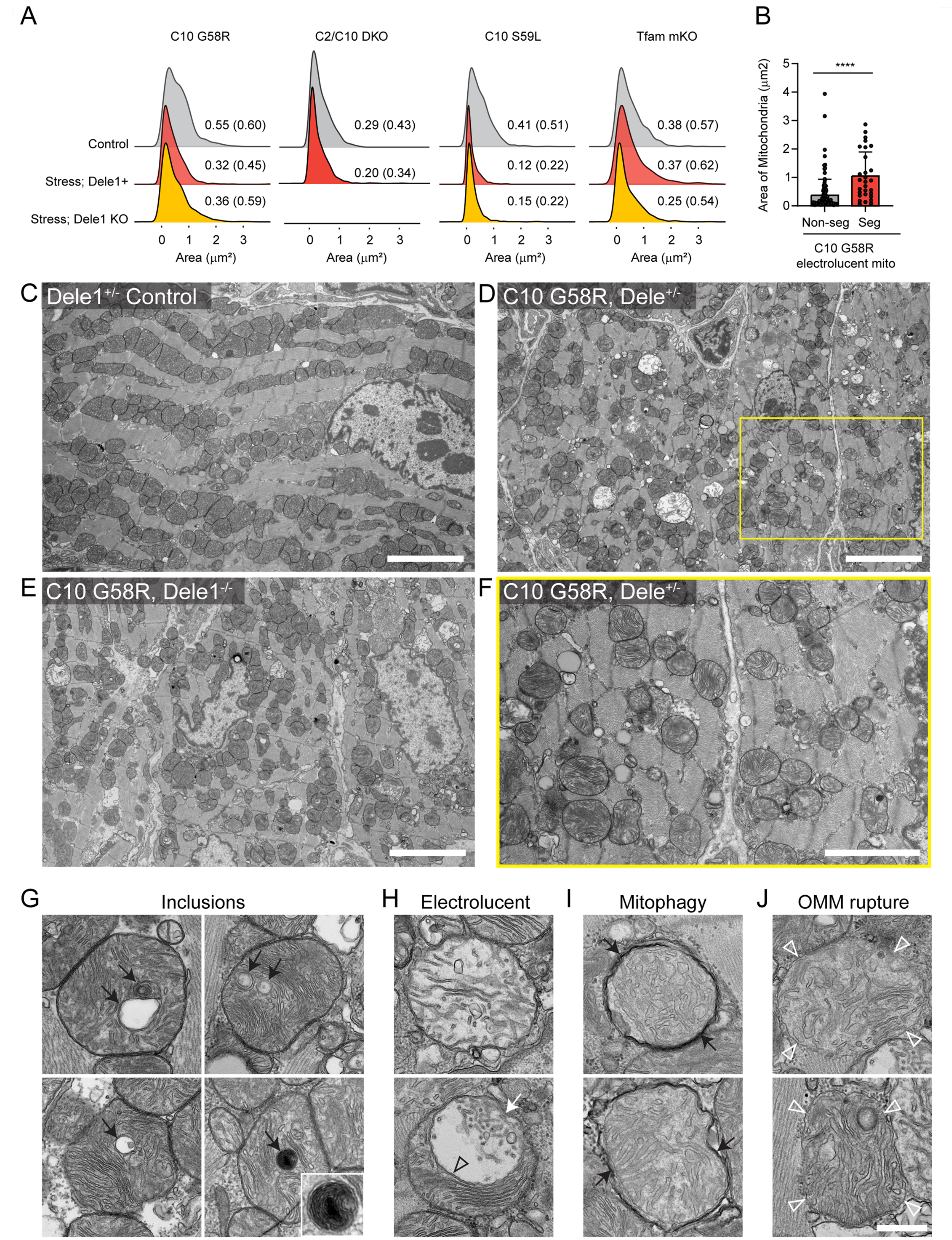

 This is a U.S. Government work and not under copyright protection in the US; foreign copyright protection may apply

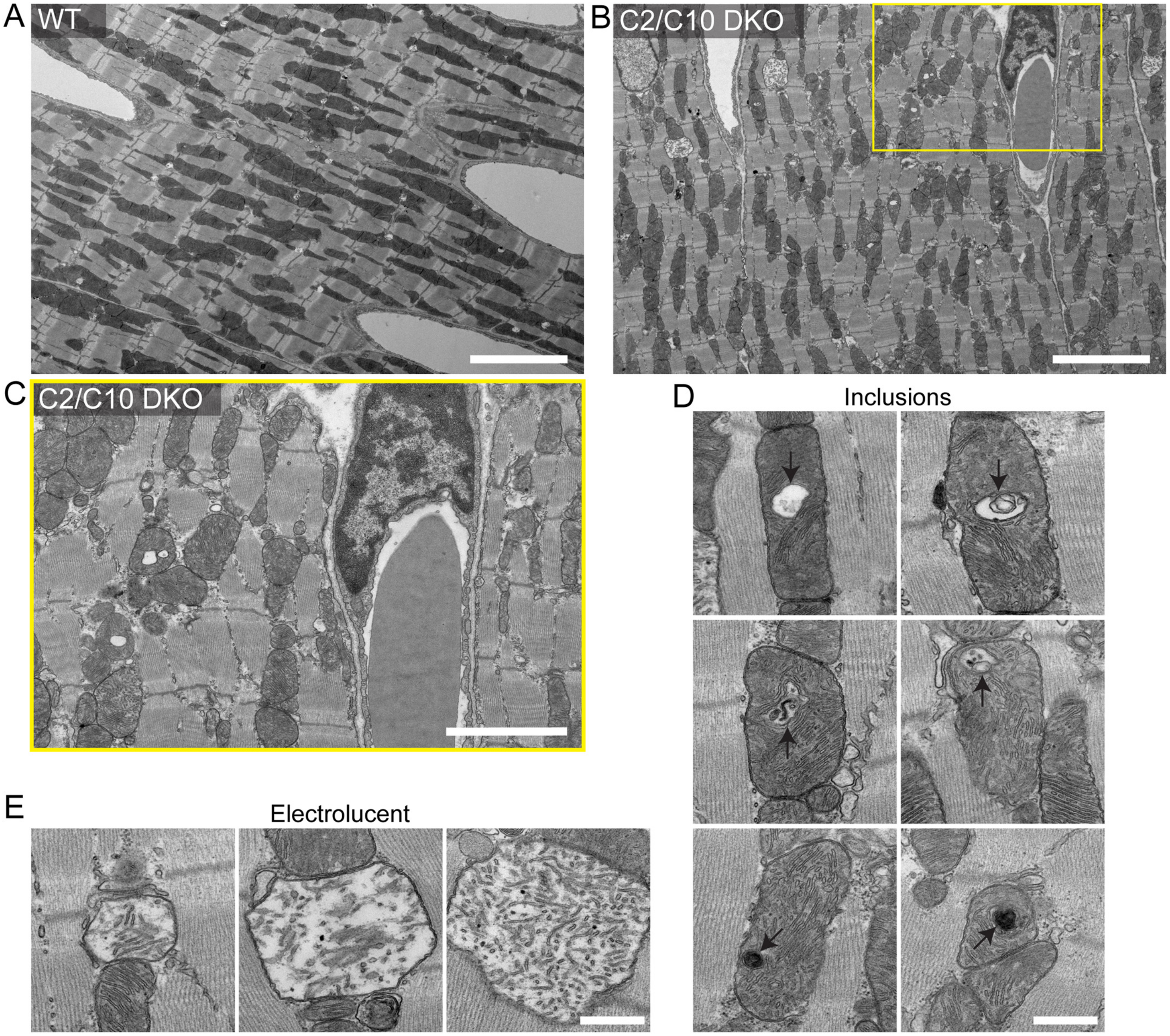

Figure EV2. TEM of myocardium and ultrastructural features of mitochondria in C2/C10 DKO.

(**A**, **B**) Representative TEM images acquired at 2000× direct magnification show areas of myocardium of indicated genotype used for analysis of mitochondria. Scale bar = 5 µm. (**C**) Image of the subarea boxed yellow in (**B**), acquired at 5000× direct magnification and representative of the images used to quantify ultrastructural features of mitochondria detailed in Fig. 3. Scale bar = 2.5 µm. (**D**) Examples of inclusions observed in C2/C10 DKO mitochondria (black arrows). Scale bar = 500 nm. (**E**) Examples of electrolucent mitochondria characterized by an enlarged matrix area absent of electron-dense substance and fewer cristae. Scale bar = 500 nm. Source data are available online for this figure.

This is a U.S. Government work and not under copyright protection in the US; foreign copyright protection may apply

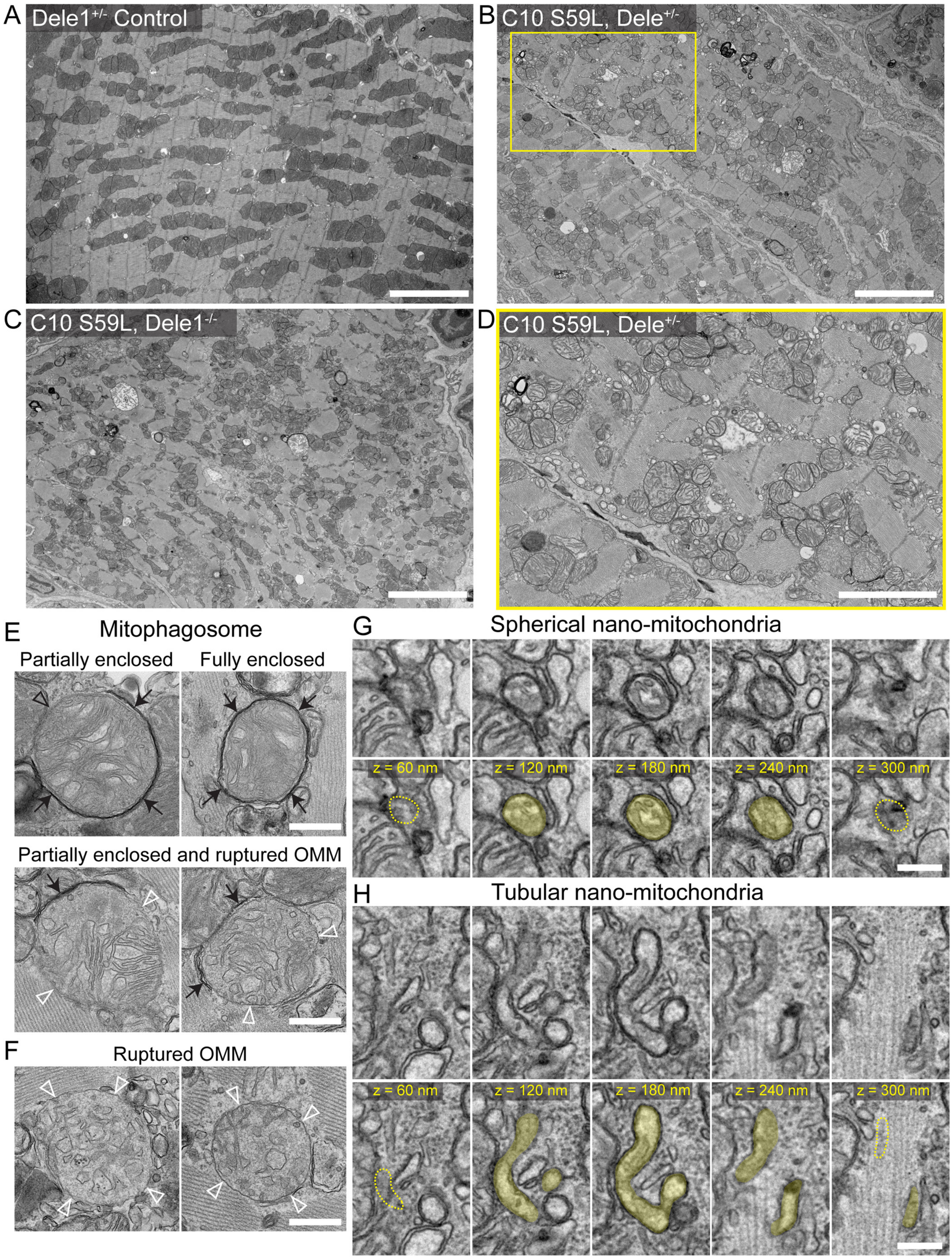

 This is a U.S. Government work and not under copyright protection in the US; foreign copyright protection may apply

Figure EV3. TEM of myocardium and ultrastructural features of mitochondria in C10 S59L on Dele1+/− and Dele1 KO backgrounds.

(**A–C**) Representative TEM images acquired at 2000× direct magnification show areas of myocardium of indicated genotype used for analysis of mitochondria. Scale bar = 5 µm. (**D**) Image of the subarea boxed yellow in (**B**), acquired at 5000× direct magnification and representative of images used to quantify ultrastructural features of mitochondria detailed in Fig. 3. Scale bar = 2.5 µm. (**E**) Examples of mitochondria that are partially or fully enclosed by electron-dense phagosome membranes (black arrows). Open black arrowhead indicates a portion of the mitochondria that is not enclosed. Partially enclosed mitochondria with ruptured OMMs were also observed. Open white arrowheads indicate sites where the intact IMM is visible, but the OMM is absent. Scale bar = 500 nm. (**F**) Examples of mitochondria with ruptured OMMs. Open white arrowheads indicate sites where the intact IMM is visible, but an OMM is absent. Scale bar = 500 nm. (**G**) Serial sections through a 250 nm diameter mitochondrion show that it is a spherical nano-mitochondrion spanning fewer than five 60-nm sections (< 300 nm in (Z)). Top row shows the five serial sections without colorization, bottom row shows the same serial sections with the nano-mitochondrion shaded yellow. Yellow dotted lines indicate absence of the mitochondrion in neighboring serial sections. Scale bar = 200 nm. (**H**) Five serial sections of 60-nm thickness show a 100 nm-wide tubule-shaped mitochondrion. Top row shows five serial sections through the tubular nano-mitochondrion, bottom row shows the same serial sections with the tubular nano-mitochondrion shaded yellow. The yellow dotted lines indicate absence of the mitochondrion in the neighboring section. Scale bar = 200 nm. Source data are available online for this figure.

This is a U.S. Government work and not under copyright protection in the US; foreign copyright protection may apply

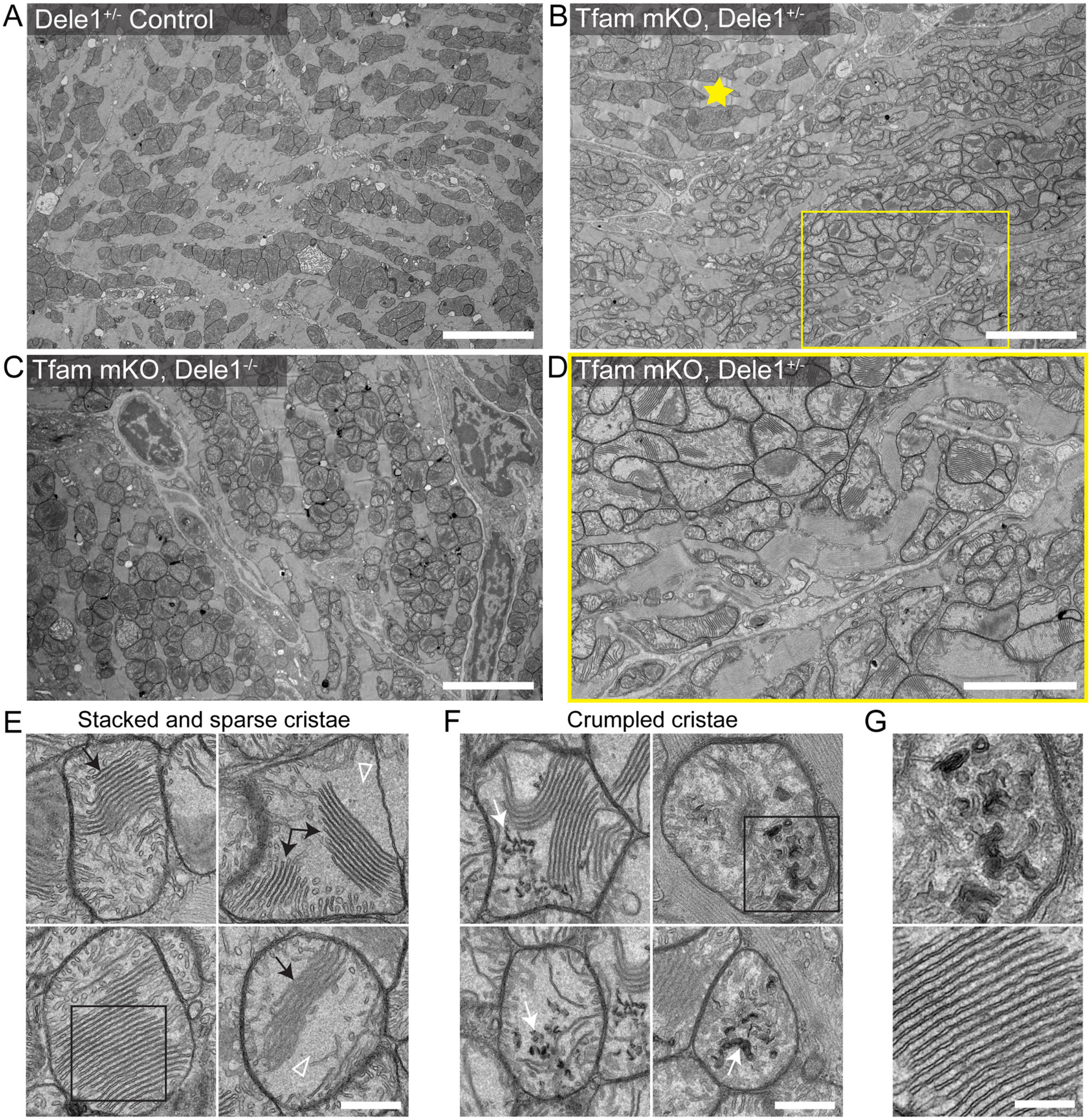

Figure EV4. TEM of myocardium and ultrastructural features of mitochondria in *Tfam* mKO on *Dele1*+/− and *Dele1* KO backgrounds.

(**A–C**) Representative TEM images acquired at 2000× direct magnification show areas of myocardium of indicated genotype used for analysis of mitochondria. Yellow star in (**B**) indicates a myocyte with milder structural phenotype compared to neighboring myocytes, illustrating the observed mosaicism of the phenotype. Scale bar = 5 µm. (**D**) Image of the subarea boxed yellow in (**B**), acquired at 5000× direct magnification and representative of images used to quantify ultrastructural features of mitochondria detailed in Fig. 3. Scale bar = 2.5 µm. (**E**) *Tfam* mKO mitochondria displayed populations of closely aligned "stacked" cristae (black arrows) and sparse areas filled with a granular matrix material and few cristae (open white arrowheads). Scale bar = 500 nm. The mitochondrion in the bottom left image is cropped from same cell as the mitochondrion shown in (Fig. 3F, middle). At least some Images of mitochondria are taken from the same cell. (**F**) Examples of crumpled cristae (white arrows) that occurred in *Tfam* mKO mitochondria. Scale bar = 500 nm. At least some Images of mitochondria are taken from the same cell. (**G**) This panel shows enlargements of images that are also shown in (Fig. EV4E, boxed area with "stacked cristae") and (Fig. EV4F, boxed area with "crumpled cristae"). Scale bar = 250 nm. Source data are available online for this figure.

 This is a U.S. Government work and not under copyright protection in the US; foreign copyright protection may apply

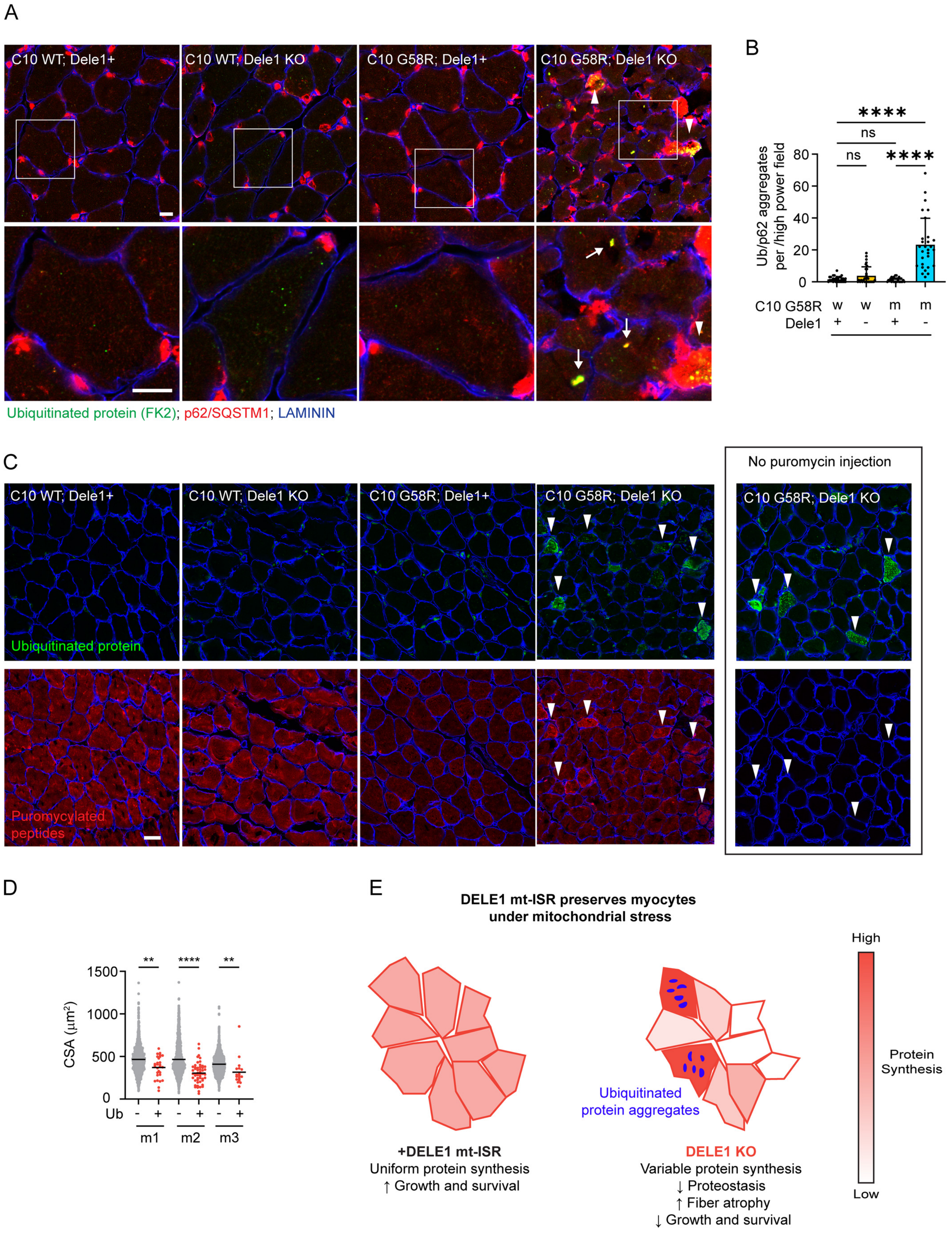
A
C10 WT; Dele1+
C10 WT; Dele1 KO
C10 G58R; Dele1+
C10 G58R; Dele1 KO
Ubiquitinated protein (FK2); p62/SQSTM1; LAMININ
B
Ub/p62 aggregates per /high power field
****
ns
ns
****
C10 G58R w w m m
Dele1 + - + -
C
No puromycin injection
Ubiquitinated protein
Puromycylated peptides
D
CSA (µm²)
Ub
m1
m2
m3
E
DELE1 mt-ISR preserves myocytes under mitochondrial stress
+DELE1 mt-ISR
Uniform protein synthesis
↑ Growth and survival
Ubiquitinated protein aggregates
DELE1 KO
Variable protein synthesis
↓ Proteostasis
↑ Fiber atrophy
↓ Growth and survival
High
Protein Synthesis
Low

Figure EV5. The *Dele1* mt-ISR prevents disruptions in translation-associated proteostasis in skeletal muscle.

(**A**) Representative immunofluorescence images of gastrocnemius muscle from C10 G58R; *Dele1* KO and indicated littermates triple-stained for the ubiquitinated protein marker, FK2 (green), the aggregate adapter protein p62 (red) and laminin (blue). In the C10 G58R; *Dele1* KO genotype, a subset of fibers displayed FK2 and p62 colocalized in individual (white arrows) or confluent aggregates (arrowheads), suggesting proteostatic collapse. High power (60X) views of the boxed areas are shown in bottom panels. Scale bars = 10 μm. Note: animals were not injected with puromycin in this experiment. (**B**) Quantification of (**A**). Aggregates positive for both FK2 and p62 were counted in 10 high power (60X) fields. *N* = 3 mice per genotype with 29 or 30 fields counted total per sample. High-power (60X) field size is 132.58 μm × 132.58 μm. Statistical analysis was performed using the Kruskal–Wallis test with Dunn's multiple comparisons test, as the data distribution was non-parametric. **** indicates $p \leq 0.0001$ and "ns" not significant. Adjusted *p*-values are >0.9999 for WT vs. *Dele1* KO, >0.9999 for WT vs. C10 G58R, <0.0001 for WT vs. C10 G58R; *Dele1* KO, and <0.0001 for C10 G58R vs. C10 G58R; *Dele1* KO. (**C**) Representative immunofluorescence images of gastrocnemius muscle from P28 C10 G58R; *Dele1* KO mice and indicated littermates injected with puromycin 30 min prior to sacrifice as in (Fig. 7E). Muscle cross-sections were immunostained for the ubiquitinated protein marker, FK2 (green), puromycin (red), and laminin (blue). Arrowheads indicate muscle fibers containing many or confluent aggregates of ubiquitinated protein that were also co-stained for elevated puromycylated polypeptides. *N* = 1 mouse for each genotype except for P28 C10 G58R; *Dele1* KO mice for which *N* = 3 mice. Scale bars = 20 μm. (**D**) Quantification of myofiber cross-sectional area (CSA) as in (Fig. 7E). The average CSA for Ub+ and Ub- myofiber is shown in graph separately for three G58R; *Dele1* KO mice (m1–3) in graph. *N* = 3 mice with 10 high power fields counted per mouse. Statistical analysis was performed using the Kruskal–Wallis test with Dunn's multiple comparisons test, as the data distribution was non-parametric. ** and **** indicates $p \leq 0.01$ and 0.0001, respectively, and "ns" not significant. *P*-values (from left to right) are 0.0055, <0.0001, and 0.0016. (**E**) Schematic summarizing data showing that *Dele1* KO results in variable protein synthesis, decreased proteostasis, and increased muscle fiber atrophy within skeletal muscle undergoing mitochondrial stress. We hypothesize that these changes are responsible for the decreased growth and survival in MM models with early mitochondrial stress, in the absence of the *Dele1* mt-ISR.

 This is a U.S. Government work and not under copyright protection in the US; foreign copyright protection may apply
